# Unravelling strong temperature-dependence of *J*_HD_ in transition metal hydrides: solvation and non-covalent interactions *versus* temperature-elastic H–H bonds†

**DOI:** 10.1039/d3sc04197b

**Published:** 2023-10-13

**Authors:** Alexey V. Polukeev, Silvia C. Capelli, Ola F. Wendt

**Affiliations:** a Centre for Analysis and Synthesis, Department of Chemistry, Lund University PO Box 124 22100 Lund Sweden alexey.polukeev@chem.lu.se; b ISIS Neutron and Muon Source, Rutherford Appleton Laboratory Harwell Science Campus Didcot OX11 0QX UK

## Abstract

A number of transition metal hydrides reveal intriguing temperature-dependent *J*_HD_ in their deuterated derivatives and possibly the temperature dependent hydrogen–hydrogen distance (*r*(H–H)) as well. Previously, theoretical studies rationalized *J*_HD_ and *r*(H–H) changes in such compounds through a “temperature-elastic” structure model with a significant population of vibrational states in an anharmonic potential. Based on the first variable temperature neutron diffraction study of a relevant complex, (*p*-H-POCOP)IrH_2_, observation of its elusive counterpart with longer *r*(H–H), crystallized as an adduct with C_6_F_5_I, and thorough spectroscopic and computational study, we argue that the model involving isomeric species in solution at least in some cases is more relevant. The existence of such isomers is enabled or enhanced by solvation and weak non-covalent interactions with solvent, such as halogen or dihydrogen bonds. “Non-classical” hydrides with *r*(H–H) ≈ 1.0–1.6 Å are especially sensitive to the above-mentioned factors.

## Abstract 2

Temperature-dependent *J*_HD_ in (X–POCOP)IrH_2_ complexes is rationalized *via* equilibrium between “short” and “long” isomers, both characterized by neutron diffraction. The effects of specific and non-specific solvation on (X–POCOP)IrH_2_ are discussed.

## Graphical abstract

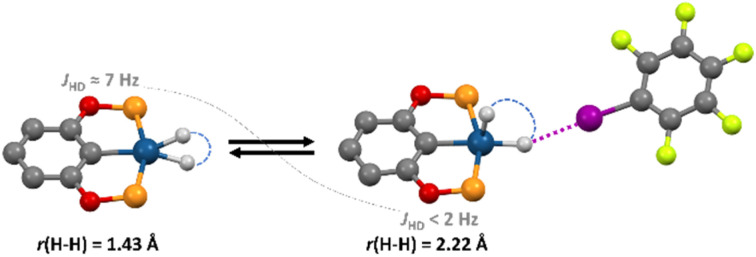

## Introduction

Transition-metal hydrides are involved in a countless number of reactions and catalytic cycles^1^ and are of fundamental importance to organometallic chemistry.^2^ More specifically, dihydrides, since they can be formed through direct reaction of metal centers with molecular hydrogen, are highly relevant to the processes of hydrogen activation and its extrusion from hydrogen-rich molecules, not the least for the purpose of hydrogen storage.^3^ Dihydrides also represent the simplest case of oxidative addition/reductive elimination^4^ and thus serve as model compounds to study the interaction of transition metal centers with other molecules. In particular, some similarity of H–H and C–H bond activation should be mentioned,^5^ and while C–H bond activation intermediates are often elusive, the respective dihydrides usually have much higher stability.^6^

**Fig. 1:**
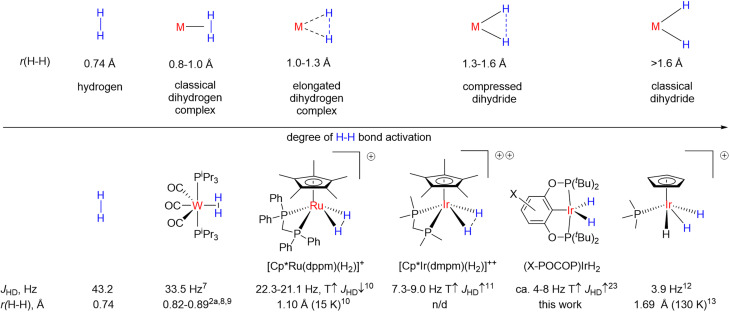
Top: H–H bond activation continuum in transition metal hydrides. Bottom: a few examples of hydride complexes including those with temperature-dependent *J*_HD_, preferably where H–H bond distance determination *via* neutron diffraction has been done.

It is believed that dihydrides form an H–H bond activation continuum^2^ as illustrated in Fig. 1.^7–13^ It begins with classical dihydrogen complexes where the H–H bond acts as a Lewis base and remains comparatively intact and ends up with classical dihydrides where oxidative addition is finalized.^2^ In the middle are the so-called elongated dihydrogen complexes and compressed dihydrides. It follows from Fig. 1 that the H–H internuclear distance, *r*(H–H), is one of the key descriptors of dihydrides. The experimental determination of *r*(H–H) is challenging: X-ray diffraction frequently fails to locate hydrides nearby to a heavy metal atom, while neutron diffraction, which would be the ideal technique, requires growing relatively large crystals, which most of the time is difficult with these sensitive materials. Common spectroscopic characterization methods include solution-state determination of *T*_1_(min)^14^ and *J*_HD_,^15,16*d*^ both of which are correlated to *r*(H–H). A number of complexes were discovered, which reveal puzzling temperature-dependent *J*_HD_^11,16^ (see Fig. 1, bottom); the examples include [Cp*Ir(dmpm)(H_2_)]^++^ (CD_2_Cl_2_, 7.3–9 Hz),^16*c*–*e*^*cis*-[Cp*Ru(dppm)(H_2_)]^+^ (CD_2_Cl_2_, 21.1–22.3 Hz),^10,16*a*,*b*^*cis*-Cp(CO)_2_ReH_2_ (5.8–6.5 Hz, toluene-*d*_8_),^16*f*^*trans*-[Os(H_2_)Cl(dppe)_2_]^+^ (13.6–14.2 Hz, CD_2_Cl_2_)^16*g*,*h*^ and a few more compounds.^17^ If one were to straightforwardly apply the *J*_HD_–*r*(H–H) correlation, it would seem that *r*(H–H) in such complexes is changing with temperature as well. Initially, this pattern was attributed to a rapid dihydrogen–dihydride equilibrium.^16*f*,18^ However, over the years convincing spectroscopic or crystallographic evidence for the presence of two compounds was never obtained. At least one of the plausible isomers always remained elusive and highly uncertain, which, along with the limited datasets available, precluded quantitative analysis attempts.^16*c*^

In an attempt to resolve this puzzling case, theoretical studies suggested the existence of unusual “temperature elastic” H–H bonds.^16*e*,*f*,19–21^ The model involved a single structure with a highly anharmonic potential energy surface, which gives rise to excited vibrational states complementary to a ground state: if the ground state is a compressed dihydride, the excited vibrational states would be mainly of elongated dihydrogen complex nature with a shorter H–H distance, and *vice versa*. The population of those states leads to a change of *r*(H–H); elongated dihydrogen complexes were predicted to have a longer *r*(H–H) and lower *J*_HD_ upon an increase in temperature, while compressed dihydrides should have shorter *r*(H–H) and higher *J*_HD_ at higher temperature^20^ – which on a semi-quantitative level is in line with the majority of experimental observations.

Both theoretical methods and *J*_HD_*–r*(H–H) correlations suggested that for certain compounds such as [Cp*Ir(dmpm)(H_2_)]^++^ and [Cp*Ru(dppm)(H_2_)]^+^ (Fig. 1) where *J*_HD_ variation reaches 1–2 Hz, *r*(H–H) values may change up to 0.02–0.08 Å over 100–300 K – the value that potentially can be detected *via* crystallographic methods. However, all relevant neutron diffraction studies were conducted at a single temperature, and no accurate experimental verification of *r*(H–H) changes with temperature was so far obtained. Furthermore, both *J*_HD_–*r*(H–H) correlation^22,23^ and the *T*_1_(min) method^14,24^ could suffer from complications or data scattering.

Iridium pincer complexes of the type (X–POCOP)IrH_2_ (POCOP = 2,6-(^*t*^Bu_2_PO)_2_C_6_H_3−*x*_-X, where X = *p*-MeO–, *p*-H–, *p*-MeOOC and X_2_ = *m*-bis-CF_3_ in this work) arguably revealed the so far highest reported *J*_HD_ variation of up to 3 Hz (ref. 25–27) (see also Table S4†) over just 50–100 K temperature span. Hence, if *r*(H–H) indeed does change with temperature, the magnitude of such changes in (X–POCOP)IrH_2_ type complexes, judging from *J*_HD_, makes them promising candidates for a crystallographic study.

Here we report the first multi-temperature neutron diffraction study of the compressed dihydride (*p*-H-POCOP)IrH_2_, as well as extensive spectroscopic and theoretical studies of this and related compounds. In a solid state, a small lengthening of *r*(H–H) was observed, as opposed to a considerable shortening that was expected form solution-state data and previous theoretical studies on compressed dihydrides. We argue that the major component that contributes to the *J*_HD_ change in solution is an equilibrium between “short” and “long” isomers of (X–POCOP)IrH_2_. Strong evidence for the existence of such isomers is presented. The solvent choice has a remarkable effect on the spectroscopic properties of (X–POCOP)IrH_2_, such as chemical shift of hydrides, isotope effect on chemical shifts (Δ*δ*), *J*_HD_ and *T*_1_(min). Due to a fairly flat potential energy surface in the H–H bond stretching region, weak interactions with solvent can significantly change the nature and equilibria between various hydride species in solution. A notable interaction is halogen bonding between hydrides and halogenated solvents. This bonding type was characterized including the first neutron diffraction study of a “long” isomer exemplified by a (*p*-MeOOC-POCOP)IrH_2_⋯IC_6_F_5_ adduct. Re-examination of literature data suggests that the model with isomers could be relevant to many hydride complexes.

## Results and discussion

### Solid-state structure of (*p*-H-POCOP)IrH_2_

**Fig. 2:**
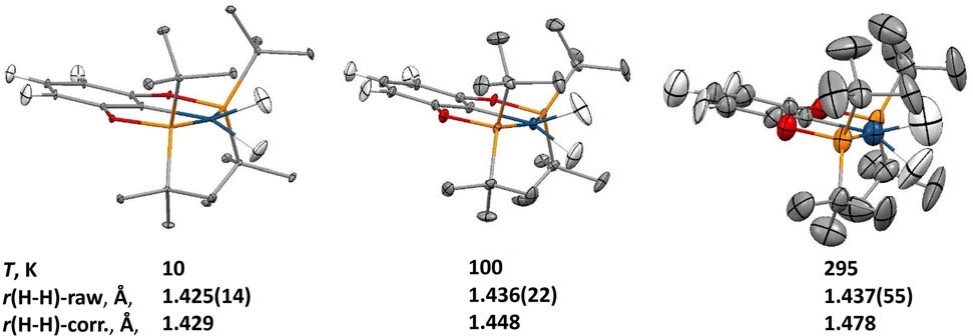
Crystal structure of the (*p*-H-POCOP)IrH_2_ compound at 10, 100 and 295 K as measured with neutron diffraction, reporting also the H–H bond distance (top row: experimental and bottom row: corrected for libration). Ellipsoids are represented at the 50% probability level, and hydrogen atoms of the ^*t*^Bu groups are omitted for clarity.

Within the family of (X–POCOP)IrH_2_ compounds, (*p*-H-POCOP)IrH_2_ was found to crystallize more readily than the other members and therefore was chosen for a neutron diffraction study. Data were collected at 10, 100 and 295 K. The analysis of the neutron diffraction results showed a raw crystallographic *r*(H–H) distance of 1.43(2) Å at 10 K, which remained virtually unchanged at 100 and 295 K (Fig. 2), in contrast to what is expected from solution-state measurements. At 295 K, the anisotropic displacement parameters (ADPs) for the dihydride hydrogen atoms were too big to be only due to intramolecular bending and stretching modes, and a normal coordinate analysis of the ADPs using the Bürgi-Capelli method^28^ was performed (ESI 1.4†). According to such analysis, an in-phase libration of the two dihydride hydrogens out of the ligand molecular plane, coupled with the rigid-body libration of the whole molecule about an axis passing through the P1–P2 atoms, was shown to have a frequency of 51(3) cm^−1^, and this combined librational motion accounted for most of the motion of these hydrogens in the crystal.

It is well known that libration in the solid state can affect the interatomic distances determined in diffraction experiments,^29^ and a correction of the bond lengths based on the librational parameters extracted from the ADP analysis was performed, and these corrected values show an elongation of the *r*(H–H) distance of 0.05 Å in the 10 to 295 K interval. Overall, the changes in the distance are small and in the opposite direction compared to what has been predicted for compressed dihydrides.^18^

### Structure of (X–POCOP)IrH_2_ and (PCP)IrH_2_ in toluene solution. The effect of non-specific solvation

**Fig. 3:**
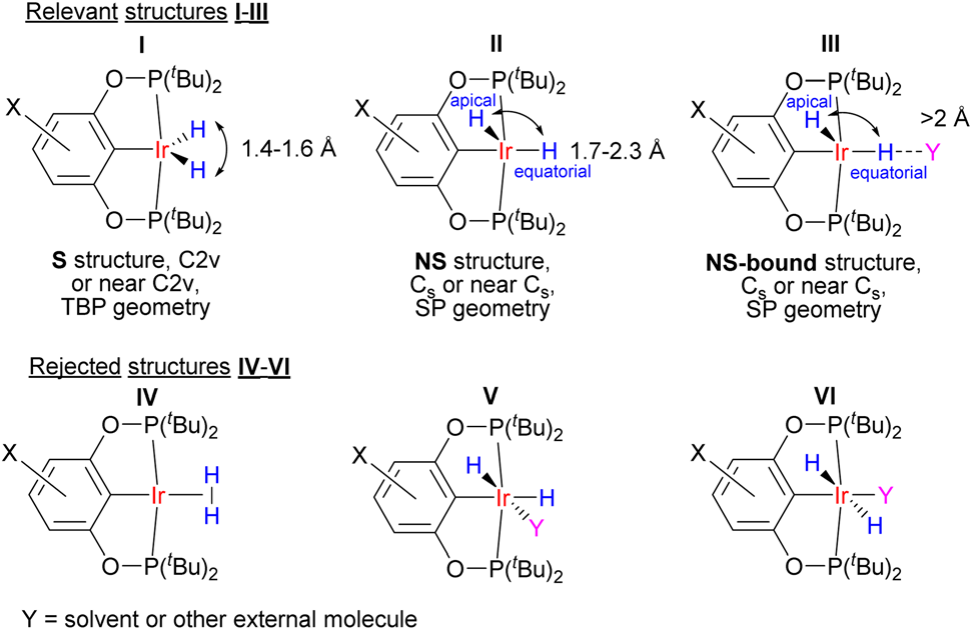
Structures that are involved in the description of (X–POCOP)IrH_2_ in solution. TBP refers to trigonal-bipyramidal and SP to square-pyramidal.

Our neutron diffraction study seems to rule out a high-magnitude *r*(H–H)–*T* dependence in a single compound, and we therefore explored an alternative two-component model. Previous observations^25–27^ on (X–POCOP)IrH_2_ did not rationalize the complex spectral patterns correctly. One proposal, in order to explain the solvent and temperature dependence of *J*_HD_, involved an equilibrium between the putative elongated dihydrogen complex of Ir(i) and an Ir(iii) dihydride with Ir-coordinated solvent (structures IV and V in Fig. 3).^23^ However, such a proposal suffers from multiple discrepancies (for example, it contradicts the data on the chemical shifts of type V structures^30–32^) and illustrates that without understanding the crucial role of solvation and non-covalent interactions, temperature-dependent *J*_HD_ in transition metal hydrides cannot be properly addressed. The present comprehensive NMR data set (Table S4†) for (X–POCOP)IrH_2_ and the related complex (PCP)IrH_2_ (PCP = 2,6-(^*t*^Bu_2_PCH_2_)_2_C_6_H_3_), together with crystallographic and computational data, allowed coming up with a quantitative model based on the discrete isomers with different *r*(H–H). The model involves two structures, symmetrical (S) and non-symmetrical (NS) with respect to the position of hydride ligands (Fig. 3), and can be further augmented with specific interactions with solvent (NS-bound, see below). All solution spectra are hence treated as the weighted-average of S and NS.

**Fig. 4:**
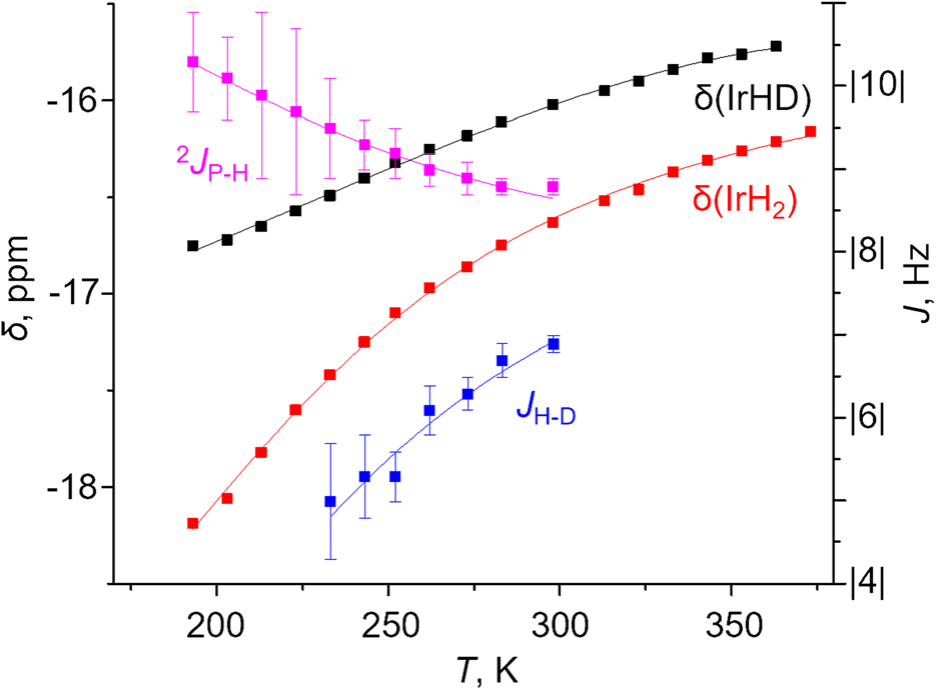
Experimental (squares) and fitted (lines) *δ*(IrH_2_), *δ*(IrHD), ^2^*J*_PH_ and *J*_HD_ for the complex (*m*-bis-CF_3_–POCOP)IrH_2_ in toluene-*d*_8_.

A two-component (S and NS) fit was found to well capture the temperature dependence of *δ*(^1^H), *δ*(^31^P), ^2^*J*_PH_ and *J*_HD_ for X = *m*-bis-CF_3_– and *p*-MeOOC– (Fig. 4, ESI 3†), and provided limiting chemical shifts and coupling constants for S and NS, as well as thermodynamic parameters (Table S6†). Pleasingly, the fitted *J*_HD_–S of *ca.* 9.6 Hz matched the one calculated for (*p*-H-POCOP)IrH_2_ using the neutron diffraction distance (8.9 Hz; equation^16*d*^) very well. Hence, *r*(H–H) for S in solution is close to the crystallographically determined value of 1.43 Å. As for the NS structures, *r*(H–H) for (*m*-bis-CF_3_–POCOP)IrH_2_ and (*p*-MeOOC–POCOP)IrH_2_ can be estimated to be 1.7 < *r*(H–H) < 2.0 Å, based on the observed *T*_1_(min) (weighted-averaged) and fitted *J*_HD_–NS. The limiting ^2^*J*_PH_ for S structures (*e.g.* 7.6 Hz for X = *m*-bis-CF_3_–) are close to that for (*p*-H-POCOP)IrH_2_ (8.3 Hz), pointing to a TBP geometry, while for NS structures ^2^*J*_PH_ (*e.g.* 11.4 Hz for X = *m*-bis-CF_3_–) approach that of square-pyramidal^33^ complexes (X–POCOP)IrHCl (*ca.* 13 Hz). S and NS differ by 1–2 kcal mol^−1^ with NS being a global minimum in solution.

As seen from Fig. 4 one aspect of the model is huge isotope effects on chemical shifts (defined as *δ*(IrH_2_) − *δ*(IrHD); Δ*δ*) of up to −1.4 ppm or −5 ppm in other solvents. These are only consistent with the presence of strongly discriminated hydride sites in the molecule. We interpret it as a non-statistical distribution of deuterium between apical and equatorial positions in NS (isotope perturbation of equilibria). To correctly fit Δ*δ*–*T* simultaneously with other parameters, one needs to balance between refining the limiting shifts and assigning “intrinsic” Δ*δ* to S and NS. A possible, but not unequivocal solution is given in Fig. 4; fit parameters and their comparison with DFT calculated values are provided in Table S8.† We would like to note that the accuracy of *J*_PH_ and *J*_HD_ measurement is strongly affected by the linewidth, which may in turn affect the fit; more discussion is provided in ESI 3.1.†

For (X–POCOP)IrH_2_ with electron-donating groups (X = *p*-MeO– and *p*-H–), the temperature dependence of *δ*(^1^H), ^2^*J*_PH_ and *J*_HD_ is much smaller. Thus, the change of *δ*(IrH_2_) over −80, …, +25 °C is only 0.4 ppm. This likely reflects smaller geometrical and energetic differences between S and NS. Therefore, S–NS equilibria changes are hard to differentiate from “non-specific” processes such as P-^*t*^Bu group rotations *etc. T*_1_(min) for X = *p*-MeO– and *p*-H– (129 and 120 ms) can be translated to an *r*(H–H) of 1.57 and 1.54 Å, respectively, using the established methodology.^14^ We therefore assume that the complexes with X = *p*-MeO– and *p*-H– in solution closely resemble the neutron diffraction structure, with the *T*_1_(min) based distances viewed as an upper limit for *r*(H–H) of S (for S-(*p*-H-POCOP)IrH_2_ 1.43 < *r*(H–H) < 1.54 Å). ^2^*J*_PH_, and to some extent, *J*_HD_ changes are obscured by line broadening. Yet, the fitted *J*_HD_–NS is consistent with an *r*(H–H) of around 1.7 Å for NS. Isotope effects Δ*δ* do not exceed 0.22 ppm, providing further evidence of nearly symmetrical hydride sites (the geometry is closer to TBP rather than SP in both S and NS). Notably, Δ*δ* for complexes with X = *p*-MeO– and *p*-H– in toluene at certain temperatures reveals *δ*(IrHD) up-field *versus δ*(IrH_2_), which was not observed for X = *m*-bis-CF_3_– and *p*-MeOOC– (Table S4†). This may be an observation of an “intrinsic” Δ*δ* in S. The temperature dependence of Δ*δ* then can be due to intermolecular isotope perturbation of S–NS equilibria with deuterium favoring NS or due to S this time being a global minimum (ESI 4†). In other solvents, where NS has longer *r*(H–H) and is undoubtfully populated, isotope effects for X = *p*-MeO– and *p*-H– are in the same direction (although smaller) as for *m*-bis-CF_3_– and *p*-MeOOC–.

Finally, the complex (PCP)IrH_2_ (ref. 34) reveals NMR spectra with very minor temperature dependence of chemical shifts and *J*_HD_ (Fig. S2, Table S4†). The *J*_HD_ of 7.6 Hz corresponds to an *r*(H–H) of 1.49 Å, in good agreement with 1.49 Å obtained from a *T*_1_(min) of 94 ms. Supposedly, for (PCP)IrH_2_NS is higher in energy than S, and is not populated, giving rise to static spectra.

### Computational study of (X–POCOP)IrH_2_ and (PCP)IrH_2_ in toluene solution

**Fig. 5:**
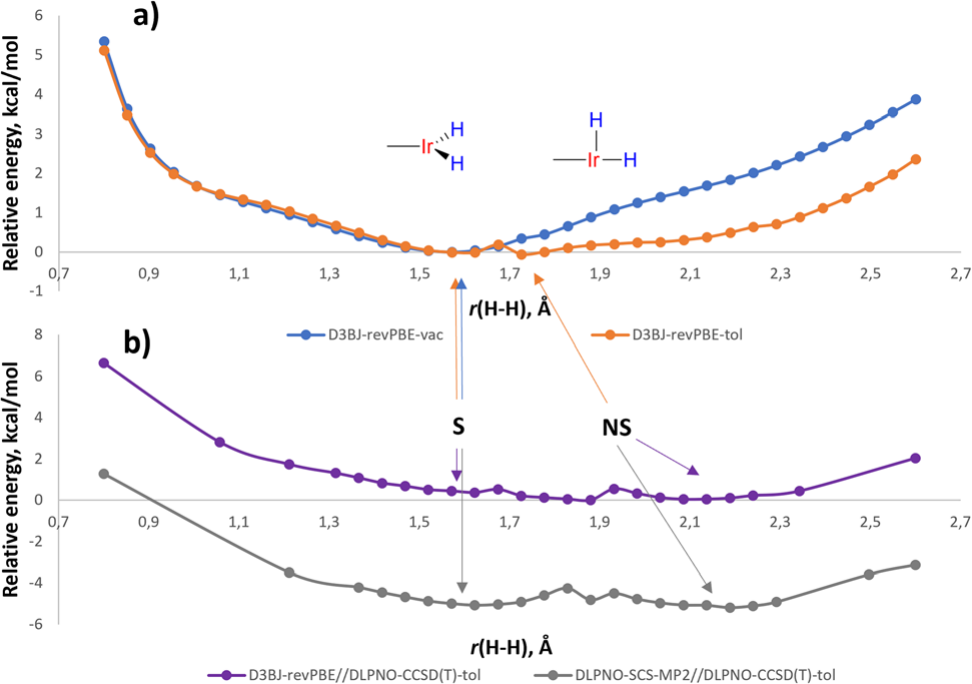
(a) Potential energy surface for (p-H-POCOP)IrH_2_ in a vacuum as well as in a solution of toluene using D3BJ-revPBE method; (b) the same PES at using D3BJ-revPBE//DLPNO-CCSD(T) and DLPNO-SCS-MP2//DLPNO-CCSD(T) methods in toluene. Energies are given in kcal mol^−1^ and normalized to zero, except for the DLPNO-SCS-MP2//DLPNO-CCSD(T) profile that is normalised *versus* D3BJ-revPBE//DLPNO-CCSD(T).

For LL′L′′MX_2_ type d^6^ complexes, the TBP geometry with a 120° MX_2_ angle is disfavored and due to Jahn-Teller effects undergoes angle compression.^35^ Indeed, the potential energy surface (PES) of (*p*-H-POCOP)IrH_2_ in the *r*(H–H) stretching region (D3BJ^36^-revPBE^37,38^ level of theory, which was noted to perform well for Ir^39^) in a vacuum reveals only an S structure (Fig. 5) with “distorted” TBP geometry. However, there is another distortion that can remove the degeneracy, which is the non-symmetrical movement of hydride ligands to give NS. When solvation is included, NS, which has a higher dipole moment, receives extra stabilization and appears as a separate minimum on the PES. Thus, with toluene set as a solvent (CPCM model)^40^ two minima are found, corresponding to S and NS structures at 1.60 and 1.63 Å, respectively (Fig. 5).

A single-point correction of electronic energies using a highly accurate DLPNO-CCSD(T)^41^ method resulted in a quite similar energy profile, with NS shifted to 2.1 Å. We have also attempted other DFT functionals and basis sets (ESI 9.2†), and the majority of methods argue for the distance in S between 1.48 and 1.65 Å in (*p*-H-POCOP)IrH_2_. That is slightly longer that the 10 K neutron diffraction distance of 1.43(2) Å. Possibly the difference reflects uncaptured solvation/packing effects, but we cannot completely rule out other reasons. The direction of the least energetic cost upon deformation of *r*(H–H) in S is towards longer distances, which may explain the small elongation upon raising the temperature observed by neutron diffraction.

When it comes to locating NS on the PES, depending on the method, *r*(H–H) for NS in (*p*-H-POCOP)IrH_2_ varies from 1.63 to 2.2 Å, while the energy gap between S and NS goes from +3.0 to −0.3 kcal mol^−1^. For compounds with electron-withdrawing groups X = *p*-MeOOC- and *m*-bis-CF_3_–, the majority of methods indicate that NS–*r*(H–H) ≈ 2.0–2.2 Å and Δ*H*(NS–S) ≈ −1, …, −2 kcal mol^−1^, which is consistent with experimental observations. For X = *p*-MeO–, NS appears higher in energy than S by *ca.* 0.3–2 kcal mol^−1^. Since NMR spectra reveal small changes of *δ*(IrH_2_) and *J*_HD_ in the same direction as for the more withdrawing groups, it could be that explicit solvation is needed for a correct description. For (PCP)IrH_2_NS is higher in energy than S (Fig. S31†), in line with the near-static NMR spectra.

**Fig. 6:**
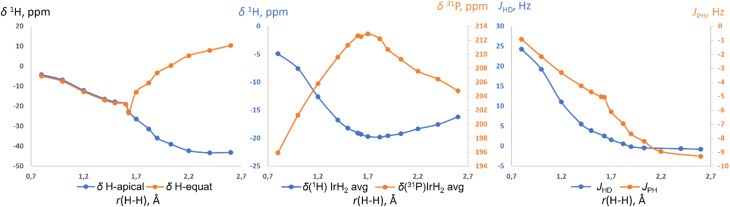
The calculated ^1^H and ^31^P chemical shifts, as well as *J*_HD_ and ^2^*J*_PH_ coupling constants in (*p*-H-POCOP)IrH_2_ as a function of *r*(H–H).

Further support for the two-component model comes from NMR calculations. Previous NMR studies on relevant compounds [Cp*Ir(dmpm)(H_2_)]^++^ (ref. 19*b* and 16*c*) and *cis*-[Cp*Ru(dppm)(H_2_)]^+^ (ref. 19*a*) were performed using non-relativistic approximations and cannot be deemed accurate. Here we used the ReSpect program^42^ to run much more trustworthy fully relativistic four-component DFT calculation of NMR properties,^42^ required for systems with late transition metals.^43^ The evaluation of a test set of compounds revealed an underestimation of the hydride resonances beyond *ca.* −30 ppm. This was noted previously,^43^ and we addressed it by applying a small empirical correction (ESI 10†). The NMR parameters of (*p*-H-POCOP)IrH_2_ as a function of *r*(H–H) are presented in Fig. 6. Upon an increase in the distance between the hydrides their chemical shifts synchronously move up-field. Beyond *ca.* 1.6 Å, the de-symmetrization of the hydride environment causes drastic discrimination of H-apical and H-equatorial. Thus, H-apical eventually moves to *ca.* −40 ppm, just as in the SP (POCOP)IrHCl counterpart, while H-equatorial moves to *ca.* 10 ppm. Hence, the difference between the two hydrides may reach 50 ppm, which explains the large isotope effects on chemical shifts in NS. The averaged *δ*(IrH_2_) goes through a minimum at *ca.* 1.8 A and then starts to increase upon further increase of *r*(H–H). ^31^P chemical shift also has an extreme point at a comparable distance (Fig. 6, middle). *J*_HD_ monotonically decreases from 24 Hz (0.8 Å) to −0.8 Hz (2.6 Å), in line with the empirical data for the *J*_HD_–*r*(H–H) curve.^15,16*d*^ The predicted chemical shifts are −16.8 ± 2 ppm for S (1.4 Å) and −19.7 ± 2 ppm for NS (1.7 Å) for the complex (*p*-H-POCOP)IrH_2_, which agrees well with the data from fitting (−16.0 and −18.0 ppm).

### Solvent effect on the structure of (X–POCOP)IrH_2_. Specific solvation

While a non-specific solvation appears important for stabilization of NS isomers, the pronounced solvent dependence of (X–POCOP)IrH_2_ spectra can nevertheless be linked to a specific solvation. In the study of a related aliphatic (PCyP)IrH_2_ complex,^26^ the authors based on DFT calculations suggested that it may be the solvent relative permittivity *ε* that mainly affects the properties of the complex in solution. Here we designed an experiment to verify this hypothesis. Thus, by adding a soluble electrolyte to an organic solvent, it is possible to vary its relative permittivity with little effect on other properties.^44^ We prepared a solution of (*p*-H-POCOP)IrH_2_ in CD_2_Cl_2_ and THF-*d*_8_, as well as in the same solvents with 0.5 M NBu_4_PF_6_, which is supposed to triple the relative permittivity.^44^ Calculation using CPCM solvent CH_2_Cl_2_ with natural *ε* = 8.9 and with *ε* set to 24.2 predicted that for (*p*-H-POCOP)IrH_2_NS should be favored by an extra 0.2 kcal mol^−1^ for higher *ε*; this is conceptually in line with previous data^26^ that employed the Poisson–Boltzmann^45^ reactive field. However, unlike the control probe with a solvatochromic dye (see ESI 8†), virtually no changes were observed in the NMR spectra of (*p*-H-POCOP)IrH_2_. Calculations thus somewhat overestimate the effect of *ε*. At the same time, a correlation of *J*_HD_ and Δ*δ* with the Gutmann acceptor number of the solvents was observed (Table 1). With that in hand, we added C_6_F_5_I, which has a high acceptor number and low polarity, to a toluene-*d*_8_ solution of (*p*-H-POCOP)IrH_2_, and observed a significant decrease of *J*_HD_ and increase of Δ*δ*. It thus follows that these are weak non-covalent interactions with solvent/dissolved compounds, which are mainly responsible for the spectral changes observed for (X–POCOP)IrH_2_. Notably, hydrides can form a halogen bond (XB)^48^ with the strong XB donor C_6_F_5_I, something that has rarely been observed previously.^49^

**Table 1:** Solvent effect on the NMR spectra of (*p*-H-POCOP)IrH_2_; data at 0 °C for CH_2_Cl_2_ and 25 °C for other solvents

	MeCy-*d*_14_	Toluene-*d*_8_	CD_2_Cl_2_	THF-*d*_8_	CD_2_Cl_2_/NBu_4_PF_6_ (0.5 M)	THF-*d*_8_/NBu_4_PF_6_ (0.5 M)	Toluene-*d*_8_/C_6_F_5_I (20/1)	Toluene-*d*_8_/C_6_F_5_I (1/5)
*ε*	2.02	2.37	8.93	7.43	∼24.2^a^	n/d^b^	n/d^b^	n/d^b^
*J*_HD_, Hz	n/d^b^	6.9	∼5^c^	6.9	∼5^c^	6.9	4.7	n/r^c^
Δ*δ*, ppm	−0.11	−0.22	−0.60	−0.21	−0.60	−0.20	−0.61	−2.41
DN^d^	0^f^	0.1^f^	1.0^f^	20.0^f^	∼1.0^f^	∼20.0^f^	n/d	n/d
AN^e^	0^f^	8.2^f^ (C_6_H_6_)	20.4^f^	8.0^[f]^	∼20.4^f^	∼8.0^f^	27.1^g^ (C_6_F_5_I)	27.1^g^ (C_6_F_5_I)

a Ref. 44.

b Not determined.

c Poorly resolved.

d DN refers to the donor number.

e AN refers to the acceptor number.

f Ref. 46.

g Ref. 47.

**Fig. 7:**
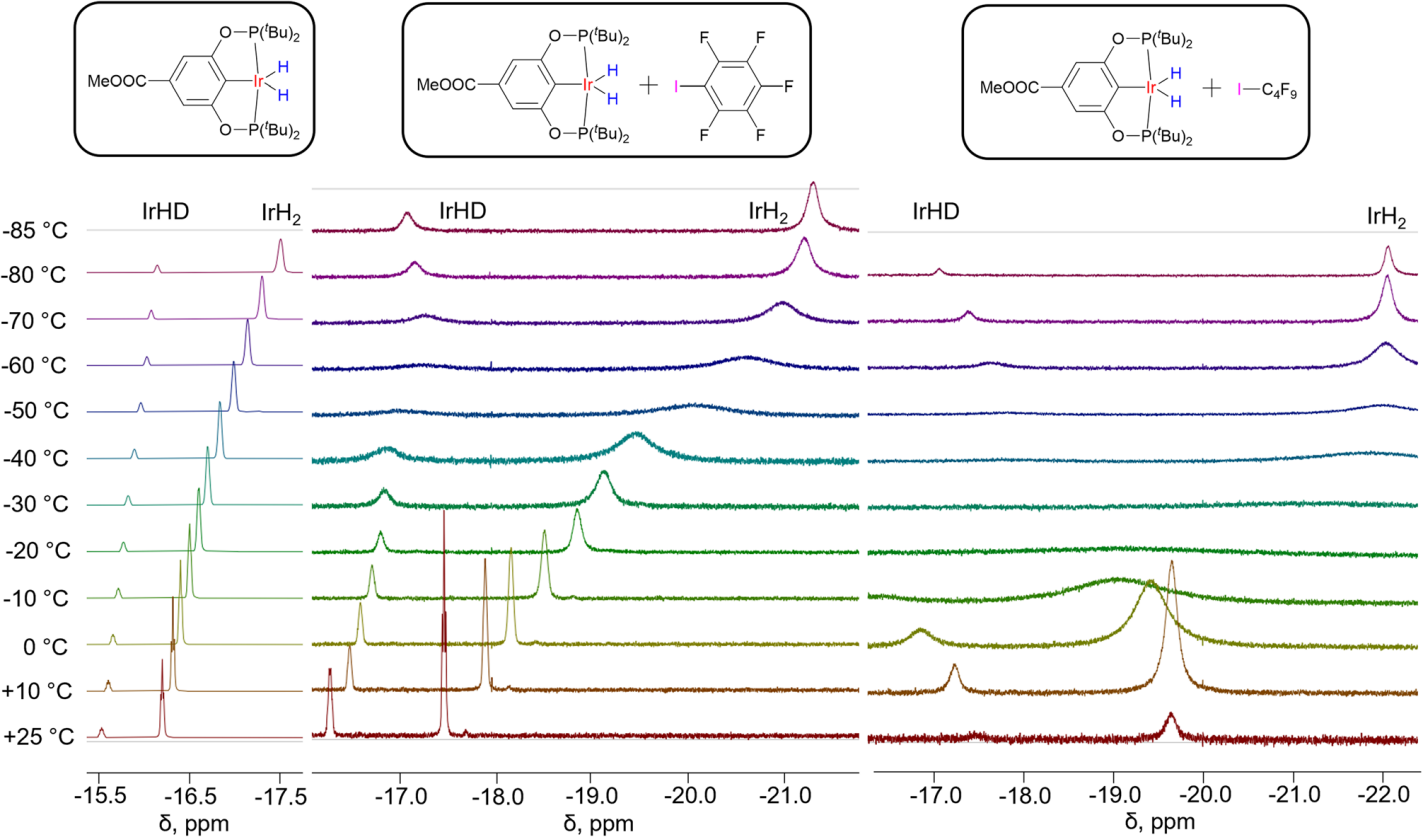
^1^H NMR spectra of partially deuterated (*p*-MeOOC-POCOP)IrH_2_ in toluene-*d*_8_, toluene-*d*_8_/C_6_F_5_I = 10/1 (v/v) and toluene-*d*_8_/C_4_F_9_I = 1/3 (v/v).

In the presence of C_6_F_5_I, (X–POCOP)IrH_2_ complexes undergo an up-field shift of hydride resonances, which is accompanied by a dramatic de-symmetrization for X = *m*-bis-CF_3_– and *p*-MeOOC–, as indicated by the difference between *δ*(IrH_2_) and *δ*(IrHD) exceeding −4 ppm. Even for X = MeO– Δ*δ* reaches −0.9 ppm, pointing towards the substantial presence of NS. Fig. 7 depicts the VT ^1^H NMR spectra of (*p*-MeOOC-POCOP)IrH_2_ in toluene-*d*_8_, as well as with C_6_F_5_I and C_4_F_9_I added. One can note a marked increase in the span of chemical shifts in the presence of C_6_F_5_I, compared to neat toluene-*d*_8_. Also, a broadening of both IrH_2_ and IrHD signals is observed at −50 and −60 °C, which is decreased upon further cooling, indicating freezing out of an exchange process. At −80 °C a new small peak in the ^1^H spectra appears at −7.7 ppm, which we interpret as a formation of the (*p*-MeOOC-POCOP)IrH(IC_6_F_5_)H adduct (calculated *δ* −5.4 ppm). While in the ^1^H NMR spectrum a high field-shift of the IrH_2_ signal is observed, as it was observed for spectra in toluene, the ^31^P NMR signal undergoes a low-field shift upon addition of C_6_F_5_I. To rationalize this the NMR calculations can be re-called (Fig. 6), which predict a decrease of ^31^P NMR shifts upon passing a maximum near 1.7–1.9 Å. Halogen bond adducts have *r*(H–H) ≥ 2.1 Å and hence should reveal low-field ^31^P shifts. Thus, for X = H– calculation gives 196.1 ppm for the ^31^P signal and −40.6 and 1.8 ppm for hydride signals (NS-bound-a). When a stronger halogen bond acceptor C_4_F_9_I is added in a larger amount, initially a low-field shift is observed in ^1^H NMR spectra upon cooling (Fig. 7). At *ca.* −30 °C the ^1^H signals become so broad that they are nearly indistinguishable from the baseline. Upon further cooling signals reappear at −7.76 (IrH(IC_4_F_9_)H) and −22.06 ppm (IrH_2_⋯IC_4_F_9_). Neither resonance shifts below −60 °C, meaning that there is no free (*p*-MeOOC-POCOP)IrH_2_, and only adducts IrH(IC_4_F_9_)H and IrH_2_⋯IC_4_F_9_ in a slow equilibrium are present. Based on changes in the ratio between these two, thermodynamic data can be extracted through the van't Hoff plot (Δ*H* = −5.7 ± 0.4 kcal mol^−1^ and Δ*S* = −25 ± 2 cal × mol^−1^ K^−1^) (Fig. S19†). Also, following the IrHD signal in IrH_2_⋯IC_4_F_9_ allows the measurement of the preference of deuterium to occupy the apical site (Δ*H* = −0.27 ± 0.01 kcal mol^−1^ and Δ*S* = −0.37 ± 0.06 cal × mol^−1^ K^−1^; Fig. S12†), which agrees very well with both fitted and DFT calculated values for C_6_F_5_I (Table S8†).

**Fig. 8:**
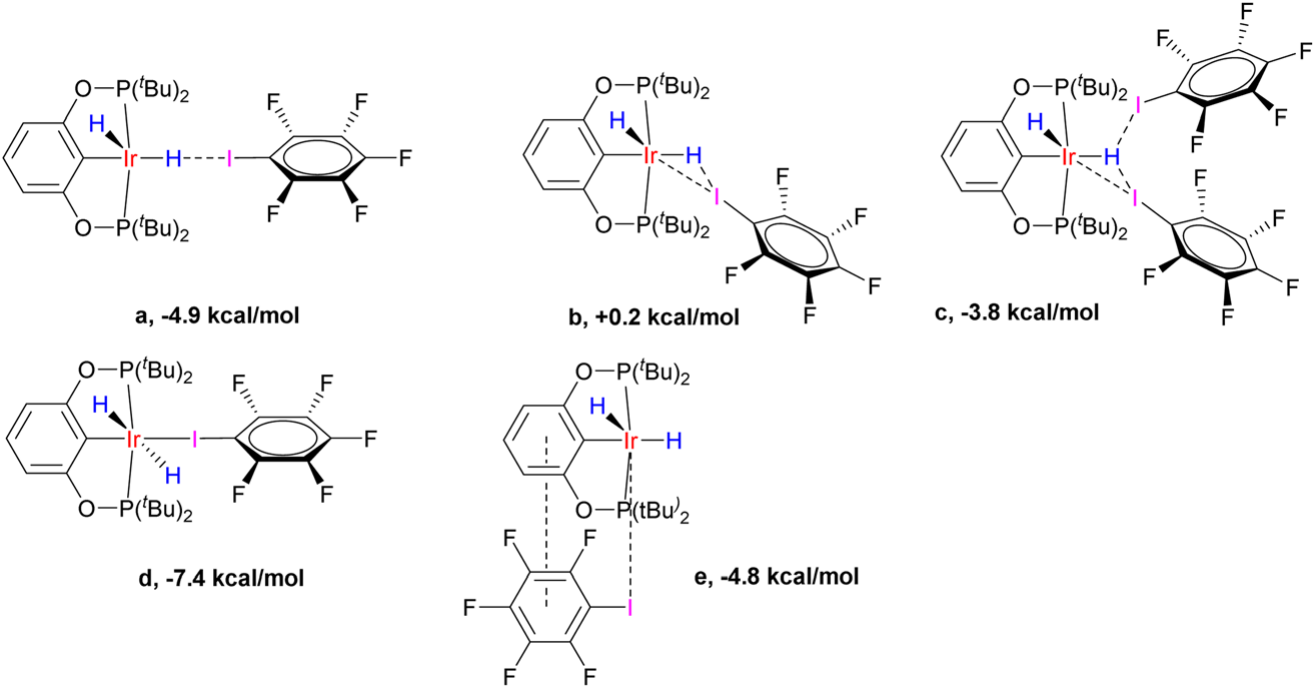
The variety of interaction modes between (*p*-H-POCOP)IrH_2_ and C_6_F_5_I (NS-bound-a, …, e). Interaction enthalpies are given at the D3BJ-revPBE//DLPNO-CCSD(T) theory level.

Computationally, there are several interaction modes of (*p*-H-POCOP)IrH_2_ and C_6_F_5_I (Fig. 8). All energies are given at the DLPNO-CCSD(T) level, which we found necessary to obtain accurate values. This is in line with benchmarks on halogen bonds^50^ that favored wavefunction methods. Structures a, b and c represent different variations of halogen bonding; c can be discarded for entropic reasons. In agreement with the experimental data, a is the favored halogen-bonded form in solution. Out of 18e adducts, d is lower in energy than e, and both are disfavored entropically *versus* a (d by −5.0 and e by −9.5 kcal × mol^−1^ K^−1^). The calculated hydride chemical shifts for e (−6 and −20 ppm; avg. −13 ppm) are in the range expected for the type V structure from Fig. 3, and hence clearly are not related to spectra depicted in Fig. 7, while those for a (−40.6 and 1.8 ppm; avg. −19.4 ppm) are close to fitted values both in terms of average shift (*ca.* −22 ppm) and the very large difference between apical and equatorial hydrides. Thus, computational methods strongly support the conclusion that the formation of a is responsible for the observed spectral changes.

The calculated XB energies for NS-bound-a with different X groups, measured *versus*S, are listed in Table 2. Somewhat counter-intuitively, the halogen bond is stronger for more electron-withdrawing X groups. This is rationalized through reducing a destabilizing interaction between aryl and hydride, which are *trans* to each other in NS and NS-bound (pincer aryl backbones with electron-withdrawing X groups have a smaller *trans*-effect). If the binding energy is measured against the most stable isomer in solution, then (PCP)IrH_2_ with its −5.1 kcal mol^−1^ will provide nearly the strongest interaction. (PCP)IrH_2_ exhibits the highest buildup of negative charge on hydride ligands, enhancing interaction with the σ-hole on iodine atoms. At the same time, such charge buildup disfavors the formation of NS and NS-bound. Thus, a more electron-rich metal center in (PCP)IrH_2_ gives rise to counter-balancing effects. The calculated halogen bond energies for C_6_F_5_I are in agreement with experimental data taking possible uncertainties into account (ESI 6†).

**Table 2:** Experimental and calculated halogen bond strengths for (X–POCOP)IrH_2_ and C_6_F_5_I measured in toluene-*d*_8_ (kcal mol^−1^ and cal × mol^−1^ K^−1^)

Complex	*p*-MeO–	*p*-H–	*p*-MeOOC–	*m*-bis–CF_3_–	(PCP)IrH_2_
H_(e*x*p)_-fit.	−2.6	−3.7	−3.9	−3.7	n/d
S_(e*x*p)_-fit.	−9.7	−12.3	−14.2	−14.1	n/d
H_(e*x*p)_-titr.	n/d	−3.0	n/d	n/d	n/d
S_(e*x*p)_-titr.	n/d	−12.6	n/d	n/d	n/d
H_(calc)_^a^	−3.9	−4.9	−6.7	−6.2	−5.1

a Calculated enthalpies are given *versus* the S structure.

**Fig. 9:**
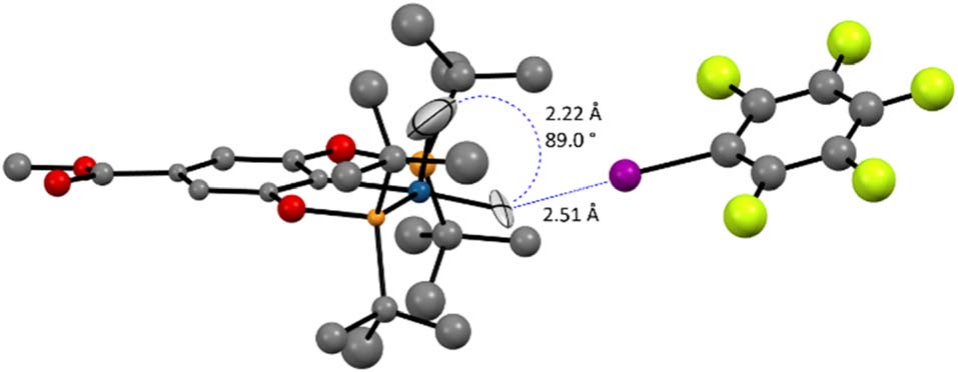
The neutron diffraction structure of the (*p*-MeOOC-POCOP)IrH_2_⋯IC_6_F_5_ adduct at 40 K. Ligand hydrogen atoms are omitted for clarity.

Ultimately, a neutron-diffraction study of a single-crystal of the (*p*-MeOOC-POCOP)IrH_2_⋯IC_6_F_5_ adduct unambiguously confirmed that the dominating compound in the (*p*-MeOOC-POCOP)IrH_2_/IC_6_F_5_ system is NS-bound-a (Fig. 9). The hydrides were clearly located in NS configuration, with *r*(H–H) was measured to be 2.22 Å and *r*(H–I) to be 2.51 Å. Fully in line with computational predictions, the equatorial Ir–H distance is elongated to 1.66(4) Å and the apical Ir–H distance is shortened to 1.52(9) Å, compared to almost identical Ir–H distances in S (1.60(1) and 1.615(8) Å raw; 1.62 Å for both after libration correction). At the same time, D3BJ-revPBE seemingly over-binds the adduct (*r*(H–I)_calc_ = 2.27 Å), and a very expensive DLPNO-SCS-MP2 method was needed to accurately reproduce the experimental *r*(H–I) (Table S12†). Pleasingly, the DLPNO-CCSD(T) halogen bond energies of the two methods were comparable, which allowed examination of a series of complexes as discussed above.

The solid-state IR spectrum of (*p*-MeOOC-POCOP)IrH_2_⋯IC_6_F_5_ revealed a broad band at 1870 cm^−1^ corresponding to a stretching vibration of a halogen-bound Ir–H unit *trans* to the aryl moiety (see ESI 5†). For comparison, a solution of (*p*-MeOOC-POCOP)IrH_2_ in hexane exhibits a band at 2119 cm^−1^ in the hydride region (asymmetric IrH_2_ vibration); a new very broad resonance at *ca.* 1860 cm^−1^ appeared when C_6_F_5_I was added. Thus, the same compounds are present in the solid state and in solution.

The quantification of the halogen bond strength experimentally is difficult, since there are complex equilibria existing in the (X–POCOP)IrH_2_/C_6_F_5_I/toluene system, and is further hampered by decomposition (ESI 6†). The thermodynamic data in Table 2 should therefore be considered to be quite approximate. Variable temperature titration with C_6_F_5_I and fitting to a 1/1 binding isotherm were performed for (*p*-H-POCOP)IrH_2_ and provided Δ*H* = −3.0 kcal mol^−1^ and Δ*S* = −12.6 cal × mol^−1^ K^−1^. Also, the fitting of *δ*(IrH_2_)–*T* dependence in the presence of a constant amount of C_6_F_5_I (S/NS/NS-bound model) was attempted for all X groups and provided comparable results, with the trend resembling the calculated one (see Fig. S16† for *δ*(IrH_2_)–*T* and van't Hoff plots). Measurements for (PCP)IrH_2_ were precluded by a rapid reaction with C_6_F_5_I at rt and the formation of the IrH(C_6_F_5_I)H adduct at low temperatures (consistently, the calculated Δ*H* of formation of IrH(C_6_F_5_I)H is −7.4 kcal mol^−1^ for (*p*-H-POCOP)IrH_2_ and −11.8 kcal mol^−1^ for (PCP)IrH_2_).

**Fig. 10:**
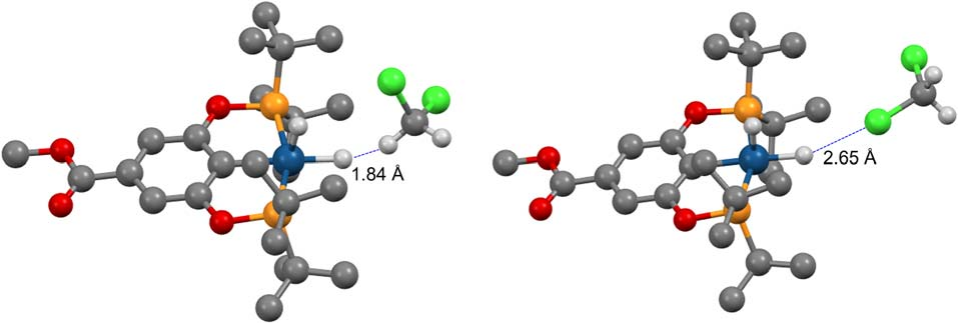
Halogen (right) and dihydrogen (left) bonds between (*p*-MeOOC-POCOP)IrH_2_ and CH_2_Cl_2_ at the D3BJ-revPBE level of theory.

We then investigated (X–POCOP)IrH_2_ in CH_2_Cl_2_, which is a weaker halogen bond donor compared to C_6_F_5_I. The experimental NMR data are given in Table S4 and ESI 7.† A strong *δ*(IrH_2_)–*T* dependence was observed, and isotope effects on chemical shift clearly indicated that X = *p*-MeOOC–, *m*-bis-CF_3_– and *p*-H– in CH_2_Cl_2_ are de-symmetrized (Δ*δ* up to −2.7 ppm), while (*p*-MeO-POCOP)IrH_2_ is not (Δ*δ* up to −0.12 ppm). It is important to note that the comparison of CH_2_Cl_2_ with THF further supports the non-covalent nature of binding with solvent, as compared to coordination to the vacant, but strongly hindered site. Thus, THF is a well-established coordinating solvent, while CH_2_Cl_2_ coordinates to metals rarely and weakly.^51^ NMR spectra in CH_2_Cl_2_ resemble observations with C_6_F_5_I, with both compounds having a high AN, while NMR spectra in THF resemble observations in toluene (Tables 1 and S4†), with both compounds having a low AN. Therefore, we re-iterate that in the given examples the AN, not the DN linked to O and Cl lone pairs, is correlated to the appearance of spectra. Computationally, S structures are virtually unchanged in CH_2_Cl_2_, while NS has longer *r*(H–H) and lower energies, *i.e.* for NS–(*p*-H-POCOP)IrH_2_*r*(H–H) is 1.63 Å in toluene and 2.05 Å in CH_2_Cl_2_, and the S–NS gap is −0.06 and −0.4 kcal mol^−1^, respectively (D3BJ-revPBE). There are numerous modes of interaction between CH_2_Cl_2_ and (X–POCOP)IrH_2_ (ESI 9.2†). It appears that the structure with a dihydrogen bond between the acidic CH_2_Cl_2_ hydrogen and Ir–H is favored (Fig. 10) over the halogen bond between CH_2_Cl_2_ chlorine and Ir–H (Δ*E* −2.3 kcal mol^−1^*vs.* −1.0 kcal mol^−1^, X = H). *T*_1_(min) data (see Table S4;† for example, 463 ms for (*p*-MeOOC-POCOP)IrH_2_ in CH_2_Cl_2_) require that both *r*(H–H) and *r*(IrH⋯H–CHCl_2_) in NS-bound are above 2.0 Å. It implies that D3BJ-revPBE slightly over-binds the adduct with CH_2_Cl_2_, just as it was observed for C_6_F_5_I; alternatively, a high weight of free NS that satisfies the 2.0 Å criteria can be proposed. Fitting to the S/NS-bound model well accounts for *δ*(^1^H), ^2^*J*_PH_ and *J*_HD_ data (ESI 7†); free NS, if present in the system, thus cannot be evaluated. The experimental binding energy estimates are between −1.3 (X = *p*-MeO–) and −2.3 (X = *m*-bis-CF_3_) kcal mol^−1^ (ESI 7†), computational values span from −1.7 to −3.9 kcal mol^−1^ (Table S10†). It thus follows that the dihydrogen bond with CH_2_Cl_2_ is *ca.* 2–4 kcal mol^−1^ weaker than the halogen bond with C_6_F_5_I, which is line with the AN of the solvents (Table 1). On a structural level, this difference is reflected by the significant de-symmetrization of NS-bound in (X–POCOP)IrH_2_ with X= *p*-MeO– by C_6_F_5_I, but not by CH_2_Cl_2_. This can be seen from the Δ*δ* values (−0.9 *vs.* −0.12 ppm) and calculated *r*(H–H) (2.01 *vs.* 1.73 Å) for C_6_F_5_I/toluene and CH_2_Cl_2_, respectively. At the same time, the halogen bond with CH_2_Cl_2_ is weaker than both XB with C_6_F_5_I and the dihydrogen bond with CH_2_Cl_2_; this is in line with the smaller *δ*-hole on Cl compared to I.

To our knowledge, dihydrogen bonds involving CH_2_Cl_2_ and transition metal hydrides are scarce, if at all known. There is an example of an interaction with CH_2_Cl_2_ that is transmitted to hydrides indirectly through binding of CH_2_Cl_2_ with a counter-anion (see below).

### Model with isomers: re-examination of [Cp*Ru(dppm)(H_2_)]^+^

Overall, the S/NS/NS-bound model provided satisfactory rationalization of experimental spectra in different solvents, in particular *J*_HD_ values, without the use of vibrational averaging corrections. It could be that such corrections may further improve fits, but at least for (X–POCOP)IrH_2_ they are not the primary reason of *J*_HD_ change and, according to VT neutron diffraction data, might have an opposite direction (elongation of *r*(H–H) instead of shortening). It is noteworthy that the ^1^*J*_HD_ change with temperature reported for gaseous HD is an order of magnitude lower than the changes reported for hydrides.^52^ There remains some uncertainty regarding the value of vibrational corrections for hydride complexes, since relevant calculations exploited truncated models and were done without solvation. However, it could be that the largest *J*_HD_ changes reported (>1–1.5 Hz over 50–80 K) are due to equilibrium between two or more species. Looking from that angle, the strongest temperature dependence of *J*_HD_ was observed in [Cp*Ir(dmpm)(H_2_)]^++^ and [Cp*Ru(dppm)(H_2_)]^+^. The complex [Cp*Ir(dmpm)(H_2_)]^++^ actually has two minima on the PES;^16*d*,*e*,21^ while initial studies exploited averaging over many vibrational states,^16*d*,*e*^ later it was suggested that a simple average over the two minima could possibly account for the majority of *J*_HD_ change.^21^ The agreement with experimental data was at best semi-quantitative; however we suppose it could be improved by taking solvent effects into account. Another complex [Cp*Ru(dppm)(H_2_)]^+^, was deemed worth re-examination. When the non-truncated version of this compound was used, and the CH_2_Cl_2_ solvation included, the two minima were present at 1.06 and 1.42 Å, respectively (ESI 11†). The former distance is close to the one determined in the solid state by neutron diffraction (1.10 Å).^10^ A reasonable *J*_HD_ fit can be constructed using that data, with the fitted Δ*H* and Δ*S* being in excellent agreement with the values reported previously for a slow-regime dihydrogen–dihydride equilibrium.^53^ Remarkably, the chemical shift of the coordinated H_2_ unit in [Cp*Ru(dppm)(H_2_)]^+^ has little temperature dependence, which was viewed as an argument for the model with vibrational corrections.^16*b*^ However, the chemical shifts calculated for the 1.06 and 1.42 isomers almost coincide (calc. −7.2 and −7.6 ppm; exp. −6.7 ppm^16*b*^) and explain the lack of significant *δ*(H_2_)–*T* dependence. At the same time, the calculated *J*_HD_ values reveal a considerable difference (18.8 and 7.2 Hz; values from correlations are 23 and 9.2 Hz).

Explicit solvation attempts with CH_2_Cl_2_ did not reveal well-defined non-covalent interactions between RuH_2_ and CH_2_Cl_2_ with the level of theory used (both Ru–H⋯Cl–CH_2_Cl and Ru–H⋯H–CHCl_2_ interactions were considered, see ESI 11†). It was found that instead, CH_2_Cl_2_ could be bound to [Cp*Ru(dppm)(H_2_)]^+^ through an interaction with the π-electron cloud of one of the Ph rings, and a hydrogen bond with the <svg xmlns="http://www.w3.org/2000/svg" version="1.0" width="10.400000pt" height="16.000000pt" viewBox="0 0 10.400000 16.000000" preserveAspectRatio="xMidYMid meet"><metadata> Created by potrace 1.16, written by Peter Selinger 2001-2019 </metadata><g transform="translate(1.000000,15.000000) scale(0.011667,-0.011667)" fill="currentColor" stroke="none"><path d="M80 1160 l0 -40 40 0 40 0 0 -40 0 -40 40 0 40 0 0 -40 0 -40 40 0 40 0 0 -40 0 -40 40 0 40 0 0 -40 0 -40 40 0 40 0 0 -40 0 -40 40 0 40 0 0 -40 0 -40 40 0 40 0 0 80 0 80 -40 0 -40 0 0 40 0 40 -40 0 -40 0 0 40 0 40 -40 0 -40 0 0 40 0 40 -40 0 -40 0 0 40 0 40 -40 0 -40 0 0 40 0 40 -80 0 -80 0 0 -40z M560 520 l0 -40 -40 0 -40 0 0 -40 0 -40 -40 0 -40 0 0 -40 0 -40 -40 0 -40 0 0 -40 0 -40 -40 0 -40 0 0 -40 0 -40 -40 0 -40 0 0 -40 0 -40 -40 0 -40 0 0 -40 0 -40 80 0 80 0 0 40 0 40 40 0 40 0 0 40 0 40 40 0 40 0 0 40 0 40 40 0 40 0 0 40 0 40 40 0 40 0 0 40 0 40 40 0 40 0 0 80 0 80 -40 0 -40 0 0 -40z"/></g></svg> P–CH_2_–P <svg xmlns="http://www.w3.org/2000/svg" version="1.0" width="10.400000pt" height="16.000000pt" viewBox="0 0 10.400000 16.000000" preserveAspectRatio="xMidYMid meet"><metadata> Created by potrace 1.16, written by Peter Selinger 2001-2019 </metadata><g transform="translate(1.000000,15.000000) scale(0.011667,-0.011667)" fill="currentColor" stroke="none"><path d="M480 1160 l0 -40 -40 0 -40 0 0 -40 0 -40 -40 0 -40 0 0 -40 0 -40 -40 0 -40 0 0 -40 0 -40 -40 0 -40 0 0 -40 0 -40 -40 0 -40 0 0 -80 0 -80 40 0 40 0 0 40 0 40 40 0 40 0 0 40 0 40 40 0 40 0 0 40 0 40 40 0 40 0 0 40 0 40 40 0 40 0 0 40 0 40 40 0 40 0 0 40 0 40 40 0 40 0 0 40 0 40 -80 0 -80 0 0 -40z M80 480 l0 -80 40 0 40 0 0 -40 0 -40 40 0 40 0 0 -40 0 -40 40 0 40 0 0 -40 0 -40 40 0 40 0 0 -40 0 -40 40 0 40 0 0 -40 0 -40 80 0 80 0 0 40 0 40 -40 0 -40 0 0 40 0 40 -40 0 -40 0 0 40 0 40 -40 0 -40 0 0 40 0 40 -40 0 -40 0 0 40 0 40 -40 0 -40 0 0 40 0 40 -40 0 -40 0 0 40 0 40 -40 0 -40 0 0 -80z"/></g></svg> fragment. Such binding has a little effect on equilibria between the isomers. Ion pairing effects also seem to be of secondary importance since a non-nucleophilic counter-anion B(Ar^F^)_4_^−^ was used for solution measurements.^16*b*^ The neutron diffraction structure^10^ was obtained with a more nucleophilic BF_4_^−^; however, unlike many other cases (see below), the latter did not reveal close contacts with RuH_2_. Instead, interaction between BF_4_^−^ and P–CH_2_–P fragment could be found in the solid state, and seemingly this interaction is preferred in solution as well, as shown by calculations (ESI 11†). We thus suppose that our model in the first approximation correctly reflects the chemistry of [Cp*Ru(dppm)(H_2_)]^+^ in solution.

### Model with isomers and non-classical hydrides

Looking at a broader picture, a comparatively flat PES, which is a feature typical of hydrides belonging to the non-classical region (*r*(H–H) of *ca.* 1.0–1.6 Å), makes such hydrides very sensitive to specific and non-specific solvation, as well as to other interactions. As a result, even weak interactions of 1–5 kcal mol^−1^, which are perhaps numerous for highly polarizable M–H units, can trigger significant changes in *r*(H–H).

**Fig. 11:**
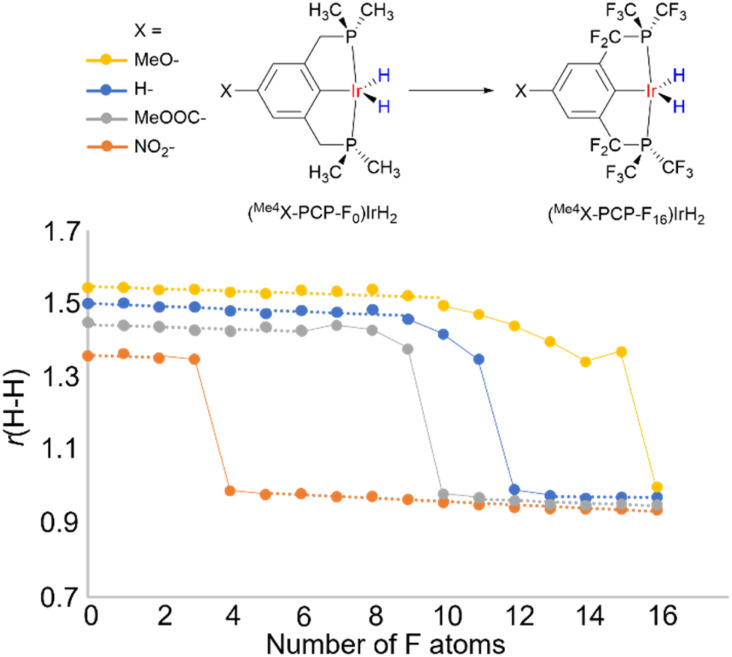
H–H distance in (^Me4^X-PCP-F_*n*_)IrH_2_ as a function of the number of F atoms in the ligand, probing the “continuum” of H–H activation. Dashed line represents classical regions where *r*(H–H) has linear low-slope dependence on the number of F atoms. Solid line represents S-shaped non-classical regions with *r*(H–H) being very sensitive to minor changes.

Another way to present it is as follows. It is believed that there is a H–H bond activation continuum in transition metal hydrides (Fig. 1).^2,54^ This view is based on the existence of hydride complexes that cover the whole possible range of *r*(H–H) (*ca.* 0.8–3.2 A). However, little is known what this continuum may look like. To address this, we computationally varied the electronic properties of the model compounds (^Me4^X–PCP)IrH_2_ in an incremental way by substituting the ligand H atoms with F, thus plotting *r*(H–H) *vs.* “electron-richness” of the ligands (Fig. 11). Instead of a straight line, the plot revealed three regions (see also ESI 12†). Thus, regions of “classical” dihydride and dihydrogen complexes were observed, where *r*(H–H) exhibited a small-slope linear dependence on the number of F atoms. These regions were connected by an S-shaped “non-classical” region, where small changes in electron properties were accompanied by big changes in *r*(H–H). Remarkably, the increment of one F atom (corresponding to a 6 cm^−1^*ν*(CO) change using the popular organometallic metrics) was big enough for a *ca.* 0.3 Å leap, bypassing the 1.3–1.0 Å region. Dihydrides revealed Mayer bond orders of 0.7–0.9 for Ir–H and 0.1–0.2 for H–H, while dihydrogen complexes of 0.4–0.5 for Ir–H and 0.4–0.5 for H–H. A conceptually similar pattern was observed for another model system, [Os(H_2_)(en)_2_X]^+^ (see ESI 12†).

Hence, the pools of more rigid, resilient classical M(*n*)MH_2_ and M(*n*+ 2)M(H_2_) structures are connected *via* the pool of more soft, fragile non-classical structures where *r*(H–H) distances are in fact in the region of transition states between dihydrogen complexes and dihydrides. If the pattern in Fig. 11 can be generalized beyond the compounds studied, then one would expect that (a) for the non-classical structures, external stimuli would likely produce considerable changes in *r*(H–H), (b) some of the apparent non-classical *r*(H–H) may be a weighted-average of isomeric structures and (c) the existence of such isomers enabled/enhanced by external stimuli is the primary reason for temperature-dependent *J*_HD_, *δ*(MH_2_) and other properties.

Literature data support the high sensitivity of “non-classical” structures to external stimuli/medium. Thus, for the “center of gravity” of the non-classical region, three neutron diffraction structures are reported: IrH(H_2_)Cl_2_(P^i^Pr_3_)_2_), 1.11 Å,^55^ [Os(H_2_)Cl(dppe)]^+^PF_6_^−^ 1.15 Å (ref. 16g) and Cp*OsH(H_2_)H(PCy_3_)^+^BF_4_^−^ 1.31 Å (ref. 56) (raw *r*(H–H) values are given). All three reveal close contacts involving MH_2_ units. In the first example the contact is an Ir–H⋯Cl–Ir hydrogen bond. The other two reveal contacts with counter-anions (Os–H⋯F–(BF_3_/PF_5_). Seemingly, *r*(H–H) distances in the solid state and in solution are different as a result of these interactions (Table S15†). This was supported by DFT calculations, where different *r*(H–H) distances were observed with and without contacts included.^22,55–57^

The effects of vibrational averaging were always a competing explanation for a mismatch between neutron diffraction and DFT results; now it is made less likely. It is very reasonable to propose that in solution both free and bound complexes are present, in a manner similar to the one established for (X–POCOP)IrH_2_. Therefore, one could expect the NMR parameters to be temperature dependent. Indeed, temperature-dependent *J*_HD_ was reported for [Os(H_2_)Cl(dppe)]^+^PF_6_^−^; perhaps, it would have been observed for the other compounds as well, should the observation window be suitable to allow it.^58^

In the known cases, ion pairing interactions shorten *r*(H–H) compared to the non-bound forms (see for example Table S15†). A remarkable example to further illustrate this is the complex [(PP_3_)Co(H_2_)]^+^, which in the solid state exists as a dihydrogen complex with a PF_6_^−^ counter-anion, and as a dihydride complex with a less nucleophilic BPh_4_^−^ counter-anion.^59^ In the dihydrogen form, PF_6_^−^ is clearly located in a position to interact with hydrides, while in the dihydride form BPh_4_^−^ does not seem to have any interactions with the CoH_2_ unit. In solution, solvents can compete with counter-anions for binding with MH_2_. Thus, the complex [Mo(CO)Cp*(H_2_)(PMe_3_)_2_]^+^BF_4_^−^ was reported to exist in a dihydrogen form in THF and in a dihydride form in CH_2_Cl_2_.^60^ This was rationalised through BF_4_^−^⋯H-CHCl_2_ interactions that make BF_4_^−^ less nucleophilic in CH_2_Cl_2_ compared to THF.^60^ As a result, the solvated ion pair in CH_2_Cl_2_ is less tight, *r*(H–H)-shortening MoH_2_⋯BF_4_ interaction is weaker and the dihydride form is favoured. The presence of solvated ion pairs at low temperatures might also better explain the spectra of [Os(H_2_)Cl(dppe)]^+^PF_6_^−^.^61,62^

The examination of X-ray diffraction structures in the 1.15–1.25 Å range revealed that almost all DFT-based distances are much shorter or much longer compared to the XRD ones (see ESI 13†). This primarily reflects the inability of X-ray diffraction to accurately locate hydrides, but in some cases could be a result of medium/packing effects. An interesting example is the complex [Os(C_6_H_4_pyOPh)(η^2^-H_2_)(P^i^Pr_3_)_2_]^+^BF_4_^−^, which demonstrates a solid state, solution and DFT-calculated *r*(H–H) close to 1.2 Å.^63^ This complex exhibits contacts between OsH_2_ and BF_4_^−^ moieties, and a reveals temperature dependence of *δ*(OsH_2_) in solution (ESI of ref. 63), which possibly could be associated with such ion pairing in solution. At the same time, two closely related analogues with *r*(H–H) 1.38 and 1.08 Å (calc.) revealed negligible temperature dependence of *δ*(OsH_2_). This highlights that it is the center of the non-classical region that is most sensitive to external stimuli, although other hydrides of course could be affected as well.

## Conclusions

Overall, we have demonstrated that *J*_HD_ changes in (X–POCOP)IrH_2_ can be rationalized through the rapid equilibrium between S and NS isomers. Both of them were deduced from solution-state spectra and unambiguously characterized by neutron diffraction in the solid state. The drastic geometric difference between the isomers (for example, 1.4 A for S *vs.* 2.2 A for NS-bound) is responsible for the very high sensitivity of (X–POCOP)IrH_2_ to the solvent environment. In particular, isotope effects on chemical shifts proved a useful tool to probe the NS structure, due to the very high difference in chemical shifts between the hydrides in NS. Thus, we established the first example of a complex with temperature-dependent *J*_HD_, where two isomers were successfully isolated and characterized, and a good quality fit of experimental data was obtained. A VT neutron diffraction study indicated only a minor *r*(H–H) change that could be associated with vibrational corrections.

We also highlighted the role of specific solvation and non-covalent interactions for metal hydrides. The complexation of (X–POCOP)IrH_2_ with C_6_F_5_I and CH_2_Cl_2_ was characterized, including the first neutron diffraction structure of a halogen bond involving hydrides. Although the binding energies are comparatively small (1–5kcal mol^−1^), they are responsible for the stabilization of the NS isomer through the formation of NS-bound, which affects the span of *δ*(IrH_2_) and *J*_HD_ in the presence of C_6_F_5_I and CH_2_Cl_2_, when compared to less interacting solvents such as toluene.

Computational methods supported the S/NS/NS-bound model chemistry. In the light of importance of solvation, we suppose that DFT calculations with the CPCM method provided overall satisfactory performance for non-specific solvation in the case of neutral complexes, even though the CPCM method minorly overestimated the effect of relative permittivity (and thus it may not in a perfectly precise way capture some of the fine features such as the geometry of NS and S–NS energy gaps). We note that the CPCM model is likely growing progressively less accurate upon charge buildup on the compounds studied,^64^ so hydrides bearing multiple charges may be especially challenging.

As for specific solvation, such as noncovalent interactions with C_6_F_5_I and CH_2_Cl_2_, we found it essential to use DLPNO-CCSD(T) corrections to DFT energies, in order to obtain good agreement with the experimental data. Worth noting is that in addition to the thermodynamic data, the NMR parameters of some key structures were calculated with highly accurate methods and revealed good agreement with experiment.

Having successfully established the model with isomers for (X–POCOP)IrH_2_ type hydrides with temperature-dependent *J*_HD_, we hypothesized that the most pronounced temperature dependence of *J*_HD_ in other compressed dihydrides and elongated dihydrogen complexes is also explained by equilibria between two or more isomeric entities, which can be additionally discriminated by non-covalent interactions with solvent. In support of that hypothesis, a re-examination of the archetypical complex [Cp*Ru(dppm)(H_2_)]^+^ found two minima on the PES with reasonable *r*(H–H) that allowed good fit of the experimental data, especially given the limitations of the model for cationic complexes. Remarkably, unlike the calculated *J*_HD_, the calculated chemical shifts for the two isomers almost coincide.

Looking at a broader picture, our attempt to access the “continuum” of H–H bond activation through incrementally decreasing the electron-deficiency of the pincer ligand (Fig. 11) revealed that at least for some compounds, there are regions of “rigid” dihydrides and dihydrogen complexes, which are connected with an S-shaped region of “sensitive” non-classical hydride complexes. In this “sensitive” region, small external stimuli related to son-specific solvation, non-covalent interactions, packing effects, *etc.*, would more likely produce noticeable changes in *r*(H–H), and as a consequence, in spectral parameters. The existence of isomers seems likely under these conditions. Since previous attempts to guess the nature of isomers were often not precise, it worth listing why the isomers could be formed. This can occur due to: differential stabilization of two dihydride complexes by non-specific solvation due to *e.g.* different dipole moments (S and NS isomers), differential stabilization of the dihydrogen complex and a dihydride complex by non-specific solvation (dihydrogen and dihydride forms of [Cp*Ru(dppm)(H_2_)]^+^), specific solvation/non-covalent interaction with solvent (formation of NS-bound*via* a dihydrogen bond with CH_2_Cl_2_ and a halogen bond with IC_6_F_5_), *etc.* To this one can add ion pairing, since several “non-classical” hydrides are known, which reveal different distances in the solid state, where contacts of between hydrides and a counter-anion could be found, and in solution (see above for discussion). Ion pairing must be to some extent present in solution as well, and such compounds might reveal temperature dependent spectra, should the combination of interaction strength and wideness of the observation window allow it. To conclude this part, it seems that “non-classical” hydrides that span approximately from 1.0 to 1.6 Å are especially sensitive to external stimuli. Due to various interactions with their environment and medium, such hydrides are likely to exhibit isomeric species and thus reveal temperature-dependent properties, including *J*_HD_.

## Data availability

The datasets supporting this article have been uploaded as part of the ESI.†

## Author contributions

AP designed the project, performed experiments and calculations and wrote the manuscript. SC carried out neutron diffraction data acquisition and analysis. OW provided assistance with administrative matters at early stages of the project. All authors discussed and participated in the finalization of the manuscript.

## Conflicts of interest

There are no conflicts to declare.

## Supplementary Material

SC-014-D3SC04197B-s001

SC-014-D3SC04197B-s002
