# Supplementary material for: Unravelling strong temperature-dependence of JHD in transition metal hydrides: solvation and non-covalent interactions versus temperature-elastic H–H bonds
Source: Chem Sci. 2023 Oct 13;14(43):12308–20. doi: 10.1039/d3sc04197b (PMC10631239; doi:10.1039/d3sc04197b)
Supplement: SC-014-D3SC04197B-s001 [file SC-014-D3SC04197B-s001.pdf]

Electronic Supplementary Material (ESI) for Chemical Science.
This journal is © The Royal Society of Chemistry 2023

Supporting Information

Unravelling Strong Temperature-Dependence of J_{HD} in Transition Metal Hydrides: Solvation and Non-Covalent Interactions with Solvent versus Temperature-Elastic H-H Bonds

Table of Contents

1. Experimental part……………………………………………………………………S3

1.1 General considerations and procedures……………………………………………...S3

1.2 X-ray crystallography…………………………………………………………......S4

1.3 Neutron diffraction study……………………………………………………………S6

1.4 Analysis of thermal motion in (*p*-H-POCOP)IrH_2…………………………………..S8

2. Additional NMR data…………………………………………………………………S11

Figure S2. Variable temperature ^{1}H NMR spectra of (X-POCOP)IrH_2 and (PCP)IrH_2 in toluene-d_8, the hydride region is shown……………………………………………..S11

Figure S3. Variable temperature ^{1}H NMR spectra of (p-MeOOC-POCOP)IrH_2 in toluene-d_8 and CD_2Cl_2……………………………………………………………S12

Table S4. Selected NMR data for (X-POCOP)IrH_2 and (PCP)IrH_2…………………...S13

3. Fitting of ^{1}H NMR chemical shifts and J_{HD} coupling constants…………………...……S14

3.1 Accuracy of coupling constant measurement…………………………………....S14

3.2 Accuracy of fitting……………………………………………………………………S16

3.3 Fitting procedures……………………………………………………………………..S17

3.4 Model chemistry………………………………………………………………………S17

3.5 Complex (*m*-bis-CF_3-POCOP)IrH_2 in toluene-d_8………………………………..S18

3.6 Complex (*p*-MeOOC-POCOP)IrH_2 in toluene-d_8………………………………S20

3.7 Complexes (*p*-H-POCOP)IrH_2 and (*p*-MeO-POCOP)IrH_2 in toluene-d_8: notes……S21

3.8 Complex (*p*-H-POCOP)IrH_2 in toluene-d_8………………………………………S22

3.9 Complex (*p*-MeO-POCOP)IrH_2 in toluene-d_8……………………………………S23

Table S6. A summary of two-component (**S** and **NS**) model fit of NMR spectra of (X-POCOP)IrH_2 in toluene-d_8…………………………………………………………...S25

Table S7. Experimental and calculated distances and energies relevant to **S** and **NS** structures in (X-POCOP)IrH_2 complexes……………………………………………S26

4. Evaluating isotope effects in (X-POCOP)IrH_2…………………………………………S27

Table S8. Experimental and calculated isotope effects on chemicals shifts in (X-POCOP)IrH_2……S30
5. IR spectroscopy of (X-POCOP)IrH_2……S32
6. Halogen bond strength estimation……S34
6.1 General notes……S34
6.2 Fitting of δ(IrH_2)-*T* dependence……S34
6.3 Fitting to 1:1 binding isotherm……S36
6.4 Analysis of (*p*-MeOOC-POCOP)IrH_2---C_4F_9I ↔ (*p*-MeOOC-POCOP)IrH(C_4F_9I)H equilibrium……S37
7. Dihydrogen bonding with CH_2Cl_2……S38
8. A control experiment evaluating the effect of relative permittivity on (*p*-H-POCOP)IrH_2……S44
9. Computational details……S45
9.1 General considerations……S45
9.2 Additional computational data……S47
9.3 Explicit solvation attempts……S56
9.4 Examining the possible multireference nature of (X-POCOP)IrH_2 and (PCP)IrH_2…S58
10. Calculation of NMR chemical shifts and coupling constants……S60
11. Re-evaluation of complex Cp*Ru(dppm)H_2^+……S62
11.1 Re-evaluation of complex Cp*Ru(dppm)H_2^+……S62
11.2 Explicit solvation by one CH_2Cl_2 molecule……S64
11.3 Possible ion pairing effects……S66
Table S15. The effect of solid-state interactions on some transition metal hydrides……S68
12. Probing the "continuum" of H-H bond activation by transition metals……S64
13. Examination of transition metal hydrides with XRD-based *r*(H-H) 1.15-1.25 Å……S69
14. References……S73
15. List of Cartesian coordinates……S77

1. Experimental part

1.1. General considerations. All manipulations were carried out under an inert gas atmosphere using standard Schlenk, high vacuum line and glovebox techniques unless otherwise stated. Toluene-d_8, methylcyclohexane-d_{12} and THF-d_8 were distilled under vacuum from Na/benzophenone. CD_2Cl_2, C_6F_5I and C_4F_9I were distilled from CaH_2. NMR spectra were recorded on Bruker Avance 400 MHz, Bruker Avance 500 MHz and Varian Unity INOVA 500 MHz instrument. ^1H and ^{13}C NMR chemical shifts are reported in parts per million and referenced to the residual signals of deuterated solvents (toluene-d_8: 7.09, 137.48; CD_2Cl_2: 5.32, 53.84; THF-d_8: 3.58, 67.21). ^{31}P NMR chemical shifts are reported relative to external 85% solution of phosphoric acid. Calibration curve obtained using neat methanol sample was used for variable temperature measurements. Spin-lattice relaxation times (T_1) were measured using a standard inversion recovery pulse sequence. All $T_1(min)$ values are provided at 500 MHz. IR spectra were recorded on a Bruker Alpha spectrometer. Complexes (*p*-H-POCOP)IrH_2, (*p*-MeO-POCOP)IrH_2, (*p*-MeOOC-POCOP)IrH_2, (*m*-bis-CF_3-POCOP)IrH_2, and (PCP)IrH_2, were prepared according to slightly modified literature procedure[4] as described below; the NMR spectra matched the literature data.[1-4]

Synthesis of dihydride complexes

In a nitrogen atmosphere glovebox, 0.080 mmol of the respective pincer hydrido-chloride complexes and 0.080 mmol (1/1) of ^{t}BuOK were placed in a thick-walled flask. The flask was evacuated and benzene (15 ml) was vacuum-transferred, followed by refilling with H_2 at -196 °C. The reaction mixture was left to stir at 25…40 °C until the reaction was complete, as was indicated by a color change from red-orange to colorless or very pale yellow (typically 24-48 h). The flask was refilled with Ar (caution when opening: ca. 4 atm pressure!), degassed water (10 ml) was added and the mixture was intensely stirred for 10 min. The organic layer was filtered through a thin pad of Celite via cannula, and the water layer was washed with 5 ml of benzene that was also passed through the same pad of Celite. The organic fractions were combined and the volatiles were evaporated in vacuum to provide a mixture of the respective dihydride and tetrahydride complexes. After proper drying in high vacuum, the respective dihydride complexes were obtained in almost quantitative yields as red or red-orange solids. For (*p*-MeOOC-POCOP)IrH_2 and (*m*-bis-CF_3-POCOP)IrH_2, and especially for (PCP)IrH_2, drying have to be performed at elevated temperatures in order to achieve IrH_4 to IrH_2 conversion in a reasonable time.

Selected "fingerprint" NMR signals of the dihydride complexes obtained at uniform conditions (toluene-d_8, 25 °C) are given below:

Complex	X = *p*-MeO-	X = *p*-H-	X = *p*-MeOOC-	X_2 = *m*-bis-CF_3-	(PCP)IrH_2
δ(^1H), Ir-H, ppm	-17.57 ($^2J_{PH}$ = ca. 8.1 Hz)	-16.99 ($^2J_{PH}$ = 8.2 Hz)	-16.16 ($^2J_{PH}$ = 8.5 Hz)	-16.16 ($^2J_{PH}$ = 8.9 Hz)	-19.16 ($^2J_{PH}$ = 8.8 Hz)
δ (^{31}P), ppm	206.6	204.0	203.9	213.4	85.8

Synthesis of partially deuterated (X-POCOP)IrH$_2$ and (PCP)IrH$_2$

In a typical experiment, a solution of the respective dihydride in C_6D_6 in a J. Young NMR tube was freeze-pump-thaw degassed and refilled with D_2 gas. After vigorous shaking, the solution was allowed to react, and once the desired degree of deuteration was achieved, typically within minutes, the solution was evaporated and dried under dynamic high vacuum to convert tetrahydride form into dihydride form. A mixture of IrH$_2$, IrHD and IrD$_2$ isotopomers was thus obtained. The mixture was used immediately after preparation due to deuterium scrambling into *tert*-butyl groups.

VT NMR analysis of (X-POCOP)IrH$_2$ and (PCP)IrH$_2$

In a typical experiment, in a nitrogen atmosphere glovebox, 0.024 mmol of the respective dihydride was placed in a J. Young NMR tube, and 0.5 ml of deuterated solvent was added via syringe. The NMR tube was freeze-pump-thaw degassed, refilled with Ar and shaken for 1 min to dissolve and mix the chemicals. After that, the tube was quickly inserted into the NMR spectrometer and was followed by ^{1}H and ^{31}P NMR.

VT NMR analysis of (X-POCOP)IrH$_2$ and (PCP)IrH$_2$ in the presence of halogen bond acceptors

In a typical experiment, in the nitrogen atmosphere glovebox, 0.024 mmol of the respective dihydride was placed in a J. Young NMR tube, and 0.5 ml of toluene-d_8 was added. The NMR tube was degassed, and refilled with Ar. This was followed by addition of the specified amount of C_6F_5I or C_4F_9I via a septum cap and quickly brough to a spectrometer pre-cooled to a desired temperature. All dihydrides studied react with C_6F_5I with formation of the respective IrHI complexes; the reactivity follows order *p*-MeOOC ≈ *m*-bis-CF$_3$ < *p*-H < *p*-MeO << (PCP)IrH$_2$. Dihydrides with X = *p*-MeOOC, *m*-bis-CF$_3$ and *p*-H allow rapid handling at room temperature, while X = *p*-MeO and especially (PCP)IrH$_2$ react with C_6F_5I within minutes. Therefore, for those complexes, C_6F_5I or C_4F_9I were added to a solution of dihydrides pre-cooled to -60 °C, and the cooling was maintained until the tube was inserted into a NMR spectrometer. For titration purposes, the concentration of (X-POCOP)IrH$_2$ was adjusted to account for an amount of dihydrides that was converted to IrHI during sample preparation.

1.2 X-ray crystallography

Intensity data were collected with an *Oxford Diffraction* Excalibur 3 system, using ω-scans and Mo Kα (λ = 0.71073 Å) radiation.[5] The data were extracted and integrated using Crysalis RED.[6] The structures were solved and refined by full-matrix least-squares calculations on F^2 using JANA2006.[7] Molecular graphics were generated using Mercury 3.10.3.[8] CCDC deposition numbers for (*p*-H-POCOP)IrH_2 and (*p*-MeOOC-POCOP)IrH_2-IC_6F_5 are 2257556 and 2257557, respectively.

Table S1. Summary of X-ray crystallographic data.

Compound	(*p*-H-POCOP)IrH_2	(*p*-MeOOC-POCOP)IrH_2-IC_6F_5
Chemical formula	$C_{22}H_{41}IrO_2P_2$	$C_{30}H_{43}IrO_4P_2F_6I$
Crystal system	Monoclinic	Triclinic
Space group	P 2_1/c	P -1
Temperature (K)	100	100
Cell parameters	a=16.5349(16)<br>b=10.5849(9)<br>c=15.6632(12)<br>β=115.113(8)	a = 10.6200(2)<br>b = 10.6749(2)<br>c=16.4369(3)<br>α=87.8506(15)<br>β=84.0788(15)<br>γ=68.7007(16)
Cell volume	2482.2(4)	1726.87(6)
Z	4	2
Crystal size	1 x 0.3 x 0.2 mm	0.85 x 0.60 x 0.20 mm
Radiation type	X-ray	
Wavelength	0.71073	
No of observed reflections	22737	37775
No parameters	244	388
R(sigma)	0.045	0.0376
R1, wR2, Goof	0.0288, 0.0674, 1.320	0.0310, 0.0669, 1.420
R1 after Fourier merging, No of unique reflections	3.91, 5891	4.23, 8337
$\Delta\rho_{max}$, $\Delta\rho_{min}$	1.80<br>-1.15	1.21<br>-0.85

1.3 Neutron diffraction studies

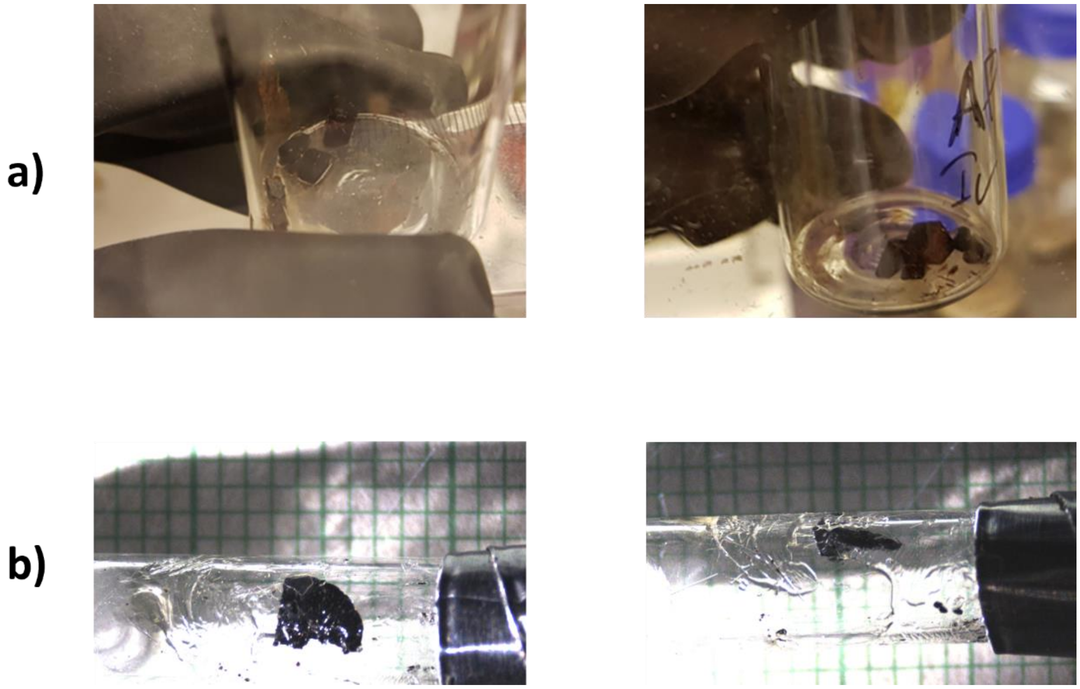

Figure S1. Images of the crystals grown and used for neutron diffraction. a) (*p*-H-POCOP)IrH_2; b) (*p*-MeOOC-POCOP)IrH_2---IC_6F_5.

Neutron diffraction data, for both (*p*-H-POCOP)IrH_2 and the (*p*-MeOOC-POCOP)IrH_2-IC_6F_5 adduct, were collected on the SXD instrument[9] at the ISIS Neutron and Muon Facility in Harwell (UK), using the time-of-flight (TOF) Laue diffraction method. A single crystal of (*p*-H-POCOP)IrH_2 of about 5 x 3 x 1 mm size was attached with aluminium tape on the tip of an aluminium pin and mounted on the central stick of a top-loading helium cryo-cooled refrigerating device (CCR). The sample chamber was first evacuated to 2 mbar and then filled with 200 mbar of He gas to ensure thermal conductivity. Bragg intensities were collected at 10, 100 and 295 K in a series of 5 angular orientations of the crystal around the vertical axis of the instrument with exposure times respectively of 5 h 50 min, 7 h and 11 h 30 min per orientation.

For the (*p*-MeOOC-POCOP)IrH_2---IC_6F_5 adduct, a single crystal of about 3 x 2 x 0.5 mm size was sealed in a quartz capillary under nitrogen atmosphere before being mounted on the sample stage of a bottom loading CCR. The sample chamber was evacuated and kept at 10^{-5} bar for the duration of the experiment. Diffraction data were collected at 40 K in a series of 6 orientations with exposure time of ~14 h each. Cell dimensions for all structures and Bragg intensities were extracted

using the line-integral procedure implemented in the SXD2001 software.[10] Data were corrected for Lorentz effect but although the absorption neutron cross section for Iridium is quite big (425 barns at 1.78 Å) no absorption correction was applied. The starting model for the structural refinement was based on the atomic coordinates of the non-hydrogen atoms from the available X-ray structure, while all hydrogen atoms were located from neutron Fourier difference maps. All structures were refined by full matrix least squares on F^2 using the SHELXL software.[11] In the (*p*-H-POCOP)IrH_2 structures, anisotropic displacement parameters (ADPs) were used for all atoms except for the hydrogens of the *tert*-buthyl groups, which were refined using an isotropic group factor. Rotational disorder the –CH_3 groups of the *tert*-butyl units was observed for all structures but the limited amount of data available at those temperatures did not allow to refine a disordered model. Also due to the limited amount of data obtained for the (*p*-MeOOC-POCOP)IrH_2---IC_6F_5 adduct, the structure was refined with isotropic temperature factors for all atoms except for the hydride hydrogens. Experimental details for all structures are given in Table S2. All crystallographic data have been deposited with the joint Cambridge Crystallographic Data Centre and Fachinformationszentrum Karlsruhe database service. The data are provided free of charge to anybody who require them via www.ccdc.cam.ac.uk/structures. Reference numbers 2262956, 2262957, 2262958 and 2262955 refer respectively to the 10, 100, 295 K neutron data of (*p*-H-POCOP)IrH_2 and the 40 K neutron data of the (*p*-MeOOC-POCOP)IrH_2---IC_6F_5 adduct.

Table S2. Summary of neutron diffraction data.

Compound	(*p*-H-POCOP)IrH_2			(*p*-MeOOC-POCOP)IrH_2---IC_6F_5
Chemical formula	$C_{22}H_{41}IrO_2P_2$			$C_{30}H_{43}IrO_4P_2F_6I$
Crystal system	Monoclinic			Triclinic
Space group	P 2_1/c			P -1
Temperature (K)	10	100	295	40
Cell parameters	a=16.656(9)<br>b=10.538(5)<br>c=15.607(8)<br>β=114.75(2)	a=16.639(9)<br>b=10.593(5)<br>c=15.698(8)<br>β=114.88(2)	a=16.607(9)<br>b=10.791(5)<br>c=15.985(8)<br>β=115.99(2)	a=10.644(9)<br>b=10.798(5)<br>c=16.580(8)<br>α=88.31(2)<br>β=84.52(2)<br>γ=68.64(2)
Cell volume	2487.7(1)	2510.2(1)	2594.7(1)	1766.5(1)

Z	4			2
Crystal size	5 x 3 x 1 mm			3 x 2 x 0.5 mm
Radiation type	Neutron			
Wavelength	Polychromatic, 0.35 – 6.5 Å			
No of observed reflections [[I>1σ(I)]	12934	8220	4122	3008
No parameters	438	439		218
R(sigma)	0.1071	0.0933	0.0614	0.1397
R1, wR2, Goof	0.0962, 0.2382, 1.037	0.1010, 0.2501, 1.101	0.1241, 0.3070, 1.469	0.1648 0.3783, 1.603
R1 after Fourier merging, No of unique reflections	0.0907, 6689	0.0994, 4030	0.1246, 1824	0.1941, 1591
$\Delta\rho_{max}$, $\Delta\rho_{min}$	3.26, -3.33	1.99, -2.27	0.94, -1.04	2.11, -1.37
Extinction coeff.	0.0006(1)	0.0005(1)	0.0021(3)	0.0004(1)

1.4. Analysis of thermal motion in (*p*-H-POCOP)IrH$_2$

The large ADP values refined for the hydride hydrogens in the (*p*-H-POCOP)IrH$_2$ structure at all temperatures (see Figure 2 in the manuscript) and the huge standard deviations on the Ir-H distance at 295 K (see Table S3) are a strong an indication of a large internal vibrations of these hydrogens. In order to get a better insight of the dynamics of the molecule in the crystal, we have performed a normal coordinate analysis of the ADP's according to the Bürgi and Capelli method,[12] as implemented in the program NKA.[13]

A unique set of normal modes was used to describe the ADP's at all temperatures. The molecular coordinate system used in the analysis had the x-axis bisecting the plane of the benzene ring in the C4-C1 direction, the y-axis along the P1-P2 direction and the z-axis completing a Cartesian right-handed coordinate system. The disordered –CH$_3$ groups of the *tert*-butyl units were not included in the calculations. The initial model of motion consisted of the six rigid-body degrees of freedom (three translations and three librations) for which six frequencies were refined. Four additive tensors were also refined to account to the high-frequency internal vibrations (such as bending and

stretching): one tensor to account for all atoms in the ligand molecular plane (C1 to C6, P1, P2, O1, O2 and Ir1), one for the linking C atoms in the *tert*-butyl group (C7, C11, C15, C19), one for the hydrogens attached to the benzene ring and one for the two hydride hydrogens. Although the molecule sits in the crystal in general position, it shows C_{2v} point symmetry and therefore the following restraints were used for the coupling of the different components: L_y and T_z were allowed to mix, as well as L_z and T_y, while L_x and T_x were refined as single-component vectors. A total of 66 parameter with 35 restraints were refined from 360 observations for the rigid-body model, resulting in an agreement factor of 27% and a goodness of fit of 1.43. The frequency for each normal mode refined to the following values: L_x = 65(8), 97%L_y+3%T_z = 52(3), L_z = 60(8), T_x = 26(1), T_y = 25(1), 97%T_z+3%L_y = 21(1) cm^{-1}. The tensorial component ε_{33} accounting for high frequency motions of the hydride hydrogens in the direction perpendicular to the Ir-H bond and out of the molecular plane of the ligand refined to 0.0617(33) $Å^2$, a value almost three times as big as what can be computed from spectroscopy for the internal motions of these hydrogens (see Table 1 in ref. 14). In order to introduce more flexibility in the model of motion, an additional in-phase libration of the hydride hydrogens about an axis passing through the Ir atoms and parallel to the P1-P2 direction was introduced. Due to its symmetry, this mode is allowed to couple with L_y and T_z. No additional frequency has been refined but only two additional vector components were added to the model of motion. The analysis showed that the H_2 libration contributes about 7% of the L_y frequency (51(3) cm^{-1}), bringing the ε_{33} for the hydride hydrogens to a more reasonable value of 0.0382(63) $Å^2$ (final agreement 26%, Goof 1.38). Bond distances corrected for the H_2 librations are given in Table S3, together with the amplitude of the librational motion. The atomic positions corrected for the effect of libration, allowed to determine an increase of the *r*(H-H) with temperature of about 0.05 Å in the 10-295 K range.

Table S3. Temperature dependence of the bond lengths of the hydride core from the neutron diffraction measurements (top) and after correction for libration (bottom) using the results of the Normal Coordinate Analysis of the multi-temperature Anisotropic Displacement Parameters.

	(*p*-H-POCOP)IrH_2			(*p*-MeOOC-POCOP)IrH_2---IC_6F_5
Temperature (K)	10	100	295	40
Ir – H1 (Å)	1.616 (7)<br>1.621	1.609 (10)<br>1.625	1.608 (24)<br>1.653	1.656 (39)
Ir – H2 (Å)	1.605 (9)<br>1.610	1.580 (14)<br>1.596	1.616 (35)<br>1.661	1.521 (89)
H1-H2 (Å)	1.425(14)<br>1.429	1.436(22)<br>1.448	1.438(55)<br>1.478	2.230 (64)
H1 …. I1 (Å)				2.514 (58)
H_2 libration amplitude (°)	3.84	6.63	11.4	

2. Additional NMR data

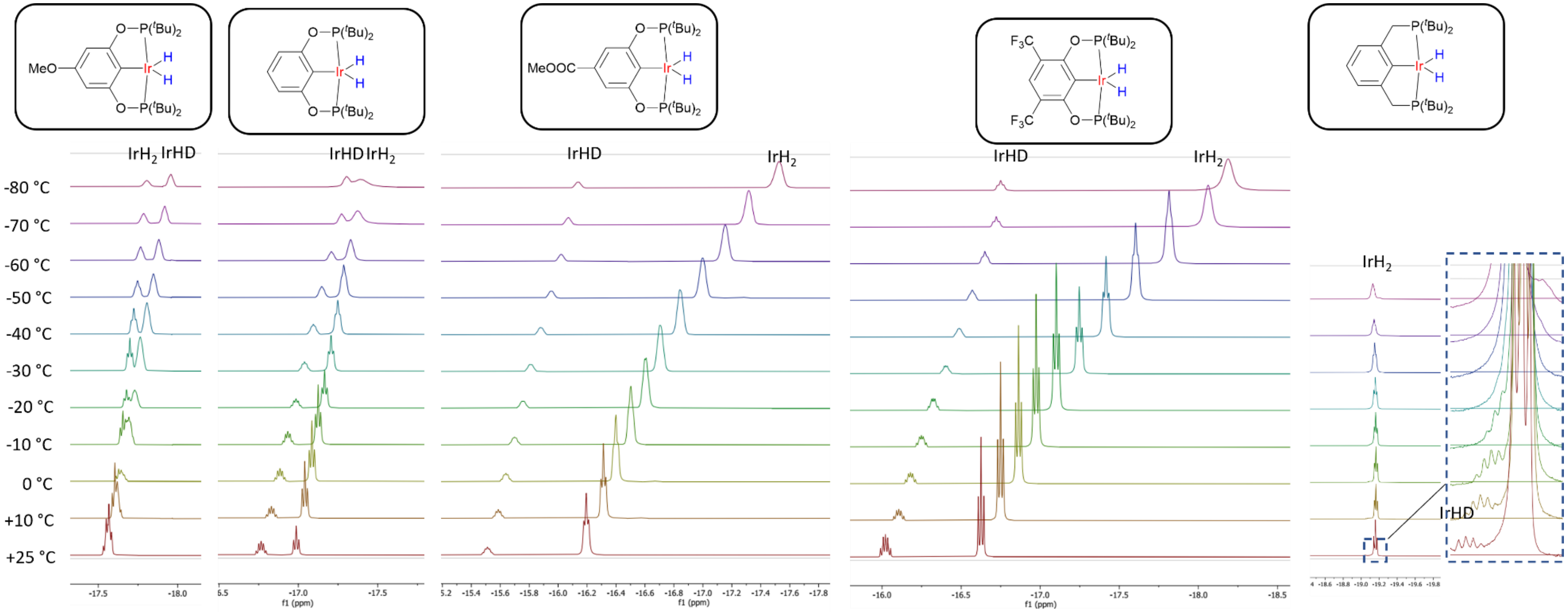

Figure S2. Variable temperature ^{1}H NMR spectra of (X-POCOP)IrH_2 and (PCP)IrH_2 in toluene-d_8, the hydride region is shown.

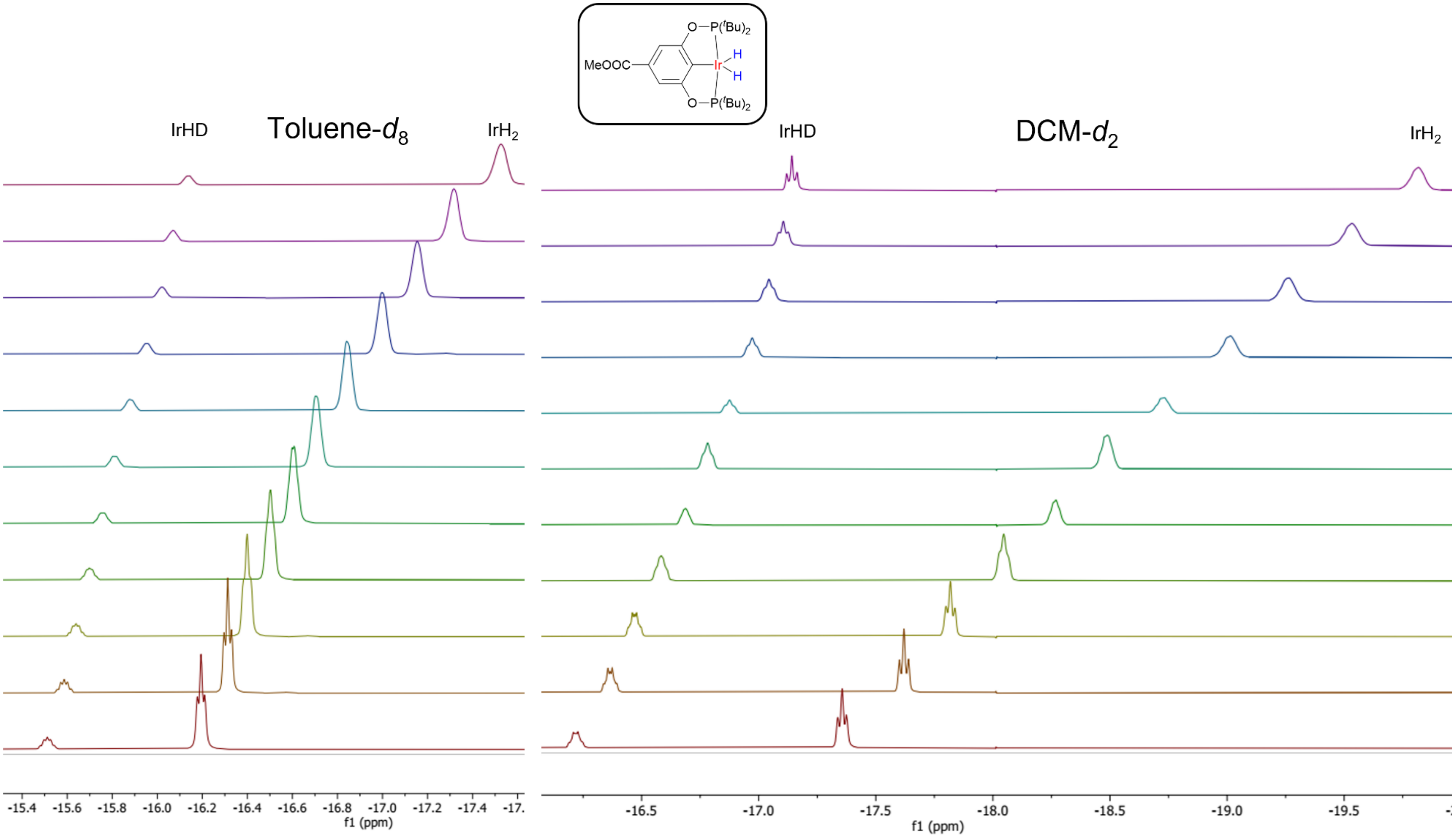

Figure S3. Variable temperature ^{1}H NMR spectra of (p-MeOOC-POCOP)IrH_2 in toluene-d_8 and CD_2Cl_2. In can be seen that in solvent with higher acceptor number (CD_2Cl_2), IrH_2 and IrHD resonances are discriminated. Due to smaller dipolar coupling with deuterium, IrHD signal linewidth is smaller and it appears as a sharp triplet, while IrH_2 gives broad singlet. Similar trend is observed for toluene-d_8, but due to smaller r(H-H), although IrHD signals has smaller linewidth compared to IrH_2, the multiplet is not resolved.

Table S4. Selected NMR data on (X-POCOP)IrH_2 and (PCP)IrH_2 in toluene-d_8, CD_2Cl_2 and THF-d_8.

	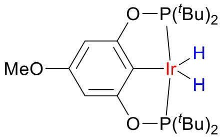				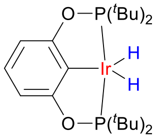				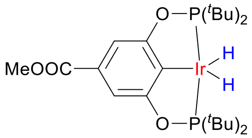				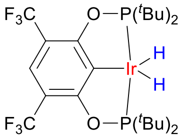				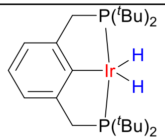	
	Toluene		CD_2Cl_2		Toluene		CD_2Cl_2		Toluene		CD_2Cl_2		Toluene		CD_2Cl_2		Toluene	
T, °C	J_{HD}	Δδ	J_{HD}	Δδ	J_{HD}	Δδ	J_{HD}	Δδ	J_{HD}	Δδ	J_{HD}	Δδ	J_{HD}	Δδ	J_{HD}	Δδ	J_{HD}	Δδ
25	5.81	-0.01	5.2	-0.12	6.95	-0.22	ca.5	-0.52	7.2	-0.68	5.1	-1.14	6.9	-0.61	n/r	-0.87	7.6	-0.12
10	5.75	0.01	-	-	6.9	-0.21	-	-	-	-0.73	4.7	-1.25	6.7	-0.64	5.2	-0.96	7.6	-0.09
0	5.67	0.02	4.9	-0.1	6.8	-0.2	ca.4.8	-0.6	6.8	-0.76	4.2	-1.35	6.3	-0.68	4.7	-1.05	7.5	-0.07
-10	5.48	0.03	4.8	-0.1	6.6	-0.19	-	-	6.5	-0.81	ca.3.3	-1.47	6.1	-0.72	4.2	-1.14	7.5	-0.04
-20	ca.5.15	0.05	n/r	-0.09	6.5	-0.17	ca.4.3	-0.69	6.1	-0.85	n/r	-1.58	5.3	-0.78	3.7	-1.3	n/r	n/r
-30	n/r	0.06	n/r	-0.09	6.3	-0.17	-	-	5.7	-0.89	n/r	-1.71	5.3	-0.85	3.1	-1.44	n/r	n/r
-40	n/r	0.09	n/r	-0.07	5.9	-0.16	n/r	-0.79	5.4	-0.96	n/r	-1.85	ca.5	-0.93	n/r	-1.62	n/r	n/r
-50	n/r	0.1	n/r	-0.07	n/r	-0.14	-	-	n/r	-1.04	n/r	-2.04	n/r	-1.03	n/r	-1.84	n/r	0.08
-60	n/r	0.11	n/r	-0.06	n/r	-0.12	n/r	-0.93	n/r	-1.13	n/r	-2.22	n/r	-1.17	n/r	-2.11	-	-
-70	n/r	0.13	n/r	-0.06	n/r	-0.1	-	-	n/r	-1.24	n/r	-2.43	n/r	-1.34	n/r	-2.45	-	-
-80	n/r	0.15	n/r	-0.06	n/r	-0.09	n/r	-1.18	n/r	-1.36	n/r	-2.68	n/r	-1.44	n/r	-2.64	-	-
$T_1(min)$	0.129		0.150		0.120		-		0.174		0.463		0.174		0.339		0.094	

	THF			THF	
T, °C	J_{HD}	Δδ		J_{HD}	Δδ
25	5.89	-0.01		6.88	-0.21
10	5.62	n/r		-	-
0	5.51	0.03		6.56	-0.19
-10	5.51	0.04		6.55	-0.18
-20	5.15	0.06		ca.5.9	-0.17
-30	5.06	0.07		-	-
-40	ca.4.8	0.09		n/r	-0.14
-50	n/r	0.11		n/r	-0.12
-60	n/r	0.12		n/r	-0.11
-70	n/r	0.14		n/r	-0.08
-80	n/r	0.16		-	-
$T_1(min)$	0.127			<0.140	

Notes: 1) n/r refers to not resolved. 2) A few J_{HD}-s with magnitude of changes ≤1 Hz are given with two digits after dot.

3. Fitting of ^{1}H NMR chemical shifts and J_{HD} coupling constants

3.1 Accuracy of coupling constant measurement

An accuracy of coupling constant measurement of ±0.2 Hz (and even better with the help of spin simulations) is reasonable for a well-resolved ^{1}H or ^{1}H{^{31}P} spectra. However, this estimate does not hold when significant broadening of lines is observed. The effect of linewidth of a signal on splitting between the lines within that signal (or on "observed" *J* in other words) appears highly relevant to the phenomena of temperature-dependent J_{HD}. A simple simulation of a triplet with J = 8 Hz indicates that when the linewidth exceeds ~50% of *J*, the observed coupling can be smaller than the real coupling by up to 1 Hz. Thus, we suppose that *J*-s, in particular J_{HD}-s that are measured in signals with broad lines are systematically underestimated. In the experimental spectra neither apodisation functions, nor line-shape analysis or empirical corrections using Table S5 data regression were found to accurately recover "real" *J*-s.

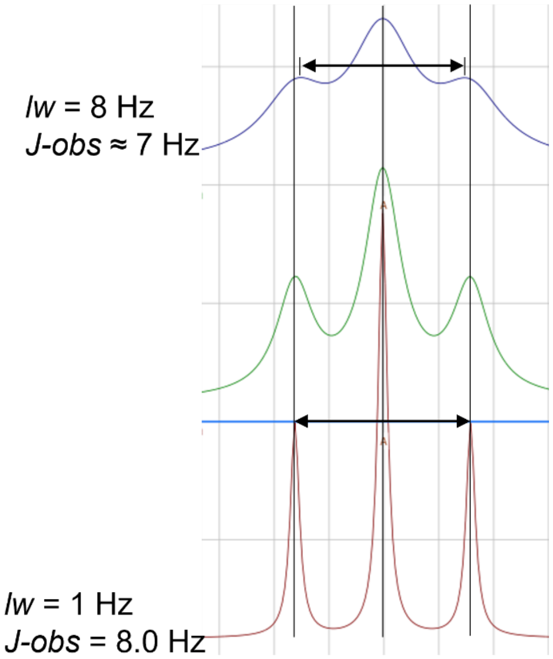

Figure S4. Simulated dependence of *J*-obs on a signal linewidth for a triplet with J = 8.0 Hz.

Table S5. Dependence of *J*-obs on a signal linewidth for a triplet with J = 8.0 Hz. MestreNova simulation, 10…-20 ppm, 64 K, 500 MHz. Peak separation measured manually.

**Linewidth, Hz**	***J*-obs, Hz**
0.5	8.0
1	8.0
2	8.0
3	8.0
4	7.97
5	7.88
6	7.79
7	7.51
8	~7

A representative practical example of the above-discussed effect is given on Figure S5. When $^2J_{PH}$ in partially deuterated (*m*-bis-CF_3-POCOP)IrH_2 is followed by ^{1}H NMR, initially the coupling constant is growing upon decreasing temperature, consistent with the **S**-**NS** equilibria being shifted towards **NS**, that has higher $^2J_{PH}$ (see the main text, Figure 5 in particular, for discussion). An unexpected decay of $^2J_{PH}$ is then observed below -20 °C. Examination of the linewidth of IrH_2 resonance indicates that at those temperatures the linewidth is comparable to the coupling, which in agreement with Table S5 data causes overlap of lines and decay of the observed splitting. While IrH_2 resonance is further broadened due to strong H---H dipole-dipole interactions, IrHD resonance is broadened to a lesser extent and clearly indicates that the "real" $^2J_{PH}$ keeps growing.

Alternative interpretations of Figure S5 such as isotope perturbation of coupling constant seem unlikely to us. Firstly, in well-resolved resonances $^2J_{PH}$ measured in IrH_2 signal matches the one measured in IrHD signal, thus ruling out a high secondary "intrinsic" isotope effect. Secondly, although certainly there is a non-statistical occupation of hydride sites by deuterium in **NS** structures, coupling constants for apical and equatorial positions are virtually the same (i.e. -8.94 and -8.95 Hz for *r*(H-H) = 2.2 Å) according to calculations. Thirdly, it is possible that deuterium favors **NS** over **S** and within the IrHD signal the relative weight of **NS** (with supposedly higher $^2J_{PH}$) is higher than in IrH_2 signal. Such deuterium distribution might affect the accuracy of measurement, but at the same time it will only make sense if there is a compound with higher $^2J_{PH}$ present, so it anyway will not change the conclusions regarding **S**-**NS** model.

The same issue is undoubtfully relevant to J_{HD} as well. In all (X-POCOP)IrH_2 complexes IrHD resonance becomes significantly more broad at low temperatures, and the raw "observed" J_{HD}-s are systematically underestimated. The J_{HD}-s used for simulations are extracted using resolution enhancement and spin simulations to improve accuracy, but this is less accurate than the direct measurement and we cannot completely rule out that a small underestimation is still present.

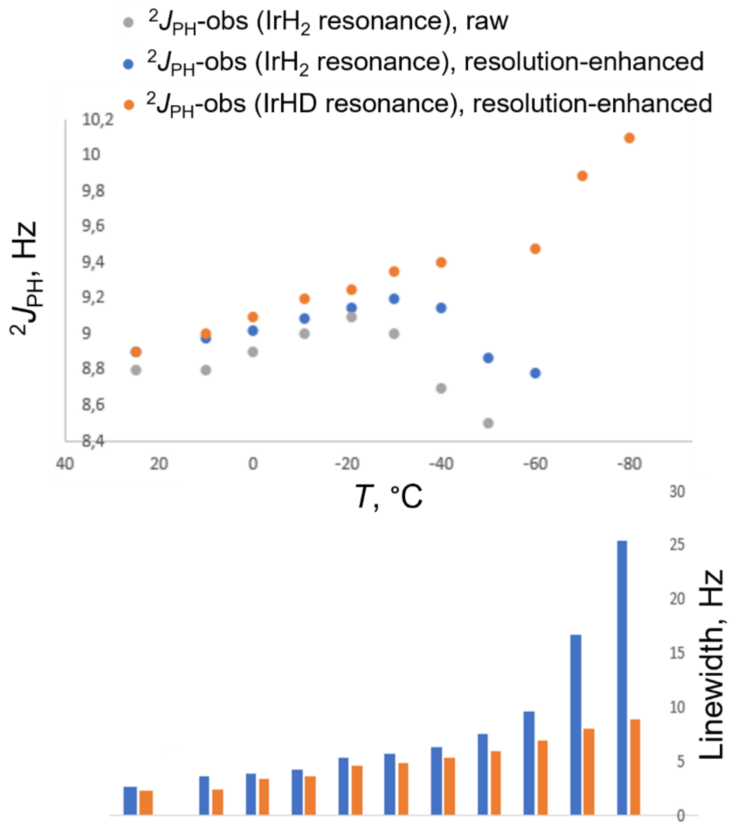

Figure S5. Top: temperature dependence of an "observed" $^2J_{PH}$ in partially deuterated (*m*-bis-CF_3-POCOP)IrH_2 measured at IrH_2 (grey dots) resonances, as well as at IrH_2 (blue dots) and IrHD (orange dots) resonances with resolution enhancement. Bottom: selected linewidths of IrH_2 and IrHD resonances measured in $^1H\{^{31}P\}$ NMR spectra of the same complex. It can be seen that when linewidth approaches $^2J_{PH}$ value, $^2J_{PH}$-obs undergoes decrease that cannot be fully repaired with resolution enhancement.

3.2 Accuracy of fitting

All observed NMR spectra of (X-POCOP)IrH_2 can be successfully fitted with the two-component model (**S** and **NS**), augmented with **NS-bound** (**S**, **NS** and **NS-bound**) in some cases. We suppose that the fittings performed well capture the key processes responsible for the observed spectral changes over the range of temperature. At the same time, the fitted limiting chemical shifts and thermodynamic parameters have comparatively high uncertainties. This is because in some spectra no plateauing is observed and the datasets are thus somewhat degenerate. There are multiple dynamic processes that can affect the chemical shifts and the lineshape of (X-POCOP)IrH_2 such as exchange with traces of dissolved hydrogen or deuterium scrambling with NMR solvents. Although care has been taken to minimize the effects of those, a small additional uncertainty cannot be completely ruled out. A special note shall be made regarding fitting of ^{31}P NMR spectra. ^{31}P NMR signals in general are known to be highly temperature dependent. It could be that rotation of *tert*-butyl groups and similar processes have higher impact on ^{31}P NMR chemical shifts compared to **S-NS** equilibria. Therefore, although in some cases ^{31}P NMR spectra were fitted to

illustrate that they do not contradict the model, the resulting limiting chemical shifts cannot be deemed as reliable.

3.3 Fitting procedures

For the two-component model the data was fitted to the standard equation:

$$X(i)_{obs} = X(i)_A/(1+ e^{(-\Delta H/RT + \Delta S/R)}) + X(i)_B \times e^{(-\Delta H/RT + \Delta S/R)}/(1+ e^{(-\Delta H/RT + \Delta S/R)})$$

where X(i) are δ, $^2J_{PH}$ and J_{HD}.

For more complex models with sophisticated analytical solutions, $X(i)_{obs}$ were numerically processed to obtain K with guess values of $X(i)_A$, $X(i)_B$, etc. for each temperature, and then the sum of least square errors over the whole temperature range was minimized with $X(i)_A$, $X(i)_B$, etc. as parameters.

3.4 Model chemistry

We have attempted different model chemistries to fit temperature dependencies of NMR spectra of the iridium hydrides studied. A simple two-component model

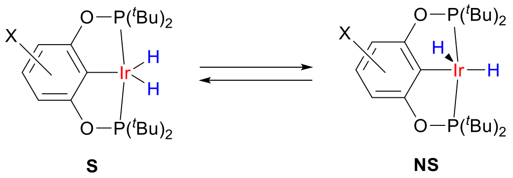

gives good fit of (X-POCOP)IrH_2 with electron-withdrawing groups, and when the effect of linewidth on J_{HD} is carefully addressed, for (X-POCOP)IrH_2 with electron-donating groups as well. Isotope effects on chemical shifts are rationalized through non-statistical occupation of apical and equatorial sites in **NS** by deuterium for (X-POCOP)IrH_2 with electron-withdrawing groups.

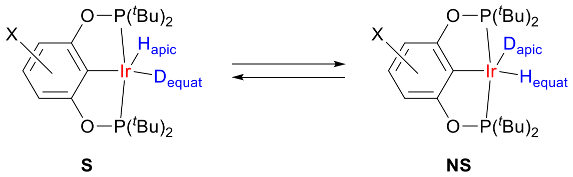

For (X-POCOP)IrH_2 with electron-donating groups, isotope effects on chemical shifts are rationalized through non-statistical distribution of deuterium between **S** and **NS** structures, or in other words, the slight preference of deuterium to occupy sites in **NS** compared to **NS**. An alternative model may involve **S** that is lower than **NS** and the presence of **S-bound** that accounts for a small up-field shift of δ(IrH_2) at low T. Because the changes observed are small, a fully unambiguous choice is not possible.

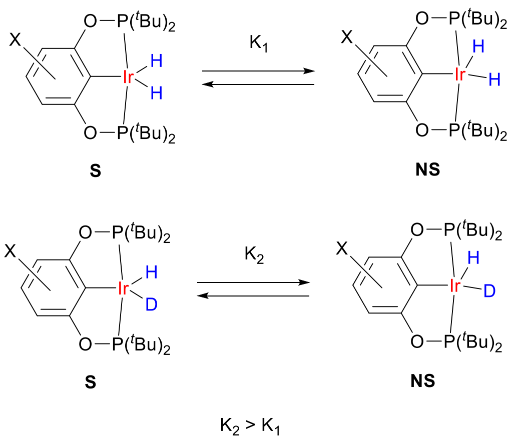

3.5 Complex (*m*-bis-CF_3-POCOP)IrH_2 in toluene-d_8:

^{1}H and ^{31}P chemical shifts, as well as J_{HD} and $^2J_{PH}$ were fitted simultaneously without restrictions. Pleasingly, **S**-J_{HD} of 9.6 Hz well matches the value that can be calculated from *r*(H-H) in (*p*-H-POCOP)IrH_2 determined by neutron diffraction (1.43 A, 8.9 Hz). **NS**-J_{HD} of 0.1 Hz has no reliable comparison, but is in the expected range (-3 to 3 Hz). **NS**-$^2J_{PH}$ of 11.4 Hz is smaller than the related coupling in fully square-pyramidal (X-POCOP)IrHCl (12.5-13.5 Hz). Hence, it is likely that **NS** geometry is in between trigonal-bipyramidal **S** and square-pyramidal (X-POCOP)IrHCl, closer to the latter. It will be shown further on that halogen-bonded adducts with C_6F_5I are even more de-symmetrized. If $^2J_{PH}$ is kept constrained to 12.9 Hz (that is the value for (*m*-bis-CF_3-POCOP)IrHCl)) during fitting, the fitting is converged to **S**-J_{HD} of 12.1 Hz which seems an overestimate (corresponds to *r*(H-H) 1.3 Å).

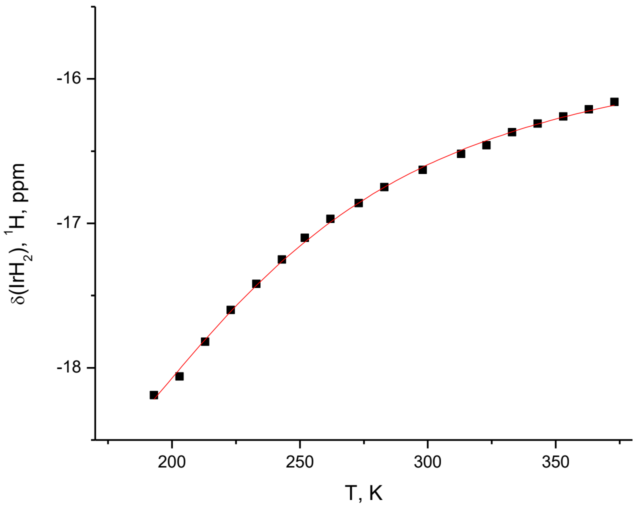

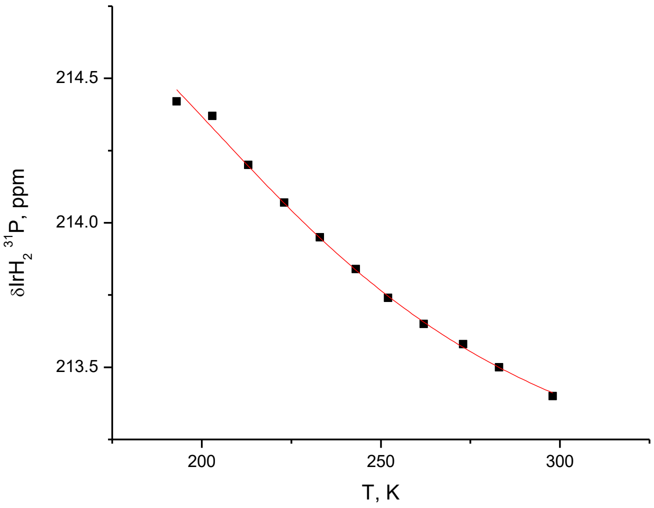

214.5
214.0
213.5
δIrH2 31P, ppm
200
250
300
T, K

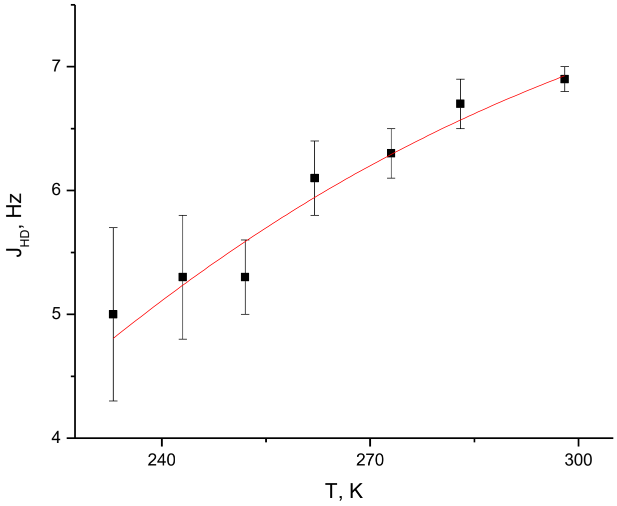

7
6
5
4
JHD, Hz
240
270
300
T, K

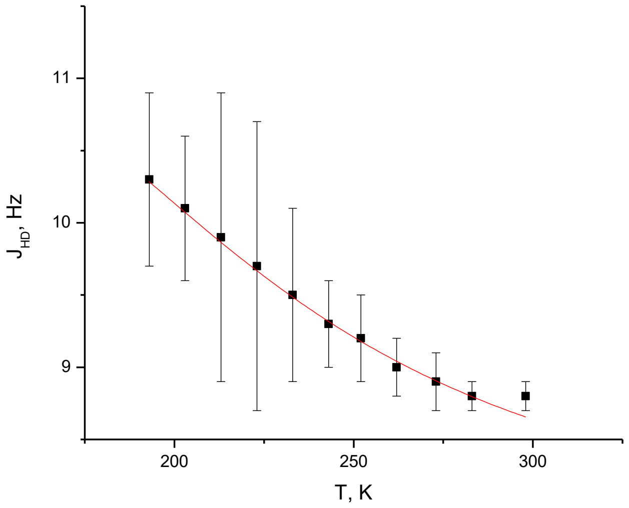

Figure S6. A two-component model (**S** and **NS**) fit of variable temperature NMR spectra of (*m*-bis-CF_3-POCOP)IrH_2 toluene-d_8. ^{1}H and ^{31}P chemical shifts, as well as J_{HD} and $^2J_{PH}$ are fitted.

3.6 Complex (*p*-MeOOC-POCOP)IrH_2 in toluene-d_8:

$^2J_{PH}$ was not fitted due to small amount of accurate data points

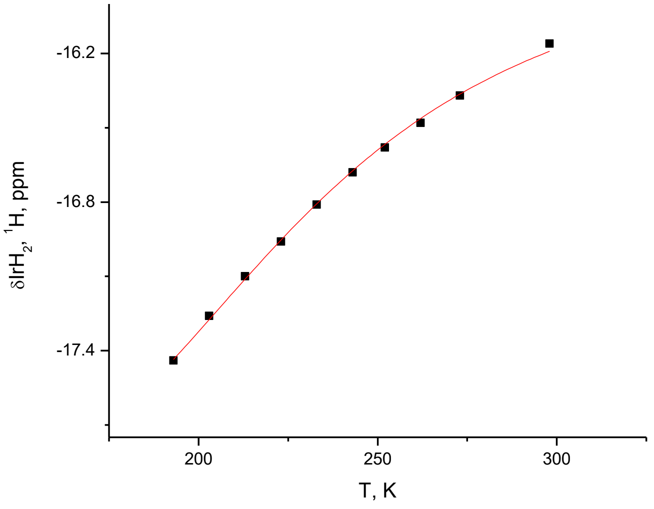

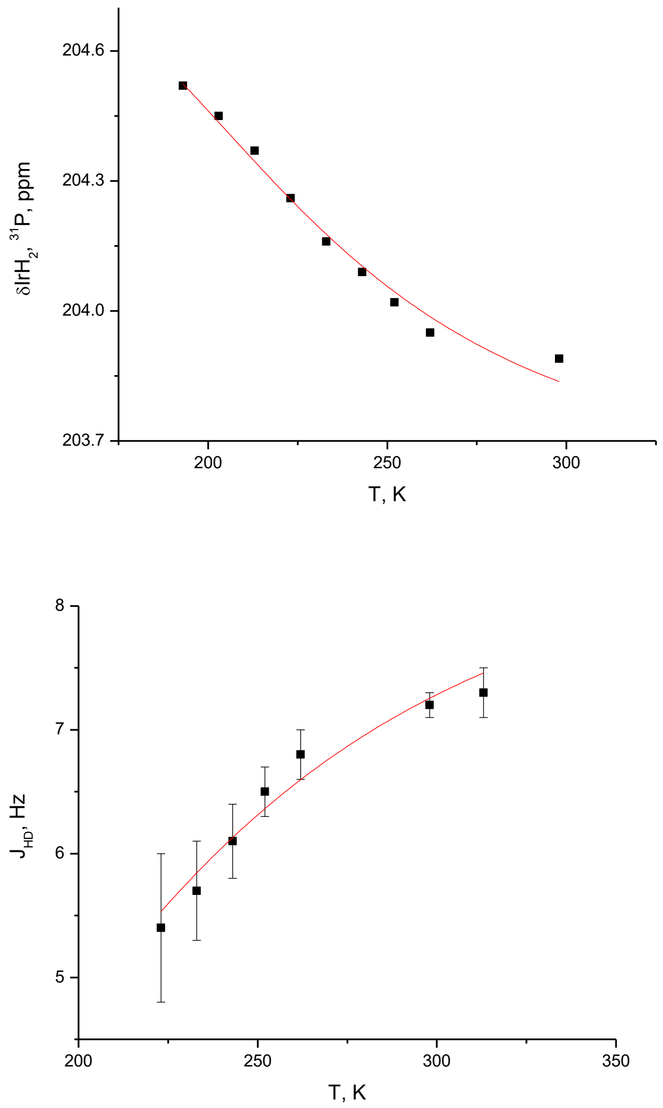

Figure S7. A two-component model (**S** and **NS**) fit of variable temperature NMR spectra of (*p*-MeOOC-POCOP)IrH_2 toluene-d_8. ^{1}H and ^{31}P chemical shifts, as well as J_{HD} are fitted.

3.7 Complexes (*p*-H-POCOP)IrH_2 and (*p*-MeO-POCOP)IrH_2 in toluene-d_8: notes

For these compounds reliable fitting is complicated since change of δ in ^{1}H NMR over +25…-80 °C is much smaller and does not exceed 0.4 ppm, which is in fact comparable to changes of δ in (*p*-H-POCOP)IrHCl (ca. 0.3 ppm). The latter complex is unlikely to be involved in any well-defined equilibria or interactions. Also, no signs of plateauing are observed in the spectra of (*p*-H-POCOP)IrH_2 and (*p*-MeO-POCOP)IrH_2.

The apparent $^{2}J_{PH}$ seem to decrease with temperature in both compounds from 8.2-8.1 to <7.7 Hz, however, when the effect of line broadening is taken into account, only ca. 0.1 Hz changes are left, that are in fact less than the confidence interval. It is clear, at the same time, that unlike complexes

with X= *m*-bis-CF_3 and MeOOC-, $^2J_{PH}$ does not indicate major structural changes happening. An accurate measurement of J_{HD} is not possible due to broad lines, however a decrease of ca. 0.5-1 Hz (after addressing the linewidth) seemingly takes place.

To deal with partial degeneracy of the data, we used ^{1}H and ^{31}P chemical shifts jointly with isotope effects aiming at R^2≈99 for van't Hoff plots for both. Subsequently, the resulting ΔH and ΔS were used to fit J_{HD}. We thus report a possible, but perhaps not a univocal solution. The uncertainties are not possible to estimate. As noted above, there could be an alternative solution using **S**/**S-bound**/**NS** model.

3.8 Complex (*p*-H-POCOP)IrH_2 in toluene-*d*$_8$:

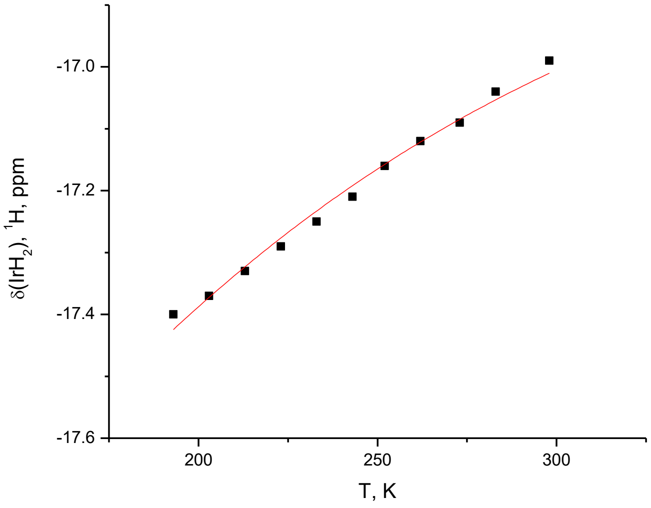

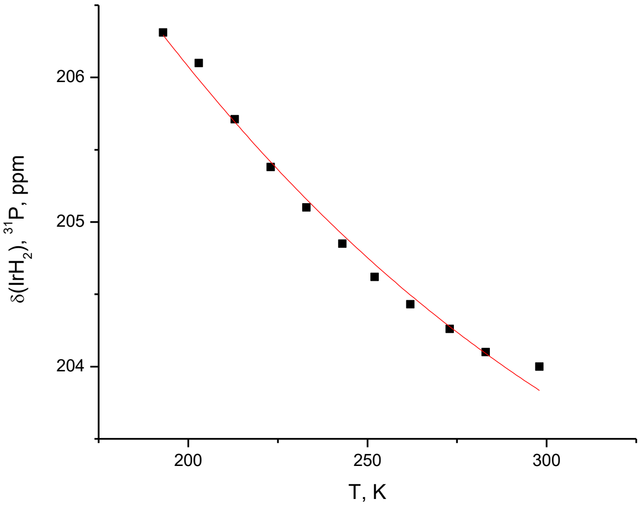

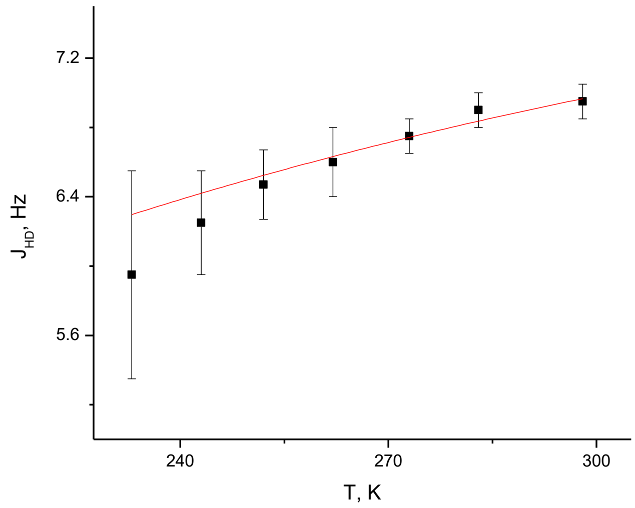

Figure S8. A two-component model (**S** and **NS**) fit of variable temperature NMR spectra of (*p*-H-POCOP)IrH_2 toluene-d_8. ^{1}H and ^{31}P chemical shifts, as well as J_{HD} are fitted.

3.9 Complex (*p*-MeO-POCOP)IrH_2 in toluene-d_8:

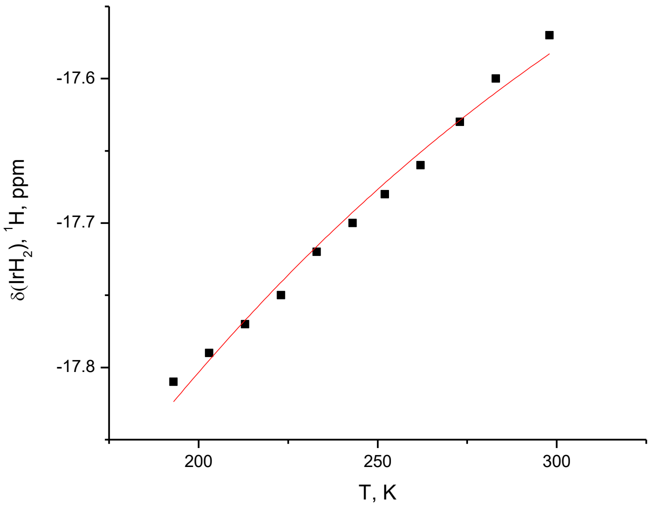

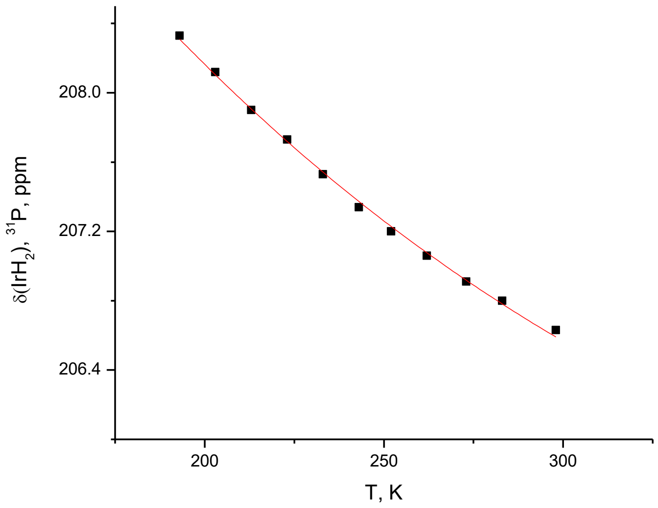

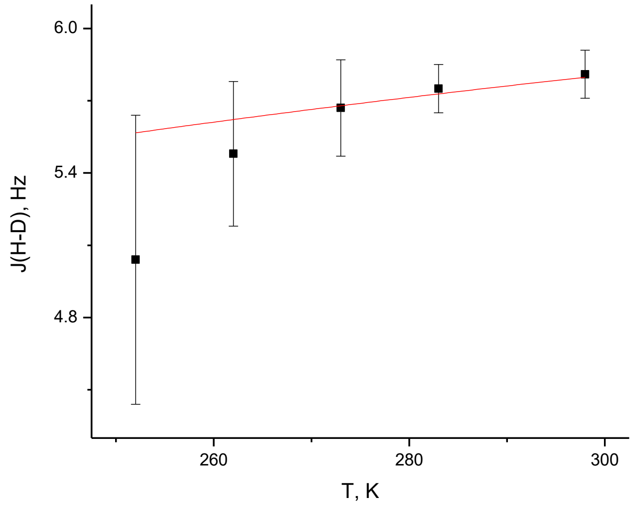

Figure S9. A two-component model (**S** and **NS**) fit of variable temperature NMR spectra of (*p*-MeO-POCOP)IrH_2 toluene-d_8. ^1H and ^{31}P chemical shifts, as well as J_{HD} are fitted.

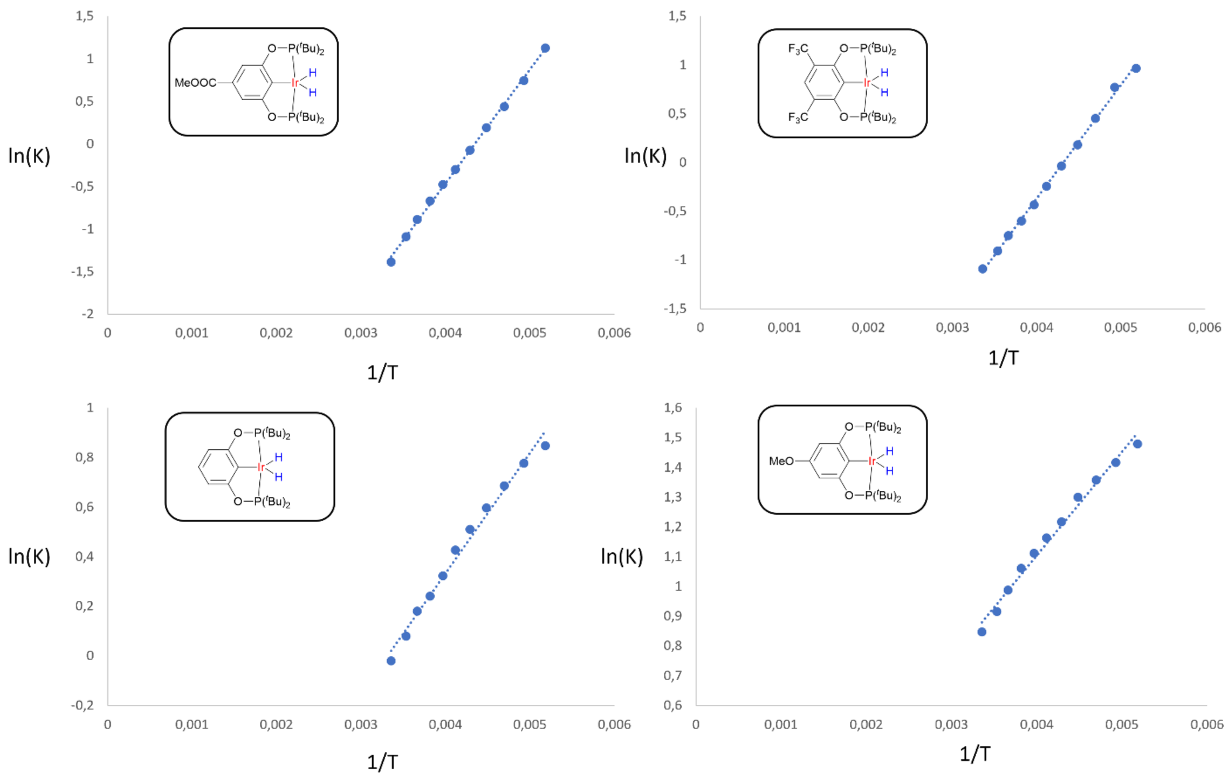

Figure S10. Van't Hoff plots for **S-NS** equilibria in (X-POCOP)IrH_2 over -80…+25 °C.

Table S6. A summary of two-component (**S** and **NS**) model fit of NMR spectra of (X-POCOP)IrH_2 in toluene-d_8.

	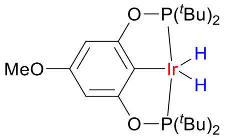	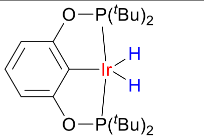	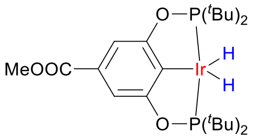	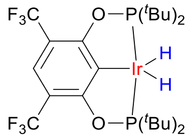
δ**S**, ^{1}H, ppm	-16.1	-16.0	-15.1±0.6	-15.6±0.4
δ**S**,^{31}P ppm	196	198	203.3±0.3	212.7±0.3
J_{HD} **S**, Hz	~9-11	~9-11	9.2±1.3	9.6±1.1
$^2J_{PH}$ **S**, Hz	-	-	-	7.6±0.4
δ**NS**,^{1}H ppm	-18.2	-18.0	-18.6±1.3	-19.3±0.7
δ**NS**,^{31}P ppm*	211	210	205.1±0.7	215.2±0.5
J_{HD} **NS**, Hz	~3-5	~3-5	2.2±2.9	0.1±2.0
$^2J_{PH}$ **NS**, Hz	-	-	-	11.4±0.7
ΔH, kcal/mol	-0.7	-1.0	-1.9±1.1	-2.0±0.6
ΔS, cal×mol^{-1}×K^{-1}	-0.7	-3.2	-8.1±3.8**	-8.6±2.3**

*Note that ^{31}P chemical shifts are well-known to be temperature dependent, and may also change because of the reasons that are not connected with **S**/**NS** equilibrium.** The entropy values may indicate participation of toluene in stabilization of **NS**.

Table S7. Experimental and calculated distances and energies relevant to **S** and **NS** structures in (X-POCOP)IrH_2 complexes in toluene.

		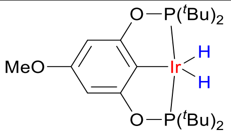	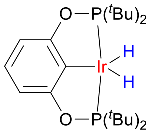	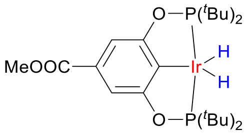	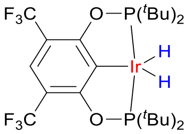
r(H-H) in S, exp., Å	Neutron diff.	-	1.43	-	-
	$J_{\text{HD-fitted}}$	~1.4	~1.4	1.42	1.40
	T_1(min)	<1.57	<1.54	<1.70	<1.70
r(H-H) in **S**, calc., Å	D3BJ-revPBE	1.64	1.60	1.57	1.57
	D3BJ-revPBE // DLPNO-CCSD(T)	1.57±0.05	1.62±0.05	-	-
r(H-H) in **NS**, exp., Å	$J_{\text{HD-fitted}}$	~1.7	~1.7	~2	~2
r(H-H) in **NS**, calc., Å	D3BJ-revPBE	1.64	1.63	2.07	1.99
	D3BJ-revPBE // DLPNO-CCSD(T)	2.14±0.05	2.08±0.05	-	-
ΔH-exp.	fitted	Ca. -0.7	Ca. -1.0	-1.9±1.1	-2.0±0.6
ΔS-exp.	fitted	Ca. -0.7	Ca. -3.2	-8.1±3.8	-8.6±2.3
ΔH-calc	D3BJ-revPBE	+1.15	-0.06	-0.69	-0.45
	revPBE// DLPNO-CCSD(T)	+1.21	-0.27	-1.88	-1.04
ΔS-calc	D3BJ-revPBE	-0.52	-0.46	-1.78	-3.62

4. Evaluating isotope effects in (X-POCOP)IrH_2

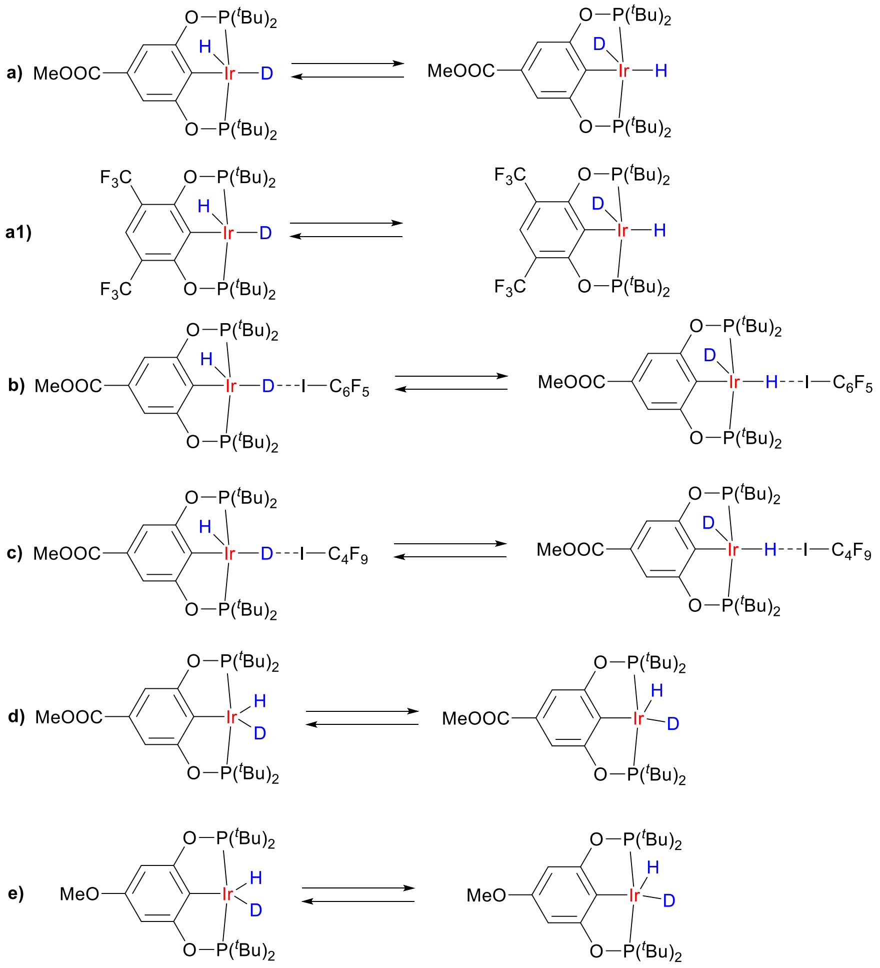

Figure S11. Equilibria relevant to isotope effect measurements in (X-POCOP)IrH_2.

A reliable, although not very precise, estimate of thermodynamics of substitution with deuterium is only available for the adduct of (*p*-MeOOC-POCOP)IrH_2---IC_4F_9 (Figure S11, (c)). This compound is static at low temperatures, that allowed to measure the affinity of deuterium for the apical position (Figure S12).

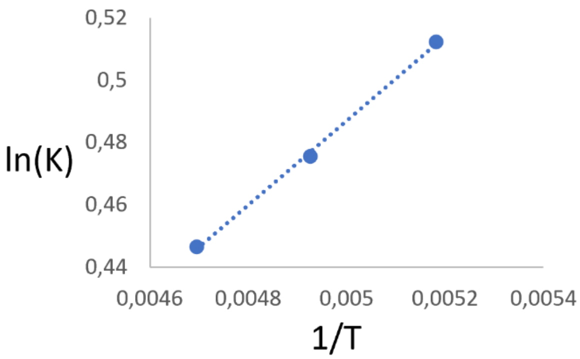

Figure S12. A van't Hoff plot for (p-MeOOC-POCOP)IrH$_{apical}$D$_{equatorial}$---IC$_4$F$_9$ ↔ (p-MeOOC-POCOP)IrH$_{equatorial}$D$_{apical}$---IC$_4$F$_9$ for at -60, -70 and -80 °C. ΔH = -0.27±0.01 kcal/mol and ΔS = -0.37±0.06 cal×mol^{-1}×K^{-1}.

The following equation was used to calculate K using experimental Δδ, that can be readily derived from the definition of K:

$$\Delta\delta = \delta_{HD} - \delta_{HH} = \frac{(\delta_A - \delta_B)(1-K)}{2(K+1)}$$

Where K is the equilibrium constant and δ_A and δ_B are the following chemical shifts:

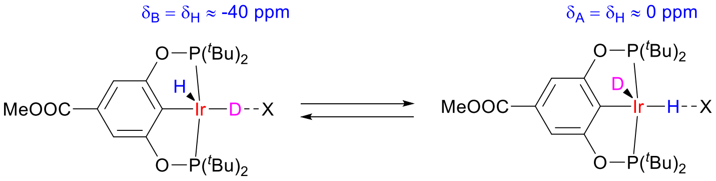

Experimental data on δ_A and δ_B are not available, but based on the NMR calculations we estimate δ_B as -40±2 ppm (which can be compared to experimental value for iso-structural (p-MeOOC-POCOP)IrHCl δ_H = -40.12 ppm) and δ_A as 0±5 ppm (which does not have a close analogue to compare, for somewhat related (p-H-POCOP)IrH(H$_2$)$^+$ with dihydrogen ligand *trans* to aryl, δ_H = 0.3 ppm). Hence, we take δ_A - δ_B ≈ 40 ppm.

For other complexes, some assumptions are to be made. Thus, we suppose δ_A - δ_B ≈ 40 ppm stays for (p-MeOOC-POCOP)IrH$_2$---IC$_6$F$_5$ adduct as well. Simplistically assuming that only **S** and **NS-bound** are present in the model, and δ(**S**-IrH$_2$) ≈ δ(**S**-IrHD), a fit gives a linear van't Hoff plot with ΔH = -0.34±0.01 kcal/mol and ΔS = -0.92±0.05 cal×mol^{-1}×K^{-1} (Figure S13), that are already fairly close to the previous estimate for related C$_4$F$_9$I (Figure S12). A more sophisticated **S**/**NS**/**NS-bound** model provides ΔH = -0.20±0.01 kcal/mol and ΔS = -0.03±0.05 cal×mol^{-1}×K^{-1}, that are in a better agreement with the DFT calculated values (ΔH = -0.23 kcal/mol and ΔS = +0.00061 cal×mol^{-1}×K^{-1})

For (p-MeOOC-POCOP)IrH$_2$ and (m-bis-CF$_3$-POCOP)IrH$_2$ in toluene-d_8, in order to obtain a good fit of Δδ one needs either to adjust δ(**S**) and, optionally, δ(**NS**), or to assign comparatively high "intrinsic" isotope chemical shifts to **S** and, optionally, **NS**. Thus, leaving δ(**S**) intact requires

ascribing an "intrinsic", temperature-independent Δδ of -0.4 and -0.3 ppm to the above-mentioned complexes, respectively. This is larger than the common range of "intrinsic" Δδ -s for transition metal hydrides (±0.02-0.1 ppm, more rarely up to ±0.2 ppm[15,16]), but still reasonable, especially in view of theories that deuterium can induce de-symmetrization in certain molecules,[17] that in the case of **S** should have a dramatic effect. Besides, it would seem that δ(IrH_2) and δ(IrHD) are asymptotically approaching a comparable Δδ at high-temperature limit. It is also possible that one needs to adjust both δ(**S**) and Δδ in balanced manner. Note also that for a wide temperature range van't Hoff plots for isotope effects may not necessarily be linear.[18]

Taking the δ_A - δ_B as ca. 30 ppm based on the fitted J_{HD}'s and calculated δ-*r*(H-H) dependence, and using the above-made assumptions as well as taking **S** to **NS** ratio from δ(IrH_2), a fit of δ(IrHD) would produce ΔH = -0.061±0.002 kcal/mol and ΔS = 0.142±0.007 cal×mol^{-1}×K^{-1} for deuterium positioning at apical vs. equatorial sites. These values are close to the calculated ones (Table S8) and argue for consistency of the model, but they are dependent on the assumptions made.

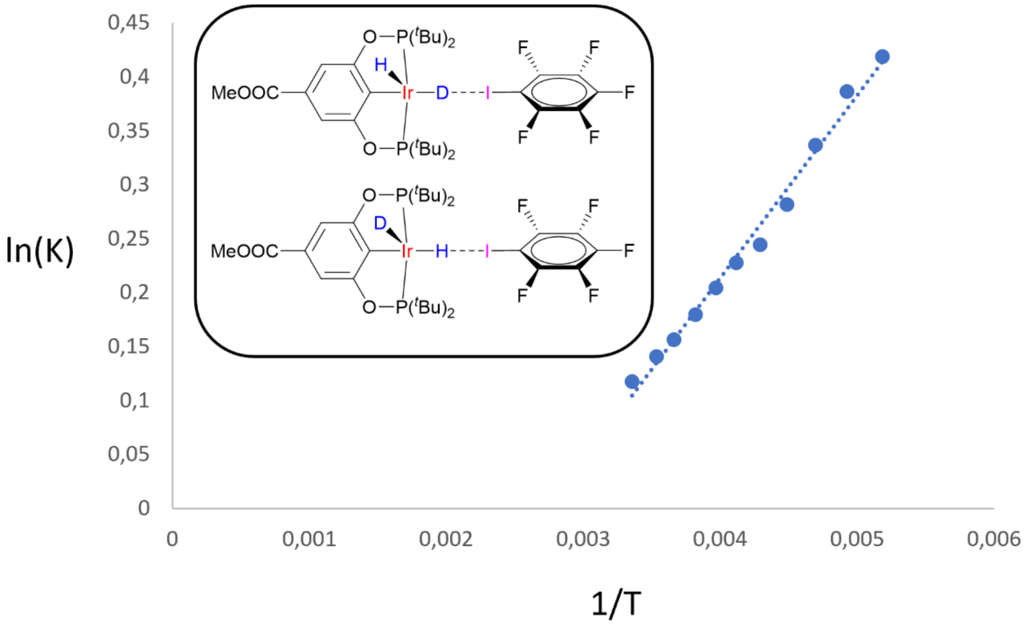

Figure 13. A van't Hoff plot for (*p*-MeOOC-POCOP)IrH$_{apical}$D$_{equatorial}$---IC_6F_5 ↔ (*p*-MeOOC-POCOP)IrH$_{equatorial}$D$_{apical}$---IC_6F_5 over +25…-80 °C using **S**/**NS-bound** model.

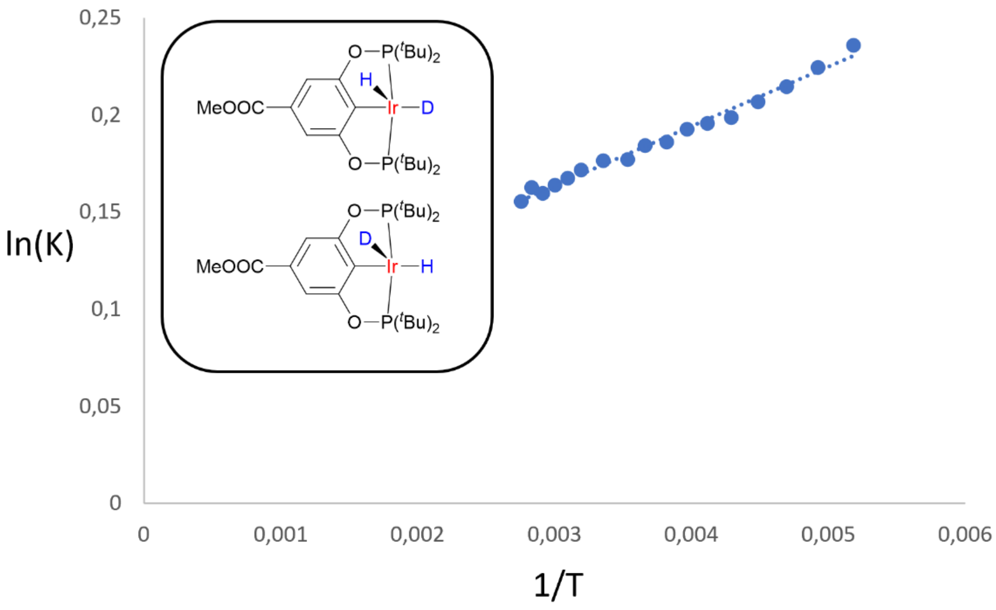

Figure S14. A van't Hoff plot for (p-MeOOC-POCOP)Ir$H_{apical}D_{equatorial}$ ↔ (p-MeOOC-POCOP)Ir$H_{equatorial}D_{apical}$ in toluene-d_8 over +100…-80 °C using **S/NS** model..

For (p-MeO-POCOP)IrH_2 at low temperatures δ(IrHD) is observed ca. 0.15 ppm to high-field from δ(IrH_2) (Table S4). This is a "normal" direction[19] (and magnitude) of Δδ and this probably reflects the "intrinsic" Δδ in **S**-(p-MeO-POCOP)IrH_2. In complex (PCP)IrH_2, where **NS** presumably is not populated, Δδ is also high-field from δ(IrH_2). Hence, for (p-MeO-POCOP)IrH_2 the small temperature dependence of Δδ can be explained by: (a) a minor population of **NS** at elevated temperatures, if **S** is lower in energy than **NS**; (b) a temperature dependent "intrinsic" Δδ; and (c) *intermolecular* isotope perturbation of equilibria, or the higher affinity of deuterium for **NS** structure, if **NS** is lower in energy than **S**. If model (c) is chosen, then, assuming there is a temperature independent intrinsic isotope effect of 0.15 ppm, a van't Hoff plot for δ(IrHD) using chemical shifts from δ(IrH_2) gives ΔH = -1.1 kcal/mol and ΔS = -2.5 cal×mol^{-1}×K^{-1}, and after subtraction of values for δ(IrH_2) fit, isotope effect is obtained as ΔH = -0.4 kcal/mol and ΔS = -1.8 cal×mol^{-1}×K^{-1}.

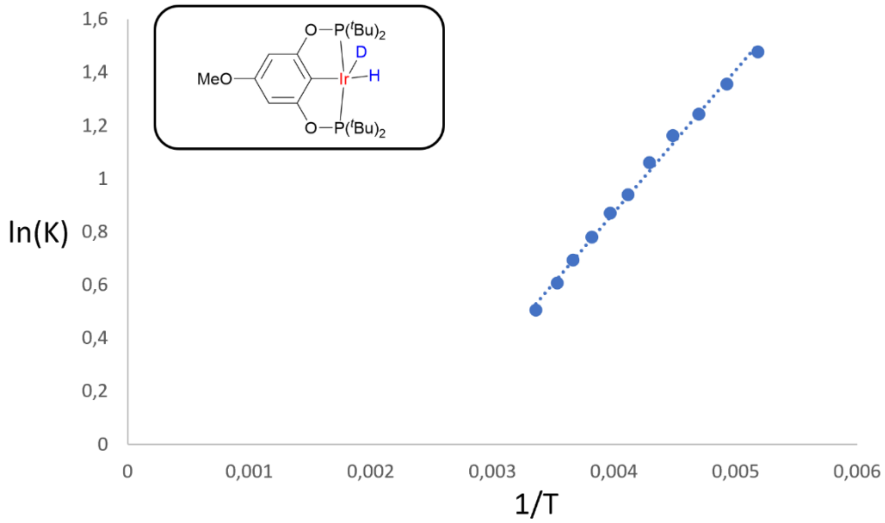

Figure S15. A van't Hoff plot for (*p*-MeO-POCOP)IrHD using a two-component model with chemical shifts taken from (*p*-MeO-POCOP)IrH_2 (see above) and using intrinsic isotope effect correction of 0.15 ppm for both **S** and **NS** structures. Temperatures range +25…-80 °C.

Table S8. Experimental and calculated isotope effects on chemicals shifts in (X-POCOP)IrH_2. For experimental values, mathematical standard errors are provided, that are likely smaller than the real errors in the case of the complex system studied.

**Reaction**	**ΔH-exp, kcal/mol**	**ΔS-exp, cal×mol^{-1}×K^{-1}**	**ΔH-calc, kcal/mol**	**ΔS-calc, cal×mol^{-1}×K^{-1}**
a)	-0.061±0.002	+0.142±0.007	-0.13	+0.11
a1)	-0.10±0.003	-0.04±0.01	-	-
b)	-0.20±0.01	-0.03±0.05	-0.23	+0.00061
c)	-0.27±0.01	-0.37±0.06	-0.23	+0.022
d)	-	-	(IrH_2: -0.69)<br>(IrHD: -0.68)<br>(for H-apic)<br>0.01	(IrH_2: -3.62)<br>(IrHD: -3.62)<br>(for H-apic)<br>0
e)	(IrH_2: -0.7)<br>(IrHD: -1.1)<br>-0.4	(IrH_2: -0.7)<br>(IrHD: -2.5)<br>-1.8	(IrH_2: 1.15)<br>(IrHD: 1.04)<br>-0.11	(IrH_2: -0.52)<br>(IrHD: -0.39)<br>+0.13

5. IR spectroscopy of (X-POCOP)IrH_2

Table S9. Experimental and calculated IR spectra of (X-POCOP)IrH_2

**X =**	*p*-MeO-	*p*-H-	*p*-MeOOC-
Hexane	2084 (br m),<br>2103 (w)	1890 (w br)?<br>~2090 (br m),<br>2110 (w)	1908 (br, w)?<br>2118 (m)
CH_2Cl_2	2086 (br),<br>1990 (br)?,<br>1933 (br, vs)?	~1898 (br s)<br>~2100 (br m),	1899 (vbr, s)?<br>2121 (m)
hexane+C_6F_5I	n/d	n/d	~1860<br>(br, vs)<br>2119 (m),
Calc.-**S**	2124 (s),<br>2136 (w)	2133 (s),<br>2146 (w)	2135 (s),<br>2153 (w)
Calc.-**NS**	2093 (s)<br>2172 (w)	2058 (s),<br>2225 (w)	1891 (s)<br>2419 (w)
Calc.-**NS-bound** (with C_6F_5I)	1903 (vs),<br>2318 (vw)	1858 (vs),<br>2405 (vw)	1865 (vs),<br>2422 (vw)
Calc.-**NS-bound** (with CH_2Cl_2)	n/d	2019 (vs)<br>2310 (vw)	1944 (vs)<br>2408 (vw)

Abbreviations: br – broad, vbr – very broad, vs – very strong, s -strong, m – medium, w -weak, vw -very weak. (?) refers to ambiguous assignment.

In the IR spectra of (*p*-MeO-POCOP)IrH_2 and (*p*-H-POCOP)IrH_2 in hexane, two main bands are observed: one sharp (at 2103 and 2110 cm^{-1}, respectively), and one broad and more intense (at ca. 2084 and ca. 2090 cm^{-1}, respectively). Although a few other bands could be occasionally noted, they are all seemingly assigned to traces of the respective IrH_4 complexes and decomposition products. Complex (*p*-MeOOC-POCOP)IrH_2 revealed only a sharp band at 2119 cm^{-1}, also, a weak broad band at 1908 cm^{-1} was observed. The latter is in the region that overlaps with IR signals of the respective IrH_4 complex, and therefore the assignment to IrH_2 is ambiguous.

Computationally, within **S** structure, Ir-H stretchings are coupled and form two bands separated by 10-15 cm^{-1}, one intense for near-symmetrical vibration and one weak for non-symmetrical vibration. In **NS** structures the coupling is gradually broken upon increasing de-symmetriztion, and ultimately two separated Ir-H stretches corresponding to apical (very weak) and equatorial (very strong) hydrogens appear; those could differ by more than 400 cm^{-1}.

Our interpretation is that sharp bands in hexane solution of (*p*-MeO-POCOP)IrH_2 and (*p*-H-POCOP)IrH_2 correspond to symmetric vibration in **S**, while broad bands are attributed to symmetric vibration in **NS**. For (*p*-MeOOC-POCOP)IrH_2 **S** band is at 2118 cm^{-1}, while **NS** band

corresponding to equatorial Ir-H stretch is possibly at 1908 cm^{-1}. Moving from hexane to CH_2Cl_2 leaves the **S** band almost unchanged, while a new strong and broad adsorption rises at ca 1900 cm^{-1} for (*p*-H-POCOP)IrH_2 and (*p*-MeOOC-POCOP)IrH_2. That is attributed to equatorial Ir-H stretch in **NS-bound** form of these compounds. Addition of C_6F_5I to hexane solution of (*p*-MeOOC-POCOP)IrH_2 resulted in appearance of a new broad band at ca. 1860 cm^{-1}, that matches the solid-state absorption in (*p*-MeOOC-POCOP)IrH_2---IC_6F_5 at 1870 cm^{-1}, and is close to computational prediction for that compound.

6. Halogen bond strength estimation

6.1 General notes.

Quantitative measurement of halogen bond strength is complicated by the complexity of the system. All halogen bond formation reactions with C_4F_9I strongly interfere with formation of $IrH(C_4F_9I)H$ (see Figure 7 in the manuscript for an example) and therefore are not suitable for fitting. $(PCP)IrH_2$ reacts rapidly with C_6F_5I at near ambient temperatures and undergoes considerable $(PCP)IrH(C_4F_9I)H$ formation at low temperatures, and data for that complex is not suitable for fitting as well.

Therefore only $(X\text{-}POCOP)IrH_2/C_6F_5I$ system is left, where we suppose estimates, but not precise measurements, of binding energies could be obtained. Some of the possible error sources are:

a) competing coordination of C_6F_5I to Ir with formation of $IrH(C_6F_5I)H$
b) different **S-NS** equilibria in toluene-d_8 compared to equilibria the presence of C_6F_5I co-solvent
c) reaction of hydrides with C_6F_5I with formation of corresponding iodides
d) possible involvement of other coordination modes (for example as depicted on Figure 8) at certain conditions

Those factors were addressed as following:

a) Based on the low-temperature measurements of separated signals (where observed), extrapolation suggests that at most of the temperatures $K_{(IrH2---C6F5I)}/K_{(IrH[C6F5I]H)} \geq 50\text{-}100$ and thus (a) shall introduce comparatively small error.
c) It was accounted for numerically, through adjusting concentrations.
b) We believe b) is not significant for X = *p*-MeO- and *p*-H-, but for *p*-MeOOC and *m*-bis-CF_3 it may introduce errors and perhaps is responsible for deviations of experimental titration curves from theoretical ones.

6.2 Fitting of δ(IrH_2)-*T* dependence

To extract binding parameters of C_6F_5I to $(X\text{-}POCOP)IrH_2$, we have attempted fitting of δ(IrH_2)-*T* dependance in the presence of constant amount of C_6F_5I. We tried **S/NS-bound** and **S/NS/NS-bound** models, both were found to reproduce the experimental curves. The latter is believed to be more relevant, and therefore all results are given according to **S/NS/NS-bound** model. We have used **S** and **NS** chemical shifts obtained from fit in toluene. **NS-bound** chemical shifts give a “soft” convergence to span of ca. -21.7…-23 ppm, of which all values acceptably reproduce the experimental curves (see Figure S16 for the curves and the respective van’t Hoff plots). Residual errors slightly favored -22.0 ppm X = *p*-MeOOC- and -22.5 ppm for X = *p*-MeO-, *p*-H- and *m*-bis-CF_3. In an attempt to minimize the influence of competing dynamic processes, we then used these chemical shifts to re-fit the four lowest-temperature data points where formation of **NS-bound** is expected to be dominating. The resulting ΔH and ΔS values are given in Table 2 in the manuscript. The only directly observed chemical shift of completely frozen, static halogen bond adduct of the type **NS-bound**, observed for (*p*-MeOOC-POCOP)IrH_2---C_4F_9I (-22.06 ppm), is in good agreement with the fitted data (-22.0 ppm for X = *p*-MeOOC-).

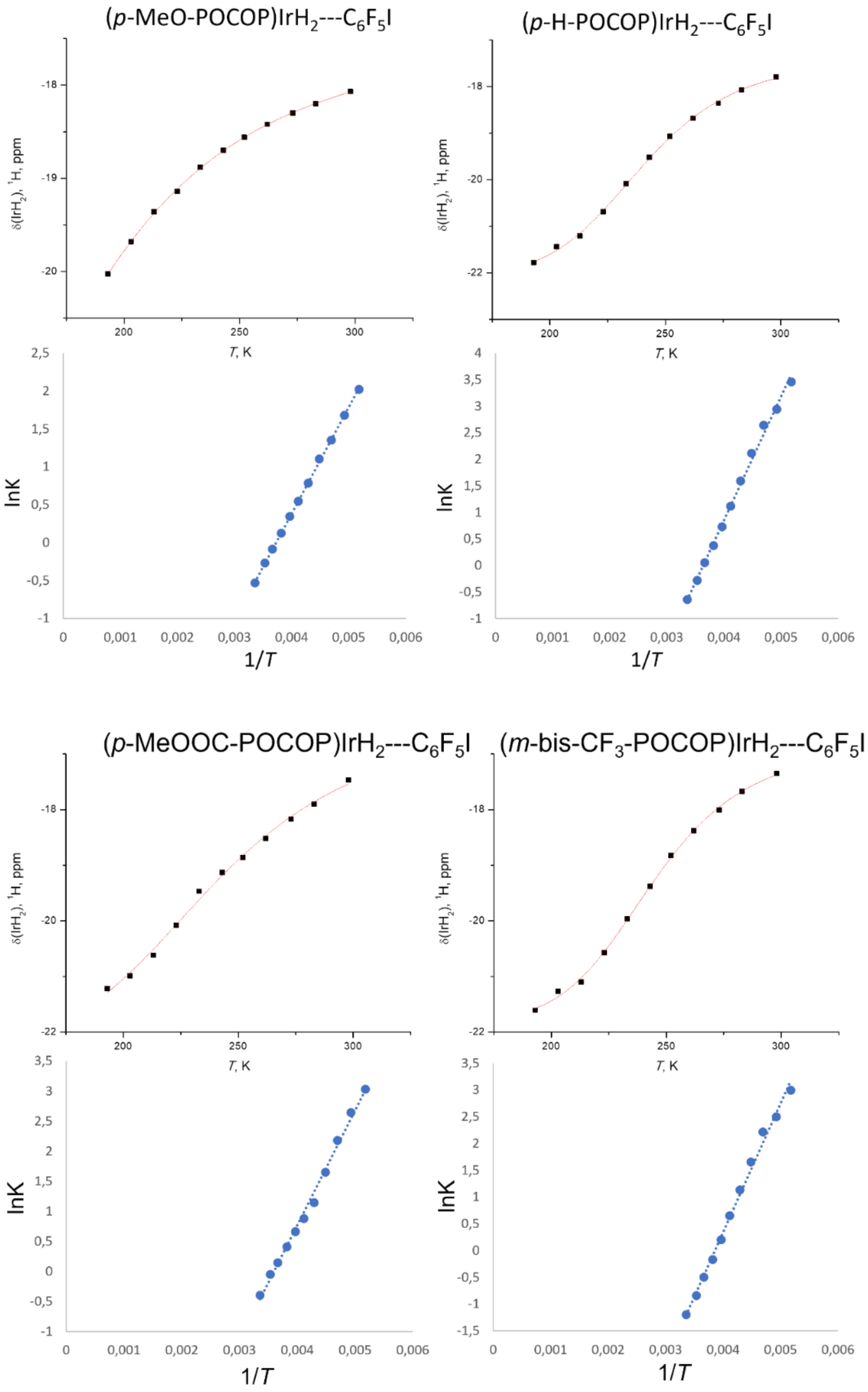

Figure S16. Fit of ^{1}H NMR chemical shifts for (X-POCOP)IrH_2/C_6F_5I/toluene-d_8 system using **S**/**NS**/**NS-bound** model and the respective van't Hoff plots.

6.3 Fitting to 1:1 binding isotherm

Also, titration of (X-POCOP)IrH_2 with C_6F_5I and subsequent fitting to 1:1 binding isotherm was attempted. For X = *p*-H- such titration was performed at three different temperatures, such as ΔH and ΔS can be extracted. Example curves for X = *p*-MeO- and van't Hoff plot for X = *p*-H- are given on Figures S17 and Figure S18, respectively. We noted some deviations from theoretically calculated curves that are small for X = *p*-MeO- but more significant for electron-withdrawing groups. We attribute it to **S-NS** equilibria change upon mixing of toluene with C_6F_5I, although the involvement of competing coordination modes of C_6F_5I cannot be excluded. The "mathematical" errors of ΔH are within 0.1-0.3 kcal/mol, however we suppose in the light of above-mentioned problems, the real errors could reach ±1 kcal/mol for ΔH and ±5 $cal \times mol^{-1} \times K^{-1}$ for ΔS.

Note that $^2J_{PH}$ and J_{HD} can also be used for fitting to 1:1 isotherm. Since the coupling constants are measured with less accuracy compared to chemical shifts and often are not resolved, we used ΔH and ΔS from δ(IrH_2) to obtain values for **NS-bound**. Thus, $^2J_{PH}$ and J_{HD} of ca. 13 Hz and ca. -3 Hz, respectively, were obtained, indicating SP geometry of **NS-bound**. Since J_{HD}-s are smaller and more sensitive to linewidth, we suppose the associated decay may not be fully filtered out, and thus **NS-bound**-J_{HD} may be slightly overestimated.

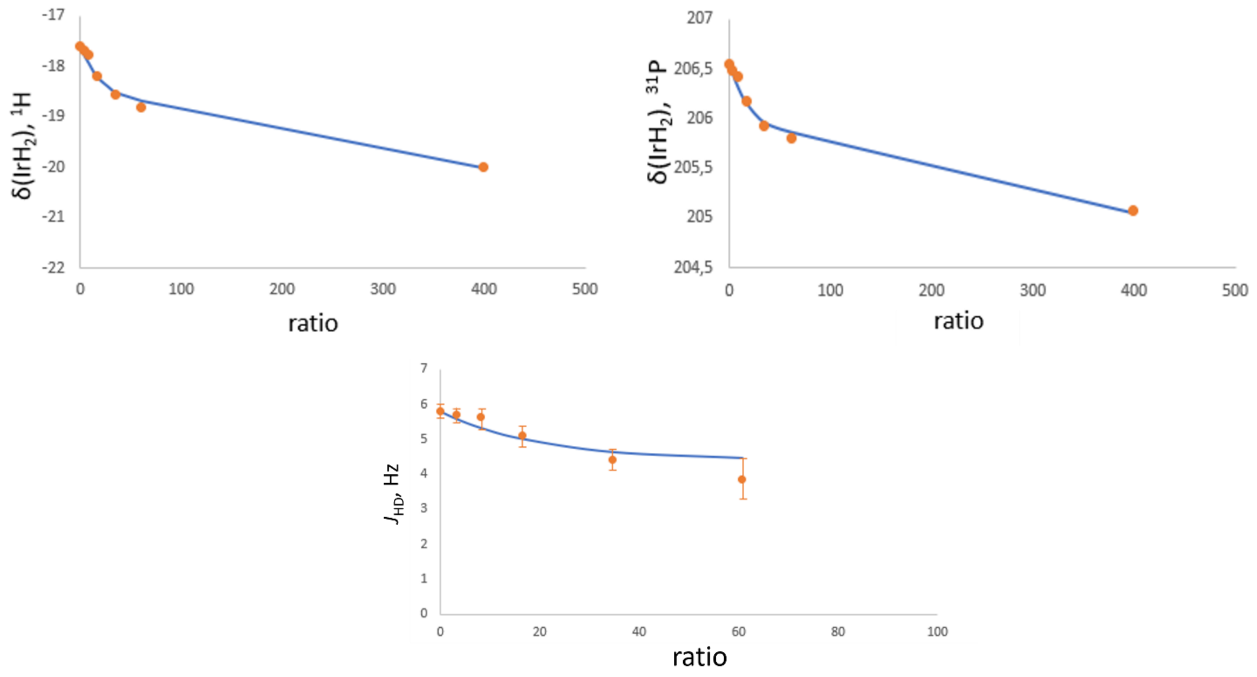

Figure S17. Titration of (*p*-MeO-POCOP)IrH_2 with C_6F_5I in toluene-d_8 followed with NMR. Orange - experimental values, blue – calculated curves.

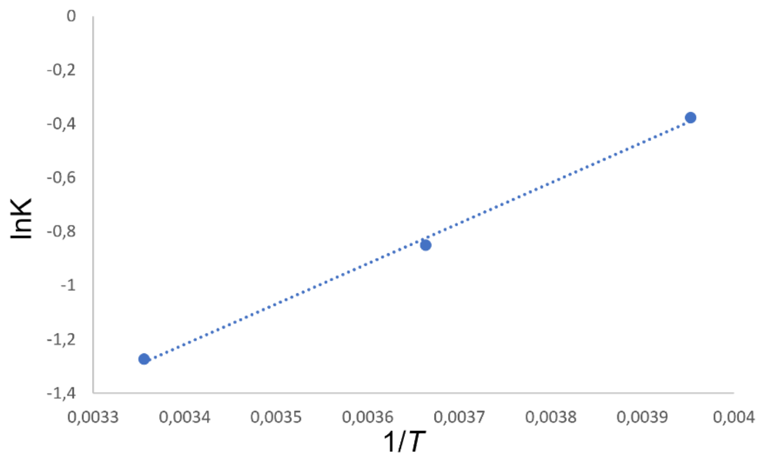

Figure S18. A van't Hoff plot for titration of (*p*-H-POCOP)IrH_2 with C_6F_5I in toluene-d_8 at +25 °C, 0 °C and -20 °C.

6.4 Analysis of (*p*-MeOOC-POCOP)IrH_2---C_4F_9I ↔ (*p*-MeOOC-POCOP)IrH(C_4F_9I)H equilibrium

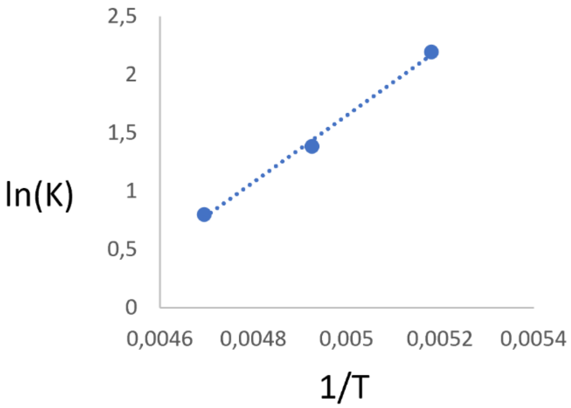

Figure S19. A van't Hoff plot for (*p*-MeOOC-POCOP)IrH_2---C_4F_9I ↔ (*p*-MeOOC-POCOP)IrH(C_4F_9I)H equilibrium at -60, -70 and -80 °C. $\Delta H = -5.7 \pm 0.4$ kcal/mol and $\Delta S = -25 \pm 2$ cal×mol^{-1}×K^{-1}.

7. Dihydrogen bonding with CH_2Cl_2

S/**NS-bound** model was used. The NMR datasets were found to be difficult to converge to unambiguous solutions. Therefore, we used the limiting chemical shifts obtained for **S** structures in toluene-d_8 as constrains, other paraments were fitted freely. Based on computational results, we suppose that the geometry of **S** structures are little dependent on medium and such restriction is justified. Thus, for **S**-(*p*-H-POCOP)IrH_2 *r*(H-H) calculated for in toluene is 1.60 Å, while in CH_2Cl_2 1.62 Å. Completely relaxed fits for X = *p*-MeOOC and *m*-bis-CF_3 converge not too far from Table S10 data.

Complex (*m*-bis-CF_3-POCOP)IrH_2 in CD_2Cl_2:

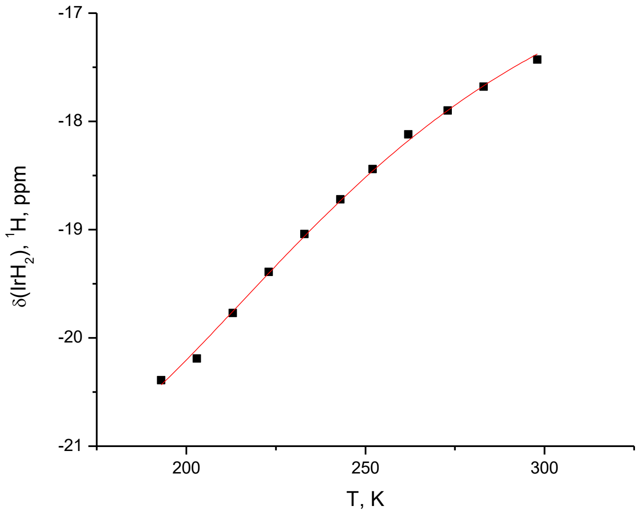

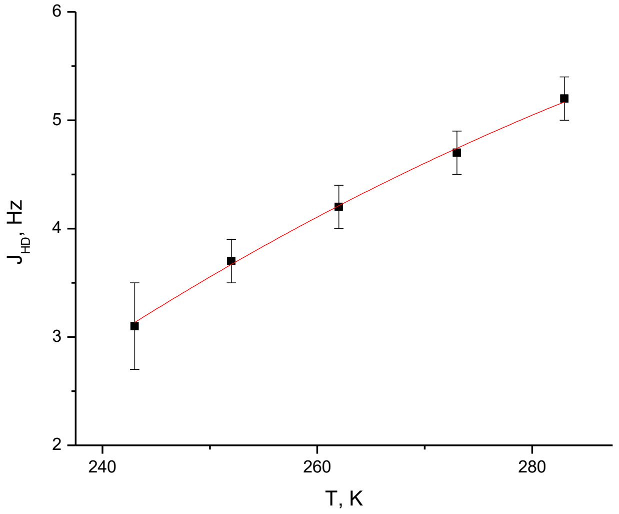

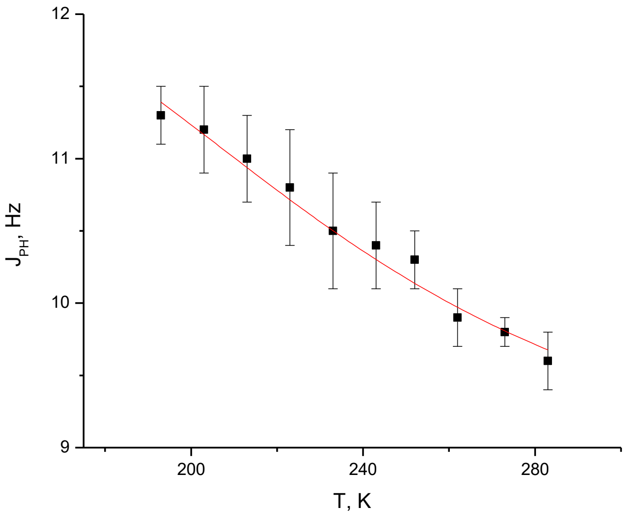

Figure S20. A two-component model (**S** and **NS-bound**) fit of variable temperature NMR spectra of (*m*-bis-CF_3-POCOP)IrH_2 in CD_2Cl_2. ^{1}H chemical shifts, as well as J_{HD} and J_{PH} are fitted.

Complex (*p*-MeOOC-POCOP)IrH_2 in CD_2Cl_2:

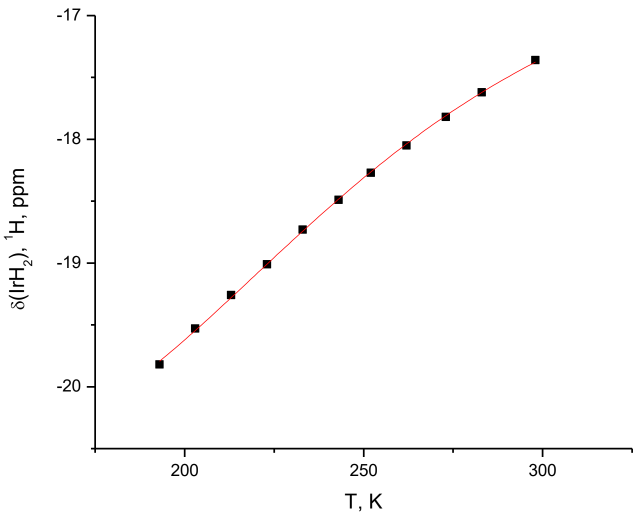

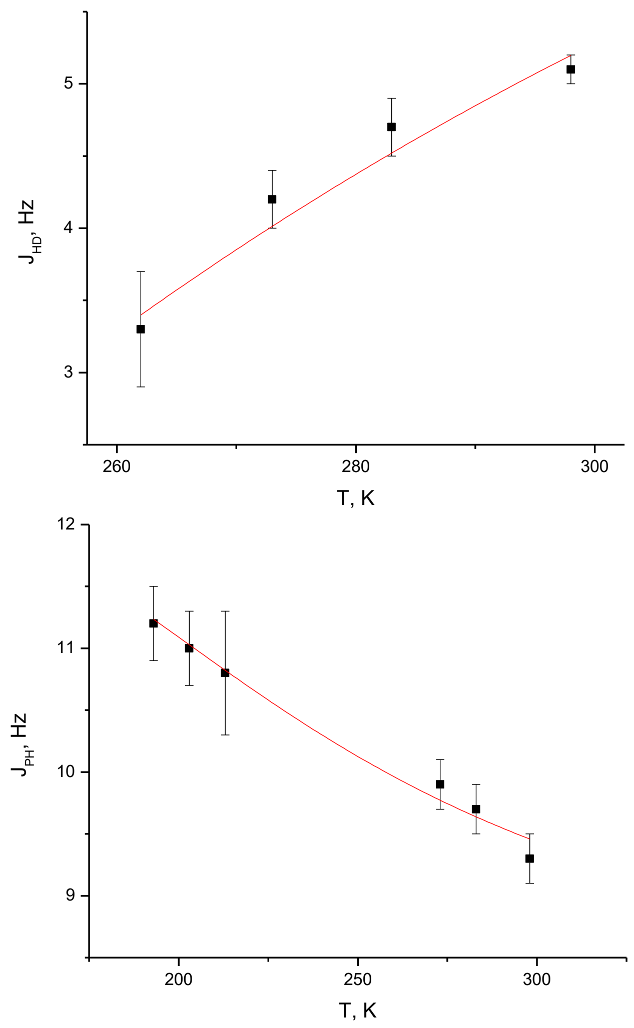

Figure S21. A two-component model (**S** and **NS-bound**) fit of variable temperature NMR spectra of (*p*-MeOOC-POCOP)IrH_2 in CD_2Cl_2. ^{1}H chemical shifts, as well as J_{HD} and J_{PH} are fitted.

Complex (*p*-H-POCOP)IrH_2 in CD_2Cl_2:

Only ^{1}H data was acquired and fitted.

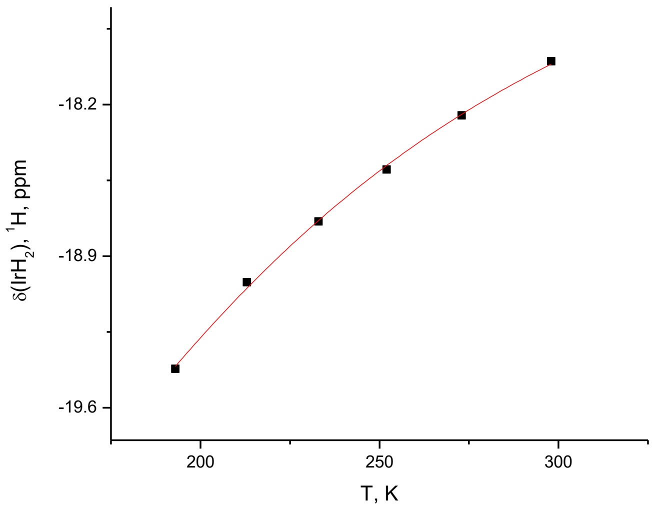

Figure S22. A two-component model (**S** and **NS-bound**) fit of variable temperature NMR spectra of (*p*-H-POCOP)IrH_2 in CD_2Cl_2. ^{1}H chemical shifts are fitted.

Complex (*p*-MeO-POCOP)IrH_2 in CD_2Cl_2:

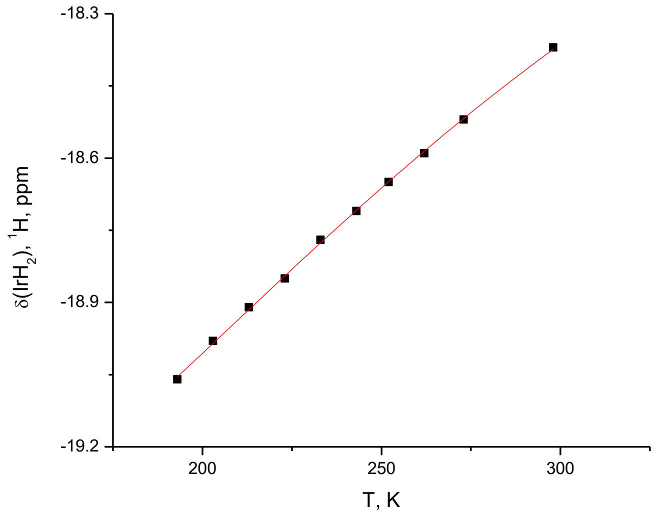

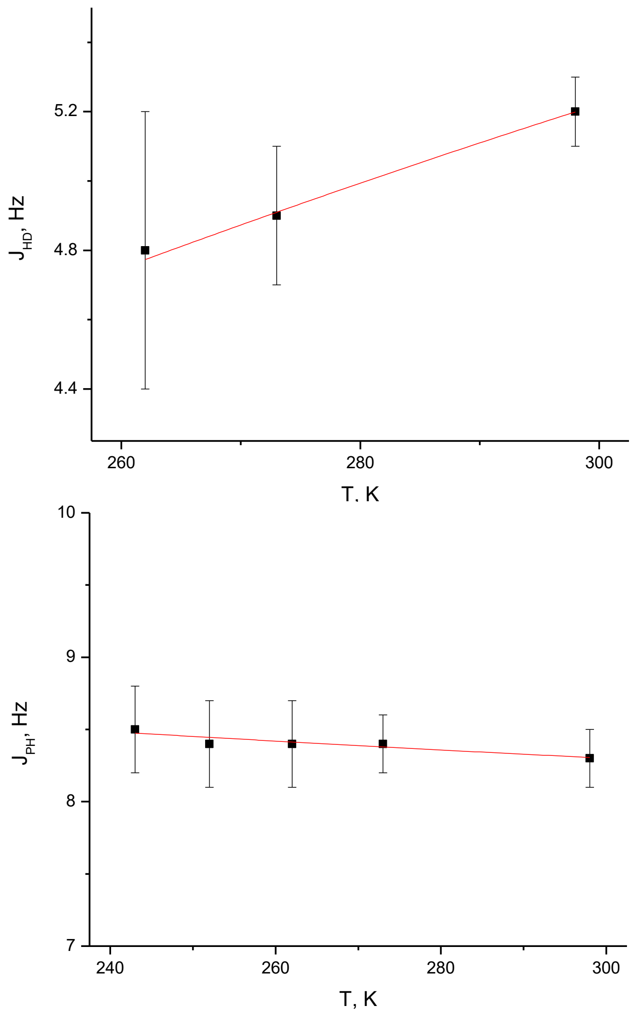

Figure S23. A two-component model (**S** and **NS-bound**) fit of variable temperature NMR spectra of (*p*-MeO-POCOP)IrH_2 in CD_2Cl_2. ^1H chemical shifts, as well as J_{HD} and J_{PH} are fitted.

Table S10. Experimental and calculated parameters of dihydrogen bonding of (X-POCOP)IrH_2 with CH_2Cl_2. Energies versus **S** structures are provided. Note that the thermodynamic corrections to electronic energy E might be somewhat overestimated for a weakly bound adduct, so we provide both ΔE and ΔH. It could be that here ΔE is more appropriate to compare with the experimental values.

	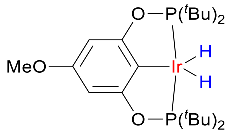	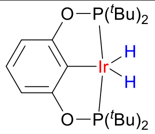	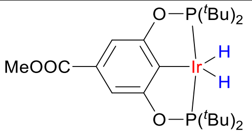	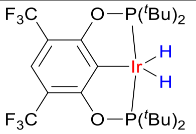	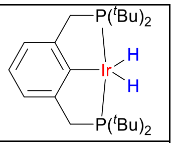
δ_H(**S**)	-16.1	-16.0	-15.1	-15.6	-
δ_H(**NS**)	-19.5	-21.3	-21.5	-22.0	-
J_{HD}, **S**	9.5	-	11.7	9.4	-
J_{HD}, **NS**	3.0	-	-6.3	-3.5	-
J_{PH}, **S**	7.2	-	7.8	8.4	-
J_{PH}, **NS**	8.9	-	12.4	12.3	-
H(exp)-fit	-1.3	-1.2	-1.8	-2.3	-
S(exp)-fit	-2.9	-4.9	-7.1	-9.5	-
E(calc)*	-1.7	-2.3	-1.2**	-3.8	-4.5
H(calc)	-0.1	-0.7	+0.3**	-2.7	-2.9***

*E refers to electronic energies at BS1//D3BJ-revPBE geometries with 2/3-extrapolation//DLPNO-CCSD(T) single point corrections, in kcal/mol. ** Given versus **NS** since calculations suggest **S** is a transition state. *** Due to comparatively low stability of (PCP)IrH_2 in CH_2Cl_2, and thus reduced temperatures span, experimental binding energies were not determined.

8. A control experiment evaluating the effect of relative permittivity on (*p*-H-POCOP)IrH_2

For the measurements, solutions of partially deuterated (*p*-H-POCOP)IrH_2 in CD_2Cl_2 and THF-d_8 under Ar atmosphere were prepared. After NMR recording was complete, the NMR tubes were transferred to a glovebox, where NBu_4PF_6 was added to create solutions with 0.05 M and 0.5 M concentrations of NBu_4PF_6. The NMR tubes were vigorously shaken to dissolve NBu_4PF_6, degassed and refilled with Ar, and then NMR spectra were recorded again. As follows from Table 1 in the manuscript, no changes were detected. In particular, $\Delta\delta$ is very sensitive to environment and can be measured with high precision, however the values with and without NBu_4PF_6 were virtually the same. We thus conclude that changes in relative permittivity do not affect **S-NS** equilibrium to an extent that is suggested by DFT calculations. We ascribe this discrepancy to imperfections of the continuum solvation model used.

As a control, we used a solvatochromic dye, namely Brooker’s merocyanine. As can be seen from Figure S24, addition of NBu_4PF_6 to CH_2Cl_2 solutions clearly caused visually detectable changes.

**C(NBu_4PF_6), M**	0	0.01	0.05	0.1	0.5
**ε**	8.93	9.77	12.4	14.1	24.2

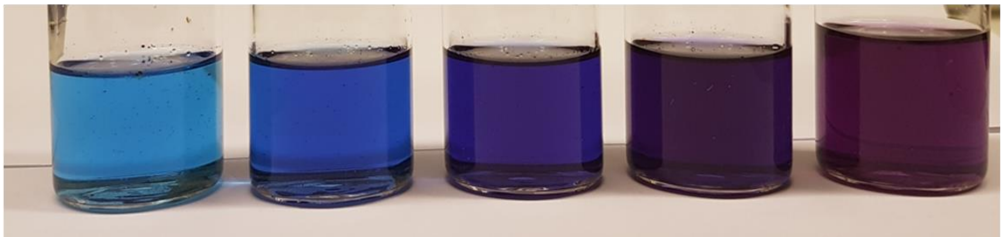

Figure S24. The effect of relative permittivity on Brooker's merocyanine dye solution in CH_2Cl_2 in a control experiment. Upon addition of NBu_4PF_6 the relative permittivity is changed from 8.93 to 24.2,[20] resulting in color change from light-blue to violet. Worth noting is that solution of the dye in acetone with ε=20.7 is also violet.

9. Computational details

9.1 General considerations

DFT calculations were performed with ORCA 4.1.1,[21] using revPBE functional[22] with D3BJ dispersion correction[23,24]m with TightSCF and TightOpt settings. For geometry optimization and frequencies, the def2-SVP basis set[25] was used for C, P, O and H atoms, while def2-TZVP[25] basis set was used for Ir, and ma-def2-TZVP[26] for H atoms in hydride positions (further on this combination is named BS1). The RI algorithm with automatic generation of auxiliary basis sets[27] was used. Solvent effects were incorporated using the CPCM solvation model[28] with toluene and CH_2Cl_2 used as solvents. Grid6 and where necessary GridX6 were used throughout all calculations.

Single-point energies were calculated using DLPNO-CCSD(T)[29] method with def2-TZVP[25] basis set. For a few complexes a 2/3 extrapolation to complete basis set was performed, which indicated that with def2-TZVP a reasonable convergence is achieved. The exceptions were adducts with CH_2Cl_2 and CF_3H; for these compounds, energies are given at 2/3 extrapolation to complete basis set level, instead of def2-TZVP.

We have examined the effect of different basis sets (def2-SVP[25], BS1 [see above], def2-TZVP[25], (SARC)-ZORA-def2-TZVP[30] with ZORA approximation[31], ma-def2-SVP,[26] def2-TZVPD[32]; Figure S26), DFT functionals (PBE0[33], M06L[34], TPSS[35], M06[36], ωB97X-D3BJ[37] as well as DLPNO-SCS-MP2[38]; Figure S29), solvation models (CPCM, SMD, vacuum; Figure S28) and solvents (vacuum, toluene, CH_2Cl_2, EtOH; Figure S27). Overall, virtually all levels of theory are consistent with **S**/**NS** model and provide reasonable convergence of results with respect to geometries and energy gap between **S** and **NS**. The chosen model, BS1//D3BJ-revPBE with def2-TZVP//DLPNO-CCSD(T) single point energies, combines accuracy with a reasonable computational cost, which was near the prohibitive limit for the biggest structures studied (i.e. **NS-bound-c** for *m-bis*-CF_3-POCOP)IrH_2---2×IC_6F_5.

Additional notes regarding the continuum solvation model. On a few occasions we observed in fact very minor, but seemingly relevant discrepancies between computational and experimental data. One of such occasions is the effect of a solvent relative permittivity. Thus, computationally, in CH_2Cl_2 ($\varepsilon = 8.93$) complex (*p*-H-POCOP)IrH_2 exhibits *r*(H-H) of 1.62 and 2.05 Å for **S** and **NS** structures. The electronic energy difference is -0.53 kcal/mol at BS1//D3BJ-revPBE level (-1.13 kcal/mol with DLPNO-CCSD(T) correction), favoring **NS**. When ε was set to 24.2, which corresponds to 0.5 M solution of NBu_4PF_6 in CH_2Cl_2 (ref. 44 in the manuscript), the distances changed to 1.63 and 2.07 Å, respectively, while the energy gap changed to -0.69 kcal/mol at BS1//D3BJ-revPBE (-1.38 kcal/mol with DLPNO-CCSD(T) correction). The difference between δ(IrH_2) and δ(IrHD) in the NMR spectra is very sensitive to the amount of **NS** and can be measured with high accuracy. Thus, we expected that the -0.16 (-0.25) kcal/mol kcal/mol difference, additionally accompanied by structural changes, would be revealed in the NMR spectra, however this was not the case. On the other hand, a solvatochromic dye used as a benchmark clearly demonstrated changes in the control experiment (see section 8). Hence, although Ir-H bonds may have comparatively high polarizability, the changes that are predicted by calculations are clearly bigger than those seen in the experiment.

Other minor deviation is geometry of **S** for (*p*-H-POCOP)IrH_2 (r(H-H) = 1.60 Å BS1//D3BJ-revPBE, 1.62±0.05 Å def2-TZVP//DLPNO-CCSD(T)-corrected, 1.48-1.65 Å for other DFT functionals tested, 1.43(2) Å experimental at 10 K). Remarkably, DLPNO-CCSD(T) does not really cure the distance. Complications with multireference nature of the wavefunction seem unlikely based on "T1 indicator" (see section 9.4). Thus, in view of the above-mentioned evaluation of relative permittivity effect, our guess is that the discrepancies could be due to small solvation or packing effects that are not fully accounted for by a continuum solvation model. There are precedents when such inaccuracies introduce data scattering and cancel out the benefits of using the DLPNO-CCSD(T). As mentioned in ref. 60 (in the manuscript) "…the errors in solvation energy remove much of the advantage in employing more consistently accurate wave function-based methods since they overshadow the improved electronic energies that these methods yield compared to DFT". We attempted to model the environment near Ir atom through placing four $^{t}Bu_2P(OMe)$ groups (with -OMe instead of -O-pincer) near IrH_2 side of the molecule into positions taken from crystallographic studies. Optimization of hydrogens with heavy atoms constrained led to r(H-H) = 1.52 Å, which is closer to the experimental distance. It could be that a better modelling of packing/solvation effects will lead to further improvements. In any case, according to PES scan the energetic difference between the experimental and calculated positions is less than 1 kcal/mol, which makes it challenging to carefully account for all possible reasons of this small deviation.

9.2 Additional computational data

Additional notes on halogen bonding with C_6F_5I. The variety of possible interactions between (X-POCOP)IrH_2 and C_6F_5I is exemplified on Figure 8 in the manuscript. Moreover, **NS-bound-a** can exist as two isomers, **NS-bound-a1** and **NS-bound-a2**. Hydrogen-hydrogen bond lengths and binding energies are listed in Table S11.

Table S11. Binding of C_6F_5I and (X-POCOP)IrH_2 and (PCP)IrH_2. Hydrogen-hydrogen bond lengths and binding energies for **NS-bound-a1** and **NS-bound-a2** isomers.

X =	*p*-MeO-	*p*-H-	*p*-MeOOC	*m-bis*-CF_3-	(PCP)IrH_2
r(H-H) **NS-bound-a1,** Å	2.25	2.36	2.38	2.35	2.45 (tilted)
r(H-H) **NS-bound-a2,** Å	2.01	2.16	2.22	2.19	2.27 (flat)
ΔH **NS-bound-a1,** kcal/mol	-1.8	-3.1	-4.5	-3.8	-5.1
ΔH **NS-bound-a2,** kcal/mol	-3.9	-4.9	-6.7	-6.2	-4.7

As mentioned in the manuscript text, D3BJ-revPBE seemingly over-binds (*p*-MeOOC-POCOP)IrH_2---IC_6F_5 adduct (**NS-bound-a2**) and other halogen-bonded adducts. This is reflected by a shorter $r(H_{eq}\text{-}I)$ distance in calculated structure compared to the experimental one (see Table S12). Also, D3BJ-revPBE binding energy of -12.0 kcal/mol is unreasonably high. Geometry of the adduct is better captured by DLPNO-SCS-MP2 (Table S12). However, this method is too costly for a more detailed study. Fortunately, DLPNO-CCSD(T) correction well cures the interaction energy with D3BJ-revPBE geometry (-7.3 kcal/mol), such as that it is close to DLPNO-SCS-MP2// DLPNO-CCSD(T) energy (-6.5 kcal/mol).

Table S12. Accuracy of some computational methods for predicting geometry of (*p*-MeOOC-POCOP)IrH_2---IC_6F_5 adduct (**NS-bound-a2**)

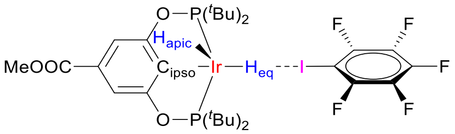

	Neutron diffraction	D3BJ-revPBE	DLPNO-SCS-MP2
r(H-H), Å	2.22	2.22	2.20
$r(H_{eq}\text{-}I)$, Å	2.51	2.27	2.55
$r(H_{apic}\text{-}I)$, Å	3.92	3.67	3.54
$r(H_{eq}\text{-}Ir)$, Å	1.65	1.69	1.66
$r(H_{apic}\text{-}I)$, Å	1.51	1.54	1.52
$r(C_{ipso}\text{-}Ir)$	2.01	2.06	2.05
r(Ir-I)	4.06	3.84	3.95
H_{apic}-Ir-H_{eq}, °	89.0	86.5	87.7
C_{ipso}-Ir-I, °	165.9	170.3	161.9

Additional notes on S/NS equilibrium in CH_2Cl_2.

Table S13. Calculated distances and energies relevant to **S** and **NS** structures in (X-POCOP)IrH_2 complexes in CH_2Cl_2.

X =		*p*-MeO-	*p*-H-	*p*-MeOOC-	*m*-*bis*-CF_3-	(PCP)IrH_2
r(H-H) in **S**, calc., Å	revPBE	1.66	1.62	1.58**	1.59	1.49
r(H-H) in **NS**, calc., Å	revPBE	1.67	2.05	2.13	2.10	2.26
ΔH-calc, kcal/mol	revPBE	+1.6	-0.4	-1.1**	-1.2	-1.1
	revPBE// DLPNO-CCSD(T)*	+1.7 (+2.1)	-1.0 (-1.4)	-2.2** (-3.0)	-2.2 (-2.7)	-1.9 (-2.4)
ΔS-calc, cal×mol^{-1}×K^{-1}	revPBE	-1.1	-2.8	-0.6**	-3.7	-5.5

** The respective electronic energies were obtained using BS1//D3BJ-revPBE geometries with def2-TZVP//DLPNO-CCSD(T) single point corrections. In parenthesis the values corresponding to 2/3-extrapolation//DLPNO-CCSD(T) are given for compatibility with Table S10.** **S** structure appears to be a transition state.

Additional notes on dihydrogen bonding with CH_2Cl_2. Just as it was for C_6F_5I, there are numerous ways of interaction between CH_2Cl_2 and (X-POCOP)IrH_2. Initially, complex (*p*-H-POCOP)IrH_2 was studied, and several isomers were found that are depicted below.

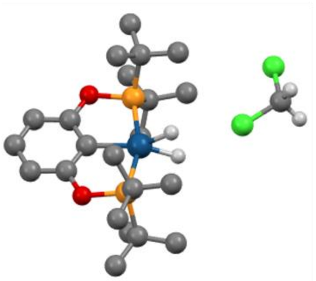
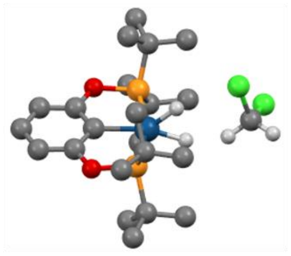
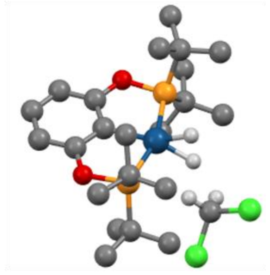
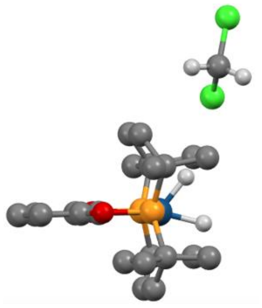

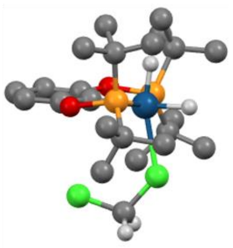
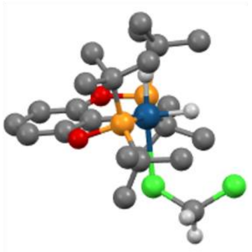
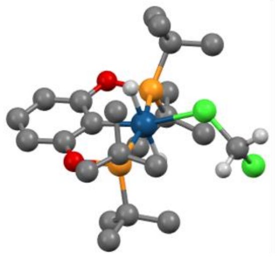

Of those, the lowest-energy isomer with dihydrogen bond as well as the lowest-energy isomer with halogen bond were chosen for a more detailed evaluation. As it was for C_6F_5I, D3BJ-revPBE seemingly over-binds the adducts (thus, for (*p*-MeOOC-POCOP)IrH_2--- CH_2Cl_2 calculated *r*(IrH---H-$CHCl_2$) is 1.84 Å versus ca. 2 Å derived from $T_1(min)$). Other methods such as DLPNO-SCS-MP2 and M06L predict *r*(IrH---H-$CHCl_2$) in a more precise way, for example for M06L the calculated *r*(IrH---H-$CHCl_2$) in (*p*-MeOOC-POCOP)IrH_2--- CH_2Cl_2 is 2.09 Å and *r*(H-H) is 2.08 Å, in a good agreement with the experimental data from $T_1(min)$. However, those methods have other problems, for example DLPNO-SCS-MP2 strongly underestimates **NS** energy, while M06L predicts the non-existent minimum in the dihydrogen complex region (see Figure S28). DLPNO-CCSD(T) corrections are given at 2/3 extrapolation level since in this case the difference between def2-TZVP and 2/3 extrapolation is significant (for example, -4.04 and -2.35 kcal/mol for (*p*-MeOOC-POCOP)IrH_2--- CH_2Cl_2 formation). Table S10, where experimental and computational data are listed, in addition to ΔH provides electronic energies, since the thermodynamic corrections, taken from D3BJ-revPBE, form a considerable fraction of the resulting enthalpies.

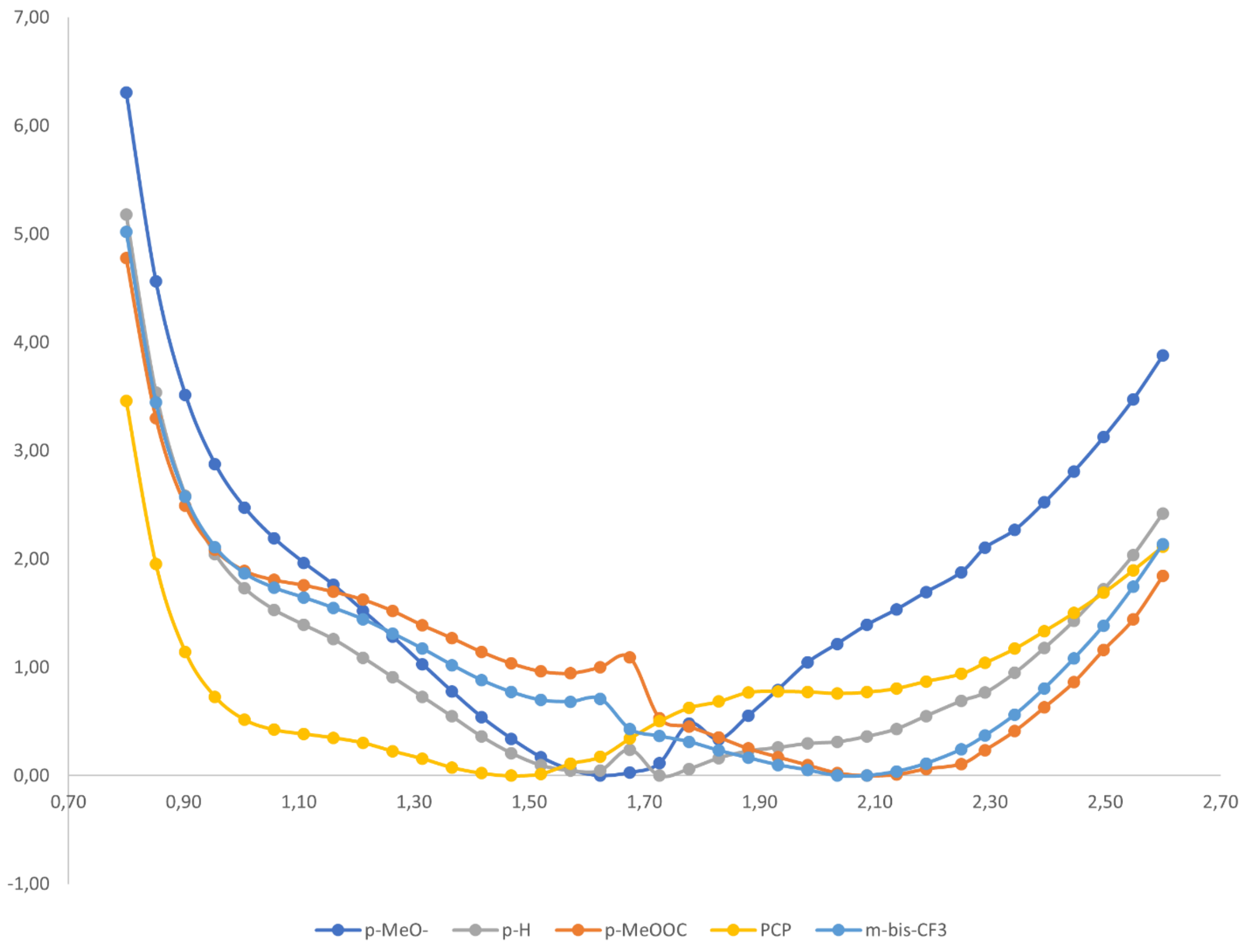

Figure S25. Dependence of potential energy surface in (X-POCOP)IrH_2 on X group, BS1//D3BJ-revPBE, solvent toluene. PES for (PCP)IrH_2 is also shown.

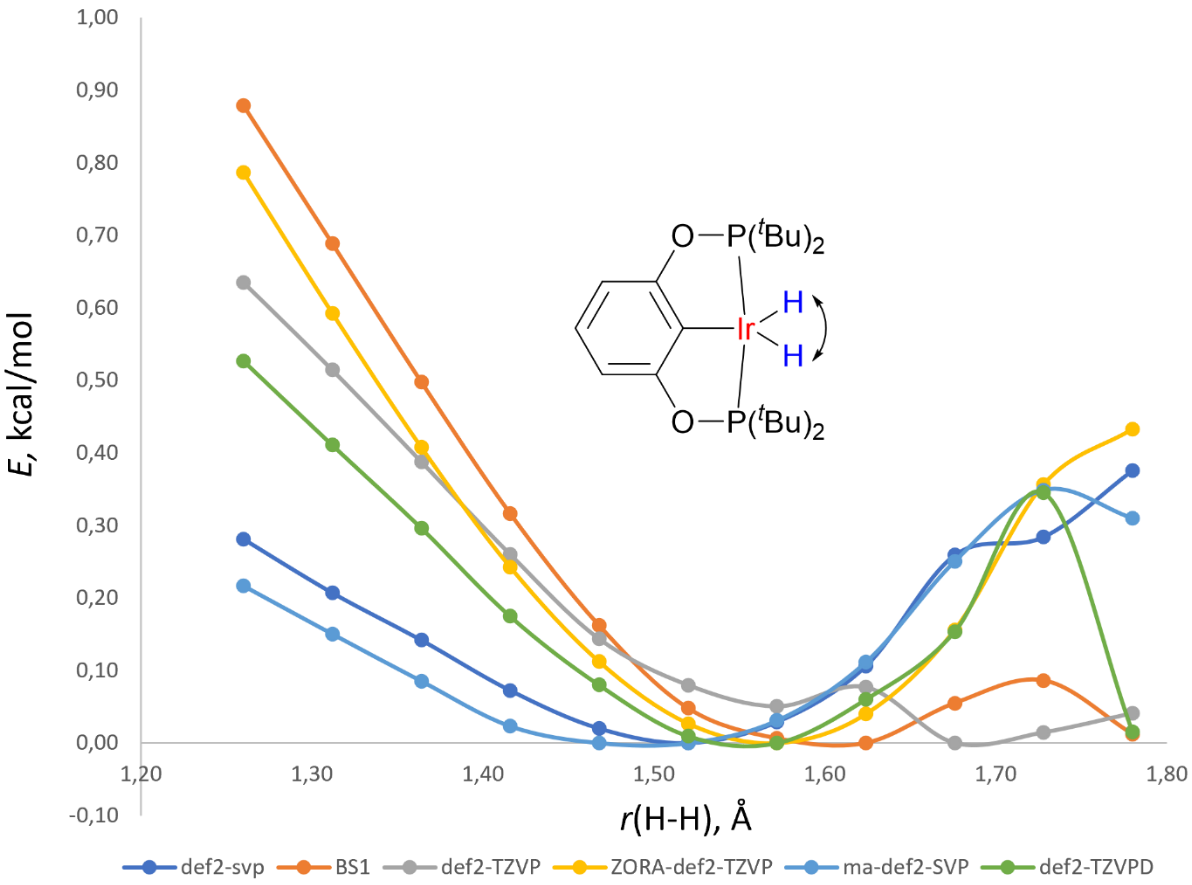

Figure S26. Basis set dependence of potential energy surface in the **S** minimum region for (p-H-POCOP)IrH_2. It can be seen that the chosen BS1 basis set reasonably well approximates larger sets such as def2-TZVPD, being significantly faster at the same time.

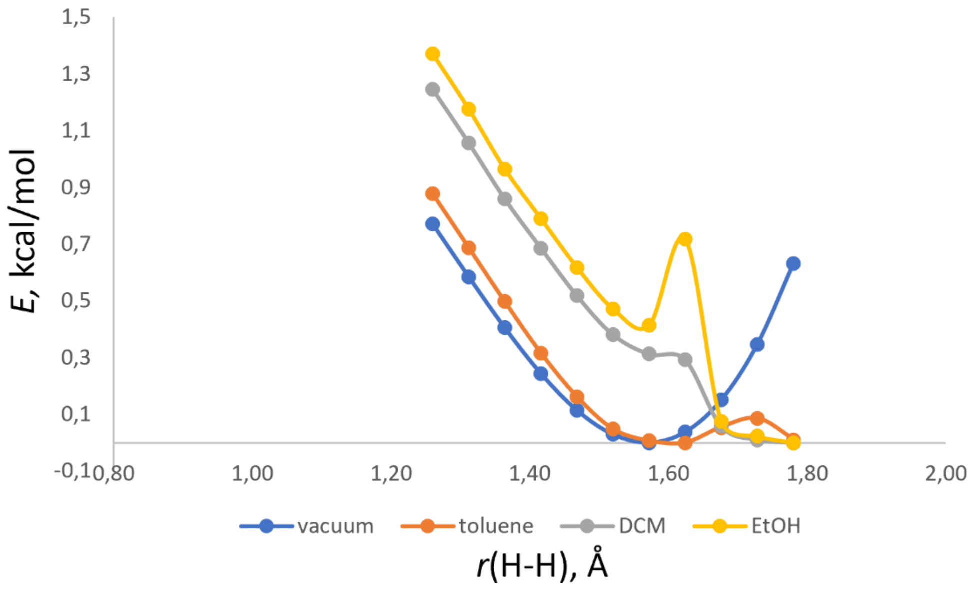

Figure S27. Solvent dependence of potential energy surface in the **S** minimum region for (*p*-H-POCOP)IrH_2. De-symmetrisation of the **S** minimum region upon introducing the solvent effects is clearly observed. BS1 basis set was used.

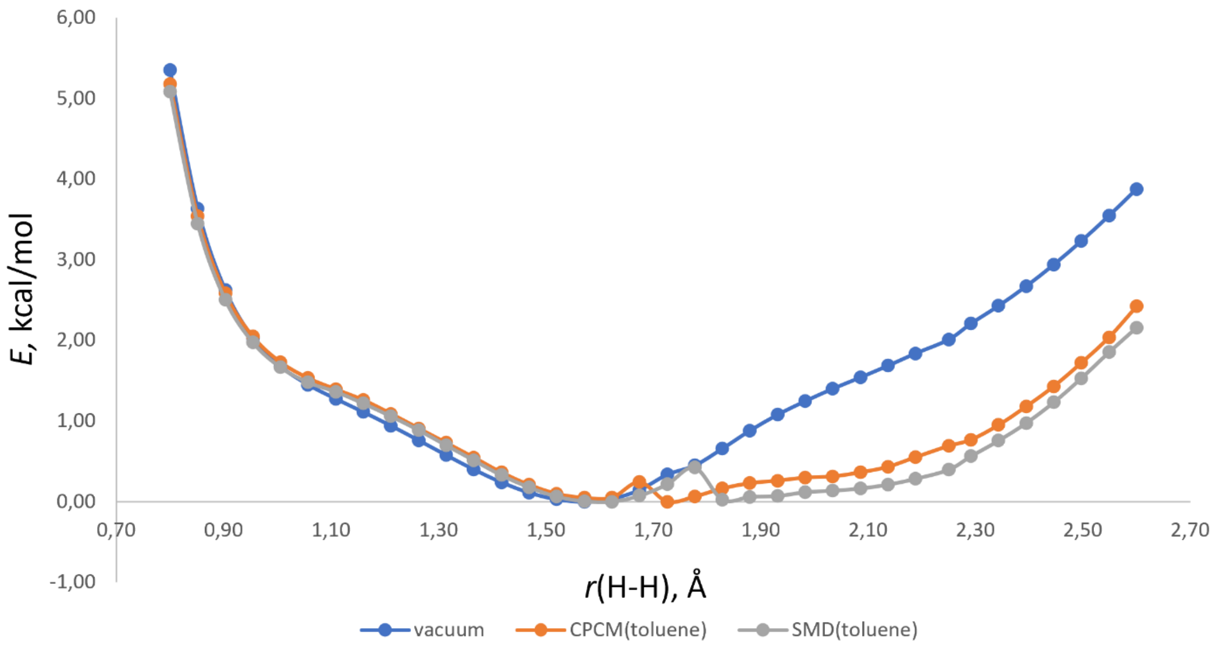

Figure S28. Potential energy surface scan in (*p*-H-POCOP)IrH_2 using CPCM and SMD solvation models at BS1//D3BJ-revPBE level of theory, compared to vacuum.

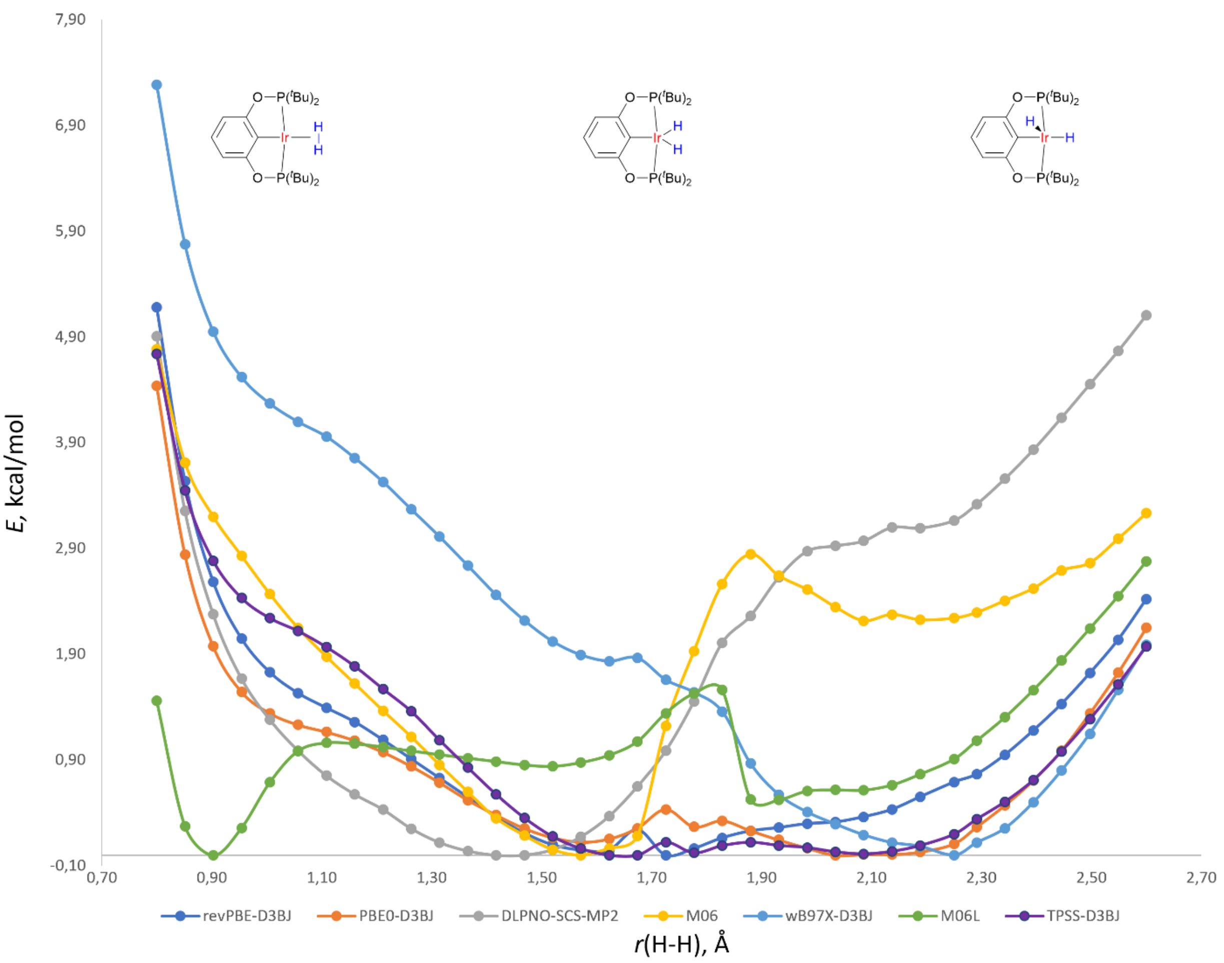

Figure S29. Potential energy surface scan in (p-H-POCOP)IrH_2 using several DFT functionals as well as DLPNO-SCS-MP2 method. BS1 basis set was used.

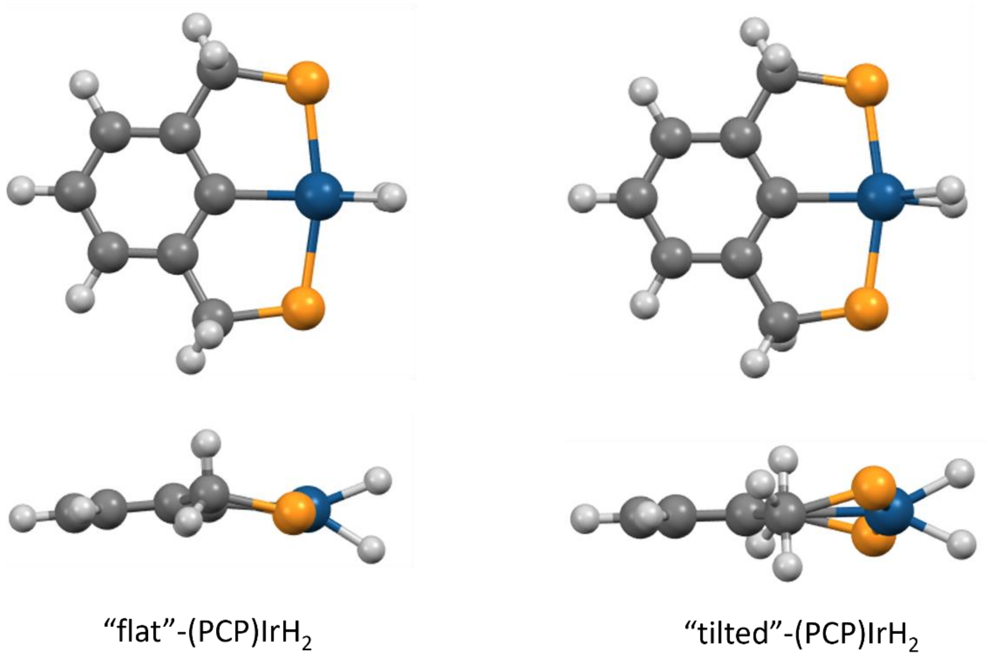

Figure S30. Tilted and flat isomers of (PCP)IrH$_2$. *Tert*-butyl groups are omitted for clarity.

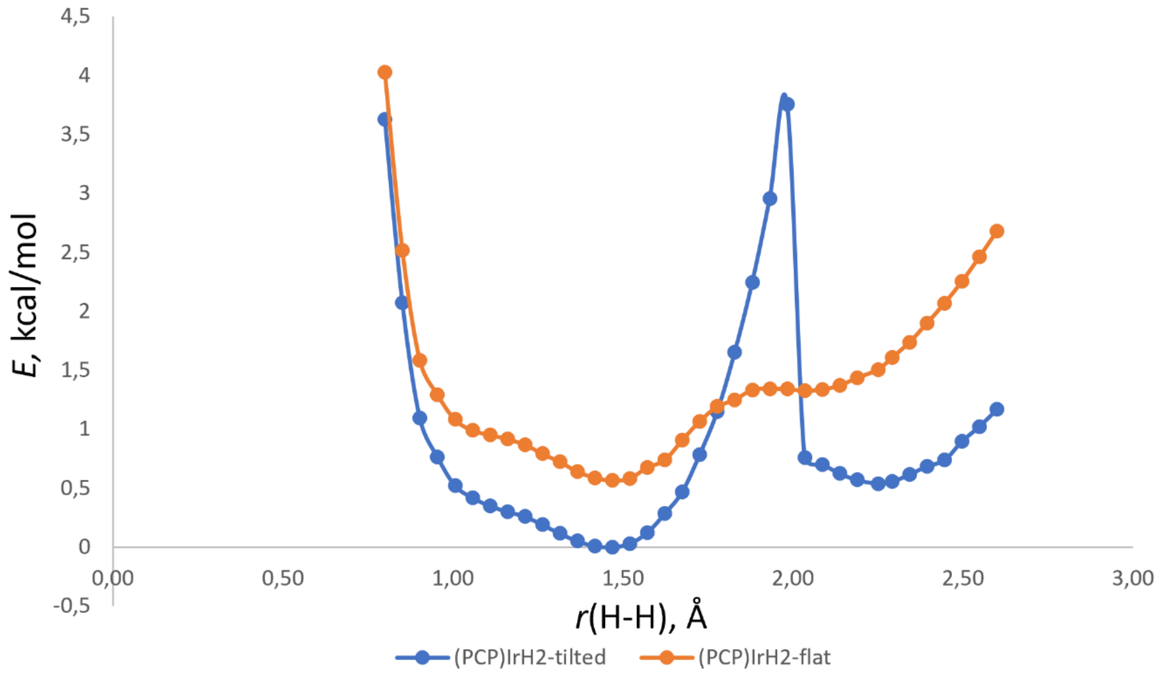

Figure S31. Potential energy surface scan in tilted and flat isomers of (PCP)IrH$_2$ at D3BJ-revPBE level of theory. BS1 basis was used.

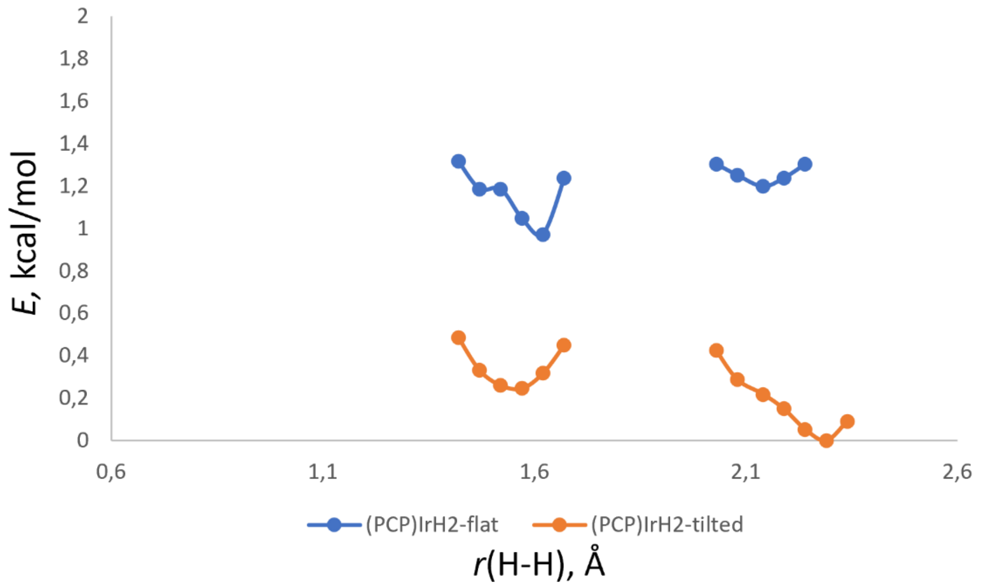

Figure S32. Potential energy surface scan in tilted and flat isomers of (PCP)IrH_2 at D3BJ-revPBE//DLPNO-CCSD(T) level of theory.

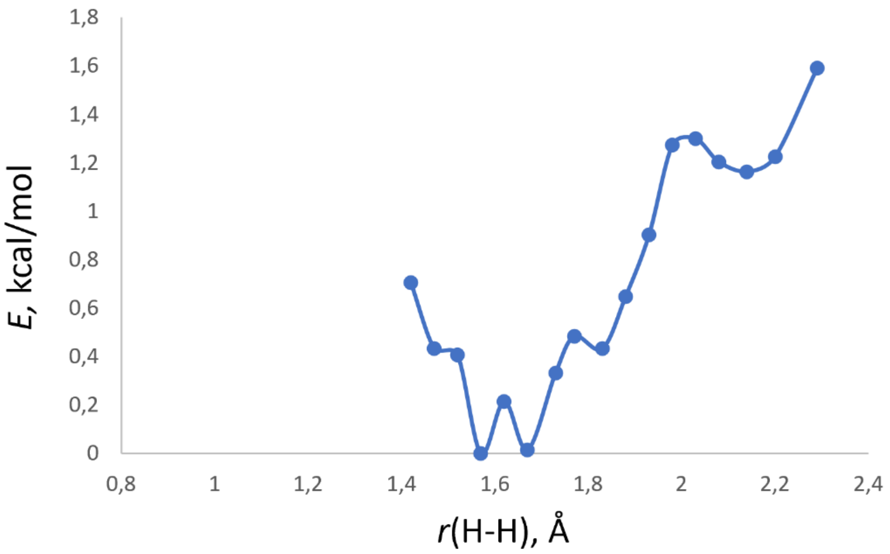

Figure S33. Potential energy surface scan in (*p*-MeO-POCOP)IrH_2 at D3BJ-revPBE//DLPNO-CCSD(T) level of theory.

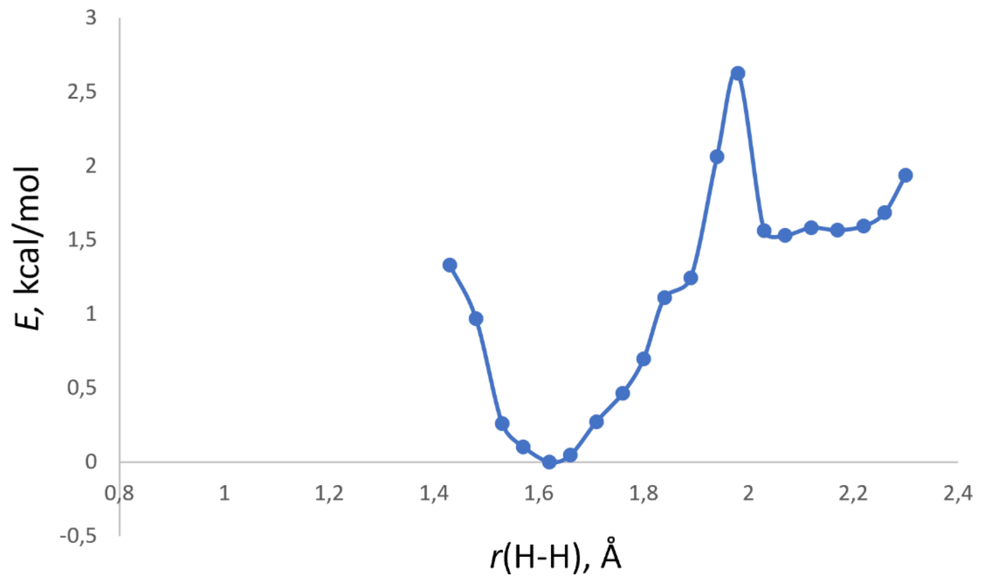

Figure S34. Potential energy surface scan in (*p*-MeO-POCOP)IrH_2 at DLPNO-SCS-MP2//DLPNO-CCSD(T) level of theory.

9.3 Explicit solvation attempts

We performed some attempts to see how explicit solvation could affect PES for (X-POCOP)IrH_2. Although a single molecule of solvent added is not enough for a correct description, it can give an idea about possible interactions between hydrides and a solvent. For that purpose, a molecule of solvent was added in a close proximity to hydrides and H-H distance scan was performed without any constrains for the solvent added. Thus, $C(CH_3)_4$ as a model of alkane solvent did not affect PES to a considerable effect (Figure S35). Benzene, as a model of aromatic solvent, induced some changes in PES (Figure S36). Benzene was found to form adducts with (X-POCOP)IrH_2, such as depicted on Figure S35 where the benzene ring is orthogonal to C_{ipso}-Ir bond, or various “side-on” adducts, with binding nergy of up to 3 kcal/mol. When the solvent with acidic hydrogen was added, modelled with CHF_3, formation of dihydrogen-bonded (X-POCOP)IrH_2---HCF_3 adduct with elongated H-H bond was detected (Figure S37).

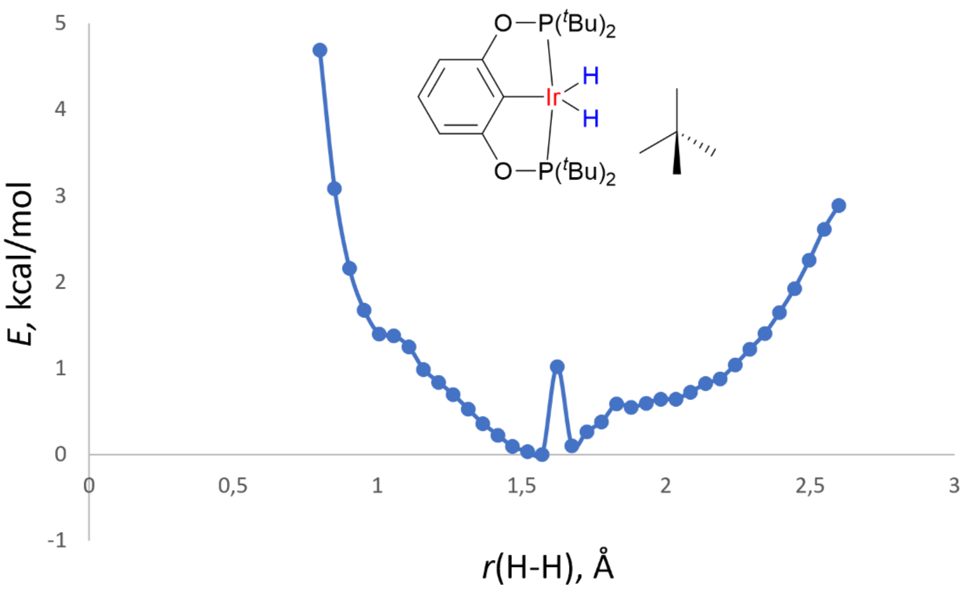

Figure S35. Potential energy surface scan in (*p*-H-POCOP)IrH_2 at D3BJ-revPBE level of theory with explicit molecule of $C(CH_3)_4$ as alkane solvent model added. CPCM solvent toluene. The profile closely resembles the one without $C(CH_3)_4$. Little, in any interaction between solvent and (*p*-H-POCOP)IrH_2 can be noted. **S** and **NS** minima at 1.57±0.05 and 1.67±0.05 are close to 1.60 and 1.63 Å respectively, observed without $C(CH_3)_4$.

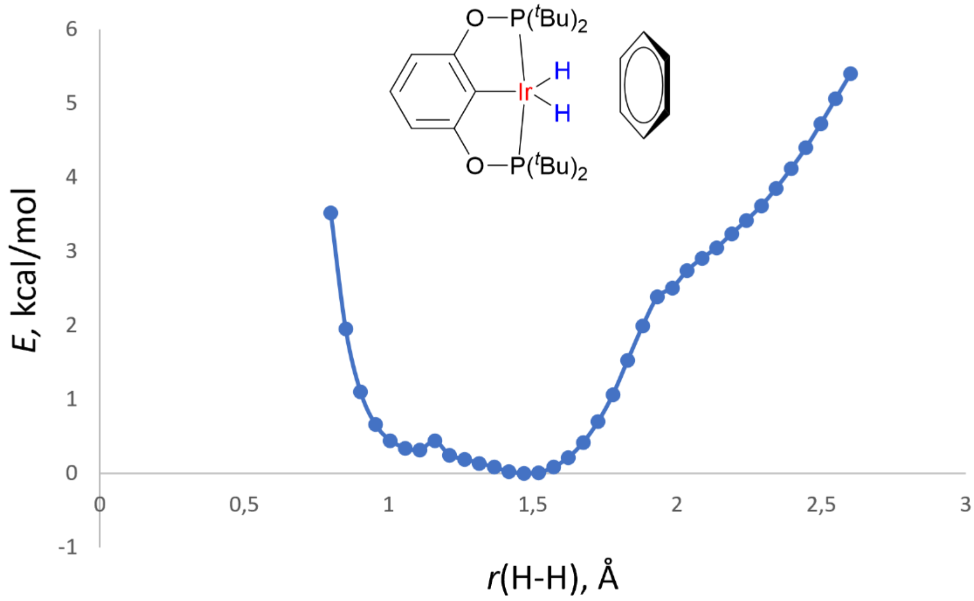

Figure S36. Potential energy surface scan in (*p*-H-POCOP)IrH_2 at D3BJ-revPBE level of theory with explicit molecule of benzene added. CPCM solvent toluene. The profile differs from the one without benzene added. There is an interaction between solvent and (*p*-H-POCOP)IrH_2. **S** minimum is located at 1.47±0.05 Å which is shorter than without C_6H_6 (1.60 Å).

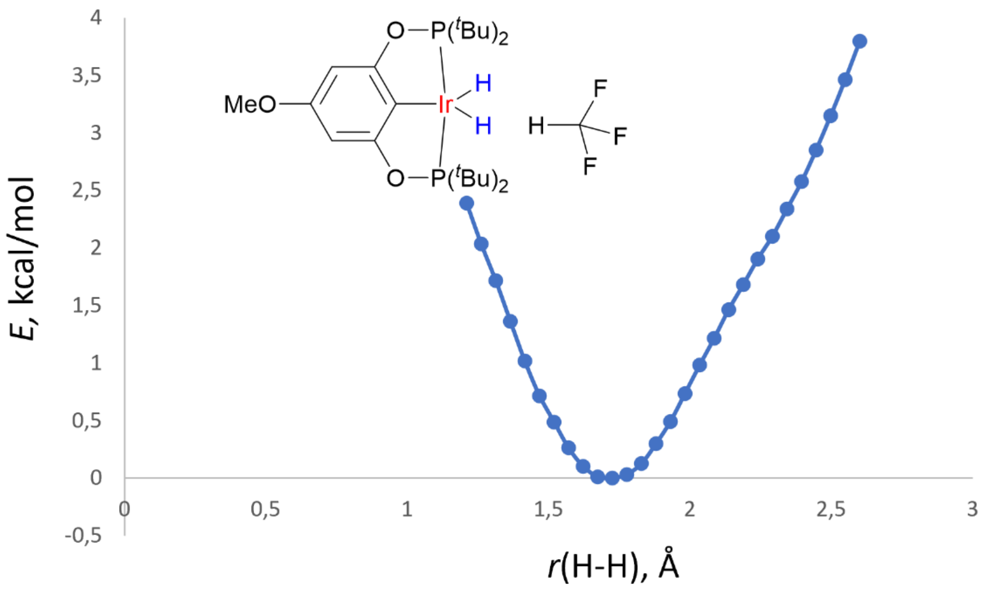

Figure S37. Potential energy surface scan in (*p*-H-POCOP)IrH_2 at D3BJ-revPBE level of theory with explicit molecule of HCF_3 added. CPCM solvent toluene. The profile differs from the one without HCF_3 added. There is an interaction (dihydrogen bond) between HCF_3 and (*p*-H-POCOP)IrH_2. **NS** minimum is located at 1.72±0.05 Å which is longer than without HCF_3 (1.60 Å).

9.4 Examining the possible multireference nature of (X-POCOP)IrH_2 and (PCP)IrH_2

So-called T1-diagnostic (shall not be confused with NMR-related T_1(min) used elsewhere in the manuscript) has been proposed as a multireference indicator when it comes to coupled cluster methods, such as DLPNO-CCSD(T). The value smaller than 0.05 is believed to be reliable for transition metal complexes[39]; a stricter threshold of 0.02 is suggested in ORCA manual. As is depicted on Figures S38-39, at all PES points T1-diagnostic for (*p*-H-POCOP)IrH_2 and (PCP)IrH_2 is significantly lower than 0.02. Therefore, multireference character of the systems studied seems unlikely, although cannot be completely excluded.

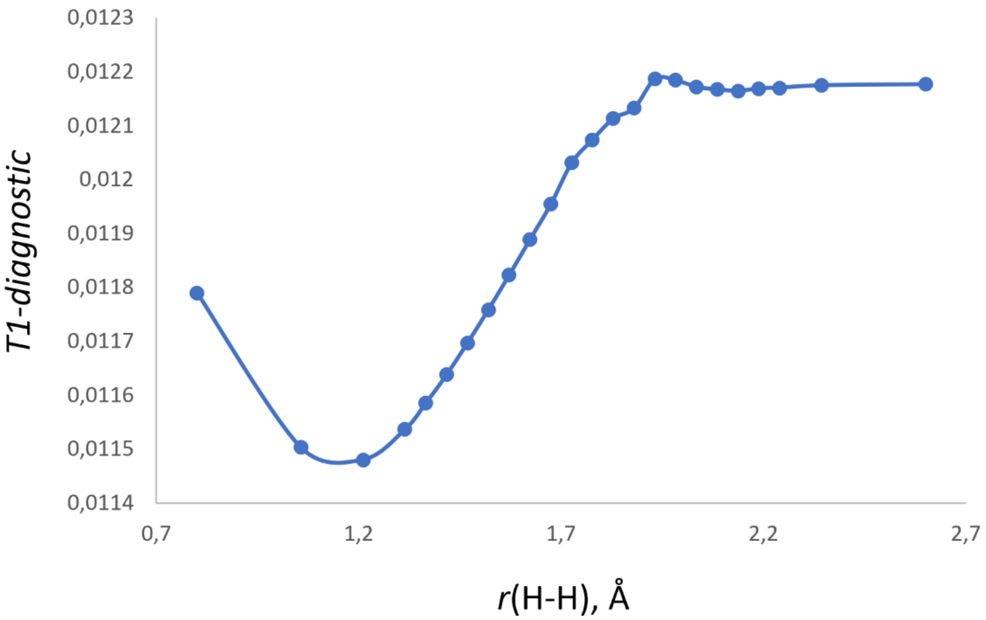

Figure S38. T1-diagnostic versus *r*(H-H) in (*p*-H-POCOP)IrH_2, calculated at D3BJ-revPBE//DLPNO-CCSD(T) level of theory.

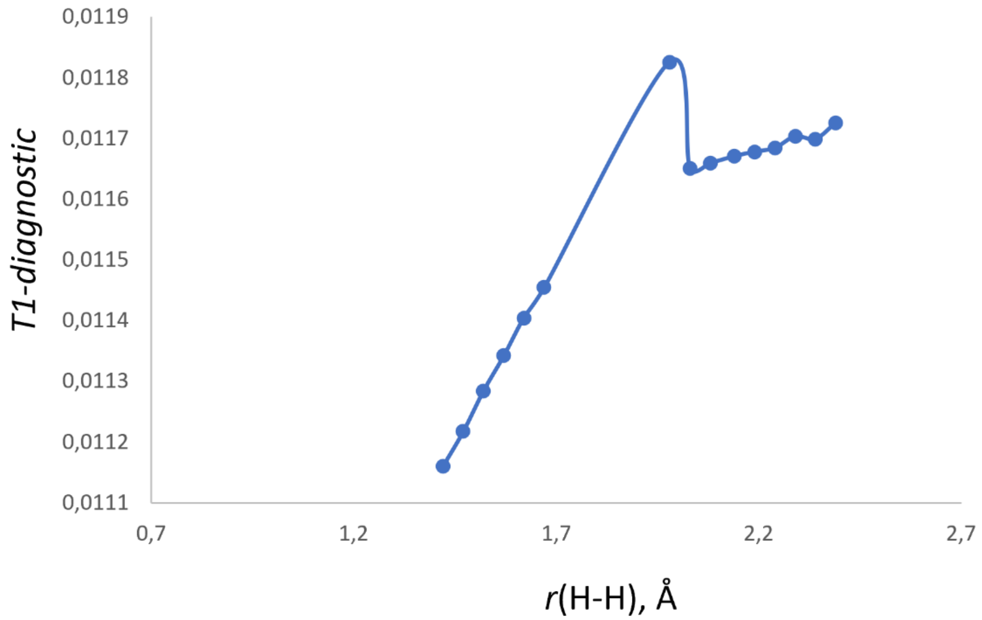

Figure S39. T1-diagnostic versus *r*(H-H) in (PCP)IrH_2, calculated at D3BJ-revPBE//DLPNO-CCSD(T) level of theory.

10. Calculation of NMR chemical shifts and coupling constants

To correctly evaluate ^{1}H chemical shifts in transition metal hydrides, fully relativistic four-component DFT calculations of NMR properties are required. Table S14 lists a dataset we used to evaluate the performance of ReSpect program for some POCOP iridium pincer complexes. It can be seen that the most high-field ^{1}H chemical shifts below -25-30 ppm are systematically underestimated, in agreement with what some of the ReSpect authors report in ref. 34 (in the manuscript). To improve agreement with the experimental data, here we propose an empirical correction of $\delta = 1.091\times\delta_{raw}-0.726$ applied for all calculated hydride chemical shifts (Figure S40). The correction coefficients are based on a simple linear regression of the dataset in ref. 43 in the manuscript, and also improves the agreement for dataset used in Table S14. Through the correction the agreement between experimental and calculated ^{1}H chemical shifts is brought to ca. 2 ppm. We also note that the calculated J_{HD} and J_{PH} are systematically smaller than the experimental values, but since larger datasets are not available, here we used the coupling constants without any corrections.

Table S14. Data set to evaluate the performance of ReSpect for iridium pincer complexes.

**Complex**	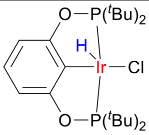	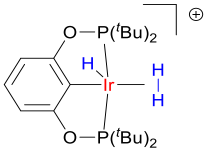	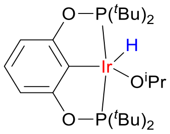	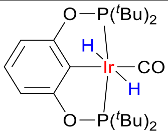
δ ^{1}H, ppm, calc	-34.48	-35.64; 0.71	-28.67	-7.63
δ ^{1}H, ppm, calc corrected	-38.34	-39.61; 0.05	-32.00	-9.05
δ ^{1}H ppm, exp	-40.66[40]	-41.9; 0.3[41]	-34.2[42]	-9.55[43]
J_{HD}, Hz, calc	-	27.1	-	-
J_{HD}, Hz, exp	-	33	-	-
δ ^{31}P, ppm, calc	198.9	-	195.8	198.4
δ ^{31}P, ppm, exp	175.5	-	171	183.4
^2J(P-H), Hz, calc	7.8	-	-	11.2
^2J(P-H), Hz, exp	13.1	-	-	15.2

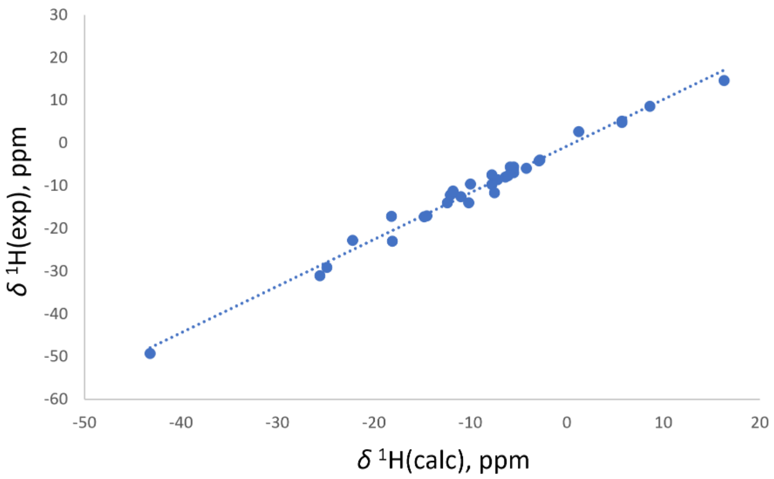

Figure S40. A linear regression of calculated and experimental ^{1}H NMR chemicals shifts in transition metal hydrides using the data from ref. 34 (in the manuscript).

The NMR calculations were done using PBE functional,[44] mdks method as implemented in ReSpect, Dyall's DZ[45] basis set for Ir and IGLO-II[46] basis set for the rest of the atoms. ^{1}H NMR chemical shifts are referred to TMS at the same level of theory, ^{31}P NMR chemical shifts are referred to (POCOP)IrHCl at the same level of theory.

11. Re-evaluation of complex Cp*Ru(dppm)H_2^+

11.1 Re-evaluation of complex Cp*Ru(dppm)H_2^+. Optimization was started from the neutron diffraction structure with CH_2Cl_2 set as solvent. It converged to a structure with a small imaginary frequency that disappeared after the two in-plane Ph groups were slightly tilted. Using this geometry, a PES scan was performed as given on Figure S41. Subsequent optimization converged to two minima at 1.06 and 1.42 Å, respectively. A reasonable fit of J_{HD}-T dependence (Figure S42, data from ref. 47) can be constructed using limiting J_{HD} values obtained from the above-mentioned distances using the known correlation.[48]

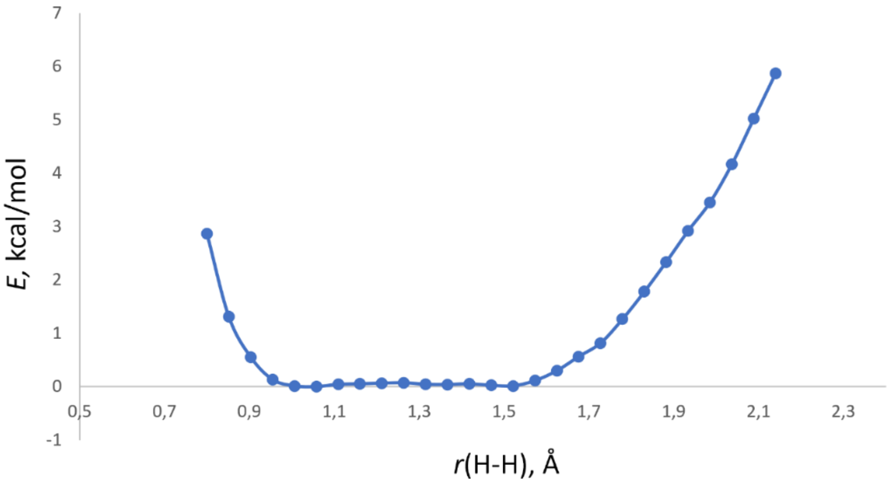

Figure S41. Potential energy surface scan in Cp*Ru(dppm)H_2^+ using BS1//D3BJ-revPBE level of theory and CH_2Cl_2 as solvent.

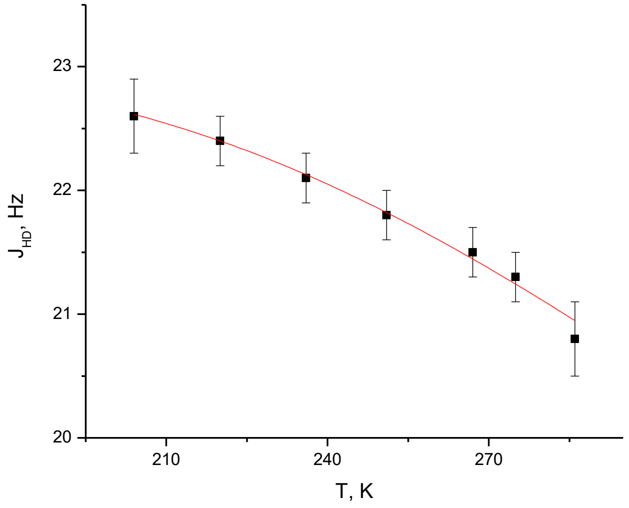

Figure S42. A possible fit of J_{HD}-T dependence in Cp*Ru(dppm)H_2^+ using data from ref. 47. Coupling constants were set to 23 and 9.2 Hz. The fitted ΔH and ΔS are -2.6 kcal/mol and -5.4 cal×mol^{-1}×K^{-1}. For comparison, the ΔH and ΔS values for a slow compared to the NMR timescale isomerization of CpRu(dmdppe)$(H_2)^+$↔ CpRu(dmdppe)$(H)_2^+$ are -0.9 kcal/mol and -4.5 cal×mol^{-1}×K^{-1} (ref. 53 in the manuscript).

The fitted ΔH is slightly lower than the one that could be expected from the PES scan. That is a satisfactory agreement between computational and experimental data, given the possible errors of implicit solvation model for cationic species (see ref. 60 in the manuscript).

Figure S43 compares different models for Cp*Ru(dppm)H_2^+, starting from the truncated model (**a**) as used in the previous studies. Unfortunately, it seems that this molecule is not very forgiving to the approximations made, and only with the highest quality models a reasonable agreement with the experimental data can be achieved.

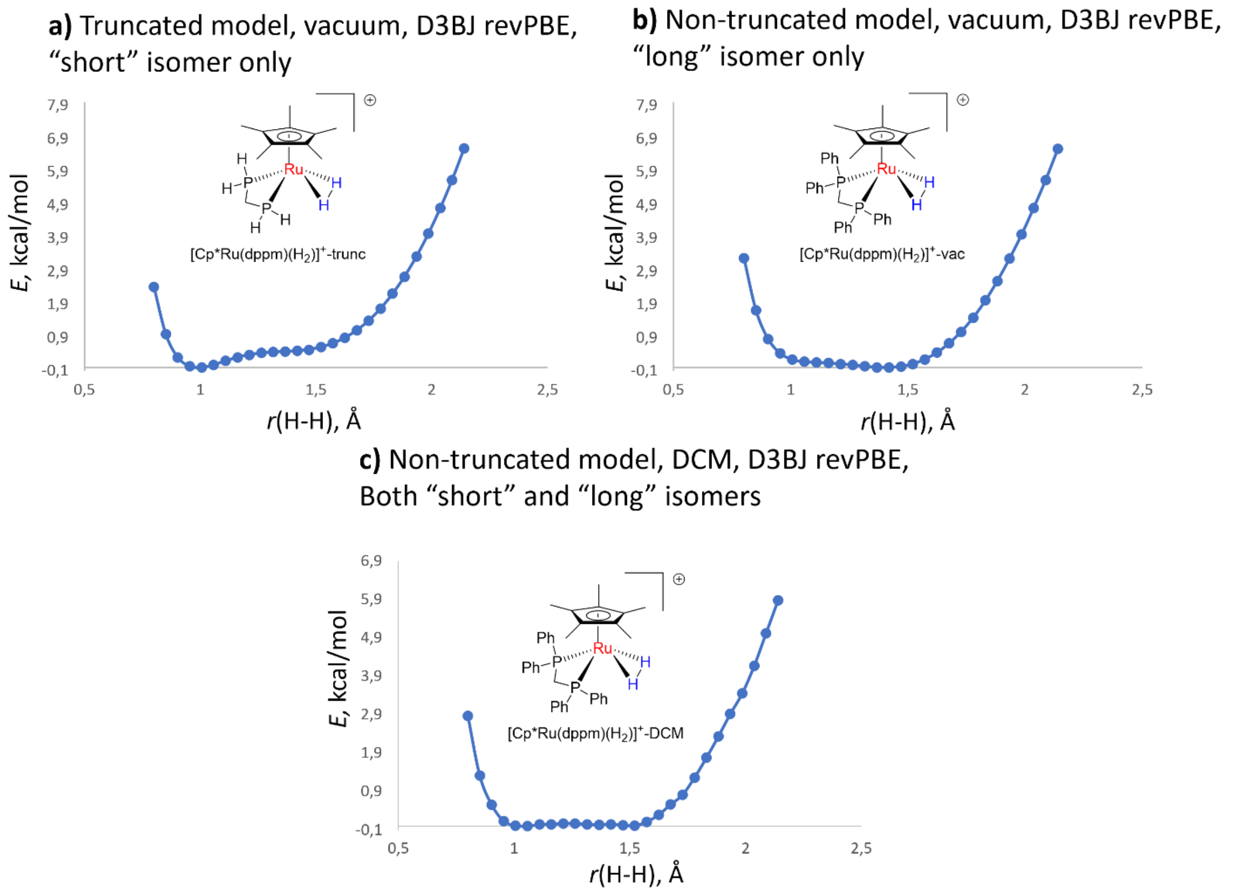

Figure S43. Comparison of different computational models for Cp*Ru(dppm)H_2^+.

We tested the computational model (c) for stability in manner similar to the one used for (X-POCOP)IrH_2. Thus, variation of the solvent (CH_2Cl_2, MeOH, water, DMSO) was examined and produced comparatively small changes in *r*(H-H) and relative energies. In all solvents the two minima were successfully observed, with "longer" isomer slightly favored in non-polar solvents. Also, change of CPCM for SMD solvation models was tested and did not induce any significant changes in geometry or relative energies. Two other DFT functionals in addition to the default D3BJ-revPBE were tried, as well as single-point DLPNO-CCSD(T) corrections. Thus, with D3BJ-TPSS the two isomers were found with *r*(H-H) of 0.97 and 1.50 Å. With D3BJ-PBE0, only the "short" isomer at 0.98 Å was observed, and a shoulder instead of a "long" isomer. The latter could be detected at ca. 1.35 Å as a shallow minimum when a bigger basis set (def2-TZVPD) was used

for a single-point correction. Perhaps most importantly, the two isomers were present when DLPNO-CCSD(T) corrections were applied (Figure S44). Thus, although the relative energies may have limited precision for a cationic compound with the implicit solvation model, majority of the methods used were consistent with the existence of the two isomers of $Cp*Ru(dppm)H_2^+$ in solution.

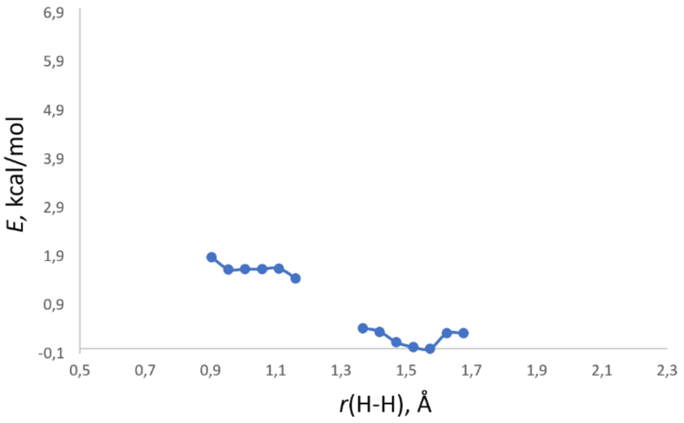

Figure S44. Potential energy surface scan in $Cp*Ru(dppm)H_2^+$ with DLPNO-CCSD(T) single-point energy corrections and CH_2Cl_2 as solvent.

11.2 Explicit solvation by one CH_2Cl_2 molecule. Further on, we examined the possible effects of explicit solvation with CH_2Cl_2. Two binding modes were explored, as exemplified below for the "longer" isomer. Firstly, we have considered dihydrogen bond Ru-H---H-$CHCl_2$. It was found that, although CH_2Cl_2 can reside in the cavity formed by the two Ph rings in front of the hydride ligands, the closest Ru-H---$HCHCl_2$ distance observed in the CH_2Cl_2 adduct was 2.5 Å, which is much longer than the distances observed for Ir compounds (1.84-2.04 Å for dihydrogen bond Ir-H---H-$CHCl_2$) and is slightly longer the respective sum of van der Waals radii (2.4 Å). Loewdin charges on Ir (-1.42 vs. -1.44) and the hydrides (0.07 and 0.08 vs. 0.06 and 0.07) were almost unaffected by the presence of CH_2Cl_2. Thus, CH_2Cl_2 did not reveal bonding interaction with hydrides. At the same time, CH_2Cl_2 was bound to the π-electron cloud of one the Ph rings through Ph---H-$CHCl_2$ interaction and to the >P-CH_2-P< fragment via -CH-H---Cl-CH_2Cl hydrogen bond. The lack of dihydrogen bond for $[Cp*Ru(dppm)(H_2)]^+$ probably reflects weaker "hyrdicity" of this complex, which is reasonable given the formal positive charge on it. Secondly, we examined the bonding with the Cl site of CH_2Cl_2. Given the formal positive charge on the complex, one would expect that a possible Ru-H---Cl-CH_2Cl interaction is a hydrogen bond rather than halogen bond. The 3.0 Å Ru-H---Cl-CH_2Cl distance was again slightly longer the sum of van der Waals radii (2.95 Å), meaning that hydrogen bond is non-existing or very weak. Bonding of CH_2Cl_2 was again through Ph---H-$CHCl_2$ and -CH-H---Cl-CH_2Cl interactions. The CH_2Cl_2 enthalpy of binding is 5 kcal/mol, given at 2/3-extrapolation//DLPNO-CCSD(T) level. Approximately half of it comes from Ph---H-$CHCl_2$ interaction (thus, formation of C_6H_6---H-$CHCl_2$ adduct has ΔH of -2.6 kcal/mol at the same level of theory), and the rest from -CH-H---Cl-CH_2Cl interaction. The distances between the hydrides are almost unchanged (1.41 and 1.45 Å vs. 1.42 in the $Cp*Ru(dppm)H_2^+$).

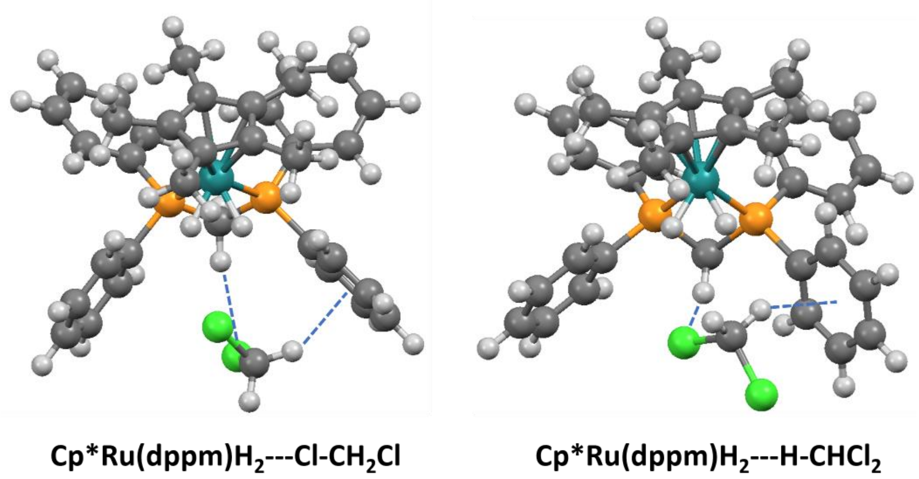

Figure S45. Interactions of complex Cp*Ru(dppm)H_2^+ ("longer" isomer) with the explicit CH_2Cl_2 molecule. The distances between hydrides and CH_2Cl_2, Ru-H---H-$CHCl_2$ of 2.5 Å and Ru-H---Cl-CH_2Cl of 3.0 Å are in the non-bonding range. The CH_2Cl_2 molecule is bound to the Ru complex through interaction with the π-electron cloud of one of the Ph rings and hydrogen bond with >P-CH_2-P< fragment (shown with dashed lines).

Virtually the same picture was observed for the "short" isomer. *r*(H-H) in the "short" isomer was slightly elongated upon coordination of CH_2Cl_2 (1.17 vs 1.06 Å in the Cp*Ru(dppm)H_2^+). Such elongation was deemed insignificant due to the exceptionally flat PES in the region of interest (see Figures S41 and S46), and was not reproduced with methods that give less shallow "short" minimum such as D3BJ-PBE0. PES scan with explicit CH_2Cl_2 did not reveal any significant energy changes between the "short" and "long" isomers (Figure S46, compare with Figure S41).

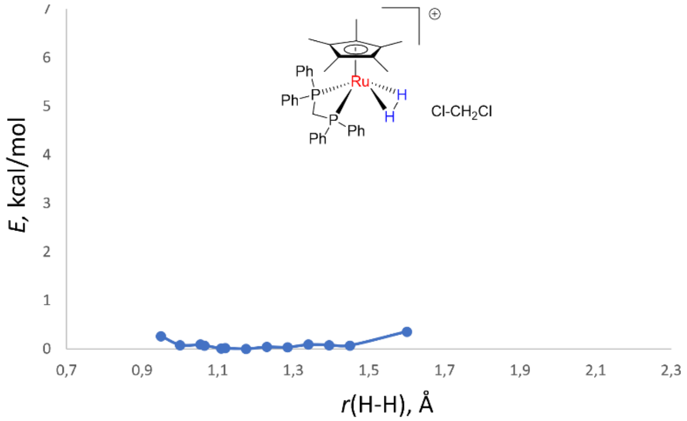

Figure S46. Potential energy surface scan in Cp*Ru(dppm)H_2^+ at D3BJ-revPBE level of theory with explicit molecule of CH_2Cl_2 added. CPCM solvent CH_2Cl_2.

We thus conclude that although an explicit CH_2Cl_2 can reveal attractive interactions with Cp*Ru(dppm)H_2^+, the hydride sites do not participate in bonding, and the equilibrium between the "short" and "long" isomers is not strongly perturbed by the inclusion of the explicit CH_2Cl_2.

11.3 Possible ion pairing effects. Finally, we explored possible effects of ion pairing. Judging from the experimental data, it is unlikely that ion pairing effects are involved. Firstly, the change of a counter-anion does not affect the NMR spectra of $[Cp^*Ru(dppm)(H_2)]^+$. Thus, there are two sets of experimental data reported for $[Cp^*Ru(dppm)(H_2)]^+$. Both were measured in CD_2Cl_2, one was recorded with BF_4^- counter-anion (ref. 10 in the manuscript), another was recorded with $B(ArF)_4^-$ counter-anion (ref 16b in the manuscript). The two datasets coincide (within the errors of measurement), which argues against significant effects of ion pairing on the NMR spectra. Secondly, unlike several other cases mentioned in the manuscript, the reported neutron diffraction structure of $[Cp^*Ru(dppm)(H_2)]^+$ that contains BF_4^- counter-anion, does not reveal close interactions between RuH_2 and BF_4^-. Thus, Ru-H---F-BF_3 distance of 3.38 Å in $[Cp^*Ru(dppm)(H_2)]^+$ clearly is in the non-bonding region. For comparison, the closest M-H---F-BF_3 contacts are 2.74 Å in $Cp^*OsH(H_2)H(PCy_3)^+BF_4^-$ (ref. 56 in the manuscript) and 2.42 Å in $Cp^*OsH(H_2)H(PPh_3)^+BF_4^-$[49]. Instead, as one would expect after studying interactions with CH_2Cl_2, in the solid state BF_4^- reveals close interaction with >P-CH_2-P< fragment (F---H-CH- 2.28 Å). The binding of BF_4^- to >P-CH_2-P< instead of hydrides explains the lack of counter-anion effect on $[Cp^*Ru(dppm)(H_2)]^+$. A good example of an opposite case, that is how replacement of PF_6^- counter-anion that interacts with hydrides, with a less nucleophilic BPh_4^- counter-anion, could affect the distance between hydrides, is given in ref. 59 (in the manuscript).

Computational study supported the conclusions made above. Thus, coordination of BF_4^- to >P-CH_2-P< site (Figure S47, structures d-f) was found slightly more favorable compared to "bifurcated" coordination to both RuH_2 and >P-CH_2-P< fragments (Figure S47, structures a-c). Exclusive coordination to RuH_2 was not observed. When RuH_2 fragment was involved in bonding, only "short" isomers were observed that revealed comparatively minor shortening of *r*(H-H) compared to the parent $[Cp^*Ru(dppm)(H_2)]^+$. For isomers that reveal coordination to >P-CH_2-P<, a minor elongation of *r*(H-H) was observed, and both "short" and "long" isomers with comparable energies were present. Given that the lowest energy isomer does not involve interaction with RuH_2 unit, and that the *r*(H-H) and relative energy changes invoked by BF_4^- coordination are minor, it is likely that ion pairing has little, if any, effect on the NMR spectra of $[Cp^*Ru(dppm)(H_2)]^+$. When a more complex model involving partial explicit solvation with CH_2Cl_2 was attempted, the isomers with coordination of BF_4^- to >P-CH_2-P< site were even more favored, with the difference reaching 4 kcal/mol (Figure S48). Thus, when three molecules of CH_2Cl_2 were added to structures (a), (b) and (d), new structures (a)×3CH_2Cl_2, (c)×3CH_2Cl_2, and (d)×3CH_2Cl_2 were obtained, as depicted on Figure S48. These structures feature weaker RuH_2---BF_4^- interaction, reflected for example by a longer *r*(H---F) of 2.34 Å in (a)×3CH_2Cl_2 compared to 2.21 in (a)), and spontaneous isomerization of (b)×3CH_2Cl_2 to "longer" isomer of (c)×3CH_2Cl_2. PES scan again exhibited almost the same energies for "short" and "long" isomers of (d)×3CH_2Cl_2 and (c)×3CH_2Cl_2 and we have not further pursued optimizing more structures since it was very time consuming due to wavefunction oscillations. To sum up, ion pairing with and without explicit solvent involves mainly interaction with >P-CH_2-P< fragment and has little effect on equilibria between the "short" and "long" isomers – fully in line with the experimental datasets.

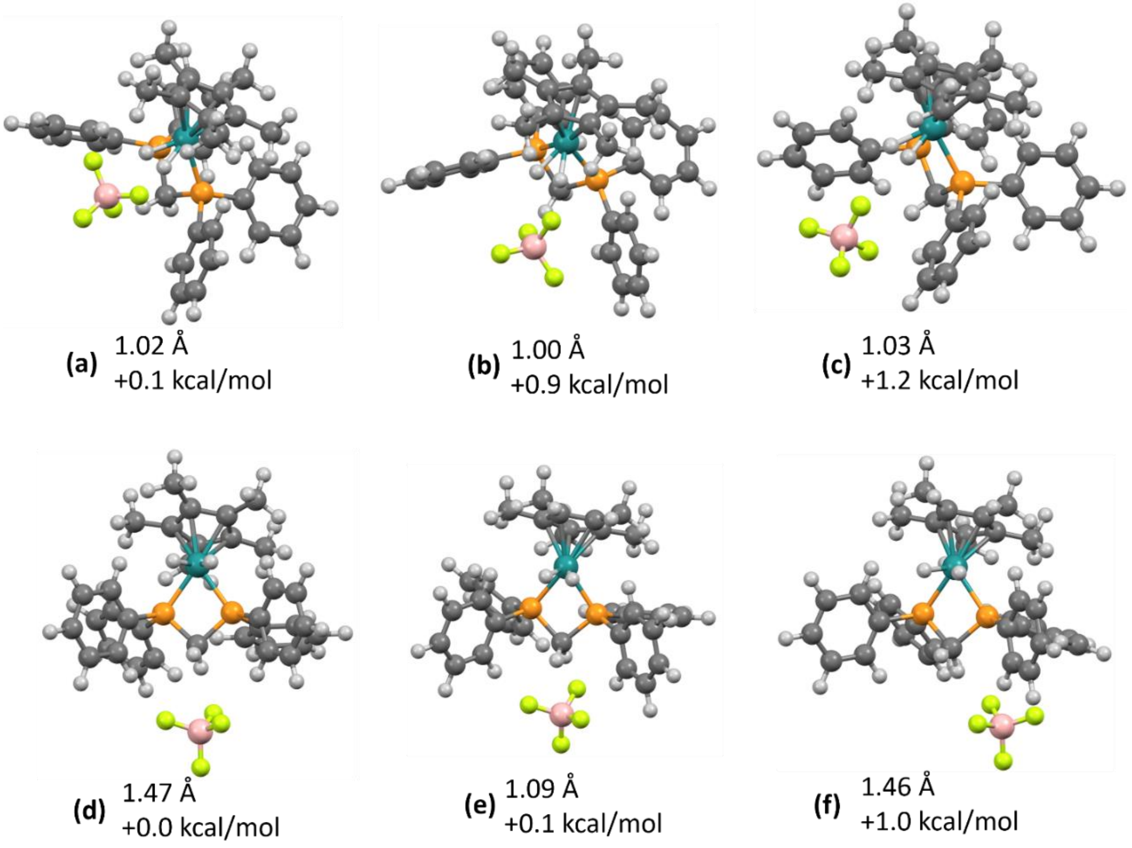

Figure S47. Isomers of $Cp^{*}Ru(dppm)H_2^{+}$ BF_4^{-} ion pair at D3BJ-revPBE level of theory. CPCM solvent CH_2Cl_2. Structures a-c feature interaction of BF_4^{-} with both RuH_2 and >P-CH_2-P< fragments. Structures d-f feature interaction of BF_4^{-} with >P-CH_2-P< fragment only. *r*(H-H) and relative energies in kcal/mol are provided.

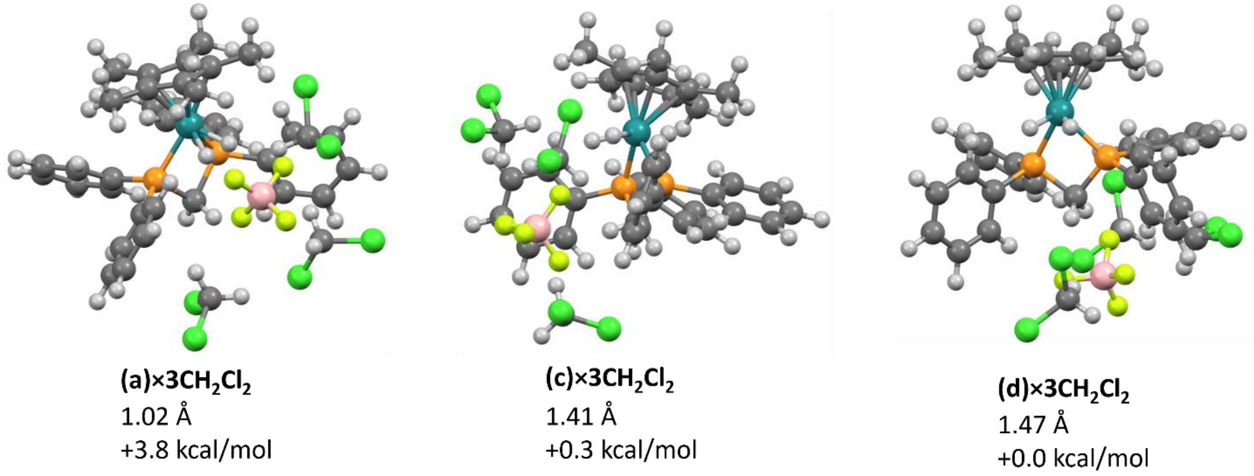

Figure S48. Selected isomers of $Cp^{*}Ru(dppm)H_2^{+}BF_4^{-}$ ion pair with partial explicit solvation with three molecules of CH_2Cl_2 at D3BJ-revPBE level of theory. CPCM solvent CH_2Cl_2.

Table S15. The effect of solid-state interactions on some transition metal hydrides. Data is taken from refs 55-57 and 59 (in the manuscript).

Part 1

	$IrH(H_2)Cl_2(P^iPr_3)_2)$	$[Os(H_2)Cl(dppe)]^+PF_6^-$	$Cp*OsH(H_2)H(PCy_3)^+BF_4^-$
Interaction	Ir-H---Cl-Ir	Os-H---PF_6^-	Os-H---PF_6^-
r(H-H) in solid state, neutron diffraction, Å	1.11	1.15	1.31
r(H-H) in solution from J_{HD}, Å	1.19-1.31 assuming H-(H_2) coupling as ±1Hz	≈1.25	0.99-1.21
r(H-H) in solution from $T_1(min)$ (slow regime)	≈1.5	1.35	1.12
r(H-H) in solution from $T_1(min)$ (fast regime)	-	1.08	-
r(H-H) from DFT calculations	1.1-bound; 1.5 non-bound	1.11 bound; 1.35 non-bound	1.51 bound; 1.10/1.68 non-bound

Part 2

	$[(PP_3)Co(H)_2]^+PF_6^-$	$[(PP_3)Co(H_2)]^+BPh_4^-$	
Interaction	Co-H---PF_6^-	-	
r(H-H) in solid state, X-ray diffraction, Å	≈1.94	coordination in the form of molecular hydrogen was deduced from the ligand geometry	

12. Probing the "continuum" of H-H bond activation by transition metals

Transition metal hydrides with temperature-dependent J_{HD} are so far exclusively found in the "non-classical" *r*(H-H) region that spans approximately from 1.0 to 1.6 A. We wondered if it is just a coincidence or there is something special about that region that differs it from regions of classical dihydrides and dihydrogen complexes. In an attempt to visualize the continuum of H-H bond activation, we computationally varied the electronic properties of the model compounds (^{Me4}X-PCP)IrH$_2$ in an incremental way by substituting the ligand H atoms with F, thus plotting *r*(H-H) vs. "electron-richness" of the ligands (Figure 11 in the manuscript). Fluorine atoms were placed into methyl groups one per group until all four groups were mono-substituted; then the procedure was repeated. Once all methyl groups were fully filled with fluorine atoms, pincer -CH$_2$- arms were populated. D3BJ-revPBE//BS1 level of theory with CPCM solvent toluene was used, as described in "Computational details". When "classical" regions with linear dependence of *r*(H-H) on the number of F atoms were long enough to allow fit, a slope of -0.003…-0.004 Å/F-atom is observed (see Figure 11 in the manuscript). In the S-shaped "non-classical" regions, slope could be two orders of magnitude higher, i.e., 0.3-0.4 Å/F-atom. Hence, in the "non-classical" regions *r*(H-H) was found very sensitive to electronic properties of the ligand, and therefore it would be very sensitive to the external stimuli as well.

Another example studied was $[Os(H_2)(en)_2X]^+$, where *r*(H-H) as a function of X was studied. Clearly, one has to account for both σ and π effects of X groups. Here we used Swain and Lupton parameters F and R taken from ref. 50. We expect that the effects behind these parameters will not be transmitted in the same way for organometallic compounds as it was for organic compounds. In an attempt to build a 2D plot, we defined a new parameter χ which consisted of a weighted sum of F and R, obtained by a multiple linear regression of *r*(H-H), F, and R: $\chi = -0.023F-0.053R$. The result is depicted on Figure S49. Again, there were two "classical" regions with linear dependence, and a "non-classical" region in between. Classical dihydrides were mainly represented by X groups with good π-donor ability, while classical dihydrogen complexes were mainly represented by X groups with good π-acceptor ability. Many "non-classical" complexes were represented by X groups that are good σ-donors with moderate/weak π-donor/acceptor properties. Within the structurally related series of compounds the trends were the same as for (^{Me4}X-PCP)IrH$_2$. For example, $[Os(H_2)(en)_2CH_3]^+$ revealed *r*(H-H) of 1.328 Å, while changing -CH$_3$ for -CH$_2$F to give $[Os(H_2)(en)_2CH_2F]^+$ resulted in *r*(H-H) change to 0.973 Å. The list of X groups studied is give in Table S16.

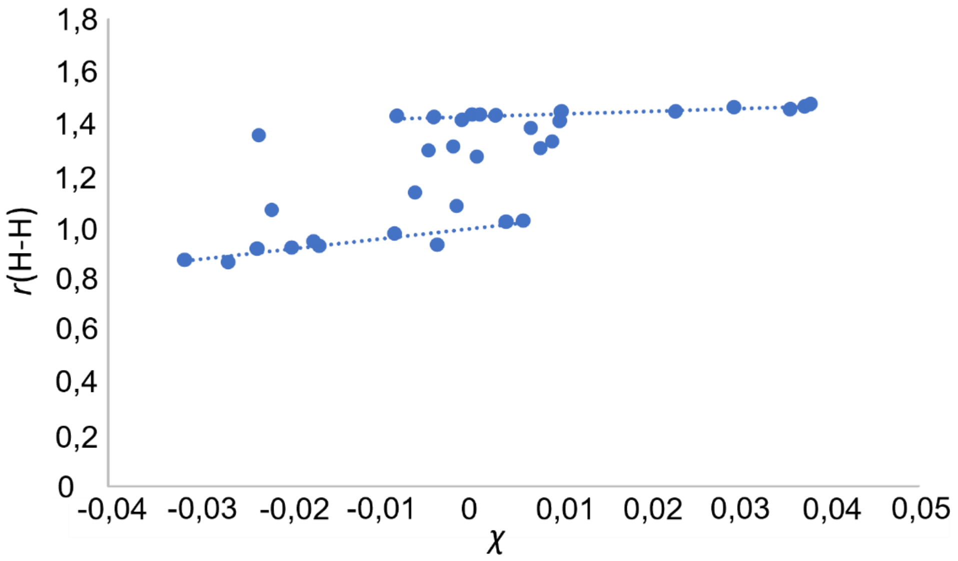

Figure S49. Dependence of *r*(H-H) on X group in $[Os(H_2)(en)_2X]^+$, plotted as χ (-0.023F-0.053R) versus *r*(H-H).

Table S16. List of X groups studied for $[Os(H_2)(en)_2X]^+$. D3BJ-revPBE//BS1 level of theory with CPCM solvent methanol was used. The groups were randomly chosen from ref 50. Groups that revealed high degree of steric bulk or additional interactions were excluded from Figure S49. In particular, for neutron-diffraction relevant $-OCOCH_3$, only the isomer that does not involve O---HN- contacts, was included.

Entry	group	F	R	r(H-H)
1	$-POCl_2$	0,7	0,2	0,862
2	$-IF_4$	0,98	0,17	0,871
3	$-SF_3$	0,63	0,17	0,914
4	-CN	0,51	0,15	0,918
5	-COMe	0,33	0,17	0,926
6	SiH_3	0,06	0,04	0,929
7	CF_3	0,38	0,16	0,942
8	$-CF_2H$	0,29	0,03	0,973
9	-Ph	0,12	-0,13	1,019
10	$-CH-CH_2$	0,13	-0,17	1,023
11	-Ph-OMe			1,041
12	$-NO_2$	0,65	0,13	1,064
13	$-CH_2F$	0,15	-0,04	1,078
14	PH2	0,05	0,09	1,131
15	$-CH_2OH$	0,03	-0,03	1,268
16	-PHMe			1,269
17	$-CH_2CN$	0,17	0,01	1,293
18	$-CH_2$CH3	0	-0,15	1,301

19	-PMe_2	0,05	0,01	1,308
20	-CH_3	0,01	-0,18	1,328
21	-$C(CN)_3$	0,92	0,04	1,35
22	-SMe	0,23	-0,23	1,379
23	-N_3	0,48	-0,4	1,407
24	-H	0,03	0	1,412
25	-$OCOCH_3$	0,42	-0,11	1,422
26	-OCN	0,69	-0,15	1,426
27	-SO_4H			1,427
28	-I	0,42	-0,24	1,428
29	-Cl	0,42	-0,19	1,432
30	-Br	0,45	-0,22	1,432
31	NH-OH	0,11	-0,45	1,441
32	-F	0,45	-0,39	1,443
33	-OMe	0,29	-0,56	1,444
34	-NH-NH_2	0,22	-0,77	1,453
35	-OH	0,33	-0,7	1,46
36	NH_2	0,08	-0,74	1,464
37	-NHMe	0,03	-0,73	1,472
38	-$B(NH_2)_2$			1,598

13. Examination of transition metal hydrides with XRD-based *r*(H-H) 1.15-1.25 Å

Structures were found in CSD database. Criteria: *r*(H-H) 1.15-1.25 Å, hydrides are not bridging, few W, Mo and Re based structures were excluded to reduce the number of calculations. In most of the cases, except for entry 3, there was a strong mismatch between the calculated and experimental data. We suppose this mainly reflects poor quality of hydride position determination by XRD, including the possibility of unresolved weighted-average positions. Also, it might indicate that structures with *r*(H-H) around 1.2 Å are comparatively rare, and, as it was for three neutron diffraction structures discussed in the text, some of them may require additional interactions in solid state for stabilization.

Table S17. Transition metal hydrides with XRD-based *r*(H-H) 1.15-1.25 Å, examined by DFT calculations. DFT-based distances from original publications are also provided where available.

Entry	Code	Complex	M	*r*(H-H)-exp, Å	*r*(H-H)-calc, Å	Reference
1	HECWUJ	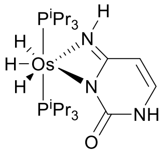	Os	1.168	This work: 1.640; 1.610 Solvent: C_6D_6	*Inorg. Chem.* 2012, **51**, 9522–9528

2	VAPPOS	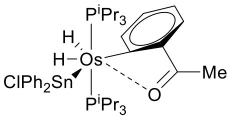	Os	1.174	Original work: truncated model 1.712	*Organometallics* 2003, **22**, 3753-3765
3	VAWLOX	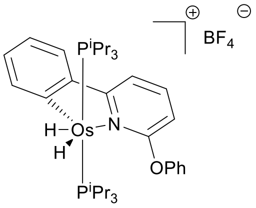	Os	1.183	Original work: 1.243 (no solvation); This work 1.135, Solvent: CH_2Cl_2	*Chem.-Eur.J.* 2017, **23**, 1526
4	ZUTTEQ	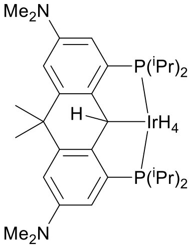	Ir	1.214	Original work: 1.90 (dihydride isomer) or 0.86 (dihydrogen isomer)	*Chem. Sci.*, 2020, **11**, 10705
5	DONXAI	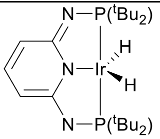	Ir	1.214 molecule 1; 1.37 molecule 2	This work: 1.607 Solvent: C_6D_6	*Dalton Trans.*, 2019, **48**, 12812
6	HINNUN	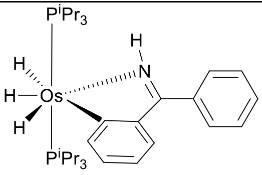	Os	1.223	Not attempted due to experimental correction to 1.46 A	*Organometallics* 1998, **17**, 4065-4076 Correction: *Organometallics* 2006, **25**, 3481-3485
7	XIQLOY	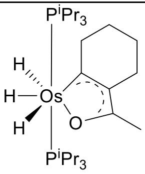	Os	1.228	This work: 1.650; 1.737 Solvent: C_6D_6	*Organometallics* 2001, **20**, 2635-2638
8	TAJWIL	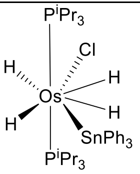	Os	1.237	Original work: 1.671 (truncated)	*Organometallics* 2003, **22**, 2087-2096

9	LIJKOG	(POP)OsH4 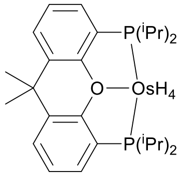	Os	1.244	Original work: 1.683	*Inorg. Chem.* 2013, **52**, 6199−6213
10	GIXMEG	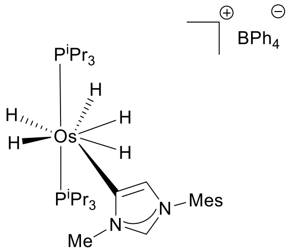	Os	1.246	This work: 1.57 Solvent: CH_2Cl_2	*Organometallics* 2008, **27**, 445–450
11	XEKMUV	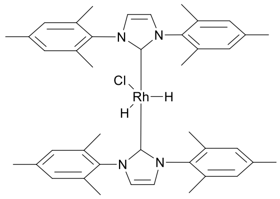	Rh	1.250	This work: 1.928 Solvent: THF	*Organometallics* 2000, **19**, 1194-1197

13. References

1) I. Gottker-Schnetmann, P. S. White and M. Brookhart, *Organometallics* 2004, **23**, 1766-1776.

2) (a) M. Gupta, C. Hagen, R. J. Flesher, W. C. Kaska and C. M. Jensen, *Chem. Commun.* 1996, 2083-2084. (b) M. Gupta, C. Hagen, W. C. Kaska, R. E. Cramer and C. M. Jensen. *J. Am. Chem. Soc.* 1997, **119**, 840-841.

3) N. T. Mucha and R. Waterman. *Organometallics* 2015, **34**, 3865-3872.

4) I. Gottker-Schnetmann, D. M. Heinekey and M. Brookhart, *J. Am. Chem. Soc.* 2006, **128**, 17114-17119.

5) Crysalis CCD; Oxford Diffraction Ltd.: Abingdon, Oxfordshire, UK (2005).

6) Crysalis RED; Oxford Diffraction Ltd.: Abingdon, Oxfordshire, UK (2005).

7) V. Petříček, M. Dušek, L. Palatinus. Crystallographic Computing System JANA2006: General features. *Z. Kristallogr. Cryst. Mater.* 2014, **229**, 345-352.

8) C. F. Macrae, P. R. Edgington, P. McCabe, E. Pidcock, G. P. Shields, R. Taylor, M. Towler and J. van de Streek. Mercury: visualization and analysis of crystal structures. *J. Appl. Cryst.*, 2006, **39**, 453-457.

9) D. A. Keen, M. J. Gutmann and C. C. Wilson, *J. App. Cryst.* 2006, **39**, 714–722.

10) M. J. Gutmann, SXD2001. ISIS Neutron and Muon Facility, Rutherford Appleton Laboratory, Oxfordshire, England, 2005.

11) G. M. Sheldrick, *Acta Cryst*., 2008, **A64**, 112-122.

12) H. B. Bürgi and S. C. Capelli, *Acta Cryst.* 2000, **A56**, 403-412.

13) H. B. Bürgi, M. Förtsch, S. C. Capelli and J. Hauser. "NKA: Program for Normal Coordinate Analysis from Anisotropic Displacement Parameters at multiple temperatures". Version 5.1.17, University of Bern, Switzerland, 2004.

14) H. B. Bürgi, S. C. Capelli, A. E. Goeta, J. A. K. Howard, M. A. Spackman and D. S. Yufit, *Chem. Eur. J.,* 2002, **8**, 3512-3521.

15) J. P. Coliman, P. S. Wagenknecht, J. E. Hutchison, N. S. Lewis, M. A. Lopez, R. Guilard, M. L'Her, A. A. Bothner-By and P. K. Mishra, *J. Am. Chem. Soc*., 1992, **114**, 5654-5664.

16) S. Sabo-Etienne, V. Rodriguez, B. Donnadieu, B. Chaudret, H. A. el Makarim, J.-C. Barthelat, S. Ulrich, H.-H. Limbach and C. Moise, *New J. Chem.*, 2001, **25**, 55-62.

17) X. S. Bogle and D. A. Singleton, *J. Am. Chem. Soc*. 2011, **133**, 17172–17175.

18) K. E. Janak and G. Parkin, *J. Am. Chem. Soc.* 2003, **125**, 13219-13224.

19) In eMagRes (eds R.K. Harris and R.L. Wasylishen), John Wiley & Sons, Ltd, 2007.

20) D. Bao, B. Millare, W. Xia, B. G. Steyer, A. A. Gerasimenko, A. Ferreira, A. Contreras and V. I. Vullev, *J. Phys. Chem. A* 2009, **113**, 1259–1267.

21) 1) (a) F. Neese, The ORCA program system" *Wiley Interdiscip. Rev. Comput. Mol. Sci.*, 2012, **2**, 73–78. (b) F. Neese, "Software update: the ORCA program system, version 4.0" *Wiley Interdiscip. Rev. Comput. Mol. Sci.*, 2017, **8**, e1327.

22) Y. Zhang and W. Yang, *Phys. Rev. Lett.* 1996, **77**, 3865.

23) Grimme, S. Ehrlich and L. Goerigk, *J. Comput. Chem.*, 2011, **32**, 1456-1465.

24) S. Grimme, J. Antony, S. Ehrlich and H. Krieg. *J. Chem. Phys.*, 2010, **132**, 154104.

25) F. Weigend and R. Ahlrichs, *Phys. Chem. Chem. Phys.* 2005, **7**, 3297.

26) J. Zheng, X. Xu and D. G. Truhlar, *Theor. Chem. Acc.* 2011, **128**, 295–305.

27) G. L. Stoychev, A. A. Auer and F. Neese, *J. Chem. Theory Comput.* 2017, **13**, 554–562.

28) M. Cossi and V. Barone, *J. Phys. Chem. A* 1998, **102**, 1995–2001.

29) (a) C. Riplinger and F. Neese, *J. Chem. Phys.* 2013, **138**, 034106. (b) C. Riplinger, B. Sandhoefer, A. Hansen and F. Neese, *J. Chem. Phys*. 2013, **139**, 134101.

30) D. A. Pantazis, X.-Y. Chen, C, R. Landis and F. Neese, *J. Chem. Theory Comput.* 2008, **4**, 908–919.

31) C. van Wüllen. *J. Chem. Phys.* 1998, **109**, 392–399.

32) D. Rappoport and F. Furche, *J. Chem. Phys.* 2010, **133**, 134105.

33) C. Adamo and V. Barone, J. *Chem. Phys.* 1999, **110**, 6158-5170.

34) Y. Zhao and D. G. Truhlar, *J. Chem. Phys.* 2006, **125**, 194101.

35) J. M. Tao, J. P. Perdew, V. N. Staroverov and G. E. Scuseria, *Phys. Rev. Lett.* 2003, **91**, 146401.

36) Y. Zhao and D. G. Truhlar, *Theor. Chem. Acc.* 2008, **120**, 215.

37) (a) A. Najibi and L. Goerigk, *J. Chem. Theory Comput.* 2018, **14**, 5725–5738. (b) J. D. Chai and M. Head-Gordon, *J. Chem. Phys.* 2008, **128**, 084106.

38) S. Grimme, *J. Chem. Phys.* 2003, **118**, 9095-9102.

39) W. Jiang, N. J. DeYonker and A. K. Wilson, *J. Chem. Theory Comput.* 2012, **8**, 460–468.

40) I. Göttker-Schnetmann, P. S. White and M. Brookhart, *J. Am. Chem. Soc*. 2004, **126**, 1804-1811.

41) M. Findlater, K. M. Schultz, W. H. Bernskoetter, A. Cartwright-Sykes, D. M. Heinekey and M. Brookhart, *Inorg. Chem.* 2012, **51**, 4672-4678.

42) A. V. Polukeev, O. Y. Abdelaziz and O. F. Wendt, *Organometallics* 2022, **41**, 859–873.

43) A. V. Polukeev, P. V. Petrovskii, A. S. Peregudov, M. G. Ezernitskaya and A. A. Koridze. *Organometallics* 2013, **32**, 1000–1015.

44) J. P. Perdew, K. Burke and M. Ernzerhof, *Phys. Rev. Lett.* 1996, **77**, 3865− 3868.

45) (a) K. G. Dyall, *Theor. Chem. Acc.* 2004, **112**, 403−409. (b) K. G. Dyall and A. S. P. Gomes, *Theor. Chem. Acc.* 2010, **125**, 97.

46) W. Kutzelnigg, U. Fleischer and M. Schindler, *In Deuterium and Shift Calculation. NMR Basic Principles and Progress*; U. Fleischer, W. Kutzelnigg, H. H. Limbach, G. J. Martin, M. L. Martin and M. Schindler, Eds.; Springer, 1991; Vol. **23**, pp 165−262; ISBN 978- 3-642-75932-1.

47) J. K. Law, H. Mellows and D. M. Heinekey. *J. Am. Chem. Soc.* 2002, **124**, 1024-1030.

48) S. Gründemann, H.-H. Limbach, G. Buntkowsky, S. Sabo-Etienne and B.Chaudret, *J. Phys. Chem. A* 1999, **103**, 4752–4754. (b) R. Gelabert, M. Moreno, J. M. Lluch, A. Lledos, V. Pons and D. M. Heinekey, *J. Am. Chem. Soc.* 2004, **126**, 8813-8822.

49) C. E. Webster, C. L. Gross, D. M. Young, G. S. Girolami, A. J. Schultz, M. B. Hall and J. Eckert, *J. Am. Chem. Soc.* 2005, **127**, 15091-15101.

50) C. Hansch, A. Leo and R. W. Taft, *Chem. Rev.* 1991, **91**, 165-195.

14. List of Cartesian coordinates

Electronic energies are given at BS1//D3BJ-revPBE level

72

(p-MeO-POCOP)IrH2 S tol, E = -1914.031455

C	-0.356080000	1.195971000	-3.609131000
C	-0.233313000	1.185359000	-2.211742000
C	-0.171273000	-0.025093000	-1.466040000
C	-0.236631000	-1.241233000	-2.191370000
C	-0.357824000	-1.281063000	-3.595698000
C	-0.417090000	-0.047871000	-4.291923000
H	-0.404360000	2.132228000	-4.180059000
H	-0.403462000	-2.247718000	-4.109334000
O	-0.180692000	-2.435166000	-1.505760000
O	-0.168987000	2.385675000	-1.538023000
P	-0.027171000	-2.264670000	0.224698000
P	-0.019564000	2.237341000	0.192665000
Ir	0.007907000	-0.011569000	0.595928000
H	0.952821000	0.013177000	1.895531000
H	-0.675962000	-0.013886000	2.052829000
C	1.601099000	-3.203382000	0.466730000
C	-1.578531000	-3.229699000	0.731010000
C	1.599023000	3.199496000	0.411358000
C	-1.579686000	3.184702000	0.701945000
C	1.981865000	-3.142404000	1.961534000
H	3.000807000	-3.563740000	2.090719000
H	1.987236000	-2.097935000	2.328536000
H	1.292618000	-3.731416000	2.594880000
C	-1.520292000	-3.490172000	2.249757000
H	-1.279556000	-2.565369000	2.811219000
H	-2.510765000	-3.853423000	2.595186000
H	-0.772592000	-4.264989000	2.506428000
C	2.635189000	-2.395279000	-0.355473000
H	2.411640000	-2.435258000	-1.438441000
H	2.646773000	-1.331799000	-0.044230000
H	3.643865000	-2.827798000	-0.189798000
C	1.562119000	-4.658510000	-0.033069000
H	2.594200000	-5.068416000	-0.030320000
H	0.949057000	-5.303984000	0.623223000
H	1.171106000	-4.723913000	-1.066206000
C	-1.783164000	-4.541199000	-0.051933000
H	-1.047286000	-5.316770000	0.223046000
H	-2.792518000	-4.940846000	0.181711000
H	-1.724801000	-4.369250000	-1.142920000
C	-2.745061000	-2.262525000	0.409666000
H	-2.639333000	-1.309144000	0.962856000
H	-2.785241000	-2.025469000	-0.671298000
H	-3.703394000	-2.744204000	0.696560000
C	1.815622000	3.462771000	1.915170000

H	1.678435000	2.539406000	2.512655000
H	2.852616000	3.824910000	2.075811000
H	1.127693000	4.239311000	2.300915000
C	-1.687668000	3.158902000	2.241444000
H	-2.669621000	3.581759000	2.540459000
H	-1.621778000	2.123468000	2.627920000
H	-0.899980000	3.764259000	2.727536000
C	2.687054000	2.228947000	-0.112079000
H	2.680124000	1.276679000	0.452963000
H	2.532758000	1.990534000	-1.182381000
H	3.682172000	2.708868000	-0.002127000
C	1.661570000	4.508566000	-0.399185000
H	0.991321000	5.287409000	0.004350000
H	2.698036000	4.904646000	-0.355477000
H	1.403660000	4.334898000	-1.460591000
C	-1.635099000	4.627362000	0.168290000
H	-2.652825000	5.036581000	0.341468000
H	-0.920149000	5.289544000	0.691157000
H	-1.430904000	4.667193000	-0.918533000
C	-2.741238000	2.358965000	0.095464000
H	-2.712520000	2.371762000	-1.010624000
H	-2.695443000	1.303462000	0.430011000
H	-3.705721000	2.797787000	0.425863000
O	-0.535243000	0.044067000	-5.649924000
C	-0.601908000	-1.158766000	-6.412878000
H	0.316263000	-1.773704000	-6.289874000
H	-1.486053000	-1.772770000	-6.134237000
H	-0.693039000	-0.847959000	-7.470193000

72

(p-MeO-POCOP)IrH2 NS tol, E = -1914.030111

Ir	0.784246000	-0.944442000	-0.004255000
P	2.301091000	0.763768000	0.016029000
P	-1.216209000	-2.045357000	0.006624000
O	1.343270000	2.220568000	0.023985000
C	4.059622000	2.369471000	1.640437000
O	-2.424683000	-0.785739000	0.003157000
C	3.821164000	2.525291000	-1.696090000
C	-3.173712000	-3.421810000	1.615175000
C	-0.877949000	3.119196000	0.020071000
C	-1.009801000	-4.283228000	-1.662744000
C	-2.815790000	1.576016000	0.007892000
C	-0.734396000	-4.090371000	1.853969000
C	-3.270715000	-3.117075000	-1.718957000
C	-1.912385000	0.492860000	0.002885000
C	3.274759000	1.045357000	1.615933000
C	-2.278486000	2.887038000	0.015996000
C	-1.525009000	-1.854019000	2.709662000
C	-0.506787000	0.673964000	0.002600000
C	4.213478000	-0.159658000	1.837351000
C	2.241397000	0.844465000	-2.703243000
C	-1.716755000	-2.925361000	1.607935000
C	4.411253000	0.049970000	-1.680526000

C	-0.018215000	2.010217000	0.014240000
C	-1.747134000	-2.931148000	-1.583488000
C	-1.248771000	-1.997610000	-2.714761000
C	3.276537000	1.089003000	-1.576859000
C	2.192137000	1.060942000	2.723306000
H	4.940072000	2.340743000	0.972066000
H	3.420321000	3.225825000	1.353395000
H	4.431929000	2.548719000	2.671157000
H	3.025725000	3.271678000	-1.512910000
H	4.655330000	2.719098000	-0.999200000
H	4.206230000	2.675795000	-2.726738000
H	-3.877229000	-2.621829000	1.315764000
H	-3.317108000	-4.292644000	0.948643000
H	-3.439158000	-3.745317000	2.643811000
H	-0.497151000	4.148598000	0.029112000
H	0.075338000	-4.164641000	-1.470786000
H	-1.418685000	-5.018865000	-0.943793000
H	-1.134409000	-4.706595000	-2.681359000
H	-3.894105000	1.381870000	0.008571000
H	0.315546000	-3.740716000	1.822033000
H	-0.929509000	-4.519182000	2.859209000
H	-0.854099000	-4.903201000	1.113691000
H	-3.669665000	-3.863715000	-1.010385000
H	-3.805474000	-2.161388000	-1.564103000
H	-3.495269000	-3.477393000	-2.745078000
H	-0.494776000	-1.446735000	2.692566000
H	-2.230307000	-1.011230000	2.580783000
H	-1.711277000	-2.319015000	3.700165000
H	5.034794000	-0.192167000	1.097396000
H	4.671756000	-0.078899000	2.845262000
H	3.656550000	-1.115112000	1.783629000
H	1.395178000	1.555350000	-2.635032000
H	2.736892000	0.986079000	-3.686648000
H	1.828826000	-0.182117000	-2.651139000
H	4.036462000	-0.977634000	-1.501841000
H	4.845318000	0.085603000	-2.701651000
H	5.228444000	0.258512000	-0.963777000
H	-0.152731000	-1.849870000	-2.657323000
H	-1.502422000	-2.450781000	-3.696112000
H	-1.729738000	-1.001832000	-2.657024000
H	1.507663000	1.922691000	2.607480000
H	1.585692000	0.134002000	2.699273000
H	2.690023000	1.139366000	3.712250000
H	1.610178000	-1.981576000	0.888394000
H	1.682908000	-2.066484000	-0.745789000
O	-3.051076000	4.016204000	0.021839000
C	-4.466448000	3.871626000	0.010075000
H	-4.819284000	3.337148000	-0.899519000
H	-4.833965000	3.327146000	0.907895000
H	-4.882166000	4.896657000	0.012504000

84

(p-MeO-POCOP)IrH2-IC6F5 NS-bound-a1, E = -2938.867375

Ir	3.448160000	4.491929000	4.147316000
P	1.467361000	3.568627000	3.461654000
P	5.161172000	6.011153000	4.427934000
O	0.675639000	4.826942000	2.578607000
C	0.046437000	1.804821000	1.722996000
O	4.627911000	7.406017000	3.544897000
C	-1.259932000	3.428457000	4.393962000
C	7.464508000	7.173678000	3.198484000
C	0.813979000	7.100806000	1.817491000
C	5.645532000	5.659455000	7.170015000
C	2.838661000	8.427956000	2.316682000
C	7.830124000	5.099810000	4.609406000
C	6.107237000	8.000656000	6.279953000
C	3.371680000	7.302528000	2.978311000
O	0.927118000	9.329462000	1.068561000
C	1.469489000	2.215363000	2.145791000
C	1.552095000	8.310884000	1.735253000
C	6.654798000	4.938503000	2.377189000
C	2.675926000	6.075528000	3.084355000
C	2.261164000	1.004840000	2.682217000
C	0.519109000	4.497385000	5.821299000
C	6.874180000	5.814750000	3.633382000
C	0.489934000	1.984465000	5.553773000
C	1.387475000	6.011544000	2.488968000
C	5.212113000	6.754156000	6.170765000
C	3.740544000	7.141718000	6.459326000
C	0.206914000	3.328207000	4.853044000
C	2.215639000	2.825633000	0.934785000
H	-0.480825000	1.258544000	2.527298000
H	-0.559673000	2.682353000	1.427348000
H	0.114835000	1.124764000	0.848280000
H	-1.440374000	4.364257000	3.832946000
H	-1.562867000	2.575745000	3.761638000
H	-1.913470000	3.429317000	5.291591000
H	6.815892000	7.678308000	2.459813000
H	7.623482000	7.860404000	4.048551000
H	8.451785000	6.984955000	2.727312000
H	-0.182779000	7.037989000	1.362028000
H	5.046652000	4.737244000	7.037499000
H	6.713243000	5.395888000	7.069469000
H	5.486015000	6.034269000	8.202646000
H	3.423017000	9.353668000	2.269690000
H	7.383533000	4.176914000	5.024245000
H	8.746128000	4.802655000	4.058797000
H	8.136428000	5.759616000	5.442763000
H	7.178947000	7.751258000	6.167038000
H	5.834919000	8.759628000	5.521931000
H	5.975761000	8.455020000	7.284642000
H	6.362236000	3.907987000	2.651061000
H	5.876518000	5.364001000	1.713494000
H	7.605688000	4.885392000	1.807643000
H	1.759990000	0.519512000	3.539619000

H	2.353654000	0.250795000	1.873190000
H	3.279838000	1.296541000	3.000469000
H	0.399557000	5.481877000	5.328118000
H	-0.176023000	4.457347000	6.685523000
H	1.554631000	4.420509000	6.215794000
H	1.564036000	1.874671000	5.804738000
H	-0.089324000	1.939276000	6.499501000
H	0.181160000	1.123365000	4.931049000
H	3.071713000	6.257956000	6.409926000
H	3.670585000	7.556501000	7.486080000
H	3.363514000	7.901400000	5.749248000
H	1.666663000	3.687421000	0.511301000
H	3.233061000	3.164143000	1.210119000
H	2.310137000	2.048502000	0.148685000
I	5.612770000	1.781556000	4.939191000
F	8.106943000	-3.008460000	6.109965000
F	10.397828000	-2.369396000	4.737817000
F	6.029105000	-1.235901000	6.208112000
F	10.615510000	0.051305000	3.458620000
F	8.549741000	1.836326000	3.542340000
C	8.203779000	-1.820374000	5.487267000
C	7.142366000	-0.895180000	5.526938000
C	7.233595000	0.348146000	4.880897000
C	8.413952000	0.654482000	4.183003000
C	9.489482000	-0.252692000	4.128601000
C	9.380151000	-1.495461000	4.783407000
H	4.231762000	3.709296000	3.077120000
H	3.857861000	3.282185000	5.258509000
C	1.606303000	10.575714000	0.942780000
H	1.817956000	11.030650000	1.935456000
H	2.563392000	10.468039000	0.386549000
H	0.929036000	11.240829000	0.375417000

84

(p-MeO-POCOP)IrH2-IC6F5 NS-bound-a2, E = -2938.867901

Ir	2.582910000	4.199336000	4.650901000
P	1.244089000	2.342811000	4.516183000
P	3.466830000	6.261042000	4.209118000
O	-0.073514000	2.835371000	3.490845000
C	0.797839000	-0.243094000	3.368996000
O	2.320313000	7.007406000	3.133938000
C	-1.076189000	1.170228000	5.777192000
C	5.418467000	7.786961000	2.766868000
C	-0.988327000	4.607072000	2.159778000
C	4.599580000	7.259746000	6.558929000
C	0.238683000	6.750692000	1.974970000
C	6.164899000	5.618578000	3.860272000
C	3.369288000	8.989958000	5.150361000
C	1.232776000	6.218067000	2.819394000
O	-1.897322000	6.332250000	0.847615000
C	1.863874000	0.857640000	3.521284000
C	-0.870524000	5.928653000	1.652301000
C	4.649266000	5.567359000	1.842187000

C	1.159314000	4.903888000	3.348903000
C	3.138586000	0.301367000	4.188843000
C	-0.060880000	3.277198000	6.706855000
C	5.015935000	6.345541000	3.129294000
C	1.152892000	1.078958000	7.006375000
C	0.024608000	4.118781000	2.996981000
C	3.405751000	7.522799000	5.618834000
C	2.080308000	7.190721000	6.350063000
C	0.249226000	1.900941000	6.065758000
C	2.223747000	1.431633000	2.129237000
H	0.610211000	-0.769380000	4.323548000
H	-0.158303000	0.164069000	2.988722000
H	1.163463000	-0.996785000	2.640412000
H	-1.688031000	1.725860000	5.042346000
H	-0.920773000	0.144075000	5.401518000
H	-1.652516000	1.096572000	6.723440000
H	4.567830000	8.352792000	2.341281000
H	5.813724000	8.337106000	3.641176000
H	6.223782000	7.753020000	2.003472000
H	-1.860399000	3.997221000	1.890729000
H	4.683320000	6.185723000	6.820190000
H	5.556235000	7.590621000	6.111400000
H	4.454164000	7.830660000	7.499565000
H	0.343278000	7.772058000	1.592605000
H	5.881563000	4.583963000	4.131368000
H	7.045244000	5.572743000	3.185927000
H	6.474683000	6.141614000	4.783573000
H	4.325130000	9.314997000	4.704182000
H	2.561429000	9.154453000	4.413058000
H	3.172861000	9.638210000	6.030122000
H	4.307807000	4.540416000	2.075100000
H	3.847974000	6.077308000	1.275039000
H	5.549264000	5.503169000	1.196446000
H	2.952517000	-0.103594000	5.200098000
H	3.538147000	-0.523688000	3.563032000
H	3.921067000	1.077409000	4.276093000
H	-0.656428000	3.916555000	6.025819000
H	-0.646700000	3.123184000	7.636988000
H	0.872408000	3.817280000	6.964854000
H	2.143627000	1.556991000	7.142044000
H	0.669120000	1.005386000	8.002629000
H	1.307287000	0.048910000	6.632029000
H	2.085905000	6.151630000	6.736812000
H	1.953611000	7.883930000	7.207608000
H	1.206315000	7.307050000	5.679577000
H	1.327072000	1.803523000	1.598880000
H	2.947728000	2.265141000	2.211950000
H	2.682000000	0.625684000	1.519588000
I	5.230154000	2.574465000	6.687937000
F	7.718653000	-2.197245000	7.732765000
F	9.731214000	-1.068235000	9.221327000
F	5.774724000	-0.621686000	6.637994000

F	9.801869000	1.647938000	9.618086000
F	7.867211000	3.241691000	8.532383000
C	7.744757000	-0.866429000	7.922195000
C	6.750481000	-0.039446000	7.364975000
C	6.768990000	1.353250000	7.557328000
C	7.807227000	1.911922000	8.321666000
C	8.812304000	1.104166000	8.888409000
C	8.778602000	-0.289626000	8.686122000
H	3.853302000	3.434678000	4.167965000
H	3.403446000	3.898804000	6.066589000
C	-1.855585000	7.645393000	0.292545000
H	-1.849572000	8.425168000	1.085104000
H	-0.965648000	7.786787000	-0.358743000
H	-2.772802000	7.751477000	-0.315780000

68

(p-H-POCOP)IrH2 S tol, E = -1799.616265

C	-0.354701000	1.218808000	-3.616747000
C	-0.231283000	1.204982000	-2.211333000
C	-0.172435000	-0.010706000	-1.481486000
C	-0.239840000	-1.230008000	-2.204501000
C	-0.361687000	-1.250776000	-3.609990000
C	-0.417591000	-0.017679000	-4.292849000
H	-0.399448000	2.171636000	-4.162545000
H	-0.411545000	-2.206276000	-4.150646000
H	-0.513358000	-0.020402000	-5.389901000
O	-0.185125000	-2.423510000	-1.516105000
O	-0.164652000	2.401992000	-1.529944000
P	-0.029182000	-2.259496000	0.211691000
P	-0.017943000	2.246393000	0.199413000
Ir	0.007246000	-0.005656000	0.590183000
H	0.936161000	0.012527000	1.899522000
H	-0.656487000	-0.015036000	2.054810000
C	1.600025000	-3.196077000	0.450822000
C	-1.580344000	-3.222162000	0.720651000
C	1.601390000	3.204310000	0.426445000
C	-1.579004000	3.189429000	0.712091000
C	1.979332000	-3.145151000	1.946286000
H	2.999074000	-3.565190000	2.073100000
H	1.982715000	-2.103521000	2.321403000
H	1.291012000	-3.740448000	2.574683000
C	-1.522872000	-3.480437000	2.239787000
H	-1.281837000	-2.555213000	2.800426000
H	-2.513802000	-3.842558000	2.585009000
H	-0.775986000	-4.255477000	2.497953000
C	2.632821000	-2.379886000	-0.365119000
H	2.409849000	-2.412147000	-1.448436000
H	2.642787000	-1.318698000	-0.045619000
H	3.642280000	-2.811613000	-0.202442000
C	1.563277000	-4.647335000	-0.060206000
H	2.596076000	-5.055325000	-0.061357000
H	0.951652000	-5.298907000	0.591312000

H	1.171646000	-4.704922000	-1.093552000
C	-1.784797000	-4.534469000	-0.060831000
H	-1.049893000	-5.310036000	0.216936000
H	-2.794874000	-4.932929000	0.171545000
H	-1.724203000	-4.364223000	-1.151959000
C	-2.745539000	-2.253905000	0.397355000
H	-2.638445000	-1.299512000	0.948891000
H	-2.786176000	-2.019624000	-0.684168000
H	-3.704539000	-2.733174000	0.685825000
C	1.818172000	3.455325000	1.932368000
H	1.679816000	2.527608000	2.522759000
H	2.855679000	3.814687000	2.095692000
H	1.131471000	4.229805000	2.324319000
C	-1.686794000	3.158813000	2.251508000
H	-2.669040000	3.580048000	2.551736000
H	-1.620482000	2.122423000	2.635352000
H	-0.899624000	3.763413000	2.739298000
C	2.687561000	2.235649000	-0.104483000
H	2.678204000	1.278666000	0.452740000
H	2.533867000	2.006838000	-1.176945000
H	3.683787000	2.711982000	0.010413000
C	1.665573000	4.519637000	-0.373595000
H	0.995971000	5.295822000	0.036161000
H	2.702416000	4.914241000	-0.326437000
H	1.407833000	4.354784000	-1.436451000
C	-1.636316000	4.633582000	0.182675000
H	-2.654821000	5.040565000	0.356319000
H	-0.922782000	5.295283000	0.708009000
H	-1.431365000	4.676891000	-0.903868000
C	-2.738865000	2.363306000	0.102823000
H	-2.709797000	2.378923000	-1.003207000
H	-2.691804000	1.306959000	0.434865000
H	-3.704305000	2.799432000	0.433853000

68

(p-H-POCOP)IrH2 NS tol, E = -1799.616415

C	-0.048925000	1.234677000	-3.638108000
C	-0.023826000	1.217198000	-2.227635000
C	-0.007486000	0.000004000	-1.498104000
C	-0.024858000	-1.217188000	-2.227619000
C	-0.049953000	-1.234680000	-3.638091000
C	-0.060199000	-0.000002000	-4.320482000
H	-0.062674000	2.188985000	-4.183016000
H	-0.064499000	-2.188985000	-4.182981000
H	-0.079977000	0.000000000	-5.421511000
O	-0.025607000	-2.412480000	-1.539352000
O	-0.023554000	2.412498000	-1.539411000
P	0.002049000	-2.252724000	0.195197000
P	0.002373000	2.252826000	0.195104000
Ir	0.037612000	0.000026000	0.581039000
H	0.692683000	0.000235000	2.069935000
H	-0.924594000	0.000136000	1.841349000
C	1.596428000	-3.221766000	0.532196000

C	-1.592547000	-3.185498000	0.609311000
C	1.596402000	3.221726000	0.533752000
C	-1.592694000	3.185563000	0.607569000
C	1.689632000	-3.506372000	2.044687000
H	2.709733000	-3.873088000	2.283889000
H	1.505729000	-2.591064000	2.642108000
H	0.971631000	-4.286939000	2.361712000
C	-1.814467000	-3.125583000	2.135897000
H	-1.787493000	-2.081860000	2.503805000
H	-2.812134000	-3.552104000	2.370561000
H	-1.059696000	-3.710700000	2.693362000
C	2.720604000	-2.241365000	0.113528000
H	2.653901000	-1.986840000	-0.962224000
H	2.664744000	-1.298183000	0.692353000
H	3.706001000	-2.718649000	0.297264000
C	1.728048000	-4.518949000	-0.288875000
H	2.755472000	-4.919383000	-0.157834000
H	1.022159000	-5.301142000	0.040665000
H	1.566811000	-4.328866000	-1.366345000
C	-1.602645000	-4.641031000	0.108636000
H	-0.919202000	-5.283664000	0.694505000
H	-2.626799000	-5.054431000	0.223559000
H	-1.325936000	-4.705138000	-0.960879000
C	-2.707216000	-2.379492000	-0.101778000
H	-2.687441000	-1.315591000	0.206694000
H	-2.597505000	-2.421642000	-1.201960000
H	-3.692637000	-2.812504000	0.168895000
C	1.687850000	3.506407000	2.046349000
H	1.503286000	2.591074000	2.643530000
H	2.707644000	3.873194000	2.286734000
H	0.969383000	4.286908000	2.362473000
C	-1.816390000	3.125326000	2.133891000
H	-2.814337000	3.551774000	2.367497000
H	-1.789835000	2.081522000	2.501604000
H	-1.062276000	3.710370000	2.692325000
C	2.720980000	2.241206000	0.116507000
H	2.664474000	1.298177000	0.695531000
H	2.655393000	1.986470000	-0.959261000
H	3.706201000	2.718508000	0.301138000
C	1.728983000	4.518830000	-0.287266000
H	1.022550000	5.300954000	0.041279000
H	2.756184000	4.919411000	-0.154910000
H	1.569214000	4.328609000	-1.364931000
C	-1.602219000	4.641230000	0.107238000
H	-2.626505000	5.054577000	0.221215000
H	-0.919410000	5.283676000	0.694056000
H	-1.324410000	4.705650000	-0.961981000
C	-2.706478000	2.379697000	-0.105088000
H	-2.595864000	2.422864000	-1.205135000
H	-2.686451000	1.315508000	0.202390000
H	-3.692289000	2.812079000	0.165167000

80

(p-H-POCOP)IrH2-IC6F5 NS-bound-a1, E = -2824.453636

Ir	3.001430000	4.046085000	4.100840000
P	1.062575000	3.034240000	3.414446000
P	4.643505000	5.643488000	4.397226000
O	0.247058000	4.238141000	2.485793000
C	-0.330090000	1.201279000	1.735360000
O	4.086826000	6.986888000	3.461820000
C	-1.652578000	2.898432000	4.378873000
C	6.953800000	6.870842000	3.247022000
C	0.319406000	6.503105000	1.656755000
C	5.018185000	5.353702000	7.163097000
C	2.279392000	7.907877000	2.158834000
C	7.326493000	4.815440000	4.684486000
C	5.471673000	7.686576000	6.248537000
C	2.848023000	6.828072000	2.864363000
H	0.559622000	8.560866000	1.004221000
C	1.088831000	1.633496000	2.151426000
C	1.015586000	7.726481000	1.558835000
C	6.242125000	4.611243000	2.407245000
C	2.188870000	5.580460000	2.985884000
C	1.885051000	0.448224000	2.734204000
C	0.138675000	4.016225000	5.755507000
C	6.388934000	5.496753000	3.667514000
C	0.117431000	1.497596000	5.560394000
C	0.918717000	5.447374000	2.372056000
C	4.605625000	6.420848000	6.125838000
C	3.117587000	6.788562000	6.345832000
C	-0.180862000	2.818906000	4.824606000
C	1.840449000	2.205378000	0.925404000
H	-0.860371000	0.684687000	2.557161000
H	-0.937426000	2.064626000	1.402776000
H	-0.253677000	0.488071000	0.888208000
H	-1.845888000	3.820769000	3.800173000
H	-1.953474000	2.030421000	3.766315000
H	-2.297826000	2.912197000	5.282391000
H	6.311574000	7.357340000	2.490784000
H	7.072521000	7.561662000	4.099992000
H	7.957732000	6.708141000	2.802180000
H	-0.666963000	6.366474000	1.191511000
H	4.429173000	4.424146000	7.037193000
H	6.089719000	5.094033000	7.097107000
H	4.829083000	5.752399000	8.181687000
H	2.816294000	8.864123000	2.085122000
H	6.890323000	3.882704000	5.088559000
H	8.271462000	4.541164000	4.172598000
H	7.579724000	5.489468000	5.524252000
H	6.551928000	7.455914000	6.191963000
H	5.220864000	8.424109000	5.462442000
H	5.283969000	8.159411000	7.235577000
H	5.961011000	3.575258000	2.671464000
H	5.485563000	5.019998000	1.708669000
H	7.217377000	4.576314000	1.879176000

H	1.375361000	-0.020474000	3.595583000
H	2.000154000	-0.325992000	1.947496000
H	2.895169000	0.761159000	3.059790000
H	0.000925000	4.987114000	5.241046000
H	-0.535347000	3.993832000	6.636862000
H	1.182511000	3.954799000	6.135254000
H	1.194850000	1.399716000	5.801809000
H	-0.450361000	1.477377000	6.513904000
H	-0.194742000	0.617747000	4.966524000
H	2.461347000	5.897403000	6.276284000
H	2.996649000	7.211282000	7.364469000
H	2.761977000	7.536362000	5.612662000
H	1.300752000	3.061075000	0.478344000
H	2.861893000	2.538559000	1.192520000
H	1.929007000	1.407120000	0.160133000
I	5.265802000	1.401310000	4.825798000
F	7.904825000	-3.372674000	5.696303000
F	10.183343000	-2.570696000	4.390468000
F	5.770636000	-1.677697000	5.894279000
F	10.331955000	-0.063403000	3.279266000
F	8.209720000	1.645645000	3.464004000
C	7.967919000	-2.142965000	5.155710000
C	6.876962000	-1.256689000	5.247736000
C	6.932768000	0.029769000	4.687628000
C	8.107350000	0.419598000	4.022352000
C	9.211874000	-0.447399000	3.917165000
C	9.137947000	-1.734505000	4.485581000
H	3.784265000	3.423183000	2.934453000
H	3.481437000	2.840582000	5.198239000

80

(p-H-POCOP)IrH2-IC6F5 NS-bound-a2, E = -2824.453483

Ir	2.580316000	4.222320000	4.658206000
P	1.245533000	2.355968000	4.540574000
P	3.456720000	6.285976000	4.194015000
O	-0.054061000	2.824734000	3.494569000
C	0.791117000	-0.273795000	3.519585000
O	2.356664000	6.982856000	3.056354000
C	-1.095730000	1.282606000	5.846607000
C	5.472205000	7.849681000	2.904738000
C	-0.937429000	4.555769000	2.066268000
C	4.455090000	7.339001000	6.585478000
C	0.295033000	6.679080000	1.841074000
C	6.168604000	5.659642000	3.993317000
C	3.195598000	9.005605000	5.124150000
C	1.274025000	6.182796000	2.724954000
C	1.858684000	0.833249000	3.602251000
C	-0.801786000	5.850520000	1.522198000
C	4.781032000	5.648103000	1.883231000
C	1.184340000	4.884864000	3.289975000
C	3.144959000	0.312161000	4.274249000
C	-0.011332000	3.389689000	6.701081000
C	5.062437000	6.396513000	3.208409000

C	1.138646000	1.167793000	7.067366000
C	0.060207000	4.091228000	2.947024000
C	3.289059000	7.542984000	5.597546000
C	1.954085000	7.139645000	6.274247000
C	0.251240000	1.983015000	6.105912000
C	2.191850000	1.340345000	2.178203000
H	0.612455000	-0.747755000	4.502897000
H	-0.168343000	0.113351000	3.126807000
H	1.149605000	-1.065562000	2.828952000
H	-1.697204000	1.835404000	5.101125000
H	-0.969587000	0.243581000	5.495961000
H	-1.667094000	1.248474000	6.798108000
H	4.648126000	8.413861000	2.427684000
H	5.793763000	8.386247000	3.816884000
H	6.332275000	7.841123000	2.202933000
H	-1.796288000	3.917203000	1.815963000
H	4.573663000	6.271660000	6.860402000
H	5.413566000	7.706778000	6.172104000
H	4.248954000	7.911199000	7.513933000
H	0.392142000	7.688359000	1.416910000
H	5.878674000	4.615678000	4.217643000
H	7.092031000	5.640425000	3.378014000
H	6.411198000	6.158405000	4.949620000
H	4.148959000	9.376221000	4.708811000
H	2.406622000	9.128132000	4.358797000
H	2.937644000	9.645210000	5.994321000
H	4.441246000	4.610292000	2.065457000
H	4.009148000	6.164099000	1.282292000
H	5.718754000	5.607556000	1.291721000
H	2.977436000	-0.040541000	5.307958000
H	3.536089000	-0.543314000	3.685154000
H	3.927321000	1.092399000	4.309285000
H	-0.584508000	4.028680000	6.000670000
H	-0.598662000	3.291741000	7.637642000
H	0.942821000	3.900251000	6.949567000
H	2.140349000	1.627173000	7.186622000
H	0.656484000	1.133266000	8.066501000
H	1.268754000	0.124593000	6.721514000
H	2.004839000	6.103639000	6.671137000
H	1.753013000	7.819598000	7.127908000
H	1.101228000	7.207353000	5.570839000
H	1.285321000	1.683368000	1.645628000
H	2.916314000	2.177171000	2.202718000
H	2.643145000	0.506776000	1.601661000
I	5.287364000	2.535710000	6.677973000
F	7.717814000	-2.304751000	7.549278000
F	9.786122000	-1.247741000	9.014408000
F	5.776179000	-0.669721000	6.541867000
F	9.913957000	1.456116000	9.476411000
F	7.982034000	3.109260000	8.478200000
C	7.772079000	-0.979584000	7.770718000
C	6.779568000	-0.121752000	7.258800000

C	6.826769000	1.264888000	7.484875000
C	7.893069000	1.786201000	8.236377000
C	8.897311000	0.947674000	8.758332000
C	8.834488000	-0.439799000	8.522375000
H	3.680460000	3.524462000	3.833637000
H	3.574213000	3.854649000	5.968616000
H	-1.574349000	6.225958000	0.833699000

80

(p-H-POCOP)IrH2-IC6F5 NS-bound-b, E = -2824.457418

Ir	1.145992000	-0.473418000	-0.758362000
P	1.547775000	1.782798000	-0.417651000
P	1.467520000	-2.771870000	-0.711692000
O	3.165134000	1.841321000	0.164134000
C	2.084775000	4.344365000	-1.601553000
O	3.115091000	-2.948417000	-0.253504000
C	1.535161000	3.785635000	1.644608000
C	1.849499000	-5.194766000	-2.207879000
C	5.104156000	0.583031000	0.832990000
C	-0.728226000	-4.241529000	0.350026000
C	5.079769000	-1.865185000	0.617570000
C	0.075523000	-3.547119000	-2.991245000
C	1.566712000	-4.957453000	1.143624000
C	3.748105000	-1.789007000	0.163547000
H	6.782598000	-0.709759000	1.315603000
C	1.696524000	2.893343000	-1.942504000
C	5.741540000	-0.668212000	0.960825000
C	2.527361000	-3.000483000	-3.237097000
C	3.039751000	-0.564359000	0.089827000
C	0.351581000	2.843253000	-2.699533000
C	0.611691000	1.511513000	2.139842000
C	1.472888000	-3.708286000	-2.353602000
C	-0.701870000	3.110519000	0.658887000
C	3.772026000	0.614488000	0.374394000
C	0.686462000	-3.759783000	0.723783000
C	0.624344000	-2.769890000	1.913305000
C	0.702093000	2.622748000	1.064779000
C	2.806509000	2.264378000	-2.817603000
H	1.273387000	4.876871000	-1.072145000
H	3.003779000	4.386493000	-0.986539000
H	2.280587000	4.893041000	-2.546443000
H	2.565419000	3.465177000	1.884392000
H	1.583745000	4.655643000	0.968252000
H	1.047913000	4.119663000	2.584508000
H	2.816766000	-5.319485000	-1.684894000
H	1.073315000	-5.769186000	-1.669476000
H	1.948706000	-5.638961000	-3.220309000
H	5.629071000	1.520909000	1.062749000
H	-1.365077000	-3.423862000	-0.033313000
H	-0.699889000	-5.052146000	-0.402445000
H	-1.221115000	-4.646520000	1.258282000
H	5.585240000	-2.839132000	0.679617000
H	-0.201652000	-2.478913000	-3.078451000

H	0.093191000	-3.988581000	-4.009265000
H	-0.713257000	-4.062800000	-2.413438000
H	1.641474000	-5.729977000	0.359787000
H	2.587195000	-4.634637000	1.417845000
H	1.099217000	-5.427503000	2.034046000
H	2.272411000	-1.935971000	-3.401159000
H	3.537230000	-3.055192000	-2.788495000
H	2.554917000	-3.500812000	-4.226949000
H	-0.472416000	3.302528000	-2.122649000
H	0.454259000	3.406410000	-3.650323000
H	0.066618000	1.800784000	-2.938701000
H	1.613916000	1.119410000	2.400508000
H	0.152868000	1.934589000	3.056553000
H	-0.019073000	0.667684000	1.808803000
H	-1.289906000	2.315506000	0.163514000
H	-1.257107000	3.414249000	1.569561000
H	-0.649048000	3.988647000	-0.012400000
H	-0.061652000	-1.926665000	1.715057000
H	0.251832000	-3.310628000	2.807755000
H	1.622715000	-2.354875000	2.152570000
H	3.780936000	2.253461000	-2.293907000
H	2.556241000	1.227800000	-3.114095000
H	2.912353000	2.866707000	-3.743284000
I	-1.760950000	-0.570079000	-0.289358000
F	-4.460045000	0.619964000	4.449035000
F	-5.957454000	-1.680664000	4.417429000
F	-2.708559000	1.142647000	2.417122000
F	-5.728983000	-3.457249000	2.337387000
F	-3.983967000	-2.953827000	0.291633000
C	-4.333228000	-0.246386000	3.427047000
C	-3.436791000	0.002191000	2.369608000
C	-3.303764000	-0.890269000	1.298565000
C	-4.077945000	-2.058085000	1.302585000
C	-4.983716000	-2.336741000	2.344291000
C	-5.103061000	-1.426610000	3.413692000
H	1.959118000	-0.399846000	-2.066771000
H	-0.105149000	-0.356468000	-1.886844000

92

(p-H-POCOP)IrH2-(IC6F5)2 NS-bound-c, E = -3849.291583

Ir	3.701280000	3.427109000	3.676093000
P	1.670639000	2.368250000	3.271754000
P	5.316537000	5.101886000	3.655723000
O	0.580985000	3.667255000	2.989043000
C	0.055574000	1.543328000	1.047866000
O	4.399586000	6.543539000	3.482290000
C	-0.692200000	1.124980000	4.322368000
C	6.682428000	6.740352000	1.744839000
C	0.328742000	6.029102000	2.610997000
C	7.154359000	4.299930000	5.587787000
C	2.279925000	7.500362000	2.865114000
C	7.690436000	4.465066000	2.201990000
C	7.117478000	6.797004000	5.111981000

C	3.071754000	6.366351000	3.132771000
C	1.500337000	1.493242000	1.588117000
C	0.908976000	7.313725000	2.593950000
C	5.514726000	4.685143000	0.923188000
C	2.551735000	5.049073000	3.087509000
C	1.986052000	0.035047000	1.696340000
C	0.763602000	2.499626000	5.848826000
C	6.377940000	5.261378000	2.071176000
C	1.568107000	0.230783000	5.064525000
C	1.155480000	4.921150000	2.884100000
C	6.271506000	5.515074000	5.230957000
C	5.190438000	5.714369000	6.319674000
C	0.762175000	1.489363000	4.676456000
C	2.405110000	2.300159000	0.625959000
H	-0.649517000	0.953471000	1.658025000
H	-0.317783000	2.581526000	0.983883000
H	0.057919000	1.110658000	0.025511000
H	-1.253020000	2.001193000	3.945385000
H	-0.744449000	0.314423000	3.572402000
H	-1.199068000	0.760407000	5.240049000
H	5.755673000	7.325412000	1.605689000
H	7.294380000	7.233854000	2.518683000
H	7.256319000	6.766979000	0.794941000
H	-0.746513000	5.881886000	2.438002000
H	6.576126000	3.357275000	5.559643000
H	8.019440000	4.195089000	4.909054000
H	7.543850000	4.427389000	6.618592000
H	2.730037000	8.502620000	2.890226000
H	7.523988000	3.424179000	2.533776000
H	8.182267000	4.422330000	1.207899000
H	8.396692000	4.950499000	2.901214000
H	7.965927000	6.670604000	4.414497000
H	6.506242000	7.659036000	4.783606000
H	7.540875000	7.037063000	6.109503000
H	5.351759000	3.598989000	1.037906000
H	4.525801000	5.178886000	0.862823000
H	6.043501000	4.850285000	-0.038180000
H	1.257821000	-0.595026000	2.240191000
H	2.099935000	-0.384348000	0.676138000
H	2.969408000	-0.041448000	2.196929000
H	0.241173000	3.437088000	5.578217000
H	0.241715000	2.041615000	6.713573000
H	1.791787000	2.744371000	6.171949000
H	2.651963000	0.443225000	5.127396000
H	1.239966000	-0.116137000	6.065068000
H	1.415159000	-0.596023000	4.348373000
H	4.589066000	4.798315000	6.468758000
H	5.692797000	5.949897000	7.280279000
H	4.508688000	6.548447000	6.066889000
H	2.113035000	3.367253000	0.588517000
H	3.468340000	2.238983000	0.916512000
H	2.312648000	1.876244000	-0.394746000

I	4.550895000	1.676511000	7.133671000
F	1.729453000	-0.282393000	11.357917000
F	3.884085000	-0.281178000	13.059720000
F	2.016754000	0.570054000	8.781684000
F	6.339813000	0.574208000	12.179643000
F	6.650453000	1.431248000	9.605843000
C	2.927336000	0.136666000	10.920588000
C	3.090381000	0.579686000	9.594229000
C	4.343912000	1.019652000	9.127081000
C	5.438245000	1.016274000	10.012012000
C	5.291365000	0.575920000	11.341683000
C	4.031696000	0.137649000	11.795711000
H	4.461800000	2.241123000	4.633286000
H	3.201759000	3.896037000	5.059096000
C	6.741423000	0.554569000	1.090444000
C	6.097613000	0.113616000	-0.073593000
C	8.139989000	0.657656000	1.085153000
C	6.826742000	-0.237036000	-1.226294000
C	8.896321000	0.314309000	-0.051844000
C	8.231671000	-0.127999000	-1.213393000
I	5.590152000	1.276178000	2.836182000
F	4.750444000	0.005679000	-0.121371000
F	6.201899000	-0.666515000	-2.336942000
F	8.938430000	-0.453016000	-2.306304000
F	10.237642000	0.409010000	-0.047302000
F	8.804118000	1.086423000	2.182374000
H	0.275854000	8.189336000	2.384991000

80

(p-H-POCOP)IrH(IC6F5)H NS-bound-d, E = -2824.459961

Ir	3.514189000	3.971147000	4.156844000
P	1.699576000	2.671501000	3.553440000
P	4.837738000	5.845506000	4.502309000
O	0.899822000	3.632201000	2.381499000
C	0.742818000	0.817624000	1.572008000
O	4.086257000	7.052831000	3.547614000
C	-0.916802000	1.720917000	4.331660000
C	7.076071000	7.424004000	3.578070000
C	0.615205000	5.861298000	1.511578000
C	5.018016000	5.608802000	7.301012000
C	2.233996000	7.602942000	2.109672000
C	7.557019000	5.196510000	4.718934000
C	5.595385000	7.899707000	6.404664000
C	2.922942000	6.663430000	2.902122000
H	0.527162000	7.913935000	0.799084000
C	1.907653000	1.062274000	2.554786000
C	1.075827000	7.190344000	1.420624000
C	6.542873000	5.288872000	2.402582000
C	2.480176000	5.328180000	3.044108000
C	2.059450000	-0.115334000	3.538824000
C	-0.159154000	3.964220000	5.143291000
C	6.599776000	5.969563000	3.789352000
C	0.873270000	1.888015000	6.129146000

C	1.329237000	4.950031000	2.314019000
C	4.693871000	6.664343000	6.222935000
C	3.217987000	7.106606000	6.360889000
C	0.299383000	2.516984000	4.842379000
C	3.209214000	1.213463000	1.736034000
H	-0.218544000	0.635816000	2.080625000
H	0.617920000	1.672862000	0.882632000
H	0.979364000	-0.083249000	0.967639000
H	-1.300239000	2.137009000	3.380626000
H	-0.696604000	0.646751000	4.191737000
H	-1.729744000	1.795171000	5.084149000
H	6.314584000	8.019825000	3.040836000
H	7.330599000	7.937185000	4.519273000
H	7.994096000	7.401133000	2.954507000
H	-0.280274000	5.523280000	0.971164000
H	4.279949000	4.784321000	7.275003000
H	6.034779000	5.184794000	7.191494000
H	4.957120000	6.083119000	8.302580000
H	2.612634000	8.632182000	2.035222000
H	7.206116000	4.161678000	4.894659000
H	8.557338000	5.133818000	4.241796000
H	7.684000000	5.699249000	5.696819000
H	6.668416000	7.635704000	6.440622000
H	5.433186000	8.645913000	5.604641000
H	5.340806000	8.381221000	7.372149000
H	6.232188000	4.233613000	2.474916000
H	5.830441000	5.808358000	1.732123000
H	7.552075000	5.339962000	1.943572000
H	1.104413000	-0.380358000	4.028020000
H	2.414013000	-1.010828000	2.987507000
H	2.798975000	0.101198000	4.329869000
H	-0.665927000	4.418262000	4.271660000
H	-0.877923000	3.936742000	5.988213000
H	0.695393000	4.605714000	5.430382000
H	1.708371000	2.499655000	6.520830000
H	0.076735000	1.857555000	6.901645000
H	1.234451000	0.854122000	5.977423000
H	2.526619000	6.267774000	6.152887000
H	3.047868000	7.453043000	7.401361000
H	2.982775000	7.938825000	5.671819000
H	3.161198000	2.085714000	1.056659000
H	4.088961000	1.350511000	2.388140000
H	3.352748000	0.297769000	1.125230000
I	4.837578000	2.251636000	5.736288000
F	5.226241000	-3.020869000	4.402075000
F	6.944142000	-2.574764000	2.307917000
F	4.375986000	-0.933055000	5.941578000
F	7.813176000	-0.032382000	1.749308000
F	6.979093000	2.064119000	3.278163000
C	5.649765000	-1.776534000	4.136709000
C	5.226497000	-0.691468000	4.926193000
C	5.644595000	0.619509000	4.637426000

C	6.537707000	0.831361000	3.572927000
C	6.971438000	-0.241791000	2.771219000
C	6.530641000	-1.548680000	3.060487000
H	4.264036000	3.651036000	2.718834000
H	2.713253000	4.317700000	5.596351000

80

(p-H-POCOP)IrH(IC6F5)H NS-bound-e, E = -2824.457898

Ir	3.834630000	4.127066000	3.053875000
P	1.996368000	2.761780000	2.691682000
P	4.811153000	6.045350000	3.919978000
O	0.939966000	3.775072000	1.755726000
C	0.664493000	0.950932000	0.887183000
O	3.767961000	7.322302000	3.384220000
C	-0.401293000	1.713885000	3.953161000
C	6.488267000	8.351986000	3.448561000
C	0.185044000	6.074324000	1.554843000
C	5.301871000	5.089240000	6.556292000
C	1.621701000	7.884488000	2.413753000
C	7.601246000	6.251685000	4.388353000
C	5.022189000	7.610971000	6.339997000
C	2.571684000	6.916826000	2.806367000
H	-0.302253000	8.189067000	1.454167000
C	1.977580000	1.137216000	1.675767000
C	0.435686000	7.444259000	1.788809000
C	6.848996000	6.338792000	2.021527000
C	2.349347000	5.539267000	2.604634000
C	2.253585000	-0.041453000	2.637299000
C	0.410549000	3.930598000	4.694048000
C	6.510657000	6.808571000	3.448701000
C	1.695538000	1.877613000	5.382212000
C	1.153911000	5.139563000	1.968491000
C	4.570285000	6.231681000	5.818370000
C	3.057932000	6.105524000	6.102192000
C	0.878308000	2.526203000	4.244285000
C	3.149208000	1.168784000	0.673976000
H	-0.218597000	0.832443000	1.536374000
H	0.490158000	1.807594000	0.210569000
H	0.754010000	0.033877000	0.267373000
H	-0.968079000	2.154311000	3.111194000
H	-0.212399000	0.647398000	3.739993000
H	-1.050793000	1.757976000	4.852449000
H	5.714262000	8.733147000	2.757350000
H	6.306768000	8.785718000	4.445473000
H	7.477460000	8.714854000	3.097602000
H	-0.735589000	5.738487000	1.057044000
H	5.007772000	4.101080000	6.152051000
H	6.400828000	5.172789000	6.496062000
H	5.020456000	5.119387000	7.629857000
H	1.819922000	8.953821000	2.570945000
H	7.574894000	5.144105000	4.427593000
H	8.593716000	6.547180000	3.988898000
H	7.529314000	6.644680000	5.417666000

H	6.113704000	7.763357000	6.275702000
H	4.512098000	8.429653000	5.797604000
H	4.743096000	7.689908000	7.411474000
H	6.915965000	5.235730000	1.967529000
H	6.102699000	6.687152000	1.288106000
H	7.835725000	6.757621000	1.733122000
H	1.427464000	-0.241504000	3.340197000
H	2.401115000	-0.957814000	2.029410000
H	3.183521000	0.124115000	3.217728000
H	-0.336117000	4.351965000	3.996995000
H	-0.064734000	3.836797000	5.692191000
H	1.242093000	4.647339000	4.765602000
H	2.581113000	2.489177000	5.641006000
H	1.055597000	1.805950000	6.286370000
H	2.046241000	0.859981000	5.135707000
H	2.690589000	5.091438000	5.876300000
H	2.885308000	6.294465000	7.181732000
H	2.458526000	6.833125000	5.524445000
H	3.004107000	1.934247000	-0.103556000
H	4.112567000	1.355208000	1.185907000
H	3.206267000	0.180638000	0.171744000
I	4.576823000	4.324652000	0.350439000
F	-0.081278000	5.054128000	-2.388458000
F	-0.072726000	7.777038000	-2.026406000
F	1.962132000	3.542203000	-1.431352000
F	2.013819000	8.989610000	-0.727176000
F	4.088060000	7.506556000	0.215489000
C	0.947691000	5.644896000	-1.760176000
C	2.009269000	4.874479000	-1.252927000
C	3.073332000	5.482069000	-0.562282000
C	3.080355000	6.881202000	-0.414364000
C	2.023029000	7.661929000	-0.911716000
C	0.954380000	7.040610000	-1.583121000
H	5.038417000	2.993266000	3.319866000
H	3.547450000	3.840816000	4.562010000

74

(p-MeOOC-POCOP)IrH2 S tol, E = -2027.284304

C	-0.351751000	1.197573000	-3.616989000
C	-0.229320000	1.189450000	-2.215661000
C	-0.172221000	-0.023787000	-1.482066000
C	-0.240645000	-1.245804000	-2.199206000
C	-0.361505000	-1.275335000	-3.601736000
C	-0.416475000	-0.042432000	-4.297640000
H	-0.396914000	2.135204000	-4.185369000
H	-0.412026000	-2.228113000	-4.141599000
O	-0.187438000	-2.435064000	-1.506235000
O	-0.161885000	2.387078000	-1.539304000
P	-0.029280000	-2.267353000	0.222427000
P	-0.018154000	2.241703000	0.192140000
Ir	0.006023000	-0.010704000	0.591128000
H	0.921545000	0.015914000	1.909394000
H	-0.639186000	-0.016429000	2.064358000

C	1.601313000	-3.200210000	0.461748000
C	-1.579548000	-3.228321000	0.734585000
C	1.600842000	3.199269000	0.415696000
C	-1.581350000	3.184080000	0.696449000
C	1.985590000	-3.135669000	1.955549000
H	3.004325000	-3.557843000	2.082715000
H	1.994184000	-2.090506000	2.320569000
H	1.297580000	-3.722438000	2.592210000
C	-1.517010000	-3.485943000	2.253693000
H	-1.274531000	-2.560699000	2.813739000
H	-2.506834000	-3.847950000	2.601958000
H	-0.769295000	-4.260922000	2.509572000
C	2.630240000	-2.390100000	-0.365071000
H	2.405774000	-2.434701000	-1.447627000
H	2.638889000	-1.325551000	-0.056532000
H	3.640987000	-2.817545000	-0.199718000
C	1.563100000	-4.656040000	-0.035927000
H	2.595922000	-5.063799000	-0.035454000
H	0.952943000	-5.301437000	0.623100000
H	1.169576000	-4.723568000	-1.067949000
C	-1.786621000	-4.540731000	-0.046007000
H	-1.050235000	-5.315976000	0.228551000
H	-2.795507000	-4.939266000	0.191066000
H	-1.731330000	-4.370733000	-1.137447000
C	-2.744688000	-2.259019000	0.414080000
H	-2.634996000	-1.303967000	0.964144000
H	-2.789548000	-2.026574000	-0.667702000
H	-3.703153000	-2.736704000	0.706616000
C	1.814468000	3.457866000	1.920842000
H	1.676568000	2.533171000	2.516114000
H	2.851243000	3.819278000	2.083771000
H	1.126232000	4.233580000	2.307512000
C	-1.693218000	3.156450000	2.235747000
H	-2.675514000	3.580156000	2.532009000
H	-1.630419000	2.120747000	2.622082000
H	-0.906419000	3.760588000	2.724698000
C	2.687142000	2.227132000	-0.108671000
H	2.676101000	1.272467000	0.452658000
H	2.536489000	1.994662000	-1.180786000
H	3.683422000	2.703020000	0.006685000
C	1.665937000	4.510360000	-0.391221000
H	0.995281000	5.288380000	0.013185000
H	2.702592000	4.905192000	-0.343668000
H	1.410897000	4.340257000	-1.453878000
C	-1.637485000	4.626873000	0.163409000
H	-2.656609000	5.033551000	0.333611000
H	-0.925569000	5.289935000	0.689183000
H	-1.430099000	4.667935000	-0.922730000
C	-2.738275000	2.355146000	0.085253000
H	-2.708099000	2.371047000	-1.020727000
H	-2.690254000	1.298967000	0.418178000
H	-3.705023000	2.789318000	0.414710000

C	-0.545571000	0.008182000	-5.790422000
O	-0.597407000	1.037883000	-6.453359000
O	-0.600586000	-1.232761000	-6.348296000
C	-0.724476000	-1.265103000	-7.781865000
H	-1.652395000	-0.753559000	-8.108187000
H	0.140947000	-0.766298000	-8.262949000
H	-0.755922000	-2.333841000	-8.059170000

74

(p-MeOOC-POCOP)IrH2 NS tol, E = -2027.285792

Ir	0.809718000	-0.908441000	-0.013975000
P	2.288061000	0.839006000	0.028936000
P	-1.170816000	-2.056040000	0.009170000
O	-2.890848000	5.214892000	0.083788000
O	-4.574447000	3.686257000	0.068962000
O	1.299797000	2.261357000	0.057782000
C	4.210671000	2.401959000	1.461341000
O	-2.397429000	-0.832062000	0.023805000
C	3.560743000	2.611018000	-1.862644000
C	-3.061926000	-3.666635000	1.431238000
C	-0.951036000	3.116698000	0.059457000
C	-0.933266000	-4.282691000	-1.678821000
C	-2.845667000	1.533351000	0.041823000
C	-0.584862000	-4.081506000	1.835170000
C	-3.124954000	-3.003473000	-1.894664000
C	-1.925551000	0.471182000	0.030004000
C	-3.256963000	4.043700000	0.070889000
C	3.327363000	1.145525000	1.575694000
C	-2.345629000	2.860856000	0.056316000
C	-1.683534000	-1.978993000	2.700579000
C	-0.523611000	0.684556000	0.029536000
C	4.181523000	-0.112865000	1.842341000
C	-5.512022000	4.776370000	0.084347000
C	2.159890000	0.678049000	-2.666827000
C	-1.670027000	-3.019504000	1.554951000
C	4.435553000	0.226856000	-1.666032000
C	-0.065908000	2.026952000	0.047421000
C	-1.611635000	-2.899394000	-1.628228000
C	-0.969805000	-1.965604000	-2.685134000
C	3.193243000	1.137369000	-1.607404000
C	2.307148000	1.332315000	2.724382000
H	5.023429000	2.269630000	0.722642000
H	3.615185000	3.292592000	1.183714000
H	4.684284000	2.600402000	2.445657000
H	2.677284000	3.267447000	-1.756081000
H	4.350485000	2.972634000	-1.181120000
H	3.943052000	2.707624000	-2.900743000
H	-3.830686000	-2.923243000	1.146032000
H	-3.070486000	-4.491333000	0.694127000
H	-3.348815000	-4.095542000	2.414258000
H	-0.587258000	4.152198000	0.074176000
H	0.141120000	-4.217208000	-1.414421000
H	-1.422550000	-5.006309000	-0.999068000

H	-1.009369000	-4.687262000	-2.709747000
H	-3.925186000	1.340822000	0.043155000
H	0.417426000	-3.618117000	1.912557000
H	-0.815382000	-4.585404000	2.796938000
H	-0.544210000	-4.857958000	1.048921000
H	-3.624293000	-3.716425000	-1.215403000
H	-3.617224000	-2.018427000	-1.793633000
H	-3.279456000	-3.364879000	-2.933264000
H	-0.710108000	-1.459270000	2.792037000
H	-2.469106000	-1.216122000	2.545454000
H	-1.887036000	-2.504118000	3.656617000
H	4.939383000	-0.279900000	1.054625000
H	4.717295000	0.014659000	2.806056000
H	3.549182000	-1.018988000	1.908640000
H	-6.515379000	4.313997000	0.076890000
H	-5.380795000	5.397743000	0.993391000
H	-5.379392000	5.424486000	-0.805576000
H	1.225816000	1.270606000	-2.613385000
H	2.589188000	0.800355000	-3.683004000
H	1.908165000	-0.396186000	-2.535666000
H	4.181948000	-0.821392000	-1.410405000
H	4.847646000	0.239574000	-2.696715000
H	5.233297000	0.575473000	-0.982712000
H	0.130631000	-1.905405000	-2.544592000
H	-1.157127000	-2.369504000	-3.701800000
H	-1.390243000	-0.942093000	-2.637912000
H	1.693955000	2.240824000	2.577059000
H	1.625068000	0.464233000	2.809602000
H	2.861065000	1.432672000	3.680526000
H	1.027747000	-1.179309000	1.485534000
H	1.883806000	-2.186913000	-0.113098000

86

(p-MeOOC-POCOP)IrH2-IC6F5 NS-bound-a1, E = -3052.123147

Ir	3.069198000	3.933005000	4.215248000
P	1.139869000	2.891621000	3.539726000
P	4.698917000	5.547641000	4.501817000
O	-0.684982000	8.525179000	0.254411000
O	1.153248000	9.771355000	0.744818000
O	0.323755000	4.076419000	2.587061000
C	-0.244063000	1.029521000	1.890229000
O	4.138201000	6.867396000	3.534618000
C	-1.573376000	2.774635000	4.506730000
C	7.011107000	6.775175000	3.357237000
C	0.390095000	6.319117000	1.715489000
C	5.049835000	5.309931000	7.274273000
C	2.340489000	7.749384000	2.202892000
C	7.383327000	4.741950000	4.825415000
C	5.488438000	7.631227000	6.323158000
C	2.906919000	6.688798000	2.930078000
C	0.407991000	8.630585000	0.801441000
C	1.173831000	1.469587000	2.302286000
C	1.075810000	7.553308000	1.592893000

C	6.321050000	4.501160000	2.539405000
C	2.255432000	5.437959000	3.070987000
C	1.968760000	0.295111000	2.908092000
C	0.574336000	10.854248000	-0.004703000
C	0.220977000	3.912156000	5.864265000
C	6.451160000	5.402704000	3.789999000
C	0.195314000	1.390525000	5.710457000
C	0.988361000	5.285412000	2.452053000
C	4.635925000	6.355021000	6.215098000
C	3.142455000	6.711536000	6.414425000
C	-0.101824000	2.700223000	4.953652000
C	1.929245000	2.020783000	1.069113000
H	-0.776334000	0.527925000	2.719988000
H	-0.850867000	1.886011000	1.539618000
H	-0.164463000	0.300881000	1.056738000
H	-1.767139000	3.689376000	3.916308000
H	-1.874157000	1.898736000	3.905441000
H	-2.218209000	2.800130000	5.410195000
H	6.372414000	7.248877000	2.589992000
H	7.120123000	7.476666000	4.202665000
H	8.018970000	6.612013000	2.921740000
H	-0.589874000	6.189150000	1.238638000
H	4.470553000	4.372599000	7.160910000
H	6.124225000	5.059282000	7.221612000
H	4.848562000	5.724889000	8.283902000
H	2.866147000	8.707270000	2.111174000
H	6.952276000	3.810509000	5.237915000
H	8.335781000	4.469353000	4.326775000
H	7.622434000	5.429845000	5.658030000
H	6.571303000	7.410504000	6.279193000
H	5.236867000	8.353111000	5.522938000
H	5.287469000	8.118287000	7.300540000
H	6.038908000	3.467811000	2.812386000
H	5.573043000	4.900526000	1.826288000
H	7.302748000	4.461755000	2.023967000
H	1.459085000	-0.156563000	3.778412000
H	2.081772000	-0.493981000	2.136110000
H	2.980118000	0.611677000	3.226189000
H	1.302672000	11.683125000	0.048259000
H	0.403622000	10.557045000	-1.059136000
H	-0.395401000	11.164058000	0.434748000
H	0.079858000	4.875143000	5.336129000
H	-0.448499000	3.903453000	6.749196000
H	1.266213000	3.855864000	6.242436000
H	1.272507000	1.294806000	5.953751000
H	-0.372359000	1.387131000	6.664149000
H	-0.118585000	0.501661000	5.131230000
H	2.495141000	5.813517000	6.351332000
H	3.006845000	7.147250000	7.425598000
H	2.787360000	7.446042000	5.667667000
H	1.390885000	2.867906000	0.604476000
H	2.950241000	2.357855000	1.333500000

H 2.020700000 1.209182000 0.318472000
I 5.366021000 1.297982000 4.950515000
F 8.044853000 -3.454503000 5.775968000
F 10.312169000 -2.622798000 4.469205000
F 5.897154000 -1.779800000 5.994188000
F 10.435714000 -0.105305000 3.378263000
F 8.300066000 1.583779000 3.583397000
C 8.096031000 -2.220380000 5.245196000
C 6.997597000 -1.344613000 5.348026000
C 7.041136000 -0.052955000 4.798065000
C 8.210148000 0.352701000 4.131813000
C 9.321472000 -0.504229000 4.016481000
C 9.260266000 -1.796594000 4.574422000
H 3.848372000 3.334440000 3.033958000
H 3.564985000 2.738006000 5.318884000
86
(p-MeOOC-POCOP)IrH2-IC6F5 NS-bound-a2, E = -3052.122851
Ir 2.614023000 4.217238000 4.584498000
P 1.294849000 2.336730000 4.497411000
P 3.455590000 6.297076000 4.123383000
O -0.018332000 2.790442000 3.463252000
C 0.855845000 -0.319093000 3.541952000
O 2.340186000 6.972998000 2.990683000
C -1.031377000 1.295153000 5.847758000
C 5.457143000 7.909149000 2.876187000
C -0.928193000 4.497793000 2.030685000
C 4.437962000 7.352949000 6.520298000
C 0.280413000 6.637262000 1.789193000
C 6.168015000 5.699866000 3.920627000
C 3.118874000 8.994672000 5.081965000
C 1.265686000 6.158998000 2.666782000
C 1.905964000 0.807243000 3.571717000
C -0.816383000 5.796867000 1.473189000
C 4.793554000 5.725136000 1.801047000
C 1.194302000 4.862738000 3.239763000
C 3.219045000 0.326160000 4.222154000
C 0.097179000 3.389800000 6.679288000
C 5.060531000 6.445256000 3.144612000
C 1.222826000 1.154043000 7.029725000
C 0.077759000 4.054278000 2.905747000
C 3.260383000 7.531594000 5.541103000
C 1.943275000 7.077889000 6.220686000
C 0.328646000 1.978541000 6.082520000
C 2.191777000 1.287020000 2.128163000
H 0.703186000 -0.767563000 4.541487000
H -0.116998000 0.041050000 3.156280000
H 1.214186000 -1.124420000 2.867211000
H -1.642409000 1.859404000 5.118889000
H -0.924089000 0.257347000 5.487283000
H -1.582292000 1.260826000 6.811157000
H 4.632341000 8.473717000 2.400769000
H 5.761880000 8.430737000 3.802700000

H	6.325026000	7.924563000	2.184342000
H	-1.780130000	3.852810000	1.784738000
H	4.586190000	6.287851000	6.789499000
H	5.383824000	7.747534000	6.102925000
H	4.222348000	7.915216000	7.452572000
H	0.343872000	7.640385000	1.348309000
H	5.884946000	4.648386000	4.118002000
H	7.095868000	5.703868000	3.311897000
H	6.398690000	6.178182000	4.890288000
H	4.056746000	9.395988000	4.659868000
H	2.318614000	9.100920000	4.325975000
H	2.851889000	9.618561000	5.960661000
H	4.467005000	4.678340000	1.955187000
H	4.018852000	6.246122000	1.208221000
H	5.734682000	5.709122000	1.213961000
H	3.085160000	-0.004044000	5.268072000
H	3.611099000	-0.535808000	3.643359000
H	3.986048000	1.122645000	4.217764000
H	-0.478214000	4.037820000	5.989067000
H	-0.474147000	3.303737000	7.626732000
H	1.063909000	3.884233000	6.913568000
H	2.230036000	1.604248000	7.136334000
H	0.754227000	1.122777000	8.035336000
H	1.338990000	0.110174000	6.681314000
H	2.030850000	6.039487000	6.606584000
H	1.726607000	7.737882000	7.085977000
H	1.082660000	7.127558000	5.525450000
H	1.265514000	1.601687000	1.612448000
H	2.903241000	2.135393000	2.111598000
H	2.642576000	0.448144000	1.559143000
I	5.349187000	2.520129000	6.674420000
F	7.648870000	-2.329409000	7.792135000
F	9.700988000	-1.259247000	9.270397000
F	5.769859000	-0.695161000	6.671647000
F	9.875377000	1.457186000	9.631514000
F	8.006437000	3.109857000	8.519374000
C	7.726339000	-0.998317000	7.963977000
C	6.766047000	-0.140989000	7.393152000
C	6.838128000	1.252300000	7.566889000
C	7.895510000	1.780807000	8.326282000
C	8.867244000	0.942300000	8.906560000
C	8.780382000	-0.451729000	8.722473000
H	3.617752000	3.572645000	3.612461000
H	3.705710000	3.823922000	5.811662000
C	-1.844409000	6.340697000	0.534516000
O	-1.807082000	7.454099000	0.020912000
O	-2.853619000	5.454146000	0.298861000
C	-3.881790000	5.908758000	-0.598766000
H	-4.375907000	6.819471000	-0.203923000
H	-3.459020000	6.142743000	-1.596737000
H	-4.607612000	5.079359000	-0.673633000

74

(m-bis-CF3-POCOP)IrH2 S tol, E = -2473.009161

C	-0.334252000	1.216761000	-3.591844000
C	-0.205102000	1.204237000	-2.177083000
C	-0.159992000	-0.010758000	-1.455394000
C	-0.231994000	-1.228837000	-2.169679000
C	-0.334503000	-1.247291000	-3.586588000
C	-0.390196000	-0.016709000	-4.270083000
H	-0.479618000	-0.019012000	-5.363232000
O	-0.199659000	-2.408203000	-1.491744000
O	-0.125914000	2.386535000	-1.508034000
P	-0.031724000	-2.262363000	0.246472000
P	-0.011515000	2.248240000	0.234990000
Ir	0.008678000	-0.006273000	0.621533000
H	0.914481000	0.009581000	1.944906000
H	-0.648564000	-0.014568000	2.086821000
C	1.598972000	-3.203507000	0.431378000
C	-1.585373000	-3.228919000	0.730010000
C	1.598414000	3.217372000	0.458799000
C	-1.592546000	3.189242000	0.675707000
C	2.014738000	-3.161454000	1.917309000
H	3.026761000	-3.605647000	2.018942000
H	2.053070000	-2.120450000	2.292530000
H	1.327545000	-3.740443000	2.562297000
C	-1.511352000	-3.539863000	2.238644000
H	-1.268601000	-2.634701000	2.830803000
H	-2.497695000	-3.917127000	2.579974000
H	-0.759466000	-4.321171000	2.461155000
C	2.611514000	-2.388909000	-0.412069000
H	2.370079000	-2.438956000	-1.490732000
H	2.625210000	-1.324119000	-0.104112000
H	3.625140000	-2.814745000	-0.262117000
C	1.538009000	-4.650057000	-0.090869000
H	2.567143000	-5.066227000	-0.110425000
H	0.930223000	-5.301352000	0.564447000
H	1.132789000	-4.692484000	-1.119680000
C	-1.805267000	-4.510370000	-0.098606000
H	-1.068815000	-5.297938000	0.137027000
H	-2.812356000	-4.912800000	0.138877000
H	-1.764796000	-4.300190000	-1.183388000
C	-2.746238000	-2.242331000	0.448849000
H	-2.629967000	-1.305419000	1.027817000
H	-2.797809000	-1.977993000	-0.625370000
H	-3.704833000	-2.724868000	0.732215000
C	1.766850000	3.535020000	1.958443000
H	1.622782000	2.632672000	2.586070000
H	2.794720000	3.914132000	2.135764000
H	1.059365000	4.317050000	2.294734000
C	-1.760905000	3.159219000	2.209790000
H	-2.746163000	3.598383000	2.470968000
H	-1.729913000	2.121579000	2.595010000
H	-0.982340000	3.748431000	2.729509000
C	2.700360000	2.230177000	-0.000731000

H	2.679609000	1.295961000	0.593526000
H	2.578984000	1.960736000	-1.068058000
H	3.691340000	2.714700000	0.122689000
C	1.680579000	4.495225000	-0.399731000
H	0.990000000	5.282962000	-0.052332000
H	2.711969000	4.899790000	-0.328235000
H	1.467280000	4.280207000	-1.463133000
C	-1.622753000	4.630964000	0.137594000
H	-2.642674000	5.044770000	0.283266000
H	-0.918182000	5.290072000	0.678042000
H	-1.391766000	4.664067000	-0.944072000
C	-2.726380000	2.365392000	0.015272000
H	-2.663139000	2.404881000	-1.088693000
H	-2.687387000	1.303647000	0.331469000
H	-3.703443000	2.791666000	0.323291000
C	-0.375467000	-2.561251000	-4.324911000
F	-0.448893000	-2.392630000	-5.674171000
F	0.736314000	-3.321261000	-4.080808000
F	-1.451000000	-3.326000000	-3.964029000
C	-0.414450000	2.527694000	-4.332319000
F	0.705162000	3.293670000	-4.154293000
F	-0.561500000	2.353697000	-5.674826000
F	-1.472016000	3.288913000	-3.913462000

74

(m-bis-CF3-POCOP)IrH2 NS tol, E = -2473.010142

C	-0.116317000	1.231529000	-3.611469000
C	-0.035188000	1.212813000	-2.194390000
C	0.004853000	0.000864000	-1.469306000
C	-0.022129000	-1.210320000	-2.195747000
C	-0.103947000	-1.228409000	-3.612766000
C	-0.149375000	0.001726000	-4.299404000
H	-0.217649000	0.001952000	-5.393662000
O	0.013856000	-2.395286000	-1.513317000
O	-0.011423000	2.397027000	-1.510780000
P	0.019652000	-2.254084000	0.223857000
P	0.008304000	2.254460000	0.227577000
Ir	0.071226000	0.001029000	0.616115000
H	0.231195000	-0.022551000	2.271915000
H	-1.385539000	-0.000528000	1.118169000
C	1.635343000	-3.177495000	0.561432000
C	-1.558222000	-3.224285000	0.586398000
C	1.627584000	3.176748000	0.552458000
C	-1.566575000	3.225674000	0.598914000
C	1.679847000	-3.563660000	2.053230000
H	2.701010000	-3.917130000	2.306517000
H	1.445617000	-2.697706000	2.704389000
H	0.974448000	-4.384048000	2.286500000
C	-1.855609000	-3.114723000	2.097196000
H	-1.906013000	-2.057402000	2.421511000
H	-2.835568000	-3.592006000	2.305847000
H	-1.092698000	-3.627782000	2.712092000
C	2.717162000	-2.108370000	0.262988000

H	2.678247000	-1.771638000	-0.791487000
H	2.597857000	-1.223843000	0.924367000
H	3.723141000	-2.538028000	0.449914000
C	1.865259000	-4.399205000	-0.348947000
H	2.899797000	-4.768888000	-0.188035000
H	1.175141000	-5.230060000	-0.120517000
H	1.753481000	-4.132743000	-1.416094000
C	-1.464288000	-4.695075000	0.139509000
H	-0.751150000	-5.271248000	0.758272000
H	-2.462134000	-5.167962000	0.253390000
H	-1.167789000	-4.775152000	-0.923728000
C	-2.670414000	-2.512010000	-0.221974000
H	-2.727163000	-1.433197000	0.020729000
H	-2.506256000	-2.616208000	-1.310684000
H	-3.645228000	-2.977324000	0.031112000
C	1.688994000	3.552511000	2.046328000
H	1.459082000	2.682740000	2.693970000
H	2.713655000	3.901768000	2.291240000
H	0.988266000	4.373192000	2.292360000
C	-1.846788000	3.130487000	2.113874000
H	-2.829241000	3.600213000	2.327897000
H	-1.883145000	2.076354000	2.450594000
H	-1.082600000	3.658222000	2.714556000
C	2.705391000	2.109066000	0.234642000
H	2.587180000	1.215978000	0.885085000
H	2.660651000	1.786267000	-0.823847000
H	3.713264000	2.533544000	0.423165000
C	1.847905000	4.404690000	-0.351797000
H	1.171305000	5.239686000	-0.098939000
H	2.889383000	4.763389000	-0.212081000
H	1.708996000	4.149529000	-1.418518000
C	-1.479057000	4.691913000	0.135860000
H	-2.477580000	5.163435000	0.249368000
H	-0.764528000	5.276139000	0.745325000
H	-1.187373000	4.761653000	-0.929391000
C	-2.686969000	2.504145000	-0.189674000
H	-2.531560000	2.591832000	-1.281173000
H	-2.744533000	1.429276000	0.069640000
H	-3.658594000	2.975579000	0.064399000
C	-0.160455000	-2.541498000	-4.350439000
F	-0.255760000	-2.374043000	-5.698595000
F	0.949289000	-3.311357000	-4.127682000
F	-1.235520000	-3.300483000	-3.970736000
C	-0.189032000	2.544725000	-4.347567000
F	0.908939000	3.330009000	-4.120516000
F	-0.277973000	2.377878000	-5.696249000
F	-1.275937000	3.287696000	-3.969868000

86

(m-bis-CF3-POCOP)IrH2-IC6F5 NS-bound-a1, E = -3497.846476

Ir	3.015458000	4.036956000	4.138399000
P	1.088901000	3.003531000	3.449601000
P	4.644804000	5.646063000	4.438260000

O	0.276228000	4.215842000	2.511147000
C	-0.331834000	1.223785000	1.748252000
O	4.091086000	6.962474000	3.446354000
C	-1.633244000	2.926317000	4.396429000
C	6.965198000	6.878387000	3.310510000
C	0.355136000	6.448125000	1.619045000
C	4.934831000	5.464701000	7.213511000
C	2.293438000	7.870345000	2.133030000
C	7.311385000	4.828524000	4.771731000
C	5.424740000	7.762709000	6.222701000
C	2.869724000	6.793645000	2.855436000
H	0.589627000	8.495810000	0.948065000
C	1.095794000	1.623609000	2.167905000
C	1.041843000	7.674391000	1.516061000
C	6.268207000	4.612644000	2.473941000
C	2.207426000	5.554054000	2.990997000
C	1.875607000	0.421527000	2.737774000
C	0.175632000	4.015552000	5.775527000
C	6.396387000	5.505472000	3.731211000
C	0.108638000	1.494313000	5.589460000
C	0.946082000	5.402812000	2.373990000
C	4.556276000	6.495946000	6.128016000
C	3.066149000	6.886420000	6.288204000
C	-0.165360000	2.819302000	4.850378000
C	1.850132000	2.205851000	0.947689000
H	-0.873514000	0.710835000	2.564737000
H	-0.918657000	2.102980000	1.420441000
H	-0.266904000	0.516444000	0.895477000
H	-1.806753000	3.843771000	3.804463000
H	-1.949813000	2.056145000	3.794982000
H	-2.279656000	2.964146000	5.298228000
H	6.316671000	7.379016000	2.569345000
H	7.105541000	7.560500000	4.166666000
H	7.960291000	6.708102000	2.849399000
H	4.330617000	4.540967000	7.118723000
H	6.002426000	5.182911000	7.171655000
H	4.740936000	5.908223000	8.212210000
H	6.865487000	3.897773000	5.170640000
H	8.266736000	4.551366000	4.281766000
H	7.545653000	5.506829000	5.613616000
H	6.505217000	7.527514000	6.207364000
H	5.195882000	8.468552000	5.401740000
H	5.209246000	8.273707000	7.184540000
H	5.978206000	3.578859000	2.737290000
H	5.529361000	5.022402000	1.757268000
H	7.253692000	4.570458000	1.966262000
H	1.362502000	-0.044222000	3.599058000
H	1.972755000	-0.349142000	1.945447000
H	2.893233000	0.714347000	3.059252000
H	0.035080000	4.986561000	5.262190000
H	-0.486100000	3.999349000	6.666009000
H	1.223896000	3.946671000	6.145136000

H	1.181877000	1.381647000	5.843254000
H	-0.469959000	1.482144000	6.536331000
H	-0.208907000	0.618921000	4.991865000
H	2.395880000	6.007644000	6.199593000
H	2.915364000	7.318473000	7.298621000
H	2.755716000	7.638528000	5.539228000
H	1.305679000	3.059739000	0.502891000
H	2.870932000	2.539716000	1.217007000
H	1.942001000	1.411380000	0.179100000
I	5.304141000	1.339661000	4.802386000
F	7.801003000	-3.543080000	5.308154000
F	10.160960000	-2.661379000	4.215862000
F	5.689085000	-1.830720000	5.570963000
F	10.413065000	-0.055439000	3.386664000
F	8.314706000	1.672544000	3.639815000
C	7.915477000	-2.266097000	4.905089000
C	6.835680000	-1.370703000	5.033040000
C	6.945606000	-0.033314000	4.616316000
C	8.161647000	0.398496000	4.058216000
C	9.254439000	-0.478746000	3.920560000
C	9.127221000	-1.816467000	4.344328000
H	3.806479000	3.435181000	2.967096000
H	3.522314000	2.836407000	5.218849000
C	-0.981820000	6.237774000	0.954087000
F	-1.378880000	7.325599000	0.238626000
F	-0.964569000	5.178434000	0.088178000
F	-1.975781000	5.978217000	1.859148000
C	3.006920000	9.196710000	2.057014000
F	2.302069000	10.121779000	1.349288000
F	3.223086000	9.731707000	3.299919000
F	4.233819000	9.096355000	1.462604000

86

(m-bis-CF3-POCOP)IrH2-IC6F5 NS-bound-a2, E = -3497.846476

Ir	2.426584000	3.959211000	4.707941000
P	0.880674000	2.280585000	4.532790000
P	3.552073000	5.926151000	4.323902000
O	-0.289722000	2.900072000	3.408346000
C	0.147977000	-0.294989000	3.551367000
O	2.487444000	6.807333000	3.263732000
C	-1.654639000	1.588452000	5.714472000
C	5.605067000	7.377771000	2.982615000
C	-0.919880000	4.729756000	1.978344000
C	4.779755000	6.803321000	6.671367000
C	0.503559000	6.734776000	1.905383000
C	6.177756000	5.068837000	3.890251000
C	3.547225000	8.616028000	5.363709000
C	1.378184000	6.127196000	2.842928000
C	1.334311000	0.683127000	3.643255000
C	-0.636318000	6.019722000	1.486335000
C	4.656079000	5.314916000	1.889030000
C	1.136497000	4.829077000	3.345543000
C	2.532417000	0.049430000	4.382382000

C	-0.318190000	3.532670000	6.602315000
C	5.093663000	5.946508000	3.233449000
C	0.472722000	1.174846000	7.064204000
C	-0.025404000	4.150086000	2.914530000
C	3.567053000	7.132122000	5.777591000
C	2.254478000	6.792420000	6.528410000
C	-0.235390000	2.089008000	6.044019000
C	1.760823000	1.109566000	2.217321000
H	-0.130594000	-0.702896000	4.540950000
H	-0.738502000	0.186111000	3.095553000
H	0.440667000	-1.150973000	2.908346000
H	-2.125797000	2.205157000	4.926932000
H	-1.663270000	0.534171000	5.387320000
H	-2.275649000	1.660134000	6.631881000
H	4.803830000	8.030176000	2.586368000
H	6.024740000	7.833571000	3.898753000
H	6.418183000	7.337308000	2.228188000
H	4.835058000	5.721032000	6.905068000
H	5.732859000	7.111555000	6.201043000
H	4.683362000	7.354981000	7.629387000
H	5.822267000	4.032505000	4.040080000
H	7.064826000	5.037032000	3.224340000
H	6.507742000	5.455990000	4.871354000
H	4.493884000	8.933711000	4.893337000
H	2.718433000	8.828082000	4.663218000
H	3.401513000	9.233216000	6.274934000
H	4.217075000	4.308252000	2.030648000
H	3.917819000	5.948608000	1.363636000
H	5.551098000	5.211732000	1.241936000
H	2.274527000	-0.263348000	5.411199000
H	2.859638000	-0.852942000	3.825814000
H	3.386715000	0.750585000	4.442373000
H	-0.769538000	4.231177000	5.870875000
H	-0.946563000	3.539613000	7.516694000
H	0.687730000	3.910668000	6.885411000
H	1.517864000	1.497367000	7.245157000
H	-0.071279000	1.221537000	8.030306000
H	0.479656000	0.118411000	6.734790000
H	2.260249000	5.739939000	6.883699000
H	2.157191000	7.446887000	7.419140000
H	1.363765000	6.951156000	5.888900000
H	0.910888000	1.534288000	1.651430000
H	2.576813000	1.857901000	2.237938000
H	2.129986000	0.213717000	1.677355000
I	5.342702000	2.428982000	6.673773000
F	8.576009000	-0.861164000	9.673297000
F	10.890466000	0.399633000	8.903017000
F	6.165189000	-0.001117000	8.720659000
F	10.795094000	2.530273000	7.173336000
F	8.395358000	3.406269000	6.208882000
C	8.520037000	0.180795000	8.826551000
C	7.284510000	0.636137000	8.326395000

C	7.215754000	1.725079000	7.440602000
C	8.411667000	2.357930000	7.057143000
C	9.657450000	1.918940000	7.545201000
C	9.708979000	0.825703000	8.432568000
H	3.376667000	3.215581000	3.752951000
H	3.334192000	3.270551000	5.944597000
H	-1.317766000	6.476919000	0.759288000
C	-2.136619000	3.964996000	1.521432000
F	-2.864823000	4.655476000	0.601725000
F	-1.806056000	2.767868000	0.946774000
F	-2.983946000	3.673759000	2.556335000
C	0.810609000	8.109422000	1.366615000
F	0.856943000	9.054947000	2.354208000
F	2.024558000	8.154398000	0.734362000
F	-0.117849000	8.529370000	0.464106000

72

(PCP)IrH2 tilted S tol, E = -1727.849173

Ir	-0.000876000	-0.489905000	0.003558000
C	-0.000239000	1.624114000	-0.011108000
P	-2.280135000	-0.186793000	-0.102866000
P	2.278732000	-0.186665000	0.105905000
C	-2.457506000	1.597407000	-0.655135000
C	-3.265840000	-1.155109000	-1.422371000
C	-3.125733000	-0.224788000	1.613896000
C	2.456958000	1.604899000	0.633436000
C	3.264328000	-1.137218000	1.438324000
C	3.124264000	-0.248769000	-1.610239000
C	-1.191481000	2.359185000	-0.305512000
C	-1.186675000	3.771251000	-0.307912000
C	0.000544000	4.474792000	-0.030615000
C	1.191395000	2.362486000	0.273194000
C	1.187367000	3.774455000	0.256275000
H	-2.110707000	4.326887000	-0.535831000
H	0.000837000	5.576210000	-0.038111000
H	2.111691000	4.332647000	0.476647000
H	-2.578750000	1.568383000	-1.757878000
H	-3.372821000	2.078688000	-0.254815000
H	2.577983000	1.591138000	1.736513000
H	3.372566000	2.080040000	0.226520000
C	-3.441294000	-2.613536000	-0.954458000
C	-2.368859000	-1.137638000	-2.684046000
C	-4.633062000	-0.532037000	-1.772302000
C	-2.786100000	-1.564600000	2.302916000
C	-4.649675000	-0.015635000	1.569201000
C	-2.468528000	0.915031000	2.428494000
C	2.784799000	-1.598181000	-2.280312000
C	2.466774000	0.879312000	-2.440789000
C	4.648179000	-0.038647000	-1.568580000
C	3.440492000	-2.601658000	0.989839000
C	4.631299000	-0.509168000	1.780270000
C	2.367047000	-1.103296000	2.699413000
H	-2.838532000	0.866787000	3.474432000

H	-1.365810000	0.810106000	2.434818000
H	-2.713035000	1.917140000	2.027608000
H	-4.931075000	0.901258000	1.014673000
H	-5.033664000	0.091543000	2.606226000
H	-5.172083000	-0.877137000	1.112388000
H	-3.231188000	-2.432588000	1.783208000
H	-3.181003000	-1.551620000	3.341003000
H	-1.689219000	-1.709916000	2.341467000
H	-5.344718000	-0.567110000	-0.929493000
H	-5.083871000	-1.101527000	-2.613042000
H	-4.538408000	0.520399000	-2.102795000
H	-2.205460000	-0.109070000	-3.062783000
H	-1.376467000	-1.579985000	-2.473285000
H	-2.862659000	-1.719603000	-3.490995000
H	-3.823086000	-3.228830000	-1.796408000
H	-4.169776000	-2.695867000	-0.125421000
H	-2.476897000	-3.045987000	-0.621974000
H	2.837270000	0.816861000	-3.485802000
H	1.364125000	0.773605000	-2.446097000
H	2.710464000	1.886993000	-2.053629000
H	3.230654000	-2.458697000	-1.748969000
H	3.178939000	-1.599390000	-3.318767000
H	1.687956000	-1.744503000	-2.316028000
H	4.929345000	0.886020000	-1.026995000
H	5.032068000	0.054085000	-2.607030000
H	5.170884000	-0.893497000	-1.099748000
H	3.822302000	-3.205520000	1.840018000
H	4.169304000	-2.694615000	0.162223000
H	2.476453000	-3.039058000	0.662826000
H	2.203568000	-0.069850000	3.064596000
H	1.374703000	-1.548314000	2.494094000
H	2.860604000	-1.674682000	3.514037000
H	5.342972000	-0.554637000	0.937977000
H	5.082340000	-1.067733000	2.628186000
H	4.536231000	0.547333000	2.097386000
H	0.009138000	-1.919189000	0.742274000
H	-0.012111000	-1.929033000	-0.715635000

72

(PCP)IrH2 flat S tol, E = -1727.848269

C	-3.692941000	-0.884386000	1.219812000
C	-2.287749000	-0.744390000	1.225790000
C	-1.559373000	-0.621345000	-0.000103000
C	-2.287253000	-0.745818000	-1.226180000
C	-3.692416000	-0.885910000	-1.220621000
C	-4.392552000	-0.937118000	-0.000522000
H	-4.245695000	-0.956634000	2.170400000
H	-4.244703000	-0.959514000	-2.171363000
H	-5.489725000	-1.034401000	-0.000640000
P	0.189120000	-0.053936000	-2.283851000
P	0.188933000	-0.053357000	2.283872000
Ir	0.485430000	-0.085482000	0.000046000
C	0.013916000	1.714993000	-2.994337000

C	1.317587000	-1.165865000	-3.350688000
C	0.015372000	1.715561000	2.994816000
C	1.316670000	-1.166366000	3.350438000
C	1.265753000	2.542592000	-2.631957000
H	1.116352000	3.591671000	-2.964761000
H	1.430490000	2.540067000	-1.537768000
H	2.180848000	2.161081000	-3.120220000
C	2.712262000	-0.517191000	-3.454974000
H	3.085830000	-0.208142000	-2.458688000
H	3.429819000	-1.250077000	-3.880720000
H	2.706465000	0.367470000	-4.119921000
C	-1.200971000	2.329989000	-2.258550000
H	-2.154334000	1.837368000	-2.528359000
H	-1.074234000	2.250313000	-1.160837000
H	-1.283245000	3.403190000	-2.531202000
C	-0.223918000	1.753473000	-4.514787000
H	-0.478853000	2.791412000	-4.817982000
H	0.679575000	1.457828000	-5.080602000
H	-1.062698000	1.100759000	-4.826915000
C	0.762842000	-1.479166000	-4.755051000
H	0.715418000	-0.585593000	-5.401441000
H	1.431983000	-2.214349000	-5.251260000
H	-0.247141000	-1.930995000	-4.712953000
C	1.434419000	-2.483032000	-2.545346000
H	1.828622000	-2.291428000	-1.528198000
H	0.455107000	-2.990436000	-2.436730000
H	2.118457000	-3.178418000	-3.076695000
C	1.268428000	2.541892000	2.633750000
H	1.434808000	2.538482000	1.539803000
H	1.119386000	3.591319000	2.965584000
H	2.182485000	2.160002000	3.123660000
C	2.711800000	-0.518568000	3.454438000
H	3.429087000	-1.252079000	3.879544000
H	3.085108000	-0.209304000	2.458130000
H	2.706757000	0.365805000	4.119755000
C	-1.198215000	2.332177000	2.258159000
H	-1.070200000	2.253335000	1.160527000
H	-2.152343000	1.840156000	2.526357000
H	-1.280001000	3.405218000	2.531595000
C	-0.223531000	1.753805000	4.515128000
H	0.679372000	1.457381000	5.081474000
H	-0.477826000	2.791885000	4.818380000
H	-1.063024000	1.101709000	4.826711000
C	0.762281000	-1.479483000	4.754956000
H	1.431046000	-2.215276000	5.250748000
H	0.715768000	-0.585985000	5.401508000
H	-0.248040000	-1.930618000	4.713432000
C	1.432696000	-2.483412000	2.544836000
H	0.453353000	-2.990849000	2.436826000
H	1.826136000	-2.291701000	1.527412000
H	2.116962000	-3.179013000	3.075589000
C	-1.520690000	-0.782510000	2.535548000

H	-2.065830000	-0.300525000	3.371347000
H	-1.375632000	-1.841586000	2.834090000
C	-1.519577000	-0.784979000	-2.535519000
H	-1.372469000	-1.844260000	-2.832288000
H	-2.064865000	-0.304996000	-3.372391000
H	1.647844000	1.019869000	-0.000036000
H	2.066551000	-0.393881000	-0.000011000

72

(PCP)IrH2 tilted NS tol, E = -1727.84831

Ir	-0.011616000	-0.485070000	0.090175000
C	0.012503000	1.616664000	0.040157000
P	-2.290830000	-0.163901000	-0.026681000
P	2.272776000	-0.208989000	0.128367000
C	-2.446190000	1.620250000	-0.588584000
C	-3.127625000	-1.147732000	-1.430494000
C	-3.247599000	-0.232403000	1.617746000
C	2.474899000	1.577691000	0.653596000
C	3.347269000	-1.176981000	1.372902000
C	2.985522000	-0.277594000	-1.647706000
C	-1.170627000	2.371709000	-0.243976000
C	-1.149536000	3.783392000	-0.257013000
C	0.048156000	4.479533000	-0.005038000
C	1.214489000	2.351367000	0.301800000
C	1.229173000	3.761941000	0.268323000
H	-2.072180000	4.345783000	-0.476426000
H	0.061674000	5.580576000	-0.021403000
H	2.165796000	4.308182000	0.468386000
H	-2.571376000	1.588737000	-1.691144000
H	-3.359756000	2.107370000	-0.191870000
H	2.602012000	1.552276000	1.756850000
H	3.396850000	2.038541000	0.244246000
C	-3.345568000	-2.604669000	-0.977729000
C	-2.064657000	-1.127520000	-2.557800000
C	-4.445186000	-0.539464000	-1.949004000
C	-2.942559000	-1.572920000	2.320478000
C	-4.766332000	-0.052208000	1.438014000
C	-2.685794000	0.922225000	2.481381000
C	2.595274000	-1.630105000	-2.285412000
C	2.280800000	0.851116000	-2.438499000
C	4.508765000	-0.069079000	-1.717518000
C	3.562826000	-2.614871000	0.861359000
C	4.700032000	-0.496351000	1.670839000
C	2.513809000	-1.236935000	2.675053000
H	-3.131608000	0.859934000	3.496203000
H	-1.585179000	0.857443000	2.577710000
H	-2.930850000	1.917145000	2.063916000
H	-5.013579000	0.868635000	0.873711000
H	-5.245614000	0.029575000	2.436715000
H	-5.225226000	-0.915686000	0.920675000
H	-3.351458000	-2.438037000	1.767823000
H	-3.401369000	-1.567319000	3.331737000
H	-1.851411000	-1.723862000	2.426830000

H -5.244330000 -0.556243000 -1.186775000
H -4.800428000 -1.133002000 -2.818455000
H -4.317354000 0.504712000 -2.293881000
H -1.846987000 -0.099958000 -2.911673000
H -1.116698000 -1.589912000 -2.205601000
H -2.427445000 -1.713816000 -3.428133000
H -3.622844000 -3.225949000 -1.855478000
H -4.165942000 -2.686650000 -0.239333000
H -2.421323000 -3.023203000 -0.533083000
H 2.604866000 0.799904000 -3.499183000
H 1.178162000 0.748173000 -2.407289000
H 2.528434000 1.857475000 -2.052017000
H 3.100698000 -2.486298000 -1.804577000
H 2.879994000 -1.624761000 -3.358864000
H 1.502829000 -1.803425000 -2.214039000
H 4.822475000 0.870856000 -1.222218000
H 4.819867000 -0.004163000 -2.782098000
H 5.064945000 -0.909671000 -1.262344000
H 4.003958000 -3.230591000 1.673384000
H 4.259461000 -2.649922000 0.002427000
H 2.601812000 -3.077332000 0.561147000
H 2.254641000 -0.227612000 3.052887000
H 1.573290000 -1.797013000 2.515551000
H 3.105260000 -1.750655000 3.461958000
H 5.345643000 -0.423042000 0.778430000
H 5.245270000 -1.097035000 2.429705000
H 4.569639000 0.520275000 2.088945000
H -0.047081000 -0.500520000 1.625875000
H -0.032481000 -2.171558000 0.152104000
84
(PCP)IrH2-IC6F5-tilted, E = -2752.690815
Ir 0.036117000 -0.302192000 -0.110431000
C 0.046478000 1.778136000 -0.111587000
P -2.257772000 -0.006206000 -0.081841000
P 2.341951000 -0.024730000 -0.113950000
C -2.437870000 1.772656000 -0.628959000
C -3.393093000 -0.962426000 -1.277103000
C -2.892626000 -0.046172000 1.720587000
C 2.510646000 1.739349000 0.492972000
C 3.292811000 -1.036408000 1.194129000
C 3.192984000 -0.013734000 -1.819538000
C -1.155549000 2.525534000 -0.325397000
C -1.150641000 3.935056000 -0.268864000
C 0.044967000 4.634213000 -0.014669000
C 1.243370000 2.509660000 0.169852000
C 1.237741000 3.920679000 0.205283000
H -2.086757000 4.493275000 -0.432478000
H 0.045069000 5.734722000 0.017951000
H 2.171190000 4.466512000 0.418633000
H -2.599476000 1.732657000 -1.726919000
H -3.335818000 2.258251000 -0.197165000
H 2.631291000 1.673901000 1.594506000

H	3.427717000	2.230753000	0.110882000
C	-3.642990000	-2.385885000	-0.741002000
C	-2.610268000	-1.053239000	-2.608433000
C	-4.734559000	-0.241261000	-1.531133000
C	-2.531407000	-1.412848000	2.345605000
C	-4.401646000	0.225251000	1.852935000
C	-2.105260000	1.053233000	2.473800000
C	2.853078000	-1.317089000	-2.570867000
C	2.587663000	1.178407000	-2.598481000
C	4.719701000	0.163748000	-1.717709000
C	3.543178000	-2.463028000	0.668701000
C	4.617417000	-0.390385000	1.649179000
C	2.316268000	-1.103420000	2.394714000
H	-2.388719000	1.024129000	3.546587000
H	-1.010061000	0.895831000	2.403989000
H	-2.317305000	2.068198000	2.088719000
H	-4.697712000	1.176006000	1.367654000
H	-4.664039000	0.304959000	2.929299000
H	-5.009125000	-0.593641000	1.424825000
H	-3.089566000	-2.248920000	1.888914000
H	-2.774434000	-1.392290000	3.428790000
H	-1.450463000	-1.629948000	2.234660000
H	-5.338247000	-0.127802000	-0.613896000
H	-5.329002000	-0.843200000	-2.250510000
H	-4.591860000	0.759954000	-1.980687000
H	-2.333546000	-0.054031000	-3.000380000
H	-1.686556000	-1.648674000	-2.489306000
H	-3.248535000	-1.549124000	-3.369345000
H	-4.112792000	-2.999009000	-1.538188000
H	-4.328165000	-2.384962000	0.127717000
H	-2.697057000	-2.882574000	-0.453400000
H	2.973262000	1.153246000	-3.639001000
H	1.483219000	1.123474000	-2.634799000
H	2.864367000	2.154851000	-2.158207000
H	3.233405000	-2.220689000	-2.062490000
H	3.306981000	-1.279474000	-3.583516000
H	1.759895000	-1.436787000	-2.685116000
H	4.999595000	1.060703000	-1.131039000
H	5.136635000	0.290742000	-2.739067000
H	5.209853000	-0.719807000	-1.268224000
H	3.910914000	-3.100399000	1.500136000
H	4.310042000	-2.480956000	-0.128569000
H	2.615578000	-2.918811000	0.272730000
H	2.068138000	-0.097082000	2.789236000
H	1.373129000	-1.612534000	2.108839000
H	2.782564000	-1.679809000	3.220959000
H	5.360507000	-0.331703000	0.834851000
H	5.057743000	-1.010886000	2.458406000
H	4.469789000	0.627077000	2.059292000
H	-0.009667000	-1.997928000	0.132214000
H	0.012839000	-0.315994000	-1.646943000
I	0.150809000	-3.739081000	-1.227265000

C	0.335516000	-5.563381000	-2.415708000
C	-0.792878000	-6.313217000	-2.780651000
C	1.595754000	-5.997824000	-2.854915000
C	-0.678093000	-7.479449000	-3.562206000
C	1.740599000	-7.159959000	-3.637235000
C	0.595742000	-7.900399000	-3.992578000
F	2.713987000	-5.308816000	-2.533702000
F	-2.028878000	-5.934068000	-2.387909000
F	-1.766498000	-8.193129000	-3.906087000
F	0.719162000	-9.009636000	-4.739964000
F	2.954070000	-7.569486000	-4.052123000

12

IC6F5 S tol, E = -1024.819381

I	3.205353000	-0.037483000	-0.024314000
F	8.125274000	2.267686000	-0.017344000
F	9.457637000	-0.132274000	-0.027777000
F	5.398175000	2.320860000	-0.015654000
F	8.053003000	-2.490787000	-0.036515000
F	5.325606000	-2.461122000	-0.034967000
C	7.433870000	1.118771000	-0.021530000
C	6.025752000	1.135554000	-0.020656000
C	5.294093000	-0.069116000	-0.025164000
C	5.988877000	-1.295416000	-0.030484000
C	7.396857000	-1.321383000	-0.031379000
C	8.119301000	-0.111984000	-0.026966000

68

(p-H-POCOP)IrH2 S CH_2Cl_2, E = -1799.619572

C	-0.355759000	1.219202000	-3.618669000
C	-0.232887000	1.205719000	-2.212309000
C	-0.172450000	-0.010647000	-1.482883000
C	-0.237534000	-1.230657000	-2.205789000
C	-0.358754000	-1.251086000	-3.612235000
C	-0.416346000	-0.017650000	-4.295171000
H	-0.401942000	2.171542000	-4.165630000
H	-0.407043000	-2.206106000	-4.154310000
H	-0.511624000	-0.020384000	-5.392345000
O	-0.181426000	-2.423193000	-1.518674000
O	-0.168533000	2.401775000	-1.531877000
P	-0.028714000	-2.262147000	0.212959000
P	-0.018924000	2.249238000	0.200810000
Ir	0.005895000	-0.005609000	0.588119000
H	0.944751000	0.012533000	1.888942000
H	-0.666463000	-0.015036000	2.048313000
C	1.600229000	-3.197450000	0.454408000
C	-1.581121000	-3.224074000	0.717537000
C	1.601450000	3.205230000	0.424012000
C	-1.579267000	3.191911000	0.714290000
C	1.976461000	-3.145547000	1.950841000
H	2.993606000	-3.571065000	2.079079000
H	1.985862000	-2.103269000	2.324206000
H	1.283875000	-3.736558000	2.578415000
C	-1.525910000	-3.482947000	2.236859000

H	-1.289914000	-2.557488000	2.799320000
H	-2.516797000	-3.848475000	2.578196000
H	-0.777209000	-4.255759000	2.495896000
C	2.635593000	-2.383097000	-0.360035000
H	2.416898000	-2.418967000	-1.444258000
H	2.645028000	-1.321168000	-0.042587000
H	3.644062000	-2.814926000	-0.192500000
C	1.563831000	-4.649932000	-0.053546000
H	2.596809000	-5.057093000	-0.050427000
H	0.950373000	-5.299257000	0.598231000
H	1.175350000	-4.710429000	-1.087954000
C	-1.783293000	-4.537483000	-0.062905000
H	-1.047387000	-5.311407000	0.216249000
H	-2.792841000	-4.936075000	0.170987000
H	-1.724029000	-4.368871000	-1.154422000
C	-2.746819000	-2.257279000	0.391885000
H	-2.640748000	-1.301182000	0.940822000
H	-2.788436000	-2.026843000	-0.690565000
H	-3.704969000	-2.736792000	0.682183000
C	1.823396000	3.451062000	1.930265000
H	1.691153000	2.520807000	2.518105000
H	2.860583000	3.812954000	2.089130000
H	1.136091000	4.221998000	2.327670000
C	-1.682725000	3.164521000	2.254268000
H	-2.663010000	3.589697000	2.554867000
H	-1.619716000	2.128770000	2.640561000
H	-0.892614000	3.768354000	2.737958000
C	2.686675000	2.239908000	-0.114784000
H	2.678700000	1.279139000	0.436041000
H	2.532158000	2.019169000	-1.188943000
H	3.682586000	2.715978000	0.002431000
C	1.662163000	4.524624000	-0.369778000
H	0.993351000	5.297923000	0.046384000
H	2.699097000	4.918634000	-0.322419000
H	1.402707000	4.365594000	-1.433178000
C	-1.637722000	4.635866000	0.184127000
H	-2.655985000	5.041602000	0.361261000
H	-0.922809000	5.297289000	0.707538000
H	-1.436502000	4.679575000	-0.903145000
C	-2.741218000	2.365261000	0.109791000
H	-2.716501000	2.380604000	-0.996505000
H	-2.693723000	1.309272000	0.443307000
H	-3.704954000	2.802473000	0.443884000

68

(p-H-POCOP)IrH2 NS CH_2Cl_2, E = -1799.62042

C	-0.183594000	1.113060000	-3.701922000
C	-0.154217000	1.180320000	-2.292859000
C	-0.123211000	0.016671000	-1.484113000
C	-0.114100000	-1.238483000	-2.142663000
C	-0.142786000	-1.347017000	-3.549162000
C	-0.177782000	-0.159866000	-4.312367000
H	-0.207635000	2.033841000	-4.302384000

H	-0.135220000	-2.335282000	-4.031059000
H	-0.200671000	-0.228445000	-5.411083000
O	-0.069081000	-2.391321000	-1.375512000
O	-0.148626000	2.420060000	-1.674306000
P	-0.058445000	-2.129586000	0.340096000
P	-0.125959000	2.373511000	0.060502000
Ir	-0.117707000	0.145882000	0.592272000
H	1.376649000	0.192396000	0.961936000
H	-0.213617000	0.246388000	2.259819000
C	1.512849000	-3.074990000	0.793142000
C	-1.672829000	-3.016218000	0.782570000
C	1.419847000	3.410650000	0.382214000
C	-1.763340000	3.262944000	0.400237000
C	1.811334000	-2.829540000	2.288101000
H	2.783905000	-3.300785000	2.540947000
H	1.877862000	-1.747009000	2.509537000
H	1.041097000	-3.270886000	2.947407000
C	-1.696564000	-3.285531000	2.300222000
H	-1.446127000	-2.373115000	2.877473000
H	-2.716490000	-3.609433000	2.595062000
H	-0.993528000	-4.090919000	2.586406000
C	2.634576000	-2.445699000	-0.067568000
H	2.466990000	-2.627702000	-1.145649000
H	2.707195000	-1.352482000	0.093219000
H	3.603303000	-2.904168000	0.219609000
C	1.412637000	-4.581580000	0.490152000
H	2.407661000	-5.047230000	0.650075000
H	0.695458000	-5.090611000	1.160938000
H	1.114227000	-4.765816000	-0.559625000
C	-1.923408000	-4.306706000	-0.020136000
H	-1.221794000	-5.114765000	0.251223000
H	-2.950979000	-4.666207000	0.198737000
H	-1.845568000	-4.122310000	-1.107755000
C	-2.758755000	-1.971708000	0.420749000
H	-2.631410000	-1.042676000	1.016322000
H	-2.731962000	-1.708206000	-0.654670000
H	-3.762556000	-2.385272000	0.651071000
C	1.730479000	3.362056000	1.893954000
H	1.834531000	2.318168000	2.247286000
H	2.687375000	3.892826000	2.079913000
H	0.947543000	3.855837000	2.498889000
C	-1.790023000	3.717739000	1.872955000
H	-2.818269000	4.045960000	2.131833000
H	-1.511102000	2.891820000	2.557469000
H	-1.110094000	4.572291000	2.052638000
C	2.555615000	2.709742000	-0.401289000
H	2.657583000	1.646977000	-0.107311000
H	2.380114000	2.752505000	-1.492555000
H	3.512595000	3.225278000	-0.178795000
C	1.276496000	4.864511000	-0.105154000
H	0.548819000	5.433740000	0.503007000
H	2.258941000	5.373157000	-0.012600000

H	0.968089000	4.908710000	-1.167196000
C	-2.053406000	4.436579000	-0.554137000
H	-3.090369000	4.790677000	-0.374498000
H	-1.374587000	5.291504000	-0.389195000
H	-1.973622000	4.121403000	-1.611154000
C	-2.820525000	2.152016000	0.178303000
H	-2.792120000	1.759757000	-0.857094000
H	-2.662773000	1.306817000	0.881716000
H	-3.834248000	2.562911000	0.365672000

68

(p-H-POCOP)IrH2 S CH_2Cl_2 with epsilon=24.2, E = -1799.620412

C	-0.353432000	1.221865000	-3.618709000
C	-0.234494000	1.208125000	-2.211800000
C	-0.171729000	-0.008569000	-1.482897000
C	-0.230486000	-1.228563000	-2.206569000
C	-0.347732000	-1.248592000	-3.613583000
C	-0.407769000	-0.014922000	-4.296099000
H	-0.401119000	2.174241000	-4.165598000
H	-0.390978000	-2.203389000	-4.156585000
H	-0.499867000	-0.017405000	-5.393564000
O	-0.171665000	-2.421050000	-1.520284000
O	-0.175598000	2.403841000	-1.530811000
P	-0.028336000	-2.261664000	0.213117000
P	-0.022427000	2.251295000	0.202461000
Ir	0.000500000	-0.004434000	0.588492000
H	0.953472000	0.012389000	1.876529000
H	-0.663332000	-0.013280000	2.054318000
C	1.599167000	-3.196467000	0.464371000
C	-1.583232000	-3.224876000	0.707894000
C	1.601474000	3.201325000	0.422521000
C	-1.581117000	3.196204000	0.716175000
C	1.968738000	-3.138587000	1.962384000
H	2.983524000	-3.567653000	2.096942000
H	1.980955000	-2.094570000	2.330737000
H	1.271027000	-3.723721000	2.589729000
C	-1.534177000	-3.489152000	2.226486000
H	-1.302021000	-2.565513000	2.793560000
H	-2.526148000	-3.857147000	2.561872000
H	-0.785726000	-4.262090000	2.485796000
C	2.639013000	-2.386805000	-0.348841000
H	2.425936000	-2.428406000	-1.434008000
H	2.647498000	-1.323311000	-0.036688000
H	3.646287000	-2.818314000	-0.173757000
C	1.563838000	-4.651528000	-0.036311000
H	2.596356000	-5.059577000	-0.024387000
H	0.945643000	-5.296482000	0.615332000
H	1.182051000	-4.717697000	-1.072885000
C	-1.782635000	-4.535806000	-0.077537000
H	-1.047526000	-5.310552000	0.201269000
H	-2.792811000	-4.935027000	0.152382000
H	-1.720563000	-4.363767000	-1.168380000
C	-2.747599000	-2.257022000	0.380715000

H	-2.643919000	-1.302784000	0.933430000
H	-2.785020000	-2.023052000	-0.701181000
H	-3.706855000	-2.737480000	0.665582000
C	1.845765000	3.413398000	1.930592000
H	1.724359000	2.469395000	2.498343000
H	2.884522000	3.774110000	2.081352000
H	1.163167000	4.173042000	2.356396000
C	-1.665491000	3.201399000	2.257376000
H	-2.648871000	3.617188000	2.560790000
H	-1.580824000	2.175997000	2.666848000
H	-0.879875000	3.829656000	2.716806000
C	2.678064000	2.246969000	-0.152030000
H	2.671663000	1.272030000	0.373483000
H	2.513225000	2.055535000	-1.230196000
H	3.676470000	2.716631000	-0.031079000
C	1.651169000	4.538069000	-0.342017000
H	0.993942000	5.303263000	0.106633000
H	2.690179000	4.927726000	-0.306789000
H	1.369574000	4.405205000	-1.403465000
C	-1.655954000	4.627983000	0.155461000
H	-2.676368000	5.028348000	0.332098000
H	-0.942891000	5.306595000	0.658450000
H	-1.463555000	4.649647000	-0.934037000
C	-2.744678000	2.349521000	0.142918000
H	-2.729252000	2.336188000	-0.963718000
H	-2.689968000	1.302698000	0.503151000
H	-3.707428000	2.791648000	0.473315000

68

(p-H-POCOP)IrH2 NS CH_2Cl_2 with epsilon=24.2, E = -1799.621512

C	-0.179989000	1.112529000	-3.702601000
C	-0.150612000	1.180290000	-2.293309000
C	-0.120268000	0.016877000	-1.484181000
C	-0.112090000	-1.238495000	-2.142337000
C	-0.140779000	-1.347494000	-3.549046000
C	-0.174832000	-0.160596000	-4.312897000
H	-0.203779000	2.033139000	-4.303465000
H	-0.134228000	-2.335961000	-4.030701000
H	-0.197748000	-0.229517000	-5.411621000
O	-0.068588000	-2.391112000	-1.375093000
O	-0.145066000	2.420054000	-1.675412000
P	-0.057543000	-2.130173000	0.341113000
P	-0.124175000	2.374800000	0.059965000
Ir	-0.114265000	0.146394000	0.591348000
H	1.386155000	0.191776000	0.934039000
H	-0.200381000	0.247645000	2.260536000
C	1.511932000	-3.078443000	0.793481000
C	-1.673345000	-3.013288000	0.784715000
C	1.419211000	3.414819000	0.382912000
C	-1.763739000	3.260023000	0.398336000
C	1.811886000	-2.832428000	2.288059000
H	2.782733000	-3.307200000	2.540736000
H	1.883138000	-1.749827000	2.507856000

H	1.040338000	-3.270172000	2.948192000
C	-1.694767000	-3.288096000	2.301497000
H	-1.441783000	-2.378692000	2.882403000
H	-2.714950000	-3.610967000	2.596398000
H	-0.993020000	-4.096062000	2.583342000
C	2.635115000	-2.452925000	-0.067981000
H	2.466512000	-2.634599000	-1.145984000
H	2.711963000	-1.359997000	0.093121000
H	3.602325000	-2.914768000	0.218750000
C	1.407340000	-4.585051000	0.491942000
H	2.400845000	-5.053303000	0.653389000
H	0.687881000	-5.091195000	1.162405000
H	1.109599000	-4.769470000	-0.558003000
C	-1.928859000	-4.300792000	-0.021292000
H	-1.226837000	-5.110471000	0.244078000
H	-2.955789000	-4.659646000	0.201357000
H	-1.856241000	-4.112757000	-1.108646000
C	-2.757372000	-1.964975000	0.428427000
H	-2.626850000	-1.038145000	1.026839000
H	-2.732484000	-1.698333000	-0.646285000
H	-3.761679000	-2.376585000	0.659817000
C	1.729936000	3.363704000	1.894584000
H	1.840020000	2.319300000	2.244630000
H	2.683908000	3.899175000	2.081811000
H	0.944311000	3.851681000	2.500768000
C	-1.792766000	3.715270000	1.870919000
H	-2.821709000	4.042764000	2.127736000
H	-1.514650000	2.889798000	2.556327000
H	-1.113632000	4.570244000	2.051375000
C	2.557179000	2.718982000	-0.401839000
H	2.662902000	1.656007000	-0.109729000
H	2.381980000	2.763713000	-1.493104000
H	3.512371000	3.237137000	-0.177938000
C	1.271697000	4.869477000	-0.100914000
H	0.541705000	5.434849000	0.508013000
H	2.252581000	5.380599000	-0.005973000
H	0.964256000	4.915605000	-1.163165000
C	-2.055527000	4.432922000	-0.556386000
H	-3.093365000	4.784534000	-0.377206000
H	-1.378623000	5.289240000	-0.390940000
H	-1.974435000	4.117703000	-1.613301000
C	-2.818073000	2.146582000	0.175649000
H	-2.788686000	1.754692000	-0.859854000
H	-2.658860000	1.301511000	0.878996000
H	-3.832758000	2.554845000	0.363199000

73

(p-H-POCOP)IrH2---CH_2Cl_2 dihydrogen bonded, E = -2758.847255

C	-0.091514000	1.203633000	-3.608744000
C	-0.127155000	1.191264000	-2.197777000
C	-0.140830000	-0.023107000	-1.463100000
C	-0.115510000	-1.243107000	-2.188148000
C	-0.079511000	-1.266000000	-3.599069000

C	-0.069064000	-0.033808000	-4.287391000
H	-0.078946000	2.155657000	-4.158294000
H	-0.057937000	-2.222141000	-4.141128000
H	-0.042134000	-0.038004000	-5.388321000
O	-0.115909000	-2.434942000	-1.493848000
O	-0.137810000	2.388540000	-1.512335000
P	-0.188769000	-2.272292000	0.241737000
P	-0.173775000	2.235382000	0.225270000
Ir	-0.247111000	-0.016503000	0.612496000
H	0.899171000	-0.027611000	1.677224000
H	-0.800000000	-0.033721000	2.155587000
C	1.383275000	-3.214185000	0.705488000
C	-1.800208000	-3.222848000	0.542769000
C	1.419015000	3.165568000	0.643978000
C	-1.768578000	3.204274000	0.555109000
C	1.565441000	-3.147784000	2.236388000
H	2.547285000	-3.595758000	2.496836000
H	1.554746000	-2.105844000	2.603908000
H	0.784308000	-3.709847000	2.779534000
C	-1.910496000	-3.535959000	2.048584000
H	-1.699160000	-2.642713000	2.668830000
H	-2.942601000	-3.877535000	2.273017000
H	-1.217401000	-4.344586000	2.349761000
C	2.528637000	-2.430460000	0.019117000
H	2.446185000	-2.475051000	-1.083502000
H	2.523623000	-1.366001000	0.324959000
H	3.498169000	-2.881458000	0.315905000
C	1.381614000	-4.673211000	0.213928000
H	2.392736000	-5.104728000	0.369194000
H	0.664577000	-5.296955000	0.779643000
H	1.142398000	-4.740746000	-0.864541000
C	-1.946513000	-4.500387000	-0.305726000
H	-1.241376000	-5.292785000	0.000040000
H	-2.975091000	-4.897229000	-0.172943000
H	-1.794722000	-4.287676000	-1.380399000
C	-2.903242000	-2.213979000	0.134808000
H	-2.843655000	-1.289665000	0.744468000
H	-2.818130000	-1.929851000	-0.932481000
H	-3.899017000	-2.678849000	0.291404000
C	1.656210000	3.075651000	2.166060000
H	1.709664000	2.024308000	2.502760000
H	2.625933000	3.559709000	2.405841000
H	0.870795000	3.591893000	2.748465000
C	-1.850978000	3.509914000	2.064347000
H	-2.870983000	3.874455000	2.306561000
H	-1.658682000	2.602863000	2.672105000
H	-1.133755000	4.297635000	2.364623000
C	2.536875000	2.387672000	-0.092358000
H	2.524061000	1.314604000	0.182465000
H	2.428287000	2.466197000	-1.190638000
H	3.519416000	2.816698000	0.193837000
C	1.406661000	4.631928000	0.175536000

H	0.716752000	5.251174000	0.778798000
H	2.425903000	5.055228000	0.296408000
H	1.125301000	4.716974000	-0.891367000
C	-1.916389000	4.487888000	-0.283910000
H	-2.942460000	4.887768000	-0.141540000
H	-1.206355000	5.276044000	0.021088000
H	-1.773570000	4.280492000	-1.360869000
C	-2.888924000	2.208947000	0.161205000
H	-2.825907000	1.932208000	-0.909493000
H	-2.826499000	1.279536000	0.762954000
H	-3.877113000	2.681911000	0.339917000
H	0.014976000	0.605209000	3.836427000
C	0.465090000	0.219377000	4.769431000
H	0.349707000	0.902167000	5.627927000
Cl	-0.359231000	-1.324519000	5.162188000
Cl	2.224736000	0.008000000	4.462945000

73

(p-H-POCOP)IrH2---CH_2Cl_2 halogen bonded, E = -2758.843023

C	-0.222321000	1.115707000	-3.721580000
C	-0.197932000	1.188947000	-2.311803000
C	-0.183362000	0.019317000	-1.508240000
C	-0.188802000	-1.243439000	-2.155502000
C	-0.213336000	-1.350388000	-3.563456000
C	-0.230584000	-0.161149000	-4.323122000
H	-0.232579000	2.032047000	-4.328737000
H	-0.215841000	-2.336810000	-4.048649000
H	-0.249589000	-0.231648000	-5.422051000
O	-0.162277000	-2.390376000	-1.392675000
O	-0.182429000	2.425043000	-1.700611000
P	-0.089607000	-2.122581000	0.333913000
P	-0.168153000	2.373686000	0.045529000
Ir	-0.156930000	0.151903000	0.564854000
H	0.789144000	0.249259000	1.837153000
H	-0.808484000	0.233982000	2.042648000
C	1.556715000	-2.984886000	0.700884000
C	-1.624069000	-3.115800000	0.833294000
C	1.423849000	3.344323000	0.370495000
C	-1.765926000	3.340518000	0.364323000
C	1.890375000	-2.794231000	2.195474000
H	2.907254000	-3.196958000	2.386520000
H	1.878491000	-1.724756000	2.476106000
H	1.187191000	-3.324060000	2.862325000
C	-1.578857000	-3.347201000	2.356664000
H	-1.361103000	-2.413960000	2.910776000
H	-2.565628000	-3.726644000	2.695389000
H	-0.818335000	-4.100231000	2.636624000
C	2.597213000	-2.219238000	-0.152694000
H	2.417348000	-2.359531000	-1.235462000
H	2.567949000	-1.133335000	0.066517000
H	3.610100000	-2.604779000	0.086255000
C	1.567081000	-4.476453000	0.321458000
H	2.605888000	-4.860253000	0.402820000

H	0.937414000	-5.078865000	1.002626000
H	1.224609000	-4.635388000	-0.718905000
C	-1.786424000	-4.445472000	0.071503000
H	-1.022694000	-5.190502000	0.354055000
H	-2.780385000	-4.874230000	0.318984000
H	-1.740607000	-4.289435000	-1.022485000
C	-2.808265000	-2.182841000	0.477726000
H	-2.734809000	-1.219580000	1.019820000
H	-2.836658000	-1.963130000	-0.607712000
H	-3.759748000	-2.682670000	0.755930000
C	1.674762000	3.369685000	1.894239000
H	1.651648000	2.348556000	2.321569000
H	2.677293000	3.806407000	2.084805000
H	0.931569000	3.988511000	2.430470000
C	-1.831654000	3.691397000	1.864689000
H	-2.850898000	4.057652000	2.107749000
H	-1.624796000	2.805169000	2.497610000
H	-1.116084000	4.492443000	2.131571000
C	2.532423000	2.509595000	-0.316634000
H	2.526427000	1.463783000	0.049798000
H	2.401770000	2.491725000	-1.415381000
H	3.518736000	2.964256000	-0.088505000
C	1.410807000	4.770524000	-0.208337000
H	0.732471000	5.437909000	0.355117000
H	2.433188000	5.197464000	-0.135460000
H	1.113658000	4.774987000	-1.274423000
C	-1.926192000	4.598390000	-0.510773000
H	-2.955682000	4.993131000	-0.379240000
H	-1.223354000	5.401403000	-0.228193000
H	-1.782223000	4.362388000	-1.581788000
C	-2.887850000	2.331440000	0.013519000
H	-2.846346000	2.037685000	-1.053538000
H	-2.802977000	1.411140000	0.624957000
H	-3.873715000	2.804048000	0.205945000
H	-0.498253000	-1.147807000	6.827525000
C	0.288381000	-1.458461000	6.118881000
H	1.299633000	-1.279797000	6.522361000
Cl	0.109490000	-3.227543000	5.839825000
Cl	0.105729000	-0.497814000	4.613780000

74

(p-MeOOC-POCOP)IrH2 S CH_2Cl_2, E = -2027.292362

C	-0.352971000	1.198525000	-3.618662000
C	-0.230699000	1.190920000	-2.216323000
C	-0.172322000	-0.023048000	-1.483720000
C	-0.238909000	-1.245687000	-2.200526000
C	-0.359620000	-1.274661000	-3.604028000
C	-0.416147000	-0.041602000	-4.300003000
H	-0.398979000	2.137689000	-4.184694000
H	-0.408769000	-2.228096000	-4.143234000
O	-0.184293000	-2.433624000	-1.509535000
O	-0.164849000	2.387349000	-1.541526000
P	-0.028991000	-2.269467000	0.223811000

P	-0.018571000	2.244597000	0.194350000
Ir	0.004988000	-0.010193000	0.589071000
H	0.931253000	0.016292000	1.898055000
H	-0.643584000	-0.016776000	2.060305000
C	1.601193000	-3.201113000	0.464840000
C	-1.580630000	-3.229687000	0.730780000
C	1.600904000	3.200935000	0.414290000
C	-1.581552000	3.185953000	0.698849000
C	1.984079000	-3.132297000	1.959133000
H	2.999710000	-3.561170000	2.087536000
H	2.000643000	-2.085649000	2.319700000
H	1.291620000	-3.712215000	2.597024000
C	-1.518384000	-3.491891000	2.249339000
H	-1.279733000	-2.567993000	2.813311000
H	-2.507978000	-3.857662000	2.593924000
H	-0.768955000	-4.265794000	2.502865000
C	2.631996000	-2.395099000	-0.363520000
H	2.410778000	-2.445897000	-1.446609000
H	2.640722000	-1.329098000	-0.059575000
H	3.641798000	-2.822422000	-0.193101000
C	1.561965000	-4.659292000	-0.026029000
H	2.594614000	-5.067084000	-0.019849000
H	0.949179000	-5.300053000	0.634865000
H	1.171973000	-4.732197000	-1.059086000
C	-1.787266000	-4.541000000	-0.052033000
H	-1.049082000	-5.315362000	0.219618000
H	-2.794940000	-4.940375000	0.188222000
H	-1.736382000	-4.369470000	-1.143500000
C	-2.745967000	-2.260159000	0.411912000
H	-2.636788000	-1.305564000	0.963019000
H	-2.792184000	-2.027489000	-0.669912000
H	-3.703615000	-2.738831000	0.704948000
C	1.818224000	3.454979000	1.919942000
H	1.686153000	2.527912000	2.512881000
H	2.854454000	3.819191000	2.079193000
H	1.128750000	4.227129000	2.311120000
C	-1.689151000	3.160727000	2.238741000
H	-2.669238000	3.588861000	2.535465000
H	-1.630319000	2.125385000	2.626792000
H	-0.898993000	3.763280000	2.723959000
C	2.687011000	2.232210000	-0.116703000
H	2.677608000	1.274228000	0.439080000
H	2.536111000	2.006616000	-1.190390000
H	3.682668000	2.708461000	0.001062000
C	1.662585000	4.515849000	-0.386829000
H	0.991599000	5.290755000	0.022724000
H	2.698991000	4.910838000	-0.337845000
H	1.407281000	4.350799000	-1.450303000
C	-1.639013000	4.628811000	0.165919000
H	-2.657809000	5.034207000	0.340303000
H	-0.925453000	5.291192000	0.689966000
H	-1.435732000	4.670867000	-0.921023000

C	-2.740208000	2.356298000	0.091979000
H	-2.714629000	2.372585000	-1.014261000
H	-2.691180000	1.300281000	0.425709000
H	-3.705413000	2.790748000	0.425120000
C	-0.545219000	0.007272000	-5.792948000
O	-0.597666000	1.039901000	-6.455066000
O	-0.599405000	-1.229924000	-6.350308000
C	-0.723207000	-1.269383000	-7.786640000
H	-1.652225000	-0.760788000	-8.113159000
H	0.144041000	-0.774468000	-8.267597000
H	-0.754507000	-2.339440000	-8.057221000

74

(p-MeOOC-POCOP)IrH2 NS CH_2Cl_2, E = -2027.292362

C	-0.198390000	1.091966000	-3.709469000
C	-0.152498000	1.170682000	-2.307715000
C	-0.108479000	0.012592000	-1.489402000
C	-0.104903000	-1.248304000	-2.139358000
C	-0.149902000	-1.370728000	-3.539267000
C	-0.197048000	-0.187645000	-4.321569000
H	-0.233015000	1.995655000	-4.331823000
H	-0.146521000	-2.358964000	-4.014925000
O	-0.052790000	-2.393487000	-1.363532000
O	-0.147265000	2.413368000	-1.698864000
P	-0.019794000	-2.122267000	0.350070000
P	-0.102866000	2.384809000	0.035830000
Ir	-0.071706000	0.157781000	0.578641000
H	1.452265000	0.201193000	0.783733000
H	-0.084449000	0.274436000	2.251361000
C	1.550280000	-3.072976000	0.790880000
C	-1.635013000	-2.987350000	0.823818000
C	1.432571000	3.443229000	0.329217000
C	-1.747765000	3.249006000	0.394727000
C	1.874204000	-2.804273000	2.276638000
H	2.845900000	-3.280874000	2.522260000
H	1.954782000	-1.718691000	2.477719000
H	1.110194000	-3.226327000	2.955487000
C	-1.634550000	-3.249396000	2.343025000
H	-1.364175000	-2.337113000	2.911465000
H	-2.652675000	-3.560369000	2.656955000
H	-0.935469000	-4.061000000	2.620812000
C	2.664156000	-2.469897000	-0.098055000
H	2.477351000	-2.665818000	-1.170442000
H	2.754710000	-1.375556000	0.045667000
H	3.631881000	-2.935364000	0.180625000
C	1.429516000	-4.583323000	0.514649000
H	2.421335000	-5.056865000	0.670486000
H	0.715283000	-5.072975000	1.202731000
H	1.116646000	-4.782078000	-0.528252000
C	-1.913360000	-4.277517000	0.030043000
H	-1.214097000	-5.091371000	0.289882000
H	-2.939703000	-4.626172000	0.270611000
H	-1.855724000	-4.097267000	-1.059538000

C	-2.713754000	-1.930635000	0.476901000
H	-2.567097000	-1.002079000	1.069342000
H	-2.704207000	-1.670769000	-0.599609000
H	-3.718545000	-2.329719000	0.726978000
C	1.769782000	3.395558000	1.835389000
H	1.899009000	2.352478000	2.182882000
H	2.719785000	3.943338000	2.006070000
H	0.988601000	3.872998000	2.455473000
C	-1.753501000	3.727137000	1.860371000
H	-2.781780000	4.044083000	2.132377000
H	-1.450278000	2.916967000	2.553305000
H	-1.083419000	4.594408000	2.013627000
C	2.565655000	2.763110000	-0.475969000
H	2.693871000	1.702105000	-0.185581000
H	2.371260000	2.804944000	-1.563978000
H	3.517122000	3.294603000	-0.268479000
C	1.256095000	4.895348000	-0.153190000
H	0.527451000	5.450963000	0.466342000
H	2.230773000	5.420577000	-0.073404000
H	0.932519000	4.936019000	-1.210850000
C	-2.076769000	4.400721000	-0.573442000
H	-3.114614000	4.742261000	-0.376104000
H	-1.407412000	5.268239000	-0.438952000
H	-2.016220000	4.068359000	-1.626442000
C	-2.787233000	2.114717000	0.212180000
H	-2.774175000	1.705978000	-0.817046000
H	-2.600925000	1.285415000	0.928025000
H	-3.804783000	2.508027000	0.415107000
C	-0.247222000	-0.234084000	-5.812531000
O	-0.288734000	0.752050000	-6.545703000
O	-0.242619000	-1.507149000	-6.296218000
C	-0.289211000	-1.634404000	-7.730537000
H	-1.212344000	-1.173958000	-8.136456000
H	0.588456000	-1.144106000	-8.197708000
H	-0.278233000	-2.719035000	-7.938410000

79

(p-MeOOC-POCOP)IrH2---CH_2Cl_2 dihydrogen bonded, E = -2986.518733

C	-0.011631000	1.180344000	-3.601208000
C	-0.072258000	1.173499000	-2.197564000
C	-0.108864000	-0.033658000	-1.452305000
C	-0.093186000	-1.253626000	-2.176603000
C	-0.032610000	-1.289444000	-3.581161000
C	0.009189000	-0.060150000	-4.288753000
H	0.019651000	2.120108000	-4.167805000
H	-0.017839000	-2.246426000	-4.116702000
O	-0.137734000	-2.443558000	-1.473359000
O	-0.096547000	2.375660000	-1.514625000
P	-0.217007000	-2.278080000	0.254881000
P	-0.190338000	2.237955000	0.215514000
Ir	-0.267591000	-0.015055000	0.616250000
H	1.183680000	-0.020674000	1.135274000
H	-0.538336000	-0.022765000	2.259168000

C	1.315886000	-3.272096000	0.728988000
C	-1.864300000	-3.153679000	0.568768000
C	1.350461000	3.222678000	0.685599000
C	-1.830691000	3.138455000	0.497542000
C	1.522707000	-3.155862000	2.252793000
H	2.478538000	-3.651947000	2.521922000
H	1.581764000	-2.100938000	2.574460000
H	0.715509000	-3.643753000	2.827960000
C	-1.961704000	-3.499129000	2.068648000
H	-1.692185000	-2.633943000	2.706762000
H	-3.005884000	-3.791582000	2.305467000
H	-1.306423000	-4.350113000	2.335201000
C	2.492990000	-2.584660000	-0.004169000
H	2.389003000	-2.661286000	-1.102845000
H	2.570361000	-1.513360000	0.264808000
H	3.436999000	-3.085479000	0.294019000
C	1.222293000	-4.745821000	0.291798000
H	2.204052000	-5.233646000	0.466354000
H	0.466548000	-5.301579000	0.877742000
H	0.981046000	-4.838005000	-0.784459000
C	-2.097613000	-4.395424000	-0.311783000
H	-1.420171000	-5.228127000	-0.053671000
H	-3.138896000	-4.748629000	-0.157394000
H	-1.969049000	-4.155685000	-1.383796000
C	-2.912492000	-2.069756000	0.211274000
H	-2.800564000	-1.177802000	0.864057000
H	-2.827235000	-1.749354000	-0.845666000
H	-3.933633000	-2.475266000	0.367182000
C	1.575345000	3.077834000	2.205412000
H	1.719420000	2.019091000	2.486759000
H	2.494741000	3.632867000	2.485398000
H	0.738703000	3.488786000	2.800121000
C	-1.947288000	3.493777000	1.993322000
H	-2.984009000	3.827517000	2.206958000
H	-1.729964000	2.616667000	2.635503000
H	-1.264717000	4.318332000	2.274442000
C	2.519235000	2.547457000	-0.071478000
H	2.576464000	1.464001000	0.151014000
H	2.419727000	2.673053000	-1.165809000
H	3.470613000	3.018313000	0.251061000
C	1.255290000	4.704731000	0.278382000
H	0.507043000	5.251494000	0.882221000
H	2.240213000	5.186969000	0.450530000
H	1.003113000	4.816977000	-0.793368000
C	-2.036022000	4.377673000	-0.393484000
H	-3.079469000	4.735433000	-0.266899000
H	-1.362464000	5.208794000	-0.121080000
H	-1.881678000	4.132999000	-1.460950000
C	-2.887004000	2.065347000	0.131374000
H	-2.794752000	1.742361000	-0.924031000
H	-2.791298000	1.172837000	0.786109000
H	-3.905060000	2.482149000	0.277262000

H	0.356120000	0.456503000	3.795414000
C	0.898750000	0.158845000	4.713704000
H	0.850628000	0.910485000	5.519598000
Cl	0.141371000	-1.355062000	5.310002000
Cl	2.627389000	-0.056465000	4.260772000
C	0.072899000	-0.015669000	-5.779923000
O	0.107647000	1.013516000	-6.451429000
O	0.089497000	-1.256900000	-6.339233000
C	0.150255000	-1.296983000	-7.778210000
H	-0.731904000	-0.794097000	-8.223004000
H	1.068622000	-0.797683000	-8.147245000
H	0.159483000	-2.367199000	-8.050719000

79

(p-MeOOC-POCOP)IrH2---CH_2Cl_2 halogen bonded, E = -2986.513997

C	-0.197508000	1.088701000	-3.716977000
C	-0.153296000	1.173220000	-2.315435000
C	-0.110937000	0.017882000	-1.493184000
C	-0.107154000	-1.246423000	-2.135870000
C	-0.150309000	-1.374345000	-3.535864000
C	-0.196387000	-0.194179000	-4.323151000
H	-0.230569000	1.989258000	-4.344008000
H	-0.146208000	-2.364189000	-4.008298000
O	-0.054932000	-2.385948000	-1.354580000
O	-0.146432000	2.418694000	-1.710603000
P	-0.025293000	-2.109105000	0.363485000
P	-0.100099000	2.391269000	0.025699000
Ir	-0.074187000	0.173336000	0.577154000
H	1.436669000	0.215780000	0.869530000
H	-0.100624000	0.317825000	2.235940000
C	1.546256000	-3.062040000	0.798103000
C	-1.637261000	-2.989368000	0.829519000
C	1.435486000	3.447975000	0.320290000
C	-1.745555000	3.252312000	0.386513000
C	1.873103000	-2.806916000	2.284745000
H	2.849281000	-3.280262000	2.520121000
H	1.945368000	-1.724081000	2.498829000
H	1.117088000	-3.232195000	2.968400000
C	-1.619104000	-3.312281000	2.335708000
H	-1.343086000	-2.430771000	2.944483000
H	-2.633041000	-3.640427000	2.646765000
H	-0.916285000	-4.132321000	2.574563000
C	2.658658000	-2.449142000	-0.086096000
H	2.472157000	-2.635140000	-1.160357000
H	2.745520000	-1.355902000	0.068002000
H	3.627663000	-2.915007000	0.187907000
C	1.427088000	-4.569539000	0.506626000
H	2.417440000	-5.045381000	0.665621000
H	0.707136000	-5.065274000	1.184285000
H	1.122153000	-4.758091000	-0.540546000
C	-1.926088000	-4.248310000	-0.009665000
H	-1.211195000	-5.064329000	0.196165000
H	-2.941243000	-4.617856000	0.247339000

H	-1.902297000	-4.024608000	-1.092300000
C	-2.720111000	-1.920188000	0.539132000
H	-2.563394000	-1.018347000	1.168548000
H	-2.722469000	-1.615010000	-0.525819000
H	-3.722449000	-2.330107000	0.782210000
C	1.766658000	3.403781000	1.828120000
H	1.874148000	2.360810000	2.183152000
H	2.725343000	3.936173000	1.999285000
H	0.991156000	3.899021000	2.441413000
C	-1.756732000	3.703700000	1.860667000
H	-2.785846000	4.014821000	2.136552000
H	-1.452961000	2.879944000	2.537282000
H	-1.087831000	4.568662000	2.031412000
C	2.568121000	2.758844000	-0.478087000
H	2.687459000	1.697995000	-0.183412000
H	2.377207000	2.797606000	-1.566880000
H	3.522017000	3.285702000	-0.269679000
C	1.265959000	4.898715000	-0.167863000
H	0.538334000	5.459006000	0.448641000
H	2.242416000	5.420933000	-0.089085000
H	0.943368000	4.936554000	-1.225975000
C	-2.070502000	4.419304000	-0.563928000
H	-3.112852000	4.751807000	-0.374484000
H	-1.408205000	5.288293000	-0.404993000
H	-1.994195000	4.106189000	-1.621921000
C	-2.782246000	2.119111000	0.181566000
H	-2.769675000	1.732717000	-0.856164000
H	-2.589512000	1.274413000	0.877799000
H	-3.801069000	2.503352000	0.395615000
H	-0.939608000	-1.403377000	6.797620000
C	-0.052584000	-1.667748000	6.196903000
H	0.886708000	-1.461127000	6.737783000
Cl	-0.120509000	-3.437927000	5.869092000
Cl	-0.068566000	-0.685202000	4.693068000
C	-0.245608000	-0.246967000	-5.813631000
O	-0.286188000	0.735786000	-6.551667000
O	-0.241512000	-1.522272000	-6.292411000
C	-0.287596000	-1.654967000	-7.726087000
H	-1.210613000	-1.196225000	-8.134282000
H	0.590088000	-1.166381000	-8.195075000
H	-0.276461000	-2.740386000	-7.929929000

72

(p-MeO-POCOP)IrH2 S CH_2Cl_2, E = -1914.035706

C	-0.358049000	1.196259000	-3.610250000
C	-0.235143000	1.186116000	-2.211990000
C	-0.171398000	-0.024910000	-1.466778000
C	-0.234957000	-1.241815000	-2.192470000
C	-0.356178000	-1.281639000	-3.597406000
C	-0.417324000	-0.048284000	-4.293698000
H	-0.407753000	2.133082000	-4.180834000
H	-0.400619000	-2.247305000	-4.113296000
O	-0.177412000	-2.434525000	-1.507898000

O	-0.172734000	2.385784000	-1.539508000
P	-0.026543000	-2.267533000	0.225837000
P	-0.020174000	2.240429000	0.194504000
Ir	0.007444000	-0.011286000	0.594011000
H	0.964079000	0.012513000	1.884014000
H	-0.687050000	-0.013924000	2.045438000
C	1.601390000	-3.205159000	0.469518000
C	-1.579247000	-3.231350000	0.728164000
C	1.599123000	3.201406000	0.409256000
C	-1.579742000	3.187135000	0.704843000
C	1.979665000	-3.144012000	1.965174000
H	2.995915000	-3.571181000	2.095231000
H	1.991606000	-2.099114000	2.330974000
H	1.286268000	-3.728897000	2.597632000
C	-1.523529000	-3.492338000	2.247126000
H	-1.288278000	-2.567258000	2.810542000
H	-2.513934000	-3.859307000	2.588388000
H	-0.773895000	-4.264720000	2.504903000
C	2.637841000	-2.398817000	-0.351354000
H	2.417970000	-2.441863000	-1.435099000
H	2.649532000	-1.334775000	-0.041615000
H	3.645445000	-2.831909000	-0.181663000
C	1.562343000	-4.661292000	-0.027814000
H	2.594559000	-5.070409000	-0.021169000
H	0.947534000	-5.304698000	0.628614000
H	1.174351000	-4.729237000	-1.061955000
C	-1.781451000	-4.544033000	-0.053708000
H	-1.044704000	-5.317970000	0.222998000
H	-2.790342000	-4.943700000	0.181286000
H	-1.724215000	-4.373926000	-1.145093000
C	-2.746273000	-2.265752000	0.404118000
H	-2.641936000	-1.310636000	0.954715000
H	-2.787222000	-2.032751000	-0.677824000
H	-3.703737000	-2.747832000	0.692607000
C	1.819127000	3.463204000	1.913078000
H	1.687306000	2.538799000	2.510212000
H	2.855635000	3.828117000	2.069643000
H	1.130225000	4.237283000	2.301633000
C	-1.684241000	3.162825000	2.244808000
H	-2.663966000	3.590163000	2.544264000
H	-1.622860000	2.127490000	2.632458000
H	-0.893285000	3.766019000	2.727970000
C	2.687022000	2.233153000	-0.118528000
H	2.681281000	1.278463000	0.442528000
H	2.532968000	1.999918000	-1.190129000
H	3.681586000	2.713410000	-0.006612000
C	1.659206000	4.512746000	-0.398022000
H	0.988964000	5.289607000	0.008962000
H	2.695598000	4.908575000	-0.353400000
H	1.401334000	4.342309000	-1.460025000
C	-1.635795000	4.630305000	0.172226000
H	-2.653256000	5.038300000	0.348973000

H	-0.919567000	5.291402000	0.694320000
H	-1.434839000	4.671905000	-0.915179000
C	-2.743359000	2.361964000	0.101683000
H	-2.718600000	2.375887000	-1.004637000
H	-2.697538000	1.306366000	0.436280000
H	-3.706166000	2.801673000	0.435308000
O	-0.535660000	0.043015000	-5.650932000
C	-0.601401000	-1.162796000	-6.416248000
H	0.317648000	-1.774861000	-6.291623000
H	-1.485098000	-1.775481000	-6.136033000
H	-0.692664000	-0.850686000	-7.472832000

72

(p-MeO-POCOP)IrH2 NS CH_2Cl_2, E = -1914.033484

Ir	0.780223000	-0.940486000	-0.013040000
P	2.301101000	0.765977000	0.016980000
P	-1.220608000	-2.045219000	0.007148000
O	1.338967000	2.223765000	0.031755000
C	4.100651000	2.338384000	1.624727000
O	-2.428193000	-0.781330000	0.008234000
C	3.780738000	2.551449000	-1.707454000
C	-3.167386000	-3.437272000	1.611292000
C	-0.881712000	3.122956000	0.026014000
C	-1.034045000	-4.286905000	-1.657825000
C	-2.820246000	1.579569000	0.011221000
C	-0.723740000	-4.084509000	1.854911000
C	-3.282098000	-3.097828000	-1.722253000
C	-1.916297000	0.495697000	0.005422000
C	3.278068000	1.037349000	1.615707000
C	-2.282536000	2.890329000	0.020890000
C	-1.534919000	-1.855494000	2.711684000
C	-0.510394000	0.677228000	0.004697000
C	4.185842000	-0.189815000	1.847732000
C	2.256409000	0.815276000	-2.704669000
C	-1.715158000	-2.926945000	1.608239000
C	4.441826000	0.095543000	-1.656512000
C	-0.021108000	2.013222000	0.018730000
C	-1.757013000	-2.926755000	-1.582935000
C	-1.246229000	-1.999989000	-2.714364000
C	3.276002000	1.101560000	-1.574017000
C	2.198807000	1.092132000	2.724799000
H	4.972853000	2.279240000	0.947347000
H	3.484289000	3.212273000	1.339914000
H	4.488463000	2.511267000	2.650696000
H	2.960247000	3.276998000	-1.553566000
H	4.594667000	2.782600000	-0.998519000
H	4.180904000	2.693678000	-2.733442000
H	-3.878952000	-2.642778000	1.316097000
H	-3.301308000	-4.305853000	0.939945000
H	-3.429191000	-3.768651000	2.638221000
H	-0.500415000	4.152520000	0.036621000
H	0.052364000	-4.180136000	-1.465689000
H	-1.452010000	-5.016072000	-0.937598000

H	-1.163022000	-4.710534000	-2.675677000
H	-3.898945000	1.386605000	0.012290000
H	0.323252000	-3.725232000	1.835722000
H	-0.924943000	-4.521054000	2.855476000
H	-0.829416000	-4.893362000	1.108347000
H	-3.691096000	-3.835375000	-1.010103000
H	-3.807840000	-2.135661000	-1.576735000
H	-3.505124000	-3.463165000	-2.746841000
H	-0.509881000	-1.435174000	2.693988000
H	-2.252293000	-1.022292000	2.586182000
H	-1.713606000	-2.325490000	3.701072000
H	4.994769000	-0.260543000	1.097144000
H	4.660195000	-0.100389000	2.847326000
H	3.601370000	-1.129673000	1.822093000
H	1.388130000	1.500460000	-2.647788000
H	2.753398000	0.963934000	-3.686191000
H	1.875442000	-0.223320000	-2.646547000
H	4.096833000	-0.941370000	-1.470788000
H	4.883024000	0.133884000	-2.674385000
H	5.245762000	0.336751000	-0.934923000
H	-0.148585000	-1.863434000	-2.654664000
H	-1.501769000	-2.452308000	-3.695478000
H	-1.717690000	-0.999326000	-2.659744000
H	1.545378000	1.977705000	2.609096000
H	1.560871000	0.186355000	2.704012000
H	2.701483000	1.154604000	3.712276000
H	1.574949000	-1.940843000	0.935761000
H	1.693365000	-2.079909000	-0.720255000
O	-3.056024000	4.019643000	0.027463000
C	-4.472891000	3.873483000	0.007897000
H	-4.818931000	3.338008000	-0.903259000
H	-4.843920000	3.329083000	0.903854000
H	-4.889126000	4.898190000	0.007526000

77

(p-MeO-POCOP)IrH2---CH_2Cl_2 dihydrogen bonded, E = -2873.262972

C	-0.110430000	1.180941000	-3.604086000
C	-0.142476000	1.172196000	-2.200565000
C	-0.154266000	-0.038050000	-1.452388000
C	-0.124250000	-1.255864000	-2.177891000
C	-0.091335000	-1.297208000	-3.587942000
C	-0.086570000	-0.064548000	-4.288268000
H	-0.100588000	2.116912000	-4.178264000
H	-0.067732000	-2.263125000	-4.104846000
O	-0.117780000	-2.447157000	-1.487114000
O	-0.153080000	2.372406000	-1.524098000
P	-0.183927000	-2.277913000	0.252881000
P	-0.170205000	2.225364000	0.217459000
Ir	-0.227246000	-0.023004000	0.614539000
H	0.810690000	-0.029472000	1.802343000
H	-0.893933000	-0.029564000	2.101410000
C	1.395469000	-3.212759000	0.710484000
C	-1.787727000	-3.241581000	0.558459000

C	1.433148000	3.151010000	0.611834000
C	-1.754282000	3.208564000	0.559318000
C	1.577599000	-3.147154000	2.241845000
H	2.565899000	-3.581673000	2.500914000
H	1.551258000	-2.105757000	2.611004000
H	0.804835000	-3.722080000	2.783702000
C	-1.897889000	-3.542159000	2.066844000
H	-1.699672000	-2.640751000	2.679258000
H	-2.926230000	-3.894656000	2.291834000
H	-1.195527000	-4.339011000	2.377831000
C	2.533282000	-2.415391000	0.027008000
H	2.450607000	-2.455879000	-1.075856000
H	2.515261000	-1.352257000	0.337480000
H	3.507580000	-2.857847000	0.321388000
C	1.409806000	-4.670546000	0.216140000
H	2.426578000	-5.090351000	0.367100000
H	0.702501000	-5.304373000	0.782861000
H	1.167736000	-4.738949000	-0.861653000
C	-1.916638000	-4.529609000	-0.277353000
H	-1.206133000	-5.312168000	0.040875000
H	-2.942130000	-4.934913000	-0.146218000
H	-1.760756000	-4.327041000	-1.353403000
C	-2.903493000	-2.252228000	0.138824000
H	-2.853283000	-1.318474000	0.733324000
H	-2.821517000	-1.980828000	-0.931951000
H	-3.892316000	-2.729867000	0.301691000
C	1.679148000	3.079654000	2.133735000
H	1.709488000	2.032567000	2.487121000
H	2.661215000	3.545456000	2.359403000
H	0.909781000	3.623625000	2.712446000
C	-1.826426000	3.505546000	2.070950000
H	-2.841312000	3.879344000	2.320459000
H	-1.641068000	2.592608000	2.671979000
H	-1.099437000	4.283454000	2.373240000
C	2.538439000	2.350038000	-0.118944000
H	2.515045000	1.282057000	0.174860000
H	2.422427000	2.410180000	-1.217735000
H	3.527364000	2.774343000	0.152282000
C	1.435702000	4.610424000	0.122542000
H	0.760365000	5.247766000	0.723336000
H	2.461660000	5.021834000	0.227357000
H	1.144809000	4.684125000	-0.942659000
C	-1.889458000	4.500178000	-0.269850000
H	-2.912330000	4.907753000	-0.126243000
H	-1.173454000	5.279863000	0.042706000
H	-1.746691000	4.300142000	-1.348237000
C	-2.890117000	2.231972000	0.164016000
H	-2.838829000	1.968013000	-0.910457000
H	-2.831747000	1.293614000	0.750760000
H	-3.869923000	2.716937000	0.356734000
H	-0.056549000	0.616541000	3.839401000
C	0.367393000	0.230366000	4.783945000

H	0.217842000	0.908245000	5.641062000
Cl	-0.453924000	-1.323113000	5.145000000
Cl	2.137305000	0.035754000	4.533373000
O	-0.058193000	0.025296000	-5.651289000
C	-0.031168000	-1.181326000	-6.416839000
H	0.874877000	-1.785323000	-6.194646000
H	-0.933801000	-1.802486000	-6.231164000
H	-0.012599000	-0.871354000	-7.477888000

74

(m-bis-CF3-POCOP)IrH2 S CH_2Cl_2, E = -2473.012401

C	-0.335500000	1.216599000	-3.593898000
C	-0.206143000	1.205024000	-2.178087000
C	-0.160238000	-0.010762000	-1.457264000
C	-0.231319000	-1.229607000	-2.170875000
C	-0.334261000	-1.246977000	-3.588841000
C	-0.390857000	-0.016610000	-4.272725000
H	-0.480452000	-0.018847000	-5.365911000
O	-0.197848000	-2.408020000	-1.494812000
O	-0.127678000	2.386336000	-1.510735000
P	-0.031675000	-2.264293000	0.247798000
P	-0.011205000	2.250162000	0.236315000
Ir	0.008147000	-0.006272000	0.619326000
H	0.923488000	0.009767000	1.934647000
H	-0.654773000	-0.014151000	2.081383000
C	1.598925000	-3.204137000	0.433868000
C	-1.586141000	-3.229778000	0.728561000
C	1.599069000	3.218083000	0.457662000
C	-1.592194000	3.189920000	0.677371000
C	2.011527000	-3.162844000	1.920933000
H	3.021726000	-3.610727000	2.023287000
H	2.053804000	-2.121855000	2.295900000
H	1.321105000	-3.739841000	2.564056000
C	-1.513129000	-3.541032000	2.237380000
H	-1.274061000	-2.635610000	2.830671000
H	-2.499536000	-3.920497000	2.575738000
H	-0.759791000	-4.320615000	2.460425000
C	2.613683000	-2.389760000	-0.406975000
H	2.374557000	-2.438883000	-1.486343000
H	2.628122000	-1.325292000	-0.097666000
H	3.626240000	-2.817104000	-0.254894000
C	1.538603000	-4.651199000	-0.087134000
H	2.567958000	-5.066730000	-0.102662000
H	0.928566000	-5.301113000	0.567213000
H	1.136375000	-4.694668000	-1.117142000
C	-1.804253000	-4.512380000	-0.098832000
H	-1.066410000	-5.298278000	0.137647000
H	-2.810658000	-4.915156000	0.140529000
H	-1.765240000	-4.302750000	-1.183838000
C	-2.747521000	-2.244313000	0.445828000
H	-2.632863000	-1.306925000	1.024406000
H	-2.799121000	-1.981017000	-0.628791000
H	-3.705348000	-2.727886000	0.729569000

C	1.769094000	3.534150000	1.957650000
H	1.628425000	2.630881000	2.584732000
H	2.796769000	3.914747000	2.132228000
H	1.060765000	4.314501000	2.295596000
C	-1.757671000	3.161752000	2.211999000
H	-2.741587000	3.603743000	2.472992000
H	-1.729521000	2.124434000	2.598423000
H	-0.976999000	3.750149000	2.729217000
C	2.701283000	2.232811000	-0.005194000
H	2.682583000	1.297367000	0.587217000
H	2.579593000	1.965808000	-1.073241000
H	3.691457000	2.718528000	0.118950000
C	1.679064000	4.498188000	-0.397801000
H	0.987634000	5.283870000	-0.047670000
H	2.710166000	4.902946000	-0.324684000
H	1.466313000	4.285164000	-1.461783000
C	-1.623069000	4.631677000	0.139312000
H	-2.642696000	5.044774000	0.288301000
H	-0.916694000	5.289991000	0.678041000
H	-1.394928000	4.664724000	-0.943026000
C	-2.727449000	2.365538000	0.020193000
H	-2.666157000	2.403144000	-1.084095000
H	-2.688974000	1.304373000	0.338671000
H	-3.703313000	2.793446000	0.329350000
C	-0.374924000	-2.559469000	-4.327555000
F	-0.448699000	-2.391793000	-5.677471000
F	0.736812000	-3.321418000	-4.085323000
F	-1.450232000	-3.326747000	-3.968176000
C	-0.416010000	2.526152000	-4.334570000
F	0.703408000	3.294376000	-4.158159000
F	-0.563034000	2.353182000	-5.677776000
F	-1.473529000	3.289500000	-3.917353000

74

(m-bis-CF3-POCOP)IrH2 NS CH_2Cl_2, E = -2473.014608

C	-0.123989000	1.229857000	-3.615423000
C	-0.054676000	1.211666000	-2.196787000
C	-0.024548000	0.000108000	-1.470961000
C	-0.052948000	-1.211224000	-2.197267000
C	-0.122083000	-1.228837000	-3.615955000
C	-0.156343000	0.000644000	-4.304631000
H	-0.214049000	0.000841000	-5.399473000
O	-0.026603000	-2.395827000	-1.515175000
O	-0.029840000	2.396068000	-1.514242000
P	-0.003891000	-2.258062000	0.224137000
P	-0.008165000	2.257742000	0.224903000
Ir	0.027193000	-0.000235000	0.611192000
H	0.109973000	0.001126000	2.279346000
H	-1.481134000	-0.001557000	0.912407000
C	1.636416000	-3.139718000	0.551321000
C	-1.553873000	-3.264189000	0.602408000
C	1.629631000	3.143329000	0.553498000
C	-1.560861000	3.259958000	0.602450000

C	1.692696000	-3.544479000	2.037995000
H	2.722476000	-3.878222000	2.282328000
H	1.442727000	-2.692801000	2.701982000
H	1.006111000	-4.382788000	2.262520000
C	-1.846719000	-3.142254000	2.113229000
H	-1.942949000	-2.082058000	2.417507000
H	-2.803523000	-3.658747000	2.334880000
H	-1.058667000	-3.610815000	2.731774000
C	2.688869000	-2.038464000	0.266978000
H	2.642606000	-1.686662000	-0.782183000
H	2.548702000	-1.167878000	0.943242000
H	3.705977000	-2.442358000	0.450004000
C	1.897848000	-4.344157000	-0.373236000
H	2.938726000	-4.693412000	-0.208529000
H	1.223157000	-5.191542000	-0.160068000
H	1.788384000	-4.065635000	-1.437511000
C	-1.419852000	-4.737771000	0.174514000
H	-0.683522000	-5.283918000	0.793190000
H	-2.402219000	-5.237634000	0.305175000
H	-1.131470000	-4.823788000	-0.890588000
C	-2.691089000	-2.595137000	-0.207493000
H	-2.786683000	-1.517855000	0.030571000
H	-2.527051000	-2.700434000	-1.296144000
H	-3.648462000	-3.091428000	0.052734000
C	1.684129000	3.546187000	2.040739000
H	1.436037000	2.692814000	2.703235000
H	2.712841000	3.882430000	2.286125000
H	0.994977000	4.382181000	2.266036000
C	-1.853933000	3.137850000	2.113205000
H	-2.811976000	3.652254000	2.334367000
H	-1.947886000	2.077551000	2.417818000
H	-1.067109000	3.608512000	2.731746000
C	2.684956000	2.045059000	0.268154000
H	2.546627000	1.173226000	0.943145000
H	2.640008000	1.694574000	-0.781513000
H	3.700959000	2.451262000	0.452151000
C	1.888483000	4.349580000	-0.369454000
H	1.212845000	5.195815000	-0.154530000
H	2.929003000	4.700045000	-0.205049000
H	1.778716000	4.072364000	-1.434054000
C	-1.430003000	4.733693000	0.174211000
H	-2.413461000	5.231496000	0.304522000
H	-0.694980000	5.281368000	0.793030000
H	-1.141508000	4.820082000	-0.890834000
C	-2.696153000	2.588089000	-0.207773000
H	-2.531516000	2.693032000	-1.296375000
H	-2.789727000	1.510753000	0.030802000
H	-3.654713000	3.082603000	0.051450000
C	-0.174588000	-2.540336000	-4.354356000
F	-0.263790000	-2.373449000	-5.703841000
F	0.935067000	-3.311467000	-4.129069000
F	-1.250202000	-3.302913000	-3.981931000

C	-0.178561000	2.541726000	-4.353061000
F	0.929972000	3.314318000	-4.127352000
F	-0.267509000	2.375429000	-5.702667000
F	-1.255287000	3.302409000	-3.980178000

79

(m-bis-CF3-POCOP)IrH2-CH_2Cl_2 dihydrogen bonded, E = -3432.241343

C	0.073036000	1.166330000	-3.568862000
C	-0.061833000	1.164045000	-2.154674000
C	-0.124386000	-0.040275000	-1.418323000
C	-0.079618000	-1.260080000	-2.129976000
C	0.055106000	-1.292822000	-3.543869000
C	0.130284000	-0.070747000	-4.242277000
O	-0.154383000	-2.436029000	-1.439725000
O	-0.119969000	2.354477000	-1.487840000
P	-0.240954000	-2.281880000	0.298777000
P	-0.225933000	2.232969000	0.252362000
Ir	-0.301861000	-0.018981000	0.657864000
H	1.134011000	-0.021284000	1.218973000
H	-0.587792000	-0.022449000	2.292239000
C	1.300748000	-3.280283000	0.728487000
C	-1.886591000	-3.169574000	0.575653000
C	1.317689000	3.229967000	0.680314000
C	-1.869414000	3.138444000	0.488249000
C	1.511776000	-3.223308000	2.254435000
H	2.473126000	-3.720574000	2.500164000
H	1.562584000	-2.181855000	2.618571000
H	0.710897000	-3.743097000	2.810739000
C	-1.990846000	-3.549175000	2.066979000
H	-1.730349000	-2.697144000	2.726589000
H	-3.034707000	-3.852197000	2.290811000
H	-1.332149000	-4.402331000	2.317681000
C	2.468008000	-2.557083000	0.012907000
H	2.361166000	-2.605437000	-1.087088000
H	2.536658000	-1.493825000	0.313932000
H	3.417149000	-3.057994000	0.293793000
C	1.212774000	-4.734279000	0.227889000
H	2.197837000	-5.222515000	0.380734000
H	0.461283000	-5.318879000	0.790501000
H	0.971980000	-4.777667000	-0.851428000
C	-2.107401000	-4.390445000	-0.337717000
H	-1.435159000	-5.229690000	-0.087589000
H	-3.151190000	-4.744631000	-0.204893000
H	-1.961738000	-4.127844000	-1.401880000
C	-2.935420000	-2.080881000	0.234197000
H	-2.828901000	-1.200248000	0.902953000
H	-2.847365000	-1.742598000	-0.816993000
H	-3.955699000	-2.492545000	0.378282000
C	1.542542000	3.150086000	2.204351000
H	1.688148000	2.104443000	2.530625000
H	2.462498000	3.715801000	2.459596000
H	0.706311000	3.587663000	2.780655000
C	-1.999354000	3.537061000	1.972026000

H -3.037389000 3.878524000 2.165375000
H -1.791050000 2.679093000 2.642617000
H -1.317331000 4.367799000 2.235716000
C 2.482244000 2.521391000 -0.053740000
H 2.538083000 1.447217000 0.209947000
H 2.379902000 2.608541000 -1.151515000
H 3.435005000 3.002191000 0.249080000
C 1.222633000 4.691636000 0.204672000
H 0.474871000 5.266565000 0.782114000
H 2.208176000 5.179392000 0.355779000
H 0.972671000 4.751065000 -0.871745000
C -2.065784000 4.349725000 -0.443400000
H -3.112653000 4.704908000 -0.341133000
H -1.400602000 5.191886000 -0.184828000
H -1.893429000 4.075575000 -1.500561000
C -2.920378000 2.053364000 0.141859000
H -2.820777000 1.706112000 -0.905219000
H -2.828122000 1.177242000 0.818659000
H -3.939668000 2.472553000 0.270242000
H 0.352694000 0.507572000 3.816755000
C 0.943923000 0.173582000 4.690453000
H 0.955967000 0.899575000 5.520773000
Cl 0.200832000 -1.348171000 5.283328000
Cl 2.638221000 -0.048933000 4.125087000
H 0.238654000 -0.082640000 -5.333280000
C 0.130957000 -2.612836000 -4.264897000
F 0.287564000 -2.461688000 -5.610109000
F 1.180265000 -3.380293000 -3.831829000
F -0.995225000 -3.371574000 -4.085572000
C 0.166821000 2.470011000 -4.316916000
F 1.226608000 3.231919000 -3.900249000
F 0.320333000 2.289130000 -5.658808000
F -0.948911000 3.247740000 -4.152932000
72
(PCP)IrH2 tilted S CH_2Cl_2, E = -1727.852204
Ir -0.000856000 -0.488720000 0.003541000
H 0.013170000 -1.907811000 0.757666000
H -0.016029000 -1.917641000 -0.731555000
C -0.000216000 1.625815000 -0.011120000
P -2.283410000 -0.187224000 -0.101121000
P 2.282022000 -0.187148000 0.104157000
C -2.462265000 1.600202000 -0.641713000
C -3.268389000 -1.146463000 -1.427165000
C -3.128559000 -0.236394000 1.615194000
C 2.461755000 1.607492000 0.619989000
C 3.266703000 -1.128670000 1.443026000
C 3.127216000 -0.260314000 -1.611311000
C -1.193893000 2.360585000 -0.298505000
C -1.188879000 3.773271000 -0.301086000
C 0.000612000 4.476810000 -0.030794000
C 1.193874000 2.363785000 0.266099000
C 1.189683000 3.776372000 0.249184000

H	-2.114475000	4.328683000	-0.523295000
H	0.000927000	5.578391000	-0.038375000
H	2.115591000	4.334254000	0.463778000
H	-2.593155000	1.578446000	-1.743473000
H	-3.374062000	2.079538000	-0.231680000
H	2.592357000	1.601000000	1.721987000
H	3.373870000	2.080552000	0.203444000
C	-3.445324000	-2.608161000	-0.970203000
C	-2.372543000	-1.118912000	-2.689418000
C	-4.635838000	-0.520310000	-1.771488000
C	-2.791999000	-1.581216000	2.295844000
C	-4.652179000	-0.024124000	1.571563000
C	-2.469317000	0.897240000	2.436825000
C	2.791337000	-1.614794000	-2.272806000
C	2.467384000	0.861195000	-2.448952000
C	4.650742000	-0.046610000	-1.570654000
C	3.444282000	-2.596224000	1.005437000
C	4.633928000	-0.497744000	1.779452000
C	2.370424000	-1.084649000	2.704467000
H	-2.837934000	0.842362000	3.482891000
H	-1.366526000	0.790448000	2.440137000
H	-2.715040000	1.901653000	2.042257000
H	-4.931301000	0.896588000	1.022380000
H	-5.034911000	0.077393000	2.609594000
H	-5.175634000	-0.882211000	1.109730000
H	-3.238035000	-2.444609000	1.769327000
H	-3.189299000	-1.574160000	3.333043000
H	-1.695302000	-1.728287000	2.335922000
H	-5.346276000	-0.561464000	-0.928029000
H	-5.086527000	-1.085046000	-2.615427000
H	-4.540962000	0.534151000	-2.095213000
H	-2.211452000	-0.087089000	-3.060369000
H	-1.379699000	-1.562519000	-2.482786000
H	-2.867092000	-1.695336000	-3.499811000
H	-3.830438000	-3.215228000	-1.816553000
H	-4.172115000	-2.695356000	-0.140264000
H	-2.481182000	-3.045823000	-0.643524000
H	2.836575000	0.792088000	-3.493972000
H	1.364695000	0.753344000	-2.451268000
H	2.711968000	1.871217000	-2.068262000
H	3.238521000	-2.470363000	-1.734619000
H	3.187863000	-1.621876000	-3.310299000
H	1.694740000	-1.763365000	-2.310014000
H	4.929310000	0.881854000	-1.034395000
H	5.033415000	0.040621000	-2.610003000
H	5.174729000	-0.897837000	-1.096860000
H	3.829171000	-3.191913000	1.859935000
H	4.171583000	-2.694043000	0.177134000
H	2.480539000	-3.038652000	0.684017000
H	2.209214000	-0.048047000	3.061806000
H	1.377640000	-1.530850000	2.503174000
H	2.864626000	-1.650462000	3.522516000

H	5.344479000	-0.549532000	0.936681000
H	5.084657000	-1.051459000	2.630643000
H	4.538742000	0.560755000	2.089606000

72

(PCP)IrH2 tilted NS CH_2Cl_2, E = -1727.85312

Ir	-0.163538000	-0.581425000	-0.222742000
H	-0.191058000	-0.454394000	-1.753654000
H	-0.173715000	-2.264434000	-0.400192000
C	-0.147483000	1.507964000	-0.025839000
P	-2.446707000	-0.270461000	-0.139299000
P	2.128061000	-0.307670000	-0.171793000
C	-2.636201000	1.551182000	-0.527168000
C	-3.592804000	-1.127088000	-1.400863000
C	-3.080973000	-0.455095000	1.657102000
C	2.336966000	1.433422000	0.493815000
C	2.984700000	-1.391275000	1.142426000
C	3.028250000	-0.291838000	-1.848216000
C	-1.347181000	2.277260000	-0.179677000
C	-1.335557000	3.682214000	-0.043632000
C	-0.131036000	4.361559000	0.227481000
C	1.060499000	2.223482000	0.258949000
C	1.064307000	3.631225000	0.375072000
H	-2.271201000	4.253739000	-0.160276000
H	-0.124626000	5.458715000	0.324406000
H	2.005871000	4.161820000	0.593461000
H	-2.813172000	1.607065000	-1.622266000
H	-3.529533000	1.996693000	-0.044579000
H	2.513036000	1.331345000	1.584849000
H	3.239047000	1.932032000	0.085906000
C	-3.815377000	-2.594969000	-0.986665000
C	-2.820241000	-1.101936000	-2.741114000
C	-4.943884000	-0.403983000	-1.585785000
C	-2.685955000	-1.854462000	2.180438000
C	-4.596451000	-0.232810000	1.807325000
C	-2.325111000	0.604955000	2.494059000
C	2.671291000	-1.579232000	-2.622025000
C	2.464359000	0.925424000	-2.619457000
C	4.555462000	-0.153352000	-1.707756000
C	3.153615000	-2.823616000	0.599485000
C	4.333516000	-0.844288000	1.649315000
C	1.960728000	-1.410687000	2.305337000
H	-2.602117000	0.479365000	3.561728000
H	-1.226478000	0.491657000	2.405445000
H	-2.578240000	1.638886000	2.193279000
H	-4.916176000	0.743032000	1.391887000
H	-4.859126000	-0.238090000	2.886713000
H	-5.183752000	-1.033506000	1.320615000
H	-3.215362000	-2.668076000	1.653583000
H	-2.938124000	-1.925628000	3.259537000
H	-1.597478000	-2.029936000	2.065342000
H	-5.547872000	-0.386534000	-0.661987000
H	-5.532669000	-0.938931000	-2.361001000

H	-4.811414000	0.638182000	-1.934370000
H	-2.568489000	-0.070177000	-3.058653000
H	-1.879362000	-1.679001000	-2.665721000
H	-3.453158000	-1.554167000	-3.533368000
H	-4.306205000	-3.139766000	-1.820453000
H	-4.472365000	-2.681123000	-0.100625000
H	-2.852316000	-3.096843000	-0.766784000
H	2.875424000	0.917085000	-3.650589000
H	1.359850000	0.887792000	-2.683171000
H	2.746733000	1.886780000	-2.149882000
H	3.070788000	-2.485857000	-2.132248000
H	3.106296000	-1.523701000	-3.642163000
H	1.573965000	-1.693873000	-2.711229000
H	4.839357000	0.725324000	-1.095774000
H	5.003451000	-0.018607000	-2.715126000
H	5.010990000	-1.056580000	-1.260227000
H	3.446198000	-3.499616000	1.430442000
H	3.946077000	-2.879748000	-0.171013000
H	2.205789000	-3.196967000	0.164301000
H	1.783806000	-0.400842000	2.725962000
H	0.988181000	-1.827414000	1.962598000
H	2.336160000	-2.056892000	3.126323000
H	5.106818000	-0.839140000	0.860906000
H	4.699292000	-1.494315000	2.472639000
H	4.243498000	0.181747000	2.054882000

77

(PCP)IrH2-tilted-CH_2Cl_2, E = -2687.081246

Ir	-0.152798000	-0.556333000	-0.277066000
H	-0.204946000	-0.394860000	-1.805096000
H	-0.158631000	-2.222212000	-0.542151000
C	-0.145961000	1.526961000	-0.026562000
P	-2.437246000	-0.268477000	-0.139277000
P	2.136143000	-0.281176000	-0.223042000
C	-2.645914000	1.560696000	-0.477245000
C	-3.599160000	-1.103470000	-1.400812000
C	-3.039796000	-0.501631000	1.662196000
C	2.360033000	1.472742000	0.404967000
C	2.918103000	-1.347447000	1.148971000
C	3.076457000	-0.331067000	-1.873122000
C	-1.355995000	2.288696000	-0.136189000
C	-1.353563000	3.690153000	0.030892000
C	-0.148638000	4.374890000	0.286197000
C	1.064070000	2.248504000	0.233660000
C	1.057225000	3.653650000	0.381447000
H	-2.296859000	4.255221000	-0.049945000
H	-0.149748000	5.469533000	0.408367000
H	1.999877000	4.189076000	0.582729000
H	-2.841640000	1.642133000	-1.567611000
H	-3.534096000	1.987851000	0.030746000
H	2.608884000	1.397970000	1.483525000
H	3.227380000	1.973994000	-0.069273000
C	-3.798551000	-2.582792000	-1.019491000

C	-2.852438000	-1.038854000	-2.754336000
C	-4.959828000	-0.388748000	-1.543349000
C	-2.587680000	-1.893723000	2.153197000
C	-4.559925000	-0.334677000	1.835156000
C	-2.312034000	0.569039000	2.510347000
C	2.734980000	-1.646602000	-2.604955000
C	2.529147000	0.856082000	-2.701252000
C	4.599717000	-0.189235000	-1.697335000
C	3.026653000	-2.806657000	0.664155000
C	4.279299000	-0.851356000	1.671412000
C	1.861498000	-1.260528000	2.278926000
H	-2.556020000	0.400957000	3.580241000
H	-1.212325000	0.509006000	2.391744000
H	-2.621037000	1.597945000	2.246650000
H	-4.918400000	0.636783000	1.441744000
H	-4.806649000	-0.368000000	2.917771000
H	-5.126583000	-1.146344000	1.342358000
H	-3.041880000	-2.722495000	1.583577000
H	-2.867573000	-2.017645000	3.220512000
H	-1.489203000	-2.003617000	2.078624000
H	-5.550148000	-0.406716000	-0.610965000
H	-5.554631000	-0.905934000	-2.326015000
H	-4.843558000	0.665118000	-1.861472000
H	-2.615394000	0.002255000	-3.052054000
H	-1.905688000	-1.609416000	-2.710286000
H	-3.496536000	-1.477929000	-3.544970000
H	-4.312539000	-3.108881000	-1.851501000
H	-4.424707000	-2.700388000	-0.115150000
H	-2.829815000	-3.087238000	-0.841495000
H	2.969187000	0.816646000	-3.719583000
H	1.427005000	0.807339000	-2.796249000
H	2.791715000	1.834912000	-2.256745000
H	3.132287000	-2.532595000	-2.077099000
H	3.183531000	-1.626105000	-3.620468000
H	1.639208000	-1.768885000	-2.702944000
H	4.869972000	0.705861000	-1.102956000
H	5.075536000	-0.085073000	-2.695510000
H	5.040375000	-1.079701000	-1.210796000
H	3.221098000	-3.472588000	1.530469000
H	3.858074000	-2.935405000	-0.055005000
H	2.086491000	-3.132463000	0.179807000
H	1.741762000	-0.229039000	2.663862000
H	0.872862000	-1.624054000	1.919068000
H	2.155730000	-1.911982000	3.127039000
H	5.073120000	-0.927115000	0.906927000
H	4.584496000	-1.479780000	2.535194000
H	4.236095000	0.196818000	2.025120000
C	-0.291399000	-4.700729000	1.055154000
H	0.482414000	-5.356099000	0.621144000
H	-0.303458000	-3.694738000	0.583473000
Cl	-1.890740000	-5.470728000	0.767486000
Cl	0.074995000	-4.504239000	2.805048000

5
CH_2Cl_2 in CH_2Cl_2, E = -959.2127688

H	-0.939617000	-1.401214000	6.794319000
C	-0.052527000	-1.672344000	6.196918000
H	0.886753000	-1.460342000	6.735537000
Cl	-0.120108000	-3.438307000	5.871015000
Cl	-0.069060000	-0.683175000	4.696676000

74
(p-MeOOC-POCOP)IrH2 NS toluene DLPNO-SCS-MP2, E = -2021.994798

Ir	2.889272000	4.082108000	4.067828000
P	0.916949000	3.180694000	3.410620000
P	4.530129000	5.622240000	4.397209000
O	0.041566000	4.464841000	2.708965000
C	-0.567476000	1.489148000	1.665059000
O	3.958788000	7.047763000	3.653807000
C	-1.714593000	2.896748000	4.528433000
C	6.877435000	6.853705000	3.340508000
C	0.103596000	6.766823000	2.045015000
C	5.056877000	5.039918000	7.090652000
C	2.112132000	8.094509000	2.539095000
C	7.105207000	4.526089000	4.299287000
C	5.576597000	7.407936000	6.395581000
C	2.684947000	6.964267000	3.131253000
C	0.857432000	1.954678000	1.999120000
C	0.817899000	7.981036000	1.991617000
C	5.898624000	4.906091000	2.134275000
C	2.009881000	5.730176000	3.213549000
C	1.753344000	0.749022000	2.330428000
C	0.148077000	3.839937000	5.907329000
C	6.188665000	5.491288000	3.531257000
C	0.110229000	1.384142000	5.407952000
C	0.718979000	5.666339000	2.652853000
C	4.643341000	6.209501000	6.179610000
C	3.204448000	6.628659000	6.545121000
C	-0.217487000	2.777266000	4.849008000
C	1.437635000	2.689597000	0.773997000
H	-0.985449000	0.845906000	2.452163000
H	-1.241850000	2.343580000	1.503273000
H	-0.535697000	0.896433000	0.735278000
H	-1.954174000	3.882009000	4.104368000
H	-2.056517000	2.122248000	3.829970000
H	-2.282263000	2.776789000	5.466712000
H	6.234870000	7.549232000	2.783060000
H	7.159706000	7.322240000	4.290691000
H	7.801252000	6.692959000	2.759618000
H	-0.895919000	6.680523000	1.618293000
H	4.385914000	4.176672000	6.966324000
H	6.086525000	4.706036000	6.901262000
H	5.002026000	5.375891000	8.139738000
H	2.652415000	9.041594000	2.486312000
H	6.591438000	3.581201000	4.537269000
H	7.979013000	4.289864000	3.669552000

H	7.479327000	4.971317000	5.233176000
H	6.634538000	7.124936000	6.302242000
H	5.357959000	8.222849000	5.689653000
H	5.427505000	7.791945000	7.418996000
H	5.562878000	3.860835000	2.195826000
H	5.136608000	5.492641000	1.597094000
H	6.829833000	4.930732000	1.544402000
H	1.384320000	0.180439000	3.196244000
H	1.765099000	0.069940000	1.461617000
H	2.786159000	1.064086000	2.539923000
H	-0.018427000	4.862503000	5.533228000
H	-0.480497000	3.697395000	6.802355000
H	1.202967000	3.738267000	6.222200000
H	1.189983000	1.266251000	5.591591000
H	-0.416748000	1.254638000	6.368020000
H	-0.225878000	0.582274000	4.733757000
H	2.490807000	5.799318000	6.412073000
H	3.180399000	6.921070000	7.608460000
H	2.860993000	7.481871000	5.941434000
H	0.794085000	3.526457000	0.465793000
H	2.447669000	3.080287000	0.970440000
H	1.504837000	1.974343000	-0.062671000
H	3.586862000	3.559689000	2.823287000
H	3.590578000	2.752182000	4.761362000
C	0.237252000	9.196440000	1.349381000
O	0.784227000	10.280203000	1.306717000
O	-0.973987000	8.968220000	0.806711000
C	-1.587089000	10.098485000	0.178597000
H	-1.743931000	10.907804000	0.904650000
H	-0.962006000	10.470917000	-0.644443000
H	-2.548800000	9.741385000	-0.205154000

86

(p-MeOOC-POCOP)IrH2-IC6F5 toluene DLPNO-SCS-MP2, E = -2021.994798

Ir	2.897691000	4.066189000	4.041720000
P	0.915893000	3.167470000	3.388469000
P	4.540735000	5.610335000	4.385588000
O	0.038774000	4.461068000	2.712930000
C	-0.632912000	1.551010000	1.638144000
O	3.975813000	7.024998000	3.620737000
C	-1.687793000	2.898007000	4.573383000
C	6.841477000	6.873933000	3.280180000
C	0.107229000	6.760602000	2.047279000
C	5.017057000	5.045269000	7.090354000
C	2.125724000	8.078023000	2.521214000
C	7.176989000	4.642944000	4.411286000
C	5.551487000	7.410109000	6.383775000
C	2.700953000	6.944025000	3.103498000
C	0.811373000	1.958452000	1.965676000
C	0.823987000	7.972693000	1.991084000
C	5.992822000	4.789567000	2.206498000
C	2.028956000	5.707770000	3.175720000
C	1.664583000	0.717472000	2.271137000

C	0.219993000	3.819074000	5.905587000
C	6.221836000	5.493027000	3.559154000
C	0.148900000	1.365355000	5.393069000
C	0.725270000	5.655950000	2.644158000
C	4.620872000	6.209873000	6.164919000
C	3.177429000	6.634790000	6.504526000
C	-0.183807000	2.764628000	4.852710000
C	1.410938000	2.689332000	0.747426000
H	-1.064671000	0.908089000	2.417996000
H	-1.278339000	2.431179000	1.498961000
H	-0.630415000	0.974449000	0.697756000
H	-1.930966000	3.887819000	4.162076000
H	-2.054414000	2.130447000	3.879865000
H	-2.230680000	2.776455000	5.525929000
H	6.198191000	7.473500000	2.622282000
H	7.033317000	7.446033000	4.195935000
H	7.808391000	6.716707000	2.773096000
H	-0.900360000	6.680271000	1.638869000
H	4.349688000	4.180519000	6.955111000
H	6.050774000	4.711268000	6.926039000
H	4.937841000	5.385427000	8.136514000
H	2.667190000	9.024627000	2.472094000
H	6.721084000	3.685418000	4.704344000
H	8.075986000	4.417543000	3.813833000
H	7.504279000	5.174417000	5.316868000
H	6.610601000	7.134621000	6.281938000
H	5.323472000	8.227845000	5.684051000
H	5.405957000	7.787400000	7.410141000
H	5.700973000	3.738250000	2.344119000
H	5.219147000	5.298063000	1.609459000
H	6.935865000	4.811409000	1.635466000
H	1.279944000	0.144344000	3.126960000
H	1.651646000	0.055127000	1.389574000
H	2.708507000	0.989980000	2.480255000
H	0.052335000	4.844981000	5.541035000
H	-0.384655000	3.678995000	6.817346000
H	1.282005000	3.704793000	6.193088000
H	1.233656000	1.233967000	5.534989000
H	-0.342989000	1.236802000	6.371725000
H	-0.222090000	0.571471000	4.727872000
H	2.464061000	5.805949000	6.368983000
H	3.138171000	6.937640000	7.564433000
H	2.844391000	7.483022000	5.888197000
H	0.804043000	3.560606000	0.460640000
H	2.440727000	3.027219000	0.939292000
H	1.435776000	1.987054000	-0.102230000
I	5.231529000	0.890132000	4.349206000
F	9.534557000	-2.163290000	5.606551000
F	9.029633000	-4.011677000	3.667697000
F	7.884680000	-0.049808000	5.935456000
F	6.904255000	-3.686514000	1.986886000
F	5.235615000	-1.585430000	2.297345000

C	8.491558000	-2.002551000	4.790900000
C	7.610726000	-0.931497000	4.970099000
C	6.527187000	-0.733249000	4.104723000
C	6.272209000	-1.707779000	3.131106000
C	7.145183000	-2.783212000	2.938434000
C	8.215024000	-2.969281000	3.819066000
H	3.584038000	3.590685000	2.770680000
H	3.549443000	2.745804000	4.806467000
C	0.238806000	9.193109000	1.361801000
O	0.789217000	10.274896000	1.317341000
O	-0.979901000	8.970580000	0.834669000
C	-1.598430000	10.104908000	0.218760000
H	-1.743382000	10.911743000	0.949964000
H	-0.983191000	10.478483000	-0.611122000
H	-2.566003000	9.751535000	-0.153413000

12
IC6F5 toluene DLPNO-SCS-MP2, E = -1022.436628

I	5.255220000	0.877801000	4.346944000
F	9.531948000	-2.159738000	5.605907000
F	9.030501000	-4.005914000	3.665635000
F	7.878184000	-0.048020000	5.936956000
F	6.903453000	-3.685179000	1.988172000
F	5.231959000	-1.584875000	2.298005000
C	8.489231000	-2.001291000	4.792343000
C	7.610394000	-0.929402000	4.973334000
C	6.525845000	-0.738851000	4.105541000
C	6.267963000	-1.708659000	3.127393000
C	7.142580000	-2.783810000	2.939501000
C	8.214879000	-2.966218000	3.817738000

77
Cp*Ru(dppm)H2+ short CH_2Cl_2, E = -2133.492610

Ru	-1.514431000	0.183063000	1.064139000
C	-1.459561000	-1.606958000	-0.288874000
C	-2.745858000	-0.934597000	-0.422475000
C	-3.412983000	-0.974126000	0.854688000
C	-2.540222000	-1.674939000	1.794957000
C	-1.347689000	-2.077213000	1.073572000
C	-0.509876000	-1.916414000	-1.410483000
C	-3.320598000	-0.396344000	-1.701155000
C	-4.829424000	-0.555961000	1.123289000
C	-2.947560000	-2.100746000	3.177441000
C	-0.228433000	-2.924680000	1.602283000
C	-0.263850000	2.311873000	2.995849000
P	-2.026954000	1.831874000	2.600702000
C	-2.913821000	3.353572000	2.072944000
C	-2.931987000	4.483485000	2.923575000
C	-3.620538000	5.643387000	2.529876000
C	-4.306609000	5.675031000	1.297857000
C	-4.299602000	4.546872000	0.456538000
C	-3.599775000	3.387055000	0.841526000
C	-2.867116000	1.455322000	4.192648000
C	-4.280054000	1.362912000	4.179852000

C	-4.977425000	0.994992000	5.342606000
C	-4.273508000	0.723136000	6.532766000
C	-2.871661000	0.841371000	6.557875000
C	-2.168939000	1.209480000	5.394892000
P	0.423529000	0.778085000	2.162659000
C	1.905559000	1.302540000	1.222288000
C	2.774773000	2.295160000	1.729394000
C	3.921689000	2.658948000	1.000837000
C	4.208930000	2.029583000	-0.226753000
C	3.345769000	1.037304000	-0.730852000
C	2.192725000	0.676587000	-0.010304000
C	1.095512000	-0.294071000	3.496950000
C	0.177166000	-0.895952000	4.385732000
C	0.629941000	-1.722734000	5.426366000
C	2.008459000	-1.976864000	5.572010000
C	2.925947000	-1.398577000	4.674628000
C	2.475986000	-0.556069000	3.639704000
H	-2.410137000	4.455116000	3.892487000
H	-3.626121000	6.525676000	3.187514000
H	-4.849351000	6.583340000	0.995485000
H	-4.838177000	4.567253000	-0.502717000
H	-3.582435000	2.501929000	0.187890000
H	-4.837277000	1.594342000	3.261015000
H	-6.075225000	0.924559000	5.319599000
H	-4.819427000	0.432000000	7.442566000
H	-2.315718000	0.651178000	7.488173000
H	-1.076805000	1.305203000	5.441634000
H	2.561123000	2.782555000	2.692486000
H	4.594006000	3.436397000	1.393730000
H	5.107135000	2.316868000	-0.793854000
H	3.566296000	0.546624000	-1.690651000
H	1.505301000	-0.085248000	-0.405422000
H	-0.897050000	-0.728331000	4.245628000
H	-0.096046000	-2.179487000	6.115760000
H	2.366159000	-2.632440000	6.380124000
H	4.002434000	-1.602224000	4.777828000
H	3.201127000	-0.109045000	2.945941000
H	-1.648564000	1.506868000	0.099659000
H	-0.712828000	1.051064000	-0.075239000
H	-0.837796000	-2.834328000	-1.944005000
H	0.514083000	-2.101240000	-1.036372000
H	-0.466831000	-1.096041000	-2.150840000
H	-3.845388000	-1.208466000	-2.248114000
H	-4.055079000	0.408091000	-1.510305000
H	-2.532365000	0.001239000	-2.367345000
H	-5.527311000	-1.347841000	0.776216000
H	-5.007595000	-0.407625000	2.202292000
H	-5.094845000	0.377813000	0.593506000
H	-3.749893000	-2.865817000	3.111756000
H	-2.105795000	-2.551765000	3.731129000
H	-3.345371000	-1.256456000	3.771102000
H	-0.144942000	-2.860079000	2.700626000

H	0.745695000	-2.630599000	1.168813000
H	-0.409909000	-3.987130000	1.334715000
H	0.011675000	2.490483000	4.051670000
H	-0.001891000	3.201912000	2.390595000

77

Cp*Ru(dppm)H2+ long CH_2Cl_2, E = -2133.492724

Ru	-1.515559000	0.201395000	1.034687000
C	-1.460984000	-1.619484000	-0.296720000
C	-2.724638000	-0.921002000	-0.462014000
C	-3.417090000	-0.940007000	0.807730000
C	-2.585831000	-1.670577000	1.761092000
C	-1.387397000	-2.091809000	1.069604000
C	-0.491094000	-1.953307000	-1.393322000
C	-3.270503000	-0.390067000	-1.756290000
C	-4.832014000	-0.496983000	1.045070000
C	-3.030109000	-2.082716000	3.136351000
C	-0.293602000	-2.961759000	1.615010000
C	-0.254652000	2.347227000	2.937008000
P	-2.016432000	1.845416000	2.581187000
C	-2.941795000	3.355032000	2.088265000
C	-2.962317000	4.463399000	2.967233000
C	-3.676516000	5.621131000	2.615783000
C	-4.386625000	5.671743000	1.398256000
C	-4.377119000	4.564924000	0.529285000
C	-3.651279000	3.407658000	0.871390000
C	-2.812082000	1.447165000	4.190440000
C	-4.223762000	1.337913000	4.212904000
C	-4.886713000	0.956838000	5.391456000
C	-4.148964000	0.687930000	6.561590000
C	-2.748194000	0.820867000	6.550911000
C	-2.079792000	1.202355000	5.372328000
P	0.425953000	0.802525000	2.126784000
C	1.922420000	1.295589000	1.194853000
C	2.760055000	2.332080000	1.664813000
C	3.926405000	2.665476000	0.952091000
C	4.263277000	1.962806000	-0.221484000
C	3.430680000	0.926672000	-0.688001000
C	2.259303000	0.595101000	0.016022000
C	1.076054000	-0.259388000	3.481860000
C	0.142871000	-0.912288000	4.316829000
C	0.579725000	-1.717356000	5.381189000
C	1.959131000	-1.897232000	5.604724000
C	2.893879000	-1.266880000	4.761414000
C	2.458793000	-0.446732000	3.702834000
H	-2.422108000	4.420037000	3.925384000
H	-3.683217000	6.486441000	3.295508000
H	-4.949954000	6.577870000	1.128880000
H	-4.934096000	4.599576000	-0.418918000
H	-3.632767000	2.541768000	0.193040000
H	-4.807290000	1.566765000	3.309773000
H	-5.983784000	0.873441000	5.396284000
H	-4.667751000	0.386639000	7.483862000

H	-2.166095000	0.631194000	7.465139000
H	-0.987717000	1.307148000	5.390360000
H	2.507397000	2.878041000	2.585973000
H	4.574374000	3.477040000	1.315692000
H	5.176159000	2.226573000	-0.776396000
H	3.689727000	0.378645000	-1.606199000
H	1.598007000	-0.202239000	-0.352342000
H	-0.928742000	-0.796352000	4.118289000
H	-0.158697000	-2.213873000	6.028538000
H	2.305369000	-2.535586000	6.431409000
H	3.972123000	-1.412475000	4.925909000
H	3.197184000	0.041046000	3.051813000
H	-1.841422000	1.510962000	0.181445000
H	-0.589374000	0.893742000	-0.065807000
H	-0.817372000	-2.878237000	-1.915257000
H	0.523477000	-2.140845000	-0.996110000
H	-0.427937000	-1.146166000	-2.146221000
H	-3.807488000	-1.198194000	-2.297162000
H	-3.987006000	0.434912000	-1.586654000
H	-2.464427000	-0.020560000	-2.417028000
H	-5.535258000	-1.284372000	0.698775000
H	-5.027242000	-0.327961000	2.118198000
H	-5.074592000	0.431340000	0.495629000
H	-3.864194000	-2.811372000	3.055777000
H	-2.217405000	-2.573062000	3.700298000
H	-3.400288000	-1.225171000	3.728754000
H	-0.253335000	-2.934582000	2.717175000
H	0.698538000	-2.659226000	1.230624000
H	-0.467016000	-4.013763000	1.304301000
H	0.037701000	2.557425000	3.982311000
H	-0.014591000	3.221510000	2.300421000

73

(p-H-POCOP)IrH2---CH2Cl2 halogen-bonded tol, E = -2758.838475

C	-0.210377000	1.112965000	-3.708144000
C	-0.187988000	1.187445000	-2.299397000
C	-0.179222000	0.019197000	-1.494187000
C	-0.188751000	-1.243900000	-2.140205000
C	-0.211260000	-1.352396000	-3.547180000
C	-0.222506000	-0.164270000	-4.307798000
H	-0.216115000	2.029132000	-4.315189000
H	-0.217124000	-2.339684000	-4.030033000
H	-0.239853000	-0.235993000	-5.406599000
O	-0.169174000	-2.390676000	-1.375411000
O	-0.169600000	2.425168000	-1.688876000
P	-0.102956000	-2.119941000	0.347863000
P	-0.152896000	2.372744000	0.053818000
Ir	-0.151079000	0.154187000	0.579083000
H	0.776186000	0.250071000	1.868554000
H	-0.828709000	0.242113000	2.042562000
C	1.534873000	-2.995052000	0.723968000
C	-1.647445000	-3.102204000	0.840444000
C	1.445020000	3.336581000	0.373503000

C	-1.745533000	3.348139000	0.375459000
C	1.863790000	-2.805506000	2.219607000
H	2.875776000	-3.217820000	2.416810000
H	1.861572000	-1.735255000	2.497540000
H	1.152591000	-3.326519000	2.885027000
C	-1.613442000	-3.337813000	2.363141000
H	-1.396463000	-2.406859000	2.921064000
H	-2.603718000	-3.714288000	2.695195000
H	-0.857921000	-4.094449000	2.647257000
C	2.583739000	-2.237080000	-0.126230000
H	2.406756000	-2.376270000	-1.209452000
H	2.559986000	-1.150844000	0.091671000
H	3.593658000	-2.628158000	0.116844000
C	1.535926000	-4.486467000	0.344217000
H	2.571363000	-4.878930000	0.428757000
H	0.899382000	-5.084808000	1.022769000
H	1.195550000	-4.641807000	-0.697309000
C	-1.815708000	-4.428085000	0.073263000
H	-1.060936000	-5.180989000	0.359372000
H	-2.815344000	-4.849559000	0.310645000
H	-1.758320000	-4.268782000	-1.019585000
C	-2.821968000	-2.158702000	0.480628000
H	-2.745350000	-1.199007000	1.028197000
H	-2.840173000	-1.932685000	-0.603581000
H	-3.779513000	-2.652601000	0.749007000
C	1.695556000	3.374653000	1.896691000
H	1.661838000	2.358006000	2.333832000
H	2.702311000	3.803017000	2.084832000
H	0.958179000	4.005721000	2.426888000
C	-1.813930000	3.692040000	1.877179000
H	-2.832253000	4.061031000	2.120528000
H	-1.611609000	2.801784000	2.505800000
H	-1.096150000	4.489168000	2.150317000
C	2.547171000	2.486031000	-0.304725000
H	2.534625000	1.445175000	0.075084000
H	2.413992000	2.453632000	-1.402689000
H	3.537260000	2.936791000	-0.084579000
C	1.443805000	4.756269000	-0.221047000
H	0.773005000	5.436528000	0.336137000
H	2.469969000	5.175698000	-0.156185000
H	1.143434000	4.750677000	-1.286140000
C	-1.896492000	4.609958000	-0.495474000
H	-2.925740000	5.007796000	-0.370808000
H	-1.193394000	5.410093000	-0.204855000
H	-1.744387000	4.377447000	-1.566081000
C	-2.871700000	2.346275000	0.017537000
H	-2.826780000	2.053853000	-1.049564000
H	-2.794594000	1.424346000	0.627179000
H	-3.855832000	2.824221000	0.206342000
H	-0.603325000	-1.154358000	6.815084000
C	0.202782000	-1.459636000	6.125666000
H	1.200982000	-1.267431000	6.555569000

Cl 0.051550000 -3.231809000 5.856475000
Cl 0.047440000 -0.506757000 4.615224000
73
(p-H-POCOP)IrH2---CH2Cl2 dihydrogen-bonded tol, E = -2758.843191

C	-0.431589000	1.121346000	-3.668785000
C	-0.279029000	1.169425000	-2.267093000
C	-0.185212000	-0.013752000	-1.488849000
C	-0.250615000	-1.263775000	-2.157218000
C	-0.400223000	-1.346286000	-3.557623000
C	-0.487990000	-0.143883000	-4.290757000
H	-0.505272000	2.049014000	-4.253487000
H	-0.449155000	-2.324293000	-4.056631000
H	-0.605283000	-0.194620000	-5.384540000
O	-0.175248000	-2.425004000	-1.416348000
O	-0.227018000	2.394630000	-1.635204000
P	0.021210000	-2.183577000	0.299656000
P	-0.035695000	2.313989000	0.095397000
Ir	0.087671000	0.083353000	0.567870000
H	0.877164000	0.202887000	1.986576000
H	-0.831123000	0.134912000	1.845131000
C	1.664673000	-3.097906000	0.538549000
C	-1.501703000	-3.144970000	0.892493000
C	1.556431000	3.329700000	0.246669000
C	-1.609620000	3.222248000	0.622573000
C	2.093942000	-2.947870000	2.014315000
H	3.116774000	-3.361887000	2.136279000
H	2.110611000	-1.881676000	2.312536000
H	1.425927000	-3.494665000	2.705363000
C	-1.357632000	-3.437029000	2.399069000
H	-1.106622000	-2.522440000	2.969514000
H	-2.325246000	-3.816604000	2.788649000
H	-0.590549000	-4.209252000	2.599060000
C	2.663847000	-2.324812000	-0.357506000
H	2.403222000	-2.414565000	-1.428918000
H	2.683021000	-1.247794000	-0.093561000
H	3.681430000	-2.741872000	-0.207714000
C	1.626011000	-4.577984000	0.120601000
H	2.659864000	-4.983559000	0.128093000
H	1.026338000	-5.189366000	0.820931000
H	1.218213000	-4.700355000	-0.900856000
C	-1.747085000	-4.444041000	0.098934000
H	-0.973725000	-5.208677000	0.287996000
H	-2.723350000	-4.869997000	0.412497000
H	-1.788564000	-4.246959000	-0.988202000
C	-2.689119000	-2.178882000	0.658062000
H	-2.581963000	-1.256095000	1.258070000
H	-2.768731000	-1.887341000	-0.407258000
H	-3.630472000	-2.689557000	0.950775000
C	1.761567000	3.703889000	1.728437000
H	1.630220000	2.828473000	2.394456000
H	2.792987000	4.090890000	1.865780000
H	1.061366000	4.498319000	2.050659000

C	-1.692009000	3.218747000	2.163559000
H	-2.668637000	3.649602000	2.468830000
H	-1.624810000	2.194267000	2.572541000
H	-0.896018000	3.826096000	2.631447000
C	2.665059000	2.345349000	-0.203863000
H	2.681233000	1.441819000	0.437927000
H	2.514295000	2.019648000	-1.251741000
H	3.650925000	2.851409000	-0.136140000
C	1.592939000	4.577830000	-0.655938000
H	0.887928000	5.359668000	-0.323614000
H	2.614518000	5.011873000	-0.621067000
H	1.363193000	4.319157000	-1.706379000
C	-1.698639000	4.655379000	0.067922000
H	-2.716392000	5.054184000	0.263232000
H	-0.977605000	5.333655000	0.561490000
H	-1.524934000	4.681078000	-1.024765000
C	-2.763089000	2.363746000	0.047774000
H	-2.758035000	2.366779000	-1.058720000
H	-2.686192000	1.313384000	0.390998000
H	-3.729905000	2.783999000	0.394742000
H	0.022833000	-0.703299000	5.591258000
C	-0.201666000	-0.071824000	4.714871000
H	0.262002000	-0.436736000	3.780252000
Cl	-1.981269000	-0.094728000	4.460324000
Cl	0.438126000	1.572393000	5.027376000

73

(p-H-POCOP)IrH2---CH2Cl2 dihydrogen-bonded-from-top tol, E = -2758.842669

C	-0.880512000	1.127761000	-3.576361000
C	-0.544760000	1.170465000	-2.206753000
C	-0.380931000	-0.014005000	-1.442271000
C	-0.548770000	-1.260653000	-2.099293000
C	-0.884559000	-1.338648000	-3.467142000
C	-1.047484000	-0.134391000	-4.183461000
H	-1.004800000	2.058143000	-4.147907000
H	-1.011351000	-2.315488000	-3.954426000
H	-1.309706000	-0.181263000	-5.251938000
O	-0.372382000	-2.426489000	-1.382867000
O	-0.365461000	2.393959000	-1.596776000
P	0.073784000	-2.195355000	0.282957000
P	0.042156000	2.312795000	0.093334000
Ir	0.113031000	0.074474000	0.574803000
H	1.227090000	0.132524000	1.729749000
H	-0.230993000	0.139767000	2.147885000
C	1.744359000	-3.090135000	0.258712000
C	-1.330036000	-3.174853000	1.093872000
C	1.696173000	3.234474000	0.031803000
C	-1.399408000	3.324578000	0.788404000
C	2.227297000	-3.265348000	1.712799000
H	3.289779000	-3.586642000	1.707145000
H	2.159380000	-2.316007000	2.280647000
H	1.647513000	-4.040322000	2.249584000
C	-1.164523000	-3.074677000	2.625231000

H	-1.001910000	-2.031252000	2.953547000
H	-2.083242000	-3.457279000	3.117271000
H	-0.315052000	-3.681881000	2.989624000
C	2.685749000	-2.116256000	-0.492291000
H	2.338930000	-1.937342000	-1.528529000
H	2.741350000	-1.137017000	0.020817000
H	3.702424000	-2.559811000	-0.537006000
C	1.717570000	-4.437996000	-0.487649000
H	2.761406000	-4.795237000	-0.612603000
H	1.164597000	-5.216569000	0.065922000
H	1.268113000	-4.330755000	-1.492567000
C	-1.417272000	-4.645255000	0.647427000
H	-0.597442000	-5.255016000	1.070027000
H	-2.372972000	-5.075921000	1.014038000
H	-1.401560000	-4.737393000	-0.455115000
C	-2.611303000	-2.427000000	0.648604000
H	-2.532568000	-1.340960000	0.849617000
H	-2.793838000	-2.556310000	-0.434802000
H	-3.482823000	-2.833260000	1.202756000
C	2.133135000	3.548909000	1.476983000
H	2.066952000	2.652643000	2.125668000
H	3.188839000	3.891838000	1.471659000
H	1.522140000	4.355095000	1.925935000
C	-1.281724000	3.340737000	2.327329000
H	-2.219439000	3.743516000	2.760980000
H	-1.126452000	2.326334000	2.738686000
H	-0.446927000	3.979967000	2.670564000
C	2.675951000	2.217513000	-0.603745000
H	2.737781000	1.290823000	-0.001553000
H	2.358883000	1.937118000	-1.626975000
H	3.684582000	2.677201000	-0.665169000
C	1.666049000	4.509728000	-0.832880000
H	1.079771000	5.321474000	-0.368652000
H	2.706038000	4.878188000	-0.957508000
H	1.251428000	4.303326000	-1.837294000
C	-1.490464000	4.756552000	0.231598000
H	-2.460896000	5.199039000	0.540611000
H	-0.689772000	5.406862000	0.630020000
H	-1.446082000	4.767812000	-0.874020000
C	-2.655992000	2.528065000	0.359105000
H	-2.795605000	2.560893000	-0.737807000
H	-2.580535000	1.464801000	0.659274000
H	-3.549913000	2.966787000	0.845536000
H	-2.356619000	0.231658000	2.475232000
C	-2.927768000	0.063789000	3.407649000
Cl	-4.274755000	1.243086000	3.485289000
Cl	-1.770368000	0.228555000	4.770564000
H	-3.359526000	-0.949825000	3.447826000

73

(p-H-POCOP)IrH2---CH2Cl2 halogen-bonded-from-top tol, E = -2758.837837

C	-0.802874000	1.107911000	-3.671930000
C	-0.457066000	1.182853000	-2.306455000

C	-0.285054000	0.018165000	-1.514424000
C	-0.478501000	-1.242575000	-2.135910000
C	-0.824407000	-1.354139000	-3.498698000
C	-0.979868000	-0.168170000	-4.247246000
H	-0.933125000	2.024685000	-4.264025000
H	-0.971187000	-2.342362000	-3.956864000
H	-1.249458000	-0.240716000	-5.312412000
O	-0.336140000	-2.389950000	-1.379566000
O	-0.292471000	2.421928000	-1.719277000
P	0.100657000	-2.114858000	0.282360000
P	0.139609000	2.377477000	-0.034641000
Ir	0.253756000	0.153670000	0.490722000
H	1.074829000	0.255428000	1.913541000
H	-0.650769000	0.247484000	1.761784000
C	1.734890000	-3.078166000	0.297819000
C	-1.342039000	-3.004149000	1.124823000
C	1.789410000	3.304686000	-0.148918000
C	-1.287363000	3.393712000	0.677755000
C	2.168150000	-3.294867000	1.761366000
H	3.218715000	-3.653108000	1.781065000
H	2.116304000	-2.352557000	2.342672000
H	1.546019000	-4.058749000	2.266193000
C	-1.208862000	-2.820027000	2.651777000
H	-1.141395000	-1.749801000	2.923254000
H	-2.105893000	-3.251182000	3.143807000
H	-0.323436000	-3.340190000	3.061214000
C	2.728441000	-2.117637000	-0.403192000
H	2.416497000	-1.900018000	-1.443356000
H	2.805097000	-1.155709000	0.143026000
H	3.733032000	-2.589203000	-0.433763000
C	1.691324000	-4.409472000	-0.476235000
H	2.724163000	-4.810597000	-0.551186000
H	1.074157000	-5.172812000	0.028872000
H	1.302469000	-4.263340000	-1.501100000
C	-1.433599000	-4.496397000	0.757821000
H	-0.613339000	-5.083816000	1.211182000
H	-2.389776000	-4.905603000	1.147039000
H	-1.418517000	-4.646967000	-0.338373000
C	-2.605849000	-2.264762000	0.622403000
H	-2.557192000	-1.183435000	0.852623000
H	-2.734297000	-2.381778000	-0.469959000
H	-3.496728000	-2.700408000	1.121647000
C	2.221686000	3.719406000	1.271583000
H	2.151591000	2.870366000	1.980632000
H	3.278075000	4.059725000	1.246317000
H	1.610398000	4.556836000	1.659078000
C	-1.148542000	3.417488000	2.215205000
H	-2.066100000	3.861401000	2.654142000
H	-1.027249000	2.396896000	2.624731000
H	-0.286996000	4.025505000	2.547966000
C	2.768723000	2.239969000	-0.703655000
H	2.829834000	1.364183000	-0.026247000

H	2.453941000	1.881722000	-1.703315000
H	3.780601000	2.686865000	-0.798062000
C	1.765748000	4.514425000	-1.102429000
H	1.156910000	5.348257000	-0.711604000
H	2.803827000	4.888306000	-1.228517000
H	1.377119000	4.230816000	-2.098157000
C	-1.364500000	4.822923000	0.111362000
H	-2.315186000	5.290527000	0.443768000
H	-0.536887000	5.458382000	0.478571000
H	-1.352517000	4.822210000	-0.995253000
C	-2.559553000	2.605816000	0.280721000
H	-2.708923000	2.611621000	-0.815428000
H	-2.501673000	1.553699000	0.618135000
H	-3.440957000	3.080395000	0.759358000
C	-4.112214000	-0.844553000	3.906584000
Cl	-5.650872000	-0.348695000	4.678354000
H	-4.350835000	-1.424018000	2.999224000
Cl	-3.092252000	0.549888000	3.411506000
H	-3.536422000	-1.443892000	4.632035000

73

(p-H-POCOP)IrH(CH2Cl2)H tol, E = -2758.839249

C	-0.255346000	1.089963000	-3.719145000
C	-0.286667000	1.165807000	-2.313362000
C	-0.216784000	0.013589000	-1.492991000
C	-0.176557000	-1.240551000	-2.148808000
C	-0.142422000	-1.353653000	-3.552261000
C	-0.176656000	-0.178336000	-4.330202000
H	-0.298870000	2.013906000	-4.313219000
H	-0.099601000	-2.349808000	-4.015226000
H	-0.148854000	-0.252091000	-5.427795000
O	-0.197546000	-2.396787000	-1.379382000
O	-0.409663000	2.407754000	-1.704889000
P	-0.126992000	-2.143248000	0.314391000
P	-0.218128000	2.391381000	0.000568000
Ir	-0.337729000	0.145083000	0.515127000
H	1.293252000	0.205529000	0.894723000
H	-1.968161000	0.093592000	0.185873000
C	1.606652000	-2.865156000	0.670521000
C	-1.511810000	-3.331756000	0.864610000
C	1.505876000	3.204213000	0.125090000
C	-1.603378000	3.601832000	0.491653000
C	2.013940000	-2.484705000	2.108889000
H	3.057176000	-2.817429000	2.290892000
H	1.975119000	-1.387178000	2.247922000
H	1.374929000	-2.973640000	2.869585000
C	-1.353884000	-3.610105000	2.373558000
H	-1.312028000	-2.668419000	2.955623000
H	-2.235430000	-4.181019000	2.732989000
H	-0.452494000	-4.209753000	2.600537000
C	2.558268000	-2.166664000	-0.329908000
H	2.357612000	-2.488593000	-1.368570000
H	2.457887000	-1.066252000	-0.273173000

H	3.603320000	-2.438880000	-0.074466000
C	1.706341000	-4.387388000	0.462584000
H	2.770831000	-4.689244000	0.556313000
H	1.135729000	-4.956282000	1.220129000
H	1.360746000	-4.683184000	-0.546073000
C	-1.540683000	-4.645180000	0.053568000
H	-0.686978000	-5.306791000	0.273665000
H	-2.467892000	-5.198020000	0.313142000
H	-1.556676000	-4.439823000	-1.033052000
C	-2.843201000	-2.585369000	0.621142000
H	-2.913210000	-1.664868000	1.227456000
H	-2.954588000	-2.295347000	-0.441512000
H	-3.682366000	-3.260844000	0.889484000
C	2.012874000	3.075960000	1.574759000
H	2.020153000	2.016398000	1.889177000
H	3.050736000	3.466763000	1.630305000
H	1.398500000	3.648685000	2.294092000
C	-1.325022000	4.109484000	1.922924000
H	-2.212749000	4.666827000	2.288505000
H	-1.139641000	3.274096000	2.626957000
H	-0.460152000	4.797851000	1.960658000
C	2.421961000	2.375947000	-0.807650000
H	2.363556000	1.297174000	-0.568782000
H	2.155396000	2.518983000	-1.871226000
H	3.469443000	2.712458000	-0.662766000
C	1.532389000	4.672215000	-0.339028000
H	0.996596000	5.345122000	0.355909000
H	2.588920000	5.011883000	-0.376500000
H	1.105792000	4.786504000	-1.353705000
C	-1.757852000	4.775602000	-0.499426000
H	-2.667081000	5.350225000	-0.223862000
H	-0.904402000	5.472603000	-0.477872000
H	-1.884724000	4.406969000	-1.534418000
C	-2.920202000	2.792293000	0.481399000
H	-3.104429000	2.332597000	-0.508589000
H	-2.913230000	1.980198000	1.229781000
H	-3.760117000	3.481100000	0.710739000
H	-0.589371000	-0.114950000	5.168087000
C	0.001359000	-0.208906000	4.240646000
H	0.279737000	-1.251039000	4.015518000
Cl	-1.124304000	0.326149000	2.896719000
Cl	1.470989000	0.771582000	4.367727000

73

(p-H-POCOP)IrH2(CH2Cl2)-iso1 tol, E = -2758.837133

C	-0.672956000	1.091817000	-3.665240000
C	-0.447818000	1.152484000	-2.273734000
C	-0.127198000	0.007408000	-1.514318000
C	-0.116667000	-1.235376000	-2.184974000
C	-0.333771000	-1.343573000	-3.572945000
C	-0.600740000	-0.164680000	-4.302478000
H	-0.905428000	2.006669000	-4.229218000
H	-0.306441000	-2.326875000	-4.064230000

H	-0.772097000	-0.229397000	-5.388143000
O	0.098875000	-2.391406000	-1.436387000
O	-0.552463000	2.377879000	-1.625088000
P	0.029466000	-2.144820000	0.270832000
P	-0.180075000	2.364767000	0.061097000
Ir	0.244960000	0.144701000	0.544857000
H	0.445311000	0.248621000	2.207398000
H	-1.251279000	0.095552000	0.897780000
C	1.370305000	-3.379641000	0.846980000
C	-1.729113000	-2.829488000	0.602348000
C	1.228659000	3.653271000	0.054772000
C	-1.797021000	3.124351000	0.734033000
C	1.750505000	-2.964818000	2.287123000
H	2.559451000	-3.631710000	2.652756000
H	2.106657000	-1.920202000	2.330101000
H	0.893947000	-3.058671000	2.982444000
C	-1.938970000	-2.909207000	2.127449000
H	-1.728773000	-1.934459000	2.611326000
H	-2.993963000	-3.181105000	2.341448000
H	-1.294337000	-3.677635000	2.596178000
C	2.573761000	-3.193620000	-0.102441000
H	2.342225000	-3.559417000	-1.119276000
H	2.868969000	-2.137433000	-0.183043000
H	3.436349000	-3.766646000	0.297598000
C	0.980299000	-4.869280000	0.828497000
H	1.877666000	-5.462018000	1.106106000
H	0.190137000	-5.109852000	1.562869000
H	0.657213000	-5.202238000	-0.174907000
C	-1.976711000	-4.194007000	-0.080937000
H	-1.489632000	-5.033915000	0.437714000
H	-3.068026000	-4.394824000	-0.077317000
H	-1.638332000	-4.177843000	-1.134292000
C	-2.742066000	-1.835112000	-0.014511000
H	-2.726971000	-0.853173000	0.485774000
H	-2.556153000	-1.670609000	-1.092710000
H	-3.760730000	-2.259388000	0.101701000
C	1.933561000	3.592795000	1.427671000
H	2.294162000	2.575184000	1.657914000
H	2.807178000	4.278480000	1.415412000
H	1.265748000	3.914305000	2.248721000
C	-1.547669000	3.602315000	2.179169000
H	-2.515950000	3.885204000	2.642638000
H	-1.095425000	2.798988000	2.794909000
H	-0.888518000	4.490127000	2.217587000
C	2.176155000	3.180937000	-1.073558000
H	2.312984000	2.087703000	-1.069405000
H	1.772058000	3.450771000	-2.066410000
H	3.167063000	3.665365000	-0.949982000
C	0.809699000	5.102675000	-0.251843000
H	0.214673000	5.553642000	0.563755000
H	1.730220000	5.714397000	-0.364640000
H	0.241825000	5.173765000	-1.198274000

C	-2.331909000	4.268032000	-0.156386000
H	-3.337115000	4.558753000	0.213914000
H	-1.698394000	5.168419000	-0.130949000
H	-2.433748000	3.937679000	-1.206924000
C	-2.867112000	2.008654000	0.742522000
H	-2.955070000	1.516159000	-0.245289000
H	-2.647605000	1.238746000	1.504745000
H	-3.849643000	2.460276000	0.991638000
H	4.503377000	1.225308000	-0.677706000
C	4.084890000	0.205835000	-0.631070000
H	4.843080000	-0.549315000	-0.360734000
Cl	2.862007000	0.211372000	0.758750000
Cl	3.346640000	-0.208398000	-2.184238000

73

(p-H-POCOP)IrH2(CH2Cl2)-iso2 tol, E = -2758.834485

C	-0.623625000	1.090182000	-3.639319000
C	-0.404673000	1.172196000	-2.247487000
C	-0.044874000	0.045039000	-1.477327000
C	0.015521000	-1.202424000	-2.137344000
C	-0.195666000	-1.331073000	-3.525426000
C	-0.503402000	-0.168740000	-4.265207000
H	-0.891601000	1.989767000	-4.211999000
H	-0.137596000	-2.317664000	-4.007303000
H	-0.669495000	-0.248820000	-5.350617000
O	0.245846000	-2.345540000	-1.371017000
O	-0.569806000	2.394679000	-1.607642000
P	0.078649000	-2.080451000	0.329239000
P	-0.210614000	2.412885000	0.083350000
Ir	0.261219000	0.206807000	0.601266000
H	0.441636000	0.338127000	2.253214000
H	-1.236821000	0.144989000	0.957582000
C	1.403738000	-3.291057000	0.991833000
C	-1.691142000	-2.765889000	0.569960000
C	1.151012000	3.761885000	0.090617000
C	-1.864030000	3.116072000	0.732904000
C	1.735721000	-2.801263000	2.419893000
H	2.601819000	-3.373024000	2.814644000
H	1.967255000	-1.721879000	2.446670000
H	0.883084000	-2.961070000	3.108916000
C	-2.015458000	-2.720804000	2.076512000
H	-1.879698000	-1.699664000	2.484494000
H	-3.071677000	-3.022878000	2.237191000
H	-1.376983000	-3.414172000	2.658571000
C	2.627861000	-3.155566000	0.056712000
H	2.428542000	-3.629967000	-0.921785000
H	2.900405000	-2.105138000	-0.140157000
H	3.495580000	-3.665769000	0.525334000
C	1.022104000	-4.781856000	1.048636000
H	1.911632000	-5.351163000	1.393105000
H	0.207904000	-4.981319000	1.768836000
H	0.735058000	-5.178028000	0.057642000
C	-1.878692000	-4.185647000	-0.011399000

H	-1.471421000	-4.976617000	0.637600000
H	-2.966000000	-4.378818000	-0.120517000
H	-1.419710000	-4.269125000	-1.015025000
C	-2.652513000	-1.838507000	-0.211984000
H	-2.582519000	-0.786270000	0.106753000
H	-2.454147000	-1.874565000	-1.299717000
H	-3.691065000	-2.188428000	-0.037554000
C	1.911550000	3.617275000	1.426040000
H	2.309514000	2.598440000	1.564926000
H	2.760473000	4.333091000	1.443721000
H	1.261818000	3.841607000	2.294057000
C	-1.657545000	3.542566000	2.200786000
H	-2.637947000	3.813621000	2.645595000
H	-1.225627000	2.714112000	2.797307000
H	-0.994672000	4.423823000	2.292695000
C	2.073908000	3.444787000	-1.110760000
H	2.382471000	2.387017000	-1.149757000
H	1.572679000	3.687045000	-2.065739000
H	2.992892000	4.061966000	-1.026325000
C	0.670283000	5.216034000	-0.073057000
H	0.064107000	5.564588000	0.783091000
H	1.566328000	5.870337000	-0.128108000
H	0.095960000	5.358722000	-1.007046000
C	-2.403163000	4.277208000	-0.132873000
H	-3.439290000	4.505461000	0.192898000
H	-1.816538000	5.202868000	-0.031255000
H	-2.434794000	3.992632000	-1.201439000
C	-2.913354000	1.982001000	0.672513000
H	-2.967753000	1.527695000	-0.335746000
H	-2.703260000	1.186343000	1.410009000
H	-3.909325000	2.408334000	0.912349000
H	4.961096000	0.413550000	1.203480000
C	4.004160000	-0.046771000	1.502536000
H	4.066228000	-1.146202000	1.560512000
Cl	3.504515000	0.592352000	3.073925000
Cl	2.850022000	0.340628000	0.136251000

82

"long"-[Cp*Ru(dppm)(H2)]+ --- Cl-CH2Cl in CH_2Cl_2, E = -3092,72675

Ru	-1.531201000	-0.049740000	0.965214000
C	-1.453798000	-2.000408000	-0.159780000
C	-2.763361000	-1.390769000	-0.311644000
C	-3.374703000	-1.303604000	0.998886000
C	-2.442508000	-1.876699000	1.965839000
C	-1.274245000	-2.321966000	1.240361000
C	-0.517397000	-2.382775000	-1.269922000
C	-3.418430000	-1.029272000	-1.614026000
C	-4.798612000	-0.917712000	1.277553000
C	-2.745822000	-2.120458000	3.416311000
C	-0.115362000	-3.093976000	1.794752000
C	-0.176582000	2.163664000	2.756246000
P	-1.949367000	1.724621000	2.385442000
C	-2.743427000	3.246825000	1.729953000

C	-2.426287000	4.499952000	2.297763000
C	-3.005453000	5.669636000	1.777032000
C	-3.917213000	5.590988000	0.705429000
C	-4.250297000	4.339511000	0.153730000
C	-3.659154000	3.167836000	0.660999000
C	-2.837969000	1.502049000	3.981750000
C	-4.250235000	1.418087000	3.925642000
C	-4.993117000	1.165798000	5.090915000
C	-4.335939000	1.002199000	6.326771000
C	-2.935100000	1.114360000	6.392940000
C	-2.186852000	1.366893000	5.227110000
P	0.468524000	0.654503000	1.863487000
C	1.873578000	1.214931000	0.828689000
C	2.854985000	2.062954000	1.391576000
C	3.943367000	2.488819000	0.610603000
C	4.059836000	2.064467000	-0.728797000
C	3.086456000	1.213233000	-1.287568000
C	1.992544000	0.789727000	-0.510282000
C	1.265214000	-0.404527000	3.135796000
C	0.518257000	-0.754827000	4.283104000
C	1.062503000	-1.615248000	5.249961000
C	2.351882000	-2.154681000	5.066160000
C	3.091030000	-1.824889000	3.915055000
C	2.554383000	-0.949681000	2.951040000
H	-1.716000000	4.568343000	3.135205000
H	-2.740194000	6.647322000	2.206700000
H	-4.366946000	6.509181000	0.298330000
H	-4.963615000	4.274061000	-0.681499000
H	-3.893753000	2.190205000	0.213910000
H	-4.771779000	1.563851000	2.969089000
H	-6.089991000	1.100672000	5.033638000
H	-4.917491000	0.801068000	7.238813000
H	-2.415582000	1.011210000	7.357363000
H	-1.097247000	1.471089000	5.308792000
H	2.767773000	2.396275000	2.436174000
H	4.697105000	3.161636000	1.045583000
H	4.909886000	2.403228000	-1.339781000
H	3.174084000	0.883367000	-2.333323000
H	1.221153000	0.136317000	-0.943549000
H	-0.504075000	-0.376774000	4.407194000
H	0.473009000	-1.875990000	6.141729000
H	2.776536000	-2.836510000	5.818078000
H	4.094680000	-2.249494000	3.762717000
H	3.139866000	-0.697314000	2.056000000
H	-2.043317000	1.117808000	0.012699000
H	-0.753427000	0.612658000	-0.260094000
H	-0.743736000	-3.410893000	-1.625118000
H	0.535539000	-2.372349000	-0.931433000
H	-0.609989000	-1.698979000	-2.133867000
H	-3.939320000	-1.916766000	-2.032315000
H	-4.169215000	-0.228353000	-1.481932000
H	-2.677184000	-0.690051000	-2.361400000

H	-5.471461000	-1.769636000	1.040738000
H	-4.947035000	-0.664026000	2.341349000
H	-5.123365000	-0.056977000	0.663732000
H	-3.431489000	-2.988171000	3.520387000
H	-1.829859000	-2.347185000	3.989777000
H	-3.239514000	-1.248298000	3.883416000
H	-0.007508000	-2.957496000	2.883905000
H	0.838167000	-2.803445000	1.316875000
H	-0.272474000	-4.175840000	1.598213000
H	0.121016000	2.270274000	3.815145000
H	0.108725000	3.078334000	2.202144000
H	1.928269000	3.799558000	-0.697459000
C	1.079872000	4.492049000	-0.570457000
H	1.087500000	5.304392000	-1.316669000
Cl	-0.436402000	3.557850000	-0.764072000
Cl	1.220066000	5.240570000	1.059055000

82

"long"-[Cp*Ru(dppm)(H2)]+ --- H-CHCl2 in CH_2Cl_2, E = -3092,723151

Ru	-1.546613000	0.144520000	1.013886000
C	-1.545592000	-1.702616000	-0.281004000
C	-2.788908000	-0.968540000	-0.455018000
C	-3.477245000	-0.949129000	0.816748000
C	-2.664590000	-1.690501000	1.778729000
C	-1.482911000	-2.159513000	1.090531000
C	-0.588281000	-2.082594000	-1.373776000
C	-3.317270000	-0.437164000	-1.756584000
C	-4.876165000	-0.460904000	1.058638000
C	-3.119351000	-2.064632000	3.161418000
C	-0.415744000	-3.063234000	1.635201000
C	-0.262541000	2.321220000	2.873362000
P	-2.025694000	1.820950000	2.529761000
C	-2.939708000	3.324957000	2.009818000
C	-2.895237000	4.473375000	2.833442000
C	-3.583200000	5.636240000	2.447434000
C	-4.330286000	5.651332000	1.251572000
C	-4.384779000	4.503921000	0.438516000
C	-3.685221000	3.342215000	0.814349000
C	-2.811076000	1.433786000	4.144019000
C	-4.225402000	1.414105000	4.207529000
C	-4.875029000	1.021187000	5.390161000
C	-4.121146000	0.650610000	6.521334000
C	-2.715236000	0.692860000	6.469675000
C	-2.060088000	1.085282000	5.287843000
P	0.405734000	0.767654000	2.076880000
C	1.866808000	1.236922000	1.077907000
C	2.570699000	2.441021000	1.293925000
C	3.660537000	2.771350000	0.467077000
C	4.058544000	1.899189000	-0.563130000
C	3.367667000	0.688751000	-0.769531000
C	2.270548000	0.359575000	0.045085000
C	1.104073000	-0.249336000	3.439605000
C	0.214280000	-1.004294000	4.233187000

C	0.693274000	-1.756876000	5.318292000
C	2.072204000	-1.773222000	5.605540000
C	2.965858000	-1.032667000	4.807159000
C	2.487667000	-0.268659000	3.726209000
H	-2.321849000	4.459647000	3.773094000
H	-3.538436000	6.533859000	3.082318000
H	-4.870621000	6.562608000	0.953887000
H	-4.967568000	4.512801000	-0.494623000
H	-3.704232000	2.448267000	0.174355000
H	-4.819782000	1.719442000	3.334421000
H	-5.974540000	1.007841000	5.428417000
H	-4.630298000	0.340246000	7.445961000
H	-2.119426000	0.422616000	7.354361000
H	-0.963553000	1.118693000	5.271836000
H	2.268134000	3.129792000	2.095433000
H	4.193031000	3.721015000	0.623135000
H	4.902362000	2.168666000	-1.215229000
H	3.672949000	0.006505000	-1.576679000
H	1.709790000	-0.569382000	-0.132864000
H	-0.854514000	-0.998414000	3.988929000
H	-0.010435000	-2.337435000	5.933534000
H	2.452250000	-2.368644000	6.449259000
H	4.044208000	-1.048645000	5.025707000
H	3.190901000	0.307920000	3.108710000
H	-1.847636000	1.455197000	0.159318000
H	-0.613546000	0.746595000	-0.124700000
H	-0.937924000	-3.011900000	-1.872326000
H	0.424153000	-2.283116000	-0.977564000
H	-0.511949000	-1.294768000	-2.145765000
H	-3.872577000	-1.235749000	-2.292879000
H	-4.012411000	0.408000000	-1.597988000
H	-2.499109000	-0.094312000	-2.417034000
H	-5.603128000	-1.255958000	0.786667000
H	-5.040020000	-0.213124000	2.121859000
H	-5.115392000	0.431153000	0.451396000
H	-3.985590000	-2.756074000	3.094345000
H	-2.327177000	-2.584188000	3.729086000
H	-3.446879000	-1.184031000	3.744322000
H	-0.442265000	-3.121789000	2.736253000
H	0.595842000	-2.729665000	1.336740000
H	-0.560400000	-4.088824000	1.234985000
H	0.034304000	2.534073000	3.916709000
H	-0.032039000	3.190461000	2.228160000
H	1.030605000	2.322897000	-1.127219000
C	0.536574000	3.178768000	-1.614835000
H	-0.198112000	2.858965000	-2.372959000
Cl	-0.350107000	4.098112000	-0.346939000
Cl	1.796401000	4.157982000	-2.428344000

82

"short"-[Cp*Ru(dppm)(H2)]+ --- Cl-CH2Cl in CH_2Cl_2, E = -3092,726692

Ru	-1.650537000	-0.024275000	1.042150000
C	-1.598345000	-1.959886000	-0.098028000

C	-2.914368000	-1.351790000	-0.225956000
C	-3.505131000	-1.268558000	1.089865000
C	-2.551983000	-1.831450000	2.044294000
C	-1.394803000	-2.279454000	1.297909000
C	-0.682868000	-2.340370000	-1.226332000
C	-3.585272000	-0.979459000	-1.517172000
C	-4.923193000	-0.881651000	1.394069000
C	-2.835315000	-2.078969000	3.498261000
C	-0.226505000	-3.047979000	1.837539000
C	-0.309040000	2.192345000	2.836174000
P	-2.081577000	1.758186000	2.447963000
C	-2.858176000	3.274921000	1.759875000
C	-2.551211000	4.537906000	2.310856000
C	-3.111633000	5.698912000	1.751532000
C	-3.995428000	5.601919000	0.658154000
C	-4.319462000	4.340857000	0.123003000
C	-3.746136000	3.177662000	0.668802000
C	-2.989090000	1.555468000	4.035609000
C	-4.402480000	1.504770000	3.970238000
C	-5.158383000	1.265135000	5.129922000
C	-4.513310000	1.081188000	6.369262000
C	-3.110618000	1.159943000	6.444680000
C	-2.349472000	1.399292000	5.284512000
P	0.342831000	0.679952000	1.949348000
C	1.742902000	1.245454000	0.908783000
C	2.732498000	2.088360000	1.465131000
C	3.814944000	2.513043000	0.675379000
C	3.917367000	2.093011000	-0.666704000
C	2.935744000	1.247423000	-1.219462000
C	1.847881000	0.824840000	-0.432992000
C	1.149403000	-0.373081000	3.219597000
C	0.413936000	-0.717789000	4.375944000
C	0.963731000	-1.581910000	5.336529000
C	2.247619000	-2.129162000	5.138253000
C	2.976029000	-1.803404000	3.979127000
C	2.433446000	-0.925684000	3.020886000
H	-1.861891000	4.620642000	3.164405000
H	-2.853350000	6.684230000	2.167814000
H	-4.430177000	6.513453000	0.220909000
H	-5.010816000	4.261384000	-0.729323000
H	-3.970330000	2.191332000	0.235406000
H	-4.914337000	1.666580000	3.011033000
H	-6.256124000	1.226147000	5.065541000
H	-5.105370000	0.890385000	7.276768000
H	-2.599704000	1.040442000	7.411826000
H	-1.258290000	1.477216000	5.372823000
H	2.656361000	2.417666000	2.511853000
H	4.575394000	3.181550000	1.105354000
H	4.762965000	2.430914000	-1.284309000
H	3.012474000	0.920972000	-2.267159000
H	1.070408000	0.174142000	-0.859700000
H	-0.603961000	-0.332365000	4.512343000

H 0.382917000 -1.838639000 6.235117000
H 2.676625000 -2.813513000 5.885411000
H 3.975593000 -2.233598000 3.815915000
H 3.009927000 -0.677421000 2.118885000
H -2.009242000 1.158150000 0.004006000
H -0.944531000 0.713030000 -0.210942000
H -0.924630000 -3.361956000 -1.590453000
H 0.375083000 -2.343842000 -0.903597000
H -0.780938000 -1.646264000 -2.081601000
H -4.113677000 -1.862701000 -1.935071000
H -4.333213000 -0.178359000 -1.370100000
H -2.853385000 -0.637410000 -2.272582000
H -5.599103000 -1.737765000 1.182047000
H -5.049635000 -0.615587000 2.457695000
H -5.263018000 -0.028936000 0.777078000
H -3.514787000 -2.950582000 3.610442000
H -1.910951000 -2.300960000 4.059987000
H -3.327488000 -1.209614000 3.972401000
H -0.095867000 -2.897732000 2.922433000
H 0.717534000 -2.765443000 1.336565000
H -0.390423000 -4.131900000 1.658838000
H -0.023381000 2.295169000 3.898931000
H -0.013568000 3.107996000 2.288814000
H 1.803246000 3.818092000 -0.612080000
C 0.972020000 4.533005000 -0.497647000
H 1.017999000 5.350253000 -1.237065000
Cl -0.565835000 3.644172000 -0.734835000
Cl 1.096045000 5.265200000 1.139866000
82
"short"-[Cp*Ru(dppm)(H2)]+ --- BF4- isomer a in CH_2Cl_2, E = -2557,649423
Ru -1.686282000 1.087952000 1.108111000
C -1.661963000 -0.527979000 -0.434003000
C -2.915101000 0.212835000 -0.518668000
C -3.620275000 0.051154000 0.727409000
C -2.807880000 -0.796577000 1.598996000
C -1.607839000 -1.158243000 0.867266000
C -0.693035000 -0.699491000 -1.569762000
C -3.395953000 0.952473000 -1.734810000
C -5.010448000 0.539815000 1.020754000
C -3.270340000 -1.361874000 2.913390000
C -0.533413000 -2.098322000 1.333168000
C -0.436023000 3.224892000 2.975393000
P -2.200326000 2.673027000 2.700683000
C -3.201398000 4.164204000 2.294780000
C -3.597652000 5.060091000 3.316120000
C -4.317013000 6.222711000 2.989290000
C -4.646003000 6.496722000 1.645779000
C -4.250797000 5.606544000 0.629756000
C -3.531168000 4.440631000 0.951295000
C -2.869789000 2.171991000 4.336694000
C -4.260449000 1.925421000 4.438797000
C -4.812961000 1.455025000 5.641892000

C	-3.983606000	1.226437000	6.758294000
C	-2.603828000	1.486524000	6.667508000
C	-2.047942000	1.961337000	5.464765000
P	0.236058000	1.633789000	2.250184000
C	1.769358000	2.053470000	1.342427000
C	2.544803000	3.186503000	1.667969000
C	3.718747000	3.462350000	0.943252000
C	4.121048000	2.608964000	-0.102448000
C	3.345067000	1.479525000	-0.428721000
C	2.168757000	1.205502000	0.288423000
C	0.822181000	0.613530000	3.667265000
C	-0.112813000	-0.217955000	4.319439000
C	0.270957000	-0.986034000	5.431860000
C	1.600627000	-0.936547000	5.893132000
C	2.541154000	-0.115375000	5.239515000
C	2.157186000	0.659134000	4.128927000
H	-3.345768000	4.847239000	4.365695000
H	-4.622058000	6.918107000	3.786056000
H	-5.210641000	7.407077000	1.392877000
H	-4.497400000	5.817623000	-0.421670000
H	-3.208866000	3.759570000	0.152129000
H	-4.915307000	2.118184000	3.577233000
H	-5.895304000	1.267558000	5.707808000
H	-4.415027000	0.853924000	7.699571000
H	-1.949996000	1.320970000	7.536951000
H	-0.970141000	2.158598000	5.416369000
H	2.235141000	3.858480000	2.481778000
H	4.319654000	4.349450000	1.194745000
H	5.037816000	2.829801000	-0.670049000
H	3.649803000	0.818234000	-1.253900000
H	1.540080000	0.346103000	0.017975000
H	-1.144145000	-0.244247000	3.948279000
H	-0.469732000	-1.624142000	5.936965000
H	1.906825000	-1.541460000	6.760060000
H	3.582246000	-0.078483000	5.594593000
H	2.897172000	1.293775000	3.620714000
H	-1.668731000	2.494313000	0.263538000
H	-0.816361000	1.985278000	0.047113000
H	-1.107575000	-1.402023000	-2.324252000
H	0.270104000	-1.116562000	-1.222405000
H	-0.491579000	0.265169000	-2.070673000
H	-3.766601000	0.233396000	-2.496153000
H	-4.224027000	1.640627000	-1.483819000
H	-2.584980000	1.552131000	-2.185793000
H	-5.759764000	-0.104853000	0.513697000
H	-5.225049000	0.509412000	2.103254000
H	-5.163809000	1.576710000	0.666668000
H	-4.100126000	-2.081761000	2.747840000
H	-2.461411000	-1.908724000	3.430425000
H	-3.646696000	-0.574647000	3.593973000
H	-0.438878000	-2.102788000	2.433402000
H	0.452595000	-1.828424000	0.911340000

H	-0.767759000	-3.134266000	1.007871000
H	-0.113280000	3.547023000	3.983054000
H	-0.245298000	4.021563000	2.229066000
F	0.180387000	2.766841000	-1.765850000
F	-1.796653000	3.978653000	-1.752268000
B	-0.389781000	4.052891000	-1.519605000
F	0.188995000	5.005230000	-2.386459000
F	-0.158034000	4.427500000	-0.165236000

82

"short"-[Cp*Ru(dppm)(H2)]+ --- BF4- isomer b in CH_2Cl_2, E = -2557,648068

Ru	-1.621369000	0.147614000	0.991835000
C	-1.622535000	-1.605931000	-0.392808000
C	-2.889176000	-0.891863000	-0.499938000
C	-3.549661000	-0.946177000	0.780306000
C	-2.692442000	-1.698204000	1.696122000
C	-1.514171000	-2.111236000	0.959232000
C	-0.692877000	-1.890714000	-1.538786000
C	-3.422993000	-0.271897000	-1.760663000
C	-4.940862000	-0.465391000	1.080041000
C	-3.096040000	-2.132951000	3.077919000
C	-0.406836000	-2.987935000	1.468691000
C	-0.308220000	2.407979000	2.676647000
P	-2.076609000	1.838383000	2.486418000
C	-3.087111000	3.291628000	1.981492000
C	-3.293658000	4.360768000	2.884168000
C	-4.036668000	5.483979000	2.481750000
C	-4.585334000	5.541064000	1.183853000
C	-4.387074000	4.474369000	0.287120000
C	-3.636385000	3.352144000	0.684313000
C	-2.722318000	1.483551000	4.169679000
C	-4.118209000	1.299497000	4.319302000
C	-4.657544000	0.936042000	5.564859000
C	-3.809785000	0.755441000	6.676170000
C	-2.423824000	0.955791000	6.537123000
C	-1.880604000	1.321893000	5.291327000
P	0.352534000	0.795332000	1.986267000
C	1.821295000	1.207009000	0.976189000
C	2.647326000	2.308974000	1.286116000
C	3.776088000	2.580255000	0.493466000
C	4.084680000	1.753350000	-0.603299000
C	3.259090000	0.657161000	-0.913992000
C	2.125269000	0.387435000	-0.130026000
C	1.030997000	-0.149364000	3.415452000
C	0.126785000	-0.887558000	4.209366000
C	0.580032000	-1.599978000	5.332322000
C	1.949768000	-1.594097000	5.660762000
C	2.859258000	-0.871820000	4.863273000
C	2.405427000	-0.149430000	3.743494000
H	-2.874718000	4.313083000	3.900815000
H	-4.190512000	6.317854000	3.183379000
H	-5.169931000	6.420210000	0.872950000
H	-4.815096000	4.514463000	-0.725779000

H	-3.469249000	2.516542000	-0.011115000
H	-4.786325000	1.455305000	3.460131000
H	-5.744075000	0.795511000	5.668136000
H	-4.231323000	0.467694000	7.651042000
H	-1.755653000	0.829135000	7.402242000
H	-0.797802000	1.475409000	5.204993000
H	2.409771000	2.958857000	2.141521000
H	4.414614000	3.444798000	0.730702000
H	4.965323000	1.973068000	-1.225903000
H	3.488147000	0.019716000	-1.781324000
H	1.455510000	-0.444522000	-0.385339000
H	-0.934711000	-0.894665000	3.934744000
H	-0.137447000	-2.164336000	5.946989000
H	2.310338000	-2.157484000	6.534732000
H	3.931325000	-0.870771000	5.112306000
H	3.121754000	0.409126000	3.124687000
H	-1.663544000	1.504435000	0.063906000
H	-0.842652000	0.993033000	-0.187439000
H	-1.119231000	-2.683010000	-2.190920000
H	0.290255000	-2.249744000	-1.182386000
H	-0.527308000	-0.990690000	-2.159167000
H	-3.867680000	-1.052747000	-2.413846000
H	-4.210138000	0.473586000	-1.542101000
H	-2.618096000	0.230282000	-2.329156000
H	-5.687818000	-1.205591000	0.720857000
H	-5.096177000	-0.339423000	2.165959000
H	-5.159829000	0.499262000	0.585268000
H	-3.924430000	-2.870735000	3.018479000
H	-2.261471000	-2.619494000	3.613877000
H	-3.453543000	-1.283688000	3.690706000
H	-0.295274000	-2.914292000	2.564587000
H	0.564524000	-2.720525000	1.012257000
H	-0.617646000	-4.049318000	1.218090000
H	0.047531000	2.761808000	3.661963000
H	-0.141799000	3.177823000	1.897834000
B	0.101088000	3.095286000	-1.768766000
F	-0.160681000	1.713668000	-2.054542000
F	1.474405000	3.361989000	-1.947799000
F	-0.273948000	3.383401000	-0.419669000
F	-0.676936000	3.894630000	-2.642975000

82

"short"-[Cp*Ru(dppm)(H2)]+ --- BF4- isomer c in CH_2Cl_2, E = -2557,647602

Ru	-1.519351000	0.224058000	1.053032000
C	-1.563677000	-1.480030000	-0.398700000
C	-2.818398000	-0.738740000	-0.476863000
C	-3.472979000	-0.824464000	0.804137000
C	-2.627654000	-1.621431000	1.690080000
C	-1.463268000	-2.038057000	0.930218000
C	-0.621781000	-1.742047000	-1.539649000
C	-3.374284000	-0.088505000	-1.711904000
C	-4.858822000	-0.348548000	1.130137000
C	-3.053049000	-2.108907000	3.047988000

C	-0.389929000	-2.982553000	1.386092000
C	-0.202776000	2.298063000	2.988390000
P	-1.973947000	1.856539000	2.618005000
C	-2.838865000	3.399717000	2.134895000
C	-2.764218000	4.535284000	2.970659000
C	-3.406613000	5.724388000	2.589564000
C	-4.134754000	5.779928000	1.384792000
C	-4.214737000	4.646453000	0.555623000
C	-3.561067000	3.458088000	0.927222000
C	-2.787063000	1.417556000	4.205361000
C	-4.201442000	1.433569000	4.263415000
C	-4.868343000	0.994697000	5.420299000
C	-4.131595000	0.542367000	6.533124000
C	-2.724640000	0.549547000	6.489174000
C	-2.053386000	0.988064000	5.332768000
P	0.436746000	0.752463000	2.164247000
C	1.920150000	1.167337000	1.170539000
C	2.403183000	2.485291000	1.040827000
C	3.485054000	2.751577000	0.179890000
C	4.094669000	1.708102000	-0.540160000
C	3.618185000	0.387217000	-0.404279000
C	2.529269000	0.117957000	0.440929000
C	1.095601000	-0.300678000	3.521350000
C	0.192397000	-1.103662000	4.248682000
C	0.639281000	-1.873419000	5.336436000
C	2.000645000	-1.852779000	5.696876000
C	2.908055000	-1.055880000	4.970687000
C	2.460736000	-0.278145000	3.886437000
H	-2.193969000	4.493504000	3.910994000
H	-3.334295000	6.614956000	3.232204000
H	-4.633999000	6.714873000	1.088036000
H	-4.777311000	4.689441000	-0.389261000
H	-3.589977000	2.572945000	0.274871000
H	-4.780781000	1.802672000	3.404879000
H	-5.968219000	1.009586000	5.453615000
H	-4.654326000	0.196053000	7.437376000
H	-2.140916000	0.215120000	7.360131000
H	-0.956324000	0.993905000	5.319836000
H	1.934847000	3.314884000	1.583664000
H	3.836757000	3.787483000	0.066328000
H	4.938839000	1.921538000	-1.213373000
H	4.090591000	-0.433046000	-0.965680000
H	2.149324000	-0.910503000	0.535838000
H	-0.863223000	-1.112251000	3.954028000
H	-0.076038000	-2.492717000	5.898438000
H	2.356469000	-2.459855000	6.543034000
H	3.972406000	-1.038094000	5.250132000
H	3.172621000	0.343794000	3.324952000
H	-1.535585000	1.612149000	0.166372000
H	-0.674118000	1.099988000	-0.050997000
H	-0.905310000	-2.677117000	-2.069212000
H	0.419686000	-1.859029000	-1.186842000

H	-0.640062000	-0.917919000	-2.276924000
H	-3.920848000	-0.838229000	-2.323037000
H	-4.084866000	0.720560000	-1.459732000
H	-2.573574000	0.339760000	-2.343553000
H	-5.603830000	-1.113332000	0.821477000
H	-4.982278000	-0.185073000	2.215039000
H	-5.108947000	0.592367000	0.606366000
H	-3.927360000	-2.786650000	2.948618000
H	-2.251530000	-2.680549000	3.548847000
H	-3.354997000	-1.276145000	3.710905000
H	-0.253140000	-2.962174000	2.480925000
H	0.583452000	-2.749691000	0.916004000
H	-0.660038000	-4.020506000	1.096337000
H	0.095452000	2.479371000	4.037501000
H	0.024445000	3.187122000	2.367587000
B	0.061668000	5.060379000	0.367699000
F	-0.369048000	3.735673000	0.056310000
F	1.213040000	5.368429000	-0.401156000
F	0.407563000	5.122158000	1.759176000
F	-0.973818000	5.977754000	0.097562000

82

"short"-[Cp*Ru(dppm)(H2)]+ --- BF4- isomer d in CH_2Cl_2, E = -2557,649315

Ru	-1.731313000	1.044483000	1.013242000
C	-1.619734000	-0.860440000	-0.172816000
C	-2.944289000	-0.273140000	-0.318689000
C	-3.569984000	-0.231526000	0.980615000
C	-2.624550000	-0.784337000	1.949501000
C	-1.442293000	-1.200027000	1.220887000
C	-0.678659000	-1.210024000	-1.290265000
C	-3.593957000	0.112132000	-1.616999000
C	-5.009616000	0.104250000	1.243251000
C	-2.921712000	-1.058391000	3.395937000
C	-0.278632000	-1.960517000	1.781158000
C	-0.471867000	3.206755000	2.924595000
P	-2.222417000	2.782933000	2.465864000
C	-2.992628000	4.330937000	1.837460000
C	-2.801887000	5.536941000	2.552068000
C	-3.418135000	6.716687000	2.099383000
C	-4.234987000	6.695371000	0.949823000
C	-4.432545000	5.492118000	0.246419000
C	-3.807672000	4.309434000	0.687210000
C	-3.173691000	2.534350000	4.022354000
C	-4.573444000	2.364917000	3.899629000
C	-5.360609000	2.112189000	5.035172000
C	-4.758157000	2.034921000	6.307156000
C	-3.372407000	2.240450000	6.436133000
C	-2.577365000	2.495538000	5.301392000
P	0.223067000	1.742817000	2.013879000
C	1.652239000	2.340589000	1.036703000
C	2.552016000	3.268737000	1.611612000
C	3.669434000	3.698864000	0.874451000
C	3.898105000	3.199562000	-0.424196000

C	3.004617000	2.271521000	-0.992604000
C	1.878317000	1.844133000	-0.264194000
C	1.001632000	0.672019000	3.286208000
C	0.284487000	0.376798000	4.467093000
C	0.819242000	-0.508954000	5.416346000
C	2.070068000	-1.117526000	5.188835000
C	2.785836000	-0.828151000	4.012081000
C	2.256887000	0.065658000	3.061879000
H	-2.171251000	5.551872000	3.454414000
H	-3.261308000	7.658162000	2.647459000
H	-4.717186000	7.621457000	0.601695000
H	-5.070646000	5.472663000	-0.649982000
H	-3.947581000	3.365542000	0.137965000
H	-5.050805000	2.447628000	2.913313000
H	-6.447730000	1.979789000	4.926900000
H	-5.372579000	1.833110000	7.197514000
H	-2.899094000	2.214355000	7.429389000
H	-1.509622000	2.705818000	5.439067000
H	2.376433000	3.658100000	2.626331000
H	4.365589000	4.427919000	1.316015000
H	4.775286000	3.538851000	-0.995804000
H	3.181071000	1.881931000	-2.006491000
H	1.168480000	1.126650000	-0.702290000
H	-0.698715000	0.829545000	4.644282000
H	0.255078000	-0.726580000	6.335644000
H	2.486499000	-1.815525000	5.930643000
H	3.763568000	-1.299167000	3.829789000
H	2.821334000	0.287293000	2.145018000
H	-2.005972000	2.269126000	-0.034941000
H	-1.010748000	1.862819000	-0.206103000
H	-0.921710000	-2.214388000	-1.699299000
H	0.370291000	-1.238885000	-0.940868000
H	-0.745116000	-0.483878000	-2.121812000
H	-4.106422000	-0.769088000	-2.059041000
H	-4.352271000	0.903911000	-1.471429000
H	-2.851484000	0.472253000	-2.353540000
H	-5.655408000	-0.739187000	0.917035000
H	-5.196173000	0.267947000	2.318233000
H	-5.335086000	1.004920000	0.689411000
H	-3.528608000	-1.983758000	3.495304000
H	-1.993692000	-1.200811000	3.977833000
H	-3.490810000	-0.231029000	3.858320000
H	-0.159328000	-1.795760000	2.865511000
H	0.670449000	-1.683684000	1.287053000
H	-0.440825000	-3.046990000	1.615811000
H	-0.223928000	3.224881000	3.997385000
H	-0.155820000	4.163494000	2.471054000
F	1.630757000	4.976135000	4.632004000
F	0.568231000	3.387922000	5.952932000
B	0.697483000	4.793663000	5.689590000
F	-0.572653000	5.304526000	5.299643000
F	1.144148000	5.449189000	6.854538000

82

"long"-[Cp*Ru(dppm)(H2)]+ --- BF4- isomer e in CH_2Cl_2, E = -2557,647945

Ru	-1.060386000	1.460423000	2.027937000
C	-1.231780000	-0.593838000	1.109011000
C	-2.551297000	-0.014141000	1.285140000
C	-2.765735000	0.194367000	2.702281000
C	-1.578681000	-0.279127000	3.407932000
C	-0.643968000	-0.769336000	2.420643000
C	-0.654258000	-1.094573000	-0.183642000
C	-3.565556000	0.205054000	0.198901000
C	-4.063140000	0.593251000	3.345177000
C	-1.466740000	-0.411860000	4.900223000
C	0.666724000	-1.449364000	2.684723000
C	0.601870000	3.956050000	2.949002000
P	-1.115506000	3.363013000	3.342159000
C	-2.275202000	4.737727000	2.953388000
C	-2.009649000	6.023296000	3.479842000
C	-2.893789000	7.083443000	3.216797000
C	-4.053176000	6.862357000	2.444807000
C	-4.324247000	5.580575000	1.930632000
C	-3.433198000	4.518879000	2.180355000
C	-1.273972000	3.291949000	5.174449000
C	-2.566681000	3.076238000	5.709833000
C	-2.748840000	2.956389000	7.097649000
C	-1.642479000	3.057785000	7.964961000
C	-0.360623000	3.298424000	7.437051000
C	-0.170542000	3.420014000	6.046753000
P	1.083054000	2.311097000	2.209154000
C	2.111200000	2.653919000	0.734063000
C	3.069667000	3.693709000	0.766198000
C	3.875621000	3.925009000	-0.363588000
C	3.737232000	3.123844000	-1.514538000
C	2.784915000	2.086462000	-1.541807000
C	1.968964000	1.853400000	-0.419650000
C	2.232406000	1.503678000	3.391355000
C	1.727232000	1.134127000	4.656339000
C	2.560631000	0.522129000	5.604012000
C	3.904946000	0.246835000	5.282070000
C	4.405107000	0.591546000	4.013309000
C	3.575329000	1.222556000	3.068109000
H	-1.112372000	6.194917000	4.094349000
H	-2.679347000	8.085594000	3.617685000
H	-4.746195000	7.693487000	2.244558000
H	-5.229564000	5.404385000	1.330436000
H	-3.632487000	3.516776000	1.772055000
H	-3.436450000	3.018311000	5.039714000
H	-3.757891000	2.786710000	7.502483000
H	-1.783048000	2.961185000	9.052098000
H	0.505373000	3.399062000	8.108669000
H	0.837633000	3.620944000	5.662935000
H	3.195542000	4.309683000	1.667758000
H	4.619618000	4.735743000	-0.341538000

H	4.372563000	3.310016000	-2.393820000
H	2.672263000	1.460169000	-2.439567000
H	1.213712000	1.055024000	-0.438457000
H	0.672069000	1.314070000	4.891339000
H	2.156939000	0.250513000	6.591156000
H	4.560300000	-0.240308000	6.020015000
H	5.453444000	0.377551000	3.756044000
H	3.981116000	1.504498000	2.088029000
H	-1.875295000	2.540011000	1.186747000
H	-0.665672000	2.004308000	0.579994000
H	-1.007283000	-2.129514000	-0.381201000
H	0.450420000	-1.124581000	-0.150215000
H	-0.960045000	-0.465761000	-1.040133000
H	-4.153488000	-0.723809000	0.038118000
H	-4.275102000	1.011080000	0.463028000
H	-3.083054000	0.468337000	-0.760795000
H	-4.738623000	-0.286676000	3.411186000
H	-3.906103000	0.966963000	4.371880000
H	-4.590084000	1.374572000	2.766756000
H	-2.153477000	-1.207994000	5.258338000
H	-0.444141000	-0.689537000	5.209737000
H	-1.746399000	0.522822000	5.421338000
H	1.072467000	-1.194383000	3.678381000
H	1.427853000	-1.176489000	1.930151000
H	0.528019000	-2.550451000	2.640079000
H	1.251784000	4.343225000	3.752273000
H	0.535587000	4.704540000	2.135721000
F	3.132112000	5.728166000	3.450916000
F	4.624532000	5.818747000	5.224665000
B	3.864370000	4.952652000	4.402848000
F	4.713555000	4.053129000	3.720507000
F	2.936559000	4.226314000	5.213857000

82

"long"-[Cp*Ru(dppm)(H2)]+ --- BF4- isomer f in CH_2Cl_2, E = -2557,649533

Ru	-1.746609000	1.063660000	0.993414000
C	-1.591687000	-0.878440000	-0.147224000
C	-2.901968000	-0.285519000	-0.344291000
C	-3.568414000	-0.223514000	0.941540000
C	-2.664250000	-0.787870000	1.939190000
C	-1.462566000	-1.209360000	1.255814000
C	-0.611869000	-1.242716000	-1.225649000
C	-3.517207000	0.070479000	-1.667554000
C	-5.015612000	0.120550000	1.150436000
C	-2.999880000	-1.044914000	3.379944000
C	-0.322666000	-1.975080000	1.855012000
C	-0.467626000	3.226877000	2.896999000
P	-2.218440000	2.797591000	2.457302000
C	-3.009190000	4.347752000	1.860130000
C	-2.797263000	5.542127000	2.588526000
C	-3.431074000	6.727796000	2.177264000
C	-4.286582000	6.724088000	1.056261000
C	-4.505510000	5.532152000	0.340165000

C	-3.863569000	4.343908000	0.739049000
C	-3.152838000	2.529329000	4.021296000
C	-4.548853000	2.327854000	3.904571000
C	-5.326795000	2.067586000	5.044604000
C	-4.718350000	2.013888000	6.314968000
C	-3.336787000	2.249141000	6.437286000
C	-2.550717000	2.512747000	5.298034000
P	0.218678000	1.766304000	1.980025000
C	1.655562000	2.350460000	1.008368000
C	2.557921000	3.269204000	1.594429000
C	3.690296000	3.684792000	0.871699000
C	3.931247000	3.180501000	-0.422621000
C	3.034723000	2.262058000	-1.001586000
C	1.893643000	1.849039000	-0.288269000
C	0.985687000	0.686615000	3.252747000
C	0.264070000	0.397874000	4.432490000
C	0.792968000	-0.486017000	5.386554000
C	2.041632000	-1.100839000	5.164089000
C	2.760616000	-0.819898000	3.987356000
C	2.238117000	0.073100000	3.032713000
H	-2.137716000	5.545303000	3.470076000
H	-3.257127000	7.659806000	2.736145000
H	-4.782344000	7.654518000	0.740338000
H	-5.173790000	5.525521000	-0.534101000
H	-4.022153000	3.410774000	0.177102000
H	-5.030835000	2.390218000	2.918965000
H	-6.410987000	1.910124000	4.941047000
H	-5.325143000	1.806070000	7.209143000
H	-2.859666000	2.239716000	7.429001000
H	-1.486401000	2.743153000	5.431421000
H	2.375250000	3.663885000	2.605856000
H	4.388457000	4.406687000	1.321698000
H	4.820205000	3.508242000	-0.982610000
H	3.220133000	1.868422000	-2.012254000
H	1.182780000	1.139310000	-0.736602000
H	-0.718071000	0.854344000	4.605678000
H	0.225650000	-0.697506000	6.305308000
H	2.453588000	-1.797591000	5.909539000
H	3.736290000	-1.296429000	3.808533000
H	2.806210000	0.289092000	2.116781000
H	-2.278682000	2.246396000	0.070132000
H	-0.915217000	1.753958000	-0.182816000
H	-0.827557000	-2.262591000	-1.610334000
H	0.426524000	-1.244750000	-0.845075000
H	-0.666035000	-0.541755000	-2.079032000
H	-4.018573000	-0.820702000	-2.102118000
H	-4.277612000	0.866001000	-1.559670000
H	-2.755143000	0.414517000	-2.391424000
H	-5.654590000	-0.710504000	0.782330000
H	-5.245528000	0.266857000	2.219589000
H	-5.309984000	1.034083000	0.600415000
H	-3.585363000	-1.984354000	3.475199000

H	-2.087263000	-1.154229000	3.992796000
H	-3.604836000	-0.226478000	3.810558000
H	-0.230985000	-1.802532000	2.940782000
H	0.642190000	-1.713495000	1.384260000
H	-0.491678000	-3.061255000	1.693925000
H	-0.204770000	3.248482000	3.966244000
H	-0.160411000	4.181266000	2.432634000
F	1.650104000	4.998067000	4.563285000
F	0.604929000	3.414057000	5.902795000
B	0.735824000	4.819387000	5.637936000
F	-0.539280000	5.334911000	5.271019000
F	1.206297000	5.472518000	6.794491000

97

"short"-[Cp*Ru(dppm)(H2)]+ --- BF4-*3CH2Cl2 in CH_2Cl_2, E = -5435,336546

Ru	-1.692504000	1.098253000	0.978372000
C	-1.609668000	-0.614939000	-0.457571000
C	-2.880152000	0.083303000	-0.605020000
C	-3.595684000	-0.009560000	0.642666000
C	-2.775049000	-0.780032000	1.575450000
C	-1.557264000	-1.155360000	0.884183000
C	-0.625077000	-0.865716000	-1.564286000
C	-3.386972000	0.703863000	-1.874372000
C	-5.005299000	0.451404000	0.881051000
C	-3.243879000	-1.278933000	2.913807000
C	-0.462497000	-2.029848000	1.423267000
C	-0.468190000	3.306034000	2.782366000
P	-2.228086000	2.730537000	2.525914000
C	-3.285799000	4.169418000	2.082889000
C	-3.602468000	5.145979000	3.056595000
C	-4.381578000	6.259870000	2.703723000
C	-4.856255000	6.401239000	1.383785000
C	-4.550502000	5.425395000	0.417532000
C	-3.767643000	4.308733000	0.765662000
C	-2.889349000	2.303729000	4.189396000
C	-4.269750000	2.005756000	4.289756000
C	-4.827317000	1.618828000	5.519852000
C	-4.013025000	1.529433000	6.666753000
C	-2.644411000	1.843039000	6.577073000
C	-2.083680000	2.232080000	5.345957000
P	0.219708000	1.696631000	2.112955000
C	1.776680000	2.102209000	1.239467000
C	2.680455000	3.055631000	1.760726000
C	3.896881000	3.300479000	1.099288000
C	4.210741000	2.603461000	-0.083550000
C	3.302707000	1.666862000	-0.613289000
C	2.085765000	1.417808000	0.045203000
C	0.771299000	0.688482000	3.556165000
C	-0.218071000	-0.012506000	4.279636000
C	0.127348000	-0.788703000	5.397990000
C	1.475352000	-0.889066000	5.793480000
C	2.470280000	-0.208973000	5.064700000
C	2.124008000	0.578425000	3.950105000

H	-3.229314000	5.041014000	4.085290000
H	-4.603810000	7.031084000	3.455908000
H	-5.455504000	7.281576000	1.108112000
H	-4.909995000	5.534604000	-0.616537000
H	-3.505788000	3.560692000	0.006582000
H	-4.914057000	2.094741000	3.403513000
H	-5.901517000	1.388683000	5.583153000
H	-4.447935000	1.223862000	7.630268000
H	-2.003022000	1.786515000	7.469374000
H	-1.013777000	2.469642000	5.298821000
H	2.432804000	3.615295000	2.674333000
H	4.588144000	4.059776000	1.492387000
H	5.156019000	2.809831000	-0.605909000
H	3.536265000	1.138263000	-1.549599000
H	1.358527000	0.708223000	-0.371131000
H	-1.261296000	0.055458000	3.952374000
H	-0.658145000	-1.320227000	5.956022000
H	1.751893000	-1.503454000	6.663653000
H	3.526553000	-0.291798000	5.362494000
H	2.911186000	1.097650000	3.386970000
H	-1.759350000	2.463477000	0.070220000
H	-0.862718000	2.013554000	-0.104007000
H	-0.987428000	-1.687500000	-2.218871000
H	0.361006000	-1.170627000	-1.167987000
H	-0.487357000	0.028000000	-2.199562000
H	-3.870641000	-0.072794000	-2.504891000
H	-4.135645000	1.490388000	-1.668793000
H	-2.567991000	1.154194000	-2.460924000
H	-5.724589000	-0.263434000	0.426936000
H	-5.232754000	0.509609000	1.959873000
H	-5.192415000	1.445582000	0.433738000
H	-4.061154000	-2.018431000	2.775789000
H	-2.433622000	-1.783964000	3.469051000
H	-3.641973000	-0.462715000	3.545906000
H	-0.395462000	-1.971205000	2.523815000
H	0.523030000	-1.746781000	1.008386000
H	-0.653965000	-3.088933000	1.148635000
H	-0.149680000	3.668008000	3.776860000
H	-0.270821000	4.081523000	2.016622000
F	0.076316000	3.241633000	-1.868727000
F	-2.163325000	3.861560000	-1.968461000
B	-0.843684000	4.309315000	-1.661711000
F	-0.511357000	5.400225000	-2.505945000
F	-0.780531000	4.719573000	-0.299755000
H	2.019405000	4.463598000	-0.583218000
H	-1.953380000	6.426724000	1.103400000
H	0.093646000	3.325017000	-4.457047000
C	-0.893132000	2.991573000	-4.813127000
H	-1.689987000	3.275446000	-4.108404000
Cl	-0.863381000	1.192417000	-4.920209000
Cl	-1.224836000	3.766432000	-6.399172000
C	-1.274635000	7.251605000	1.368477000

H	-0.408901000	7.299686000	0.689168000
Cl	-0.642136000	6.942839000	3.024331000
Cl	-2.178725000	8.798476000	1.258527000
C	2.192750000	5.525089000	-0.816628000
H	1.382316000	5.940850000	-1.433901000
Cl	2.246949000	6.434241000	0.735052000
Cl	3.727635000	5.658785000	-1.741674000

97

"long"-[Cp*Ru(dppm)(H2)]+ --- BF4-*3CH2Cl2 in CH_2Cl_2, E = -5435,34211

Ru	-1.688241000	-0.019728000	0.589598000
C	-1.759342000	-2.065319000	-0.349310000
C	-2.961078000	-1.316238000	-0.675978000
C	-3.665604000	-1.039215000	0.559032000
C	-2.908739000	-1.652004000	1.648817000
C	-1.749035000	-2.291347000	1.080552000
C	-0.812358000	-2.685682000	-1.335845000
C	-3.437155000	-1.006804000	-2.065645000
C	-5.040466000	-0.447475000	0.683474000
C	-3.384019000	-1.742028000	3.070362000
C	-0.746437000	-3.157097000	1.786858000
C	-0.134913000	2.312400000	2.015266000
P	-1.938311000	1.874523000	1.899668000
C	-2.839467000	3.349902000	1.290777000
C	-2.550162000	4.615697000	1.845147000
C	-3.267400000	5.743493000	1.415689000
C	-4.283445000	5.610074000	0.449041000
C	-4.577072000	4.347296000	-0.095989000
C	-3.848973000	3.217059000	0.319054000
C	-2.593114000	1.748791000	3.615635000
C	-3.998264000	1.768064000	3.790181000
C	-4.558264000	1.594446000	5.067374000
C	-3.722109000	1.403711000	6.185340000
C	-2.324443000	1.404509000	6.020091000
C	-1.760167000	1.579718000	4.742662000
P	0.375319000	0.635130000	1.384201000
C	1.807033000	0.844379000	0.267044000
C	2.595574000	2.014454000	0.274706000
C	3.713357000	2.107265000	-0.575608000
C	4.044306000	1.038388000	-1.429435000
C	3.254095000	-0.127771000	-1.440485000
C	2.135204000	-0.224212000	-0.596635000
C	1.094916000	-0.232291000	2.842734000
C	0.211889000	-0.782863000	3.795821000
C	0.709310000	-1.396790000	4.957196000
C	2.099834000	-1.482552000	5.164940000
C	2.986480000	-0.948886000	4.209201000
C	2.489754000	-0.321937000	3.050862000
H	-1.756327000	4.723362000	2.598826000
H	-3.027855000	6.733601000	1.832110000
H	-4.842732000	6.496818000	0.114045000
H	-5.357029000	4.239654000	-0.863354000
H	-4.049567000	2.230910000	-0.121679000

H	-4.656631000	1.933968000	2.924934000
H	-5.651757000	1.610057000	5.189173000
H	-4.160097000	1.265145000	7.185225000
H	-1.663307000	1.272259000	6.889823000
H	-0.668011000	1.584740000	4.637817000
H	2.340735000	2.861170000	0.925958000
H	4.316531000	3.025683000	-0.580314000
H	4.908664000	1.122926000	-2.104048000
H	3.494953000	-0.953586000	-2.125864000
H	1.500916000	-1.120846000	-0.618144000
H	-0.868556000	-0.728903000	3.613813000
H	0.009520000	-1.816014000	5.695858000
H	2.493444000	-1.970603000	6.069364000
H	4.073682000	-1.019106000	4.364917000
H	3.187425000	0.095868000	2.311303000
H	-2.002490000	1.194555000	-0.388116000
H	-0.851606000	0.437538000	-0.682863000
H	-1.243111000	-3.629286000	-1.734393000
H	0.154897000	-2.937996000	-0.863695000
H	-0.613499000	-2.016767000	-2.191980000
H	-4.018794000	-1.864392000	-2.465964000
H	-4.088692000	-0.115622000	-2.089009000
H	-2.589513000	-0.827803000	-2.752229000
H	-5.803945000	-1.255171000	0.684313000
H	-5.158322000	0.118332000	1.625117000
H	-5.272567000	0.227724000	-0.160400000
H	-4.246844000	-2.438557000	3.135467000
H	-2.597382000	-2.124151000	3.745756000
H	-3.721426000	-0.762657000	3.455581000
H	-0.866669000	-3.124836000	2.882671000
H	0.293003000	-2.860674000	1.551282000
H	-0.875578000	-4.210488000	1.460603000
H	0.272067000	2.638265000	2.989539000
H	0.059393000	3.078873000	1.239895000
B	-0.203240000	4.583984000	-1.022989000
F	-0.497261000	3.191602000	-0.994802000
F	0.792139000	4.829603000	-2.014724000
F	0.306626000	4.977647000	0.248898000
F	-1.374191000	5.318595000	-1.325558000
H	-2.770595000	3.724492000	-2.245457000
C	-2.848830000	2.878403000	-2.946505000
Cl	-4.587979000	2.448740000	-3.156058000
Cl	-2.084720000	3.368951000	-4.495311000
H	-2.318285000	1.988752000	-2.572495000
H	2.444277000	5.408154000	-0.687653000
C	3.310630000	5.966761000	-0.297669000
Cl	3.675754000	5.376756000	1.362374000
Cl	4.690001000	5.732103000	-1.428755000
H	3.110218000	7.049466000	-0.230393000
H	0.444144000	2.841725000	-3.450660000
C	1.084531000	1.970498000	-3.254291000
Cl	0.067351000	0.490162000	-3.422423000

Cl	2.447645000	1.987212000	-4.418040000
H	1.489760000	1.986369000	-2.232702000

97

"long"-[Cp*Ru(dppm)(H2)]+ --- BF4-*3CH2Cl2-iso in CH_2Cl_2, E = -5435,342604

Ru	-1.717090000	1.199790000	1.078790000
C	-1.512259000	-0.762191000	-0.016099000
C	-2.841614000	-0.214086000	-0.214107000
C	-3.498562000	-0.141653000	1.077007000
C	-2.569309000	-0.656188000	2.078145000
C	-1.362002000	-1.057291000	1.393216000
C	-0.528297000	-1.118523000	-1.093407000
C	-3.479745000	0.093264000	-1.538753000
C	-4.953486000	0.165060000	1.291500000
C	-2.880290000	-0.886080000	3.528933000
C	-0.195864000	-1.776055000	1.999685000
C	-0.455303000	3.430359000	2.903588000
P	-2.202935000	2.949117000	2.517489000
C	-3.029140000	4.472535000	1.900421000
C	-2.794869000	5.695928000	2.569189000
C	-3.439062000	6.864497000	2.129258000
C	-4.329372000	6.815111000	1.037814000
C	-4.572201000	5.594402000	0.380050000
C	-3.918495000	4.423087000	0.808026000
C	-3.099356000	2.701872000	4.106428000
C	-4.488266000	2.442658000	4.022278000
C	-5.238534000	2.209682000	5.186409000
C	-4.609778000	2.242128000	6.447774000
C	-3.236710000	2.533343000	6.537466000
C	-2.479034000	2.767693000	5.372782000
P	0.252404000	1.965241000	2.011784000
C	1.652870000	2.570582000	1.000313000
C	2.575917000	3.469863000	1.581774000
C	3.677103000	3.916048000	0.832451000
C	3.864571000	3.465085000	-0.489658000
C	2.946202000	2.566886000	-1.066681000
C	1.837736000	2.119797000	-0.322777000
C	1.075902000	0.940686000	3.292227000
C	0.403069000	0.673062000	4.506865000
C	0.996112000	-0.154457000	5.473197000
C	2.255326000	-0.737560000	5.228052000
C	2.922214000	-0.482268000	4.017472000
C	2.338004000	0.356736000	3.050662000
H	-2.102787000	5.741146000	3.423767000
H	-3.234247000	7.818753000	2.634972000
H	-4.831914000	7.733090000	0.697091000
H	-5.267004000	5.552371000	-0.472316000
H	-4.093484000	3.468012000	0.289514000
H	-4.986415000	2.438956000	3.042766000
H	-6.317292000	2.006537000	5.108548000
H	-5.195162000	2.056864000	7.361016000
H	-2.743110000	2.588987000	7.517574000
H	-1.420897000	3.038435000	5.475681000

H	2.432781000	3.827856000	2.610751000
H	4.393583000	4.617218000	1.286138000
H	4.729041000	3.816962000	-1.073027000
H	3.090291000	2.213948000	-2.098925000
H	1.111887000	1.423355000	-0.768019000
H	-0.582834000	1.111862000	4.705221000
H	0.480574000	-0.328464000	6.427559000
H	2.722107000	-1.377112000	5.991139000
H	3.913011000	-0.920361000	3.827838000
H	2.873139000	0.566991000	2.114588000
H	-2.313383000	2.342431000	0.144587000
H	-0.935827000	1.909900000	-0.120780000
H	-0.699616000	-2.159817000	-1.441238000
H	0.513053000	-1.058290000	-0.725605000
H	-0.624423000	-0.450073000	-1.968915000
H	-3.960028000	-0.821484000	-1.947382000
H	-4.261003000	0.869630000	-1.440931000
H	-2.734882000	0.441713000	-2.278267000
H	-5.571700000	-0.690805000	0.945433000
H	-5.179053000	0.325732000	2.359594000
H	-5.278223000	1.058939000	0.726483000
H	-3.419576000	-1.848923000	3.657639000
H	-1.958448000	-0.933887000	4.136176000
H	-3.519476000	-0.084589000	3.941371000
H	-0.095135000	-1.569708000	3.078866000
H	0.756119000	-1.505161000	1.508636000
H	-0.340257000	-2.870292000	1.872535000
H	-0.162083000	3.513106000	3.960882000
H	-0.192500000	4.357973000	2.365271000
F	1.900670000	5.106238000	4.436588000
F	0.655343000	3.743975000	5.844530000
B	1.007176000	5.112340000	5.549621000
F	-0.171296000	5.818323000	5.197711000
F	1.636148000	5.686719000	6.664131000
H	0.681970000	2.686598000	7.650268000
C	0.378133000	2.496427000	8.691680000
H	1.238174000	2.421787000	9.377850000
Cl	-0.513375000	0.930966000	8.750785000
Cl	-0.642715000	3.877506000	9.213046000
H	0.617936000	6.526611000	3.122597000
C	0.771861000	7.185967000	2.253493000
H	1.827085000	7.489317000	2.150994000
Cl	-0.214995000	8.664683000	2.491710000
Cl	0.322475000	6.284864000	0.757188000
H	2.603211000	2.359392000	5.188042000
C	3.534776000	2.711354000	5.655634000
H	3.473328000	3.772696000	5.937892000
Cl	4.868225000	2.527530000	4.466292000
Cl	3.798663000	1.755966000	7.157309000
